# Programmed Delay of a Virulence Circuit Promotes *Salmonella* Pathogenicity

**DOI:** 10.1128/mBio.00291-19

**Published:** 2019-04-09

**Authors:** Jeongjoon Choi, Heeju Kim, Yoonjee Chang, Woongjae Yoo, Dajeong Kim, Sangryeol Ryu

**Affiliations:** a Department of Food and Animal Biotechnology, Research Institute of Agriculture and Life Sciences, Seoul National University, Seoul, South Korea; b Department of Agricultural Biotechnology, Research Institute of Agriculture and Life Sciences, Seoul National University, Seoul, South Korea; c Center for Food and Bioconvergence, Seoul National University, Seoul, South Korea; University of Washington

**Keywords:** PhoP, *Salmonella*, *ptsN*, virulence regulation

## Abstract

Signal transduction systems dictate various cellular behaviors in response to environmental changes. To operate cellular programs appropriately, organisms have sophisticated regulatory factors to optimize the signal response. The PhoP/PhoQ master virulence regulatory system of the intracellular pathogen Salmonella enterica is activated inside acidic macrophage phagosomes. Here we report that *Salmonella* delays the activation of this system inside macrophages using an inhibitory protein, EIIA^Ntr^ (a component of the nitrogen-metabolic phosphotransferase system). We establish that EIIA^Ntr^ directly restrains PhoP binding to its target promoter, thereby negatively controlling the expression of PhoP-activated genes. PhoP furthers its activation by promoting Lon-mediated degradation of EIIA^Ntr^ at acidic pH. These results suggest that *Salmonella* ensures robust activation of its virulence system by suspending the activation of PhoP until a sufficient level of active PhoP is present to overcome the inhibitory effect of EIIA^Ntr^. Our findings reveal how a pathogen precisely and efficiently operates its virulence program during infection.

## Abstract (precis)

To accomplish successful infection, pathogens must operate their virulence programs in a precise, time-sensitive, and coordinated manner. A major question is how pathogens control the timing of virulence gene expression during infection. Here we report that the intracellular pathogen *Salmonella* controls the timing and level of virulence gene expression by using an inhibitory protein, EIIA^Ntr^. A DNA binding master virulence regulator, PhoP, controls various virulence genes inside acidic phagosomes. *Salmonella* decreases EIIA^Ntr^ amounts at acidic pH in a Lon- and PhoP-dependent manner. This, in turn, promotes expression of the PhoP-activated virulence program because EIIA^Ntr^ hampers activation of PhoP-regulated genes by interfering with PhoP binding to DNA. EIIA^Ntr^ enables *Salmonella* to impede the activation of PhoP-regulated gene expression inside macrophages. Our findings suggest that *Salmonella* achieves programmed delay of virulence gene activation by adjusting levels of an inhibitory factor.

## INTRODUCTION

Living cells control gene expression in response to changes in their surroundings through signal transduction systems that detect environmental signals and convert them into cellular processes, including the control of gene expression, thereby altering cellular behavior. To behave appropriately, cells must precisely decide where and when to operate a certain process. Identification of the responding signals of such systems provides understanding about where an organism turns such systems on or off. However, little is known about how an organism decides the timing of system activation (i.e., kinetics of the system). In bacteria, signal responses are primarily mediated by two-component regulatory systems that comprise a signal sensor and a cognate response regulator, which is typically a transcriptional regulator (1). Here we show that an inhibitory factor shapes the activation of the master virulence regulatory two-component system in the intracellular pathogen Salmonella enterica.

Given that intracellular pathogens experience an acidic pH inside the host phagosome (2–4), it is important for them to have a system that can respond to pH changes in order to survive and cause disease inside the host (5–9). The *Salmonella* PhoP/PhoQ two-component system is a master virulence regulatory complex (10, 11) that is activated by acidic pH (12, 13), low Mg^2+^ (14), and certain antimicrobial peptides (15). This system is crucial for *Salmonella* virulence because the lack of either PhoP or PhoQ impairs *Salmonella* pathogenicity (10, 11). Activation of this system by acidic pH is critical for *Salmonella* virulence because the inhibition of acidification of the *Salmonella-*containing vacuole prevents expression of PhoP-activated genes in phagocytic (9, 16) and nonphagocytic (17) cells, limits replication inside macrophages (6, 18), and attenuates virulence in mice (19). Although “turn on” of the PhoP/PhoQ system is necessary for virulence, it is also important to precisely control this system because constant activation of this system renders *Salmonella* avirulent in mice (20).

The *ptsN* gene encodes EIIA^Ntr^, a component of the nitrogen-metabolic phosphotransferase system (PTS) (21, 22). This nitrogen-metabolic PTS lacks a membrane-bound complex that controls the activities of sugar PTSs in response to particular sugar availabilities (21, 22). Recent studies have reported that EIIA^Ntr^ is involved in various cellular functions, including potassium uptake (23, 24), the stringent response (25, 26), and amino sugar homeostasis (27). Moreover, we recently reported that EIIA^Ntr^ promotes virulence by hampering SsrB, a transcriptional regulator of *Salmonella* pathogenicity island 2 (SPI-2) (28). Despite the fact that various regulatory functions of EIIA^Ntr^ have been identified, the regulation of its own expression remains largely unknown.

Here we establish that *Salmonella* alters EIIA^Ntr^ abundance, thereby controlling activation of the PhoP/PhoQ system during infection. Under acidic conditions, *Salmonella* reduces EIIA^Ntr^ amounts by Lon-mediated degradation in a PhoP-dependent manner. EIIA^Ntr^ hampers PhoP binding to its target DNA, thereby decreasing expression of PhoP-activated genes under acidic pH conditions. This double-negative regulation results in an overall positive feedback that furthers activation of the system. Our findings suggest that *Salmonella* ensures the timing and extent of its PhoP/PhoQ-mediated virulence program via regulation of an inhibitory factor during infection.

## RESULTS

### EIIA^Ntr^ amounts decrease upon environmental acidification.

**FIG 1:**
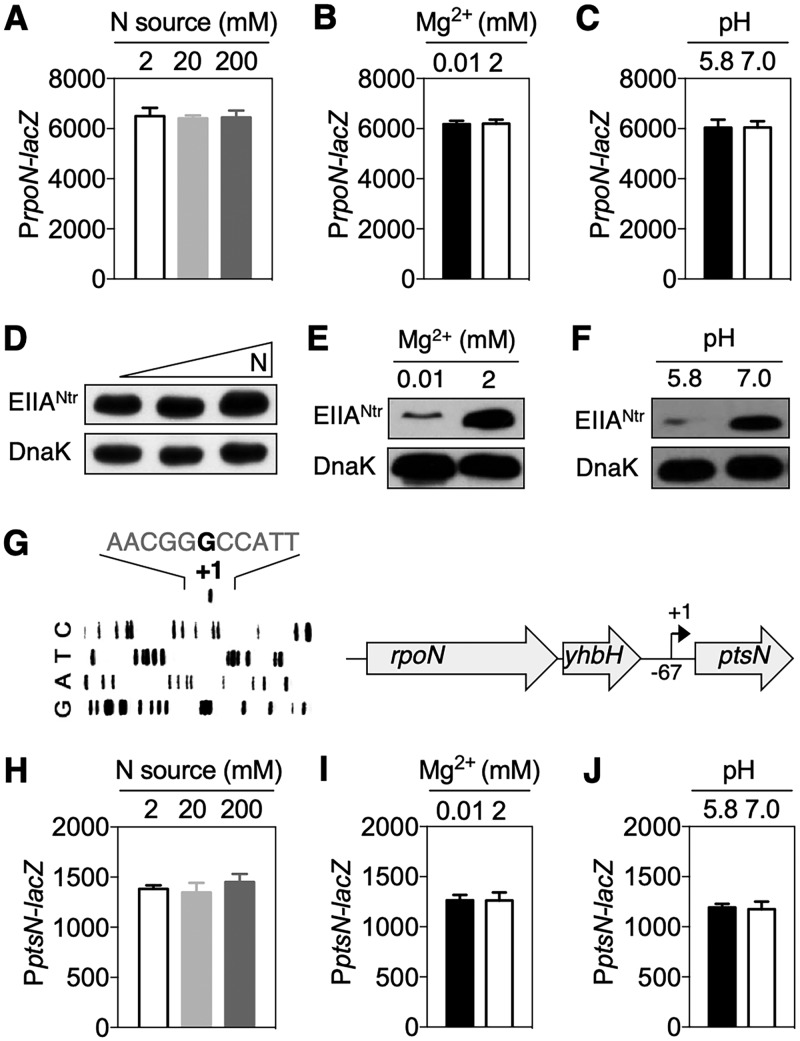
Acidic pH and low Mg^2+^ conditions decrease EIIA^Ntr^ levels. (A to C and H to J) β-Galactosidase activities were determined from wild-type *Salmonella* harboring a plasmid with a p*_rpoN_*-*lacZ* fusion (A to C) or a plasmid with the p*_ptsN_*-*lacZ* fusion (H to J). Bacteria were grown in M9 medium with the indicated variations. The means and SDs from three independent experiments are shown. (D to F) Western blot analysis of crude extracts prepared from *Salmonella* expressing EIIA^Ntr^-FLAG from its normal chromosomal location. Bacteria were grown in M9 medium with the indicated modifications (N, 2, 20, and 200 mM NH_4_Cl; Mg^2+^, 0.01 and 2 mM MgCl_2_; pH, 5.8 and 7.0). Representative results from at least three independent experiments are shown. (G) Primer extension analysis of the *ptsN* gene using total RNA prepared from wild-type *Salmonella* grown in M9 medium. The bold “G” indicates the transcriptional start site (+1) of the *ptsN* gene, located 67 nucleotides upstream from the start codon of the *ptsN* gene. Representative results from at least three independent experiments are shown. A schematic of the *ptsN* promoter with the *rpoN* gene is displayed on the right side.

To investigate the expression of EIIA^Ntr^, we first investigated its transcription levels by measuring β-galactosidase activity produced by a p*_rpoN_-lacZ* fusion given that the *ptsN* gene is located downstream of the *rpoN* gene, forming an operon (21). Although EIIA^Ntr^ is a component of a nitrogen-metabolic PTS (21, 22), transcription levels of *rpoN* remained unaltered by 100-fold changes in the concentration of a nitrogen source (Fig. 1A). We next examined *rpoN* expression at different pH values or concentrations of Mg^2+^, representing environmental conditions that *Salmonella* might encounter during infection (6, 14). However, none of those changes modified the expression of *rpoN* (Fig. 1B and C).

Despite the absence of notable changes in p*_rpoN_-lacZ* expression, we examined EIIA^Ntr^ protein amounts under these conditions. Similar to *rpoN* expression, EIIA^Ntr^ amounts were not responsive to changes in nitrogen source (Fig. 1D). Surprisingly, however, EIIA^Ntr^ abundance was significantly reduced when *Salmonella was* exposed to low-Mg^2+^ or acidic-pH conditions (Fig. 1E and F).

This *rpoN-*independent alteration of EIIA^Ntr^ levels (Fig. 1A to F) raised the possibility that transcription of the *ptsN* gene might not just be from the *rpoN* promoter. Indeed, primer extension analysis indicated the presence of a transcriptional start site 67 nucleotides upstream of the EIIA^Ntr^ start codon (Fig. 1G). Therefore, we investigated the expression of a p*_ptsN_*-*lacZ* transcriptional fusion in bacteria grown under the above-described conditions. However, none of those conditions altered the expression of p*_ptsN_-lacZ* (Fig. 1H to J). Taken together, these results suggest that *Salmonella* probably controls EIIA^Ntr^ levels through posttranscriptional regulatory mechanisms.

### PhoP decreases EIIA^Ntr^ abundance posttranscriptionally.

**FIG 2:**
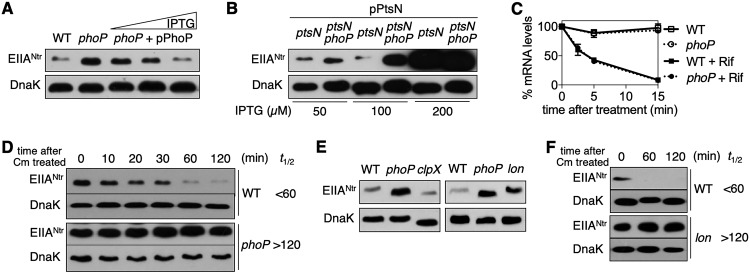
PhoP reduces EIIA^Ntr^ abundance by destabilizing it via Lon protease. (A and B) Western blot analysis of crude extracts prepared from *Salmonella* expressing EIIA^Ntr^-FLAG from the normal chromosomal location (*ptsN*-*FLAG*), an isogenic *phoP* mutant, and the *phoP* mutant harboring a plasmid expressing PhoP under the control of an IPTG-inducible promoter (A) and *Salmonella* strains with deletions of the *ptsN* or *ptsN* and *phoP* genes harboring a plasmid expressing EIIA^Ntr^-FLAG from an IPTG-inducible promoter (B). Bacteria were grown in acidified M9 medium (pH 5.8) with or without IPTG (A, 0, 10, and 100 µM IPTG [from left to right]). Representative results from at least three independent experiments are shown. (C) Stabilities of *ptsN* mRNA were determined from wild-type (WT) and *phoP* mutant *Salmonella*. Bacteria were grown in M9 medium, pH 5.8, then the cultures were split in two and one was treated with 100 µg/ml of rifampin (Rif). Samples were collected at the indicated time points upon treatment. The means and SDs from three independent experiments are shown. (D to F) Western blot analysis of crude extracts prepared from *ptsN*-*FLAG* wild-type and isogenic *phoP* mutant strains (D), *ptsN*-*FLAG* wild-type and isogenic mutants with *phoP*, *clpX*, or *lon* gene deletions (E), and *ptsN*-*FLAG* wild-type and *lon* mutant strains (F). For *ptsN*-*FLAG* wild-type and isogenic *phoP* mutant strains, bacteria were grown in acidified M9 medium, and translation was stopped by addition of 200 µg/ml of chloramphenicol. Samples were collected at the indicated time points upon treatment. For *ptsN*-*FLAG* wild-type and isogenic mutants with *phoP*, *clpX*, or *lon* gene deletions, bacteria were grown in acidified M9 medium. For *ptsN*-*FLAG* wild-type and *lon* mutant strains, bacteria were grown in acidified M9 medium, and translation was stopped by the addition of 200 µg/ml of chloramphenicol (Cm). Samples were collected at the indicated time points upon treatment. *t*_1/2_, half-life of EIIA^Ntr^. Representative results from at least three independent experiments are shown.

Given that an acidic pH and low Mg^2+^ are signals activating the sensor PhoQ (12–14), we hypothesized that the PhoP/PhoQ system might be involved in altering EIIA^Ntr^ amounts. To test this hypothesis, we examined the transcription and translation levels of EIIA^Ntr^ in isogenic wild-type and the *phoP* mutant *Salmonella* strains. Consistent with the expression of the p*_ptsN_-lacZ* fusion gene from a plasmid (Fig. 1H to J), the chromosomal *ptsN-lacZ* fusion also showed similar β-galactosidase activities under acidic and neutral pH conditions (see Fig. S1A in the supplemental material). Moreover, mutation of the *phoP* gene did not alter *ptsN* expression (Fig. S1A). In contrast, EIIA^Ntr^ amounts were significantly higher in the *phoP* null mutant than in the wild type when grown under acidic conditions (Fig. 2A), indicating that PhoP reduces EIIA^Ntr^ amounts independent of its transcription. The increased EIIA^Ntr^ abundance in the *phoP* mutant was restored by a plasmid expressing PhoP from a heterologous promoter (Fig. 2A). The absence of the cognate sensor kinase PhoQ also coordinated with a higher abundance of EIIA^Ntr^ (Fig. S1B) as in the *phoP* mutant (Fig. 2A), indicating that PhoP’s action in altering EIIA^Ntr^ abundance is dependent on PhoP’s phosphorylation. Furthermore, the lack of PhoP increased EIIA^Ntr^ abundance (Fig. 2B) even when transcription of *ptsN* was induced by isopropyl-β-d-thiogalactopyranoside (IPTG), further supporting the notion that PhoP modulates the abundance of EIIA^Ntr^ posttranscriptionally.

10.1128/mBio.00291-19.1 FIG S1 The PhoP/PhoQ system reduces EIIA^Ntr^ abundance independent of its transcription. (A) β-Galactosidase activities were determined from wild-type and *phoP Salmonella* with p*_ptsN_*-*lacZ* fusion on its normal chromosomal location. Bacteria were grown M9 medium at acidic or neutral pH. The means and SDs from three independent experiments are shown. (B) Western blot analysis of crude extracts prepared from *Salmonella ptsN*-*FLAG* wild type and an isogenic *phoQ* mutant or the *phoQ* mutant with a plasmid expressing PhoQ from a heterologous promoter. Strains were grown in acidified M9 medium with or without IPTG (0, 10, and 100 µM, from left to right). Representative results from at least three independent experiments are shown. Download FIG S1, PDF file, 0.2 MB. Copyright © 2019 Choi et al. 2019 Choi et al. This content is distributed under the terms of the Creative Commons Attribution 4.0 International license.

### EIIA^Ntr^ protein is degraded under acidic conditions in a PhoP-dependent manner.

We next investigated how PhoP controls EIIA^Ntr^ levels posttranscriptionally. PhoP may decrease EIIA^Ntr^ levels by reducing the stabilities of *ptsN* mRNA and/or EIIA^Ntr^ protein. *ptsN* mRNA showed similar levels of decay in both the wild type and the isogenic *phoP* mutant upon addition of rifampin to stop transcription (Fig. 2C). In contrast, the amount of EIIA^Ntr^ decreased in the wild type after inhibition of protein synthesis with chloramphenicol treatment (half-life [*t*_1/2_] < 60 min), whereas it remained constant in the strain lacking PhoP (*t*_1/2_ > 120 min) (Fig. 2D). Furthermore, this EIIA^Ntr^ degradation was detected when *Salmonella* was grown at an acidic pH but not at a neutral pH (Fig. S2). These results suggest that PhoP boosts the degradation of EIIA^Ntr^ protein in an acidic environment.

10.1128/mBio.00291-19.2 FIG S2 EIIA^Ntr^ is degraded at acidic pH. Western blot analysis of crude extracts prepared from *Salmonella* expressing *ptsN*-*FLAG* grown in M9 medium at pH 7.0 or 5.8. Translation was stopped by adding 200 µg/ml of chloramphenicol. Samples were collected at the indicated time points after treatment. *t*_1/2_, half-life of EIIA^Ntr^. Representative results from at least three independent experiments are shown. Download FIG S2, PDF file, 0.2 MB. Copyright © 2019 Choi et al. 2019 Choi et al. This content is distributed under the terms of the Creative Commons Attribution 4.0 International license.

### Lon protease mediates PhoP-dependent degradation of EIIA^Ntr^.

Cytoplasmic proteases, including ClpXP and Lon, are involved in the proteolysis of cytosolic proteins in Gram-negative bacteria (29), and EIIA^Ntr^ is a cytoplasmic protein. As PhoP counteracts ClpXP-mediated proteolysis of RpoS via IraP (30), we first investigated the potential role of ClpXP in controlling EIIA^Ntr^ abundance. A *Salmonella* strain lacking ClpXP produced amounts of EIIA^Ntr^ comparable to those of the wild type, unlike the *phoP* mutant (Fig. 2E). However, a lack of Lon increased the abundance of EIIA^Ntr^ protein compared with that of the wild type, similar to the case with the *phoP* mutant strain (Fig. 2E). If Lon is responsible for the degradation of EIIA^Ntr^, the *lon* mutant should make EIIA^Ntr^ stable. Like the *phoP* mutant (Fig. 2D), the *lon* mutant displayed sustained abundance of EIIA^Ntr^ after chloramphenicol treatment (*t*_1/2_ > 120 min), whereas EIIA^Ntr^ levels dwindled in the wild type (*t*_1/2_ < 60 min) (Fig. 2F). Furthermore, double deletion of the *phoP* and *lon* genes resulted in amounts of EIIA^Ntr^ comparable to those in the *phoP* or *lon* single-deletion mutants (Fig. S3). These results suggest that PhoP favors Lon protease-mediated degradation of EIIA^Ntr^.

10.1128/mBio.00291-19.3 FIG S3 PhoP decreases EIIA^Ntr^ amounts via Lon-dependent degradation. Western blot analysis of crude extracts prepared from *Salmonella ptsN-FLAG* wild-type and isogenic mutants with *phoP*, *lon*, or *phoP lon* gene deletions. Strains were grown in acidified M9 medium. Representative results from at least three independent experiments are shown. Download FIG S3, PDF file, 0.2 MB. Copyright © 2019 Choi et al. 2019 Choi et al. This content is distributed under the terms of the Creative Commons Attribution 4.0 International license.

### EIIA^Ntr^ negatively controls expression of PhoP-regulated genes.

**FIG 3:**
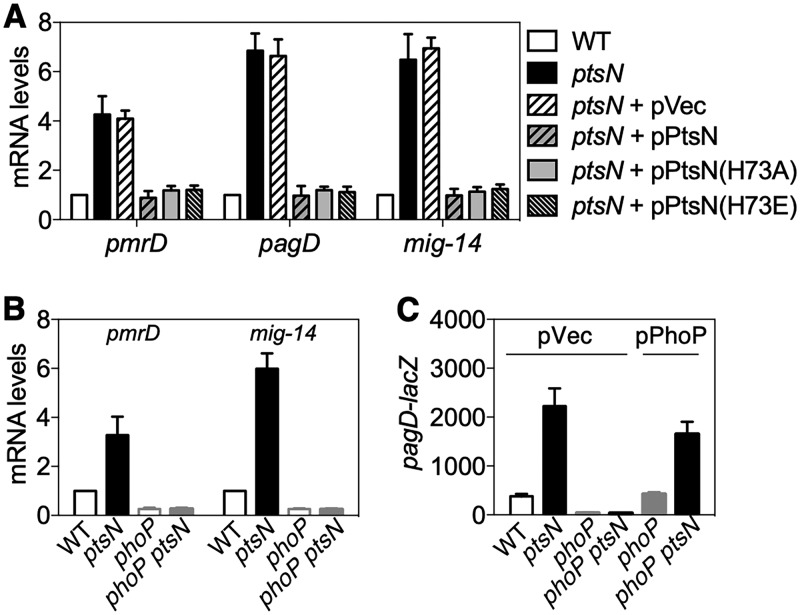
EIIA^Ntr^ reduces PhoP-regulated gene expression in a PhoP-dependent fashion. (A and B) mRNA levels of PhoP-activated *pmrD*, *pagD*, *mig-14*, and *mgtA* were determined in *Salmonella* wild-type, *ptsN* mutant, and *ptsN* mutant strains harboring a plasmid expressing EIIA^Ntr^, EIIA^Ntr^ (H73A), or EIIA^Ntr^ (H73E) [pPtsN, pPtsN(H73A), or pPtsN(H73E) or an empty vector (pVec) (A) and *Salmonella* wild-type and isogenic mutants with deletions of the *ptsN*, *phoP*, or *ptsN* and *phoP* genes (B). Bacteria were grown in M9 (pH 5.8). (C) β-Galactosidase activities of *Salmonella* with a p*_pagD_*-*lacZ* fusion in the normal chromosomal location and isogenic mutants with deletions of the *ptsN*, *phoP*, or *ptsN* and *phoP* genes with the indicated plasmids (empty vector [pVec] or plasmid expressing PhoP [pPhoP]) were determined. Bacteria were grown in acidified M9 medium with 100 µM IPTG. The means and SDs from three independent experiments are shown.

We next wondered why *Salmonella* curtails EIIA^Ntr^ amounts when it encounters an acidic pH, a PhoP-inducing condition inside macrophage phagosomes (31). To understand the role of EIIA^Ntr^, we investigated genes that are regulated by EIIA^Ntr^ using a DNA microarray experiments with wild-type and isogenic *ptsN* mutant strains grown in acidified minimal medium. We found 768 differentially expressed genes in the *ptsN* mutant compared with the wild type (>2-fold): 371 upregulated genes and 397 downregulated genes (Fig. S4). Consistent with a previous report (28), SPI-2 genes were more highly expressed in the *ptsN* mutant than in the wild type (Table S1). Interestingly, we found that transcript levels of PhoP-regulated genes were higher in the strain lacking EIIA^Ntr^ than in the wild type (Table S1). We further verified the EIIA^Ntr^’s regulatory effects on PhoP-activated genes using quantitative reverse transcription-PCR (qRT-PCR): the *ptsN* mutant displayed 3- to ∼7-fold-higher transcript levels of PhoP-regulated genes than the wild type (Fig. 3A). Moreover, the elevated expression of those genes in the *ptsN* mutant was restored to wild-type levels by a plasmid expressing the *ptsN* gene from a heterologous promoter but not by the plasmid vector (Fig. 3A). Interestingly, plasmid-driven heterologous expression of an unphosphorylatable variant of EIIA^Ntr^ (H73A) or a variant of EIIA^Ntr^ mimicking the phosphorylated form (H73E) (25) was also able to rescue the expression of PhoP-regulated genes similarly to wild-type EIIA^Ntr^ (Fig. 3A). These results suggest that EIIA^Ntr^ modulates the expression of PhoP target genes independent of EIIA^Ntr^’s phosphorylation status and PhoP transcription.

10.1128/mBio.00291-19.4 FIG S4 Differentially expressed genes by EIIA^Ntr^ at acidic pH. Fold changes (*ptsN*/wild-type) of genes from DNA microarray were plotted. Dashed lines indicate 0.05 for *P* value and 2 for fold change. Those sections above dashed lines indicate significantly regulated genes by EIIA^Ntr^, and “n” indicates number of genes in each section. Download FIG S4, PDF file, 0.2 MB. Copyright © 2019 Choi et al. 2019 Choi et al. This content is distributed under the terms of the Creative Commons Attribution 4.0 International license.

10.1128/mBio.00291-19.9 TABLE S1 Genes regulated by EIIA^Ntr^. Shown is a list of all genes from a microarray using wild-type and the *ptsN* mutant *Salmonella*. Genes that displayed 2-fold or more changes are marked with red (increase in the *ptsN* mutant) and green (decrease in the *ptsN* mutant). Download Table S1, PDF file, 0.8 MB. Copyright © 2019 Choi et al. 2019 Choi et al. This content is distributed under the terms of the Creative Commons Attribution 4.0 International license.

### Control of PhoP-regulated genes by EIIA^Ntr^ requires PhoP.

We next wondered whether EIIA^Ntr^ controls expression of PhoP to regulate PhoP regulon. If EIIA^Ntr^ directly controls PhoP-activated genes, EIIA^Ntr^ should be able to regulate those genes in the absence of PhoP. However, the absence of PhoP abrogated the regulatory effects of EIIA^Ntr^ on the expression of PhoP-activated genes (Fig. 3B), indicating that control of the PhoP regulon by EIIA^Ntr^ requires PhoP. If the regulation of PhoP-regulated genes by EIIA^Ntr^ is due to altered *phoP* transcription, heterologous expression of *phoP* from a plasmid should abolish the effect of EIIA^Ntr^ on the expression of those genes. A lack of EIIA^Ntr^ increased expression levels of PhoP-regulated genes, even when PhoP was produced from a heterologous promoter (Fig. 3C). These results indicate that EIIA^Ntr^ regulates PhoP regulon in a PhoP-dependent manner.

### EIIA^Ntr^ hampers PhoP binding to its target promoter DNA.

**FIG 4:**
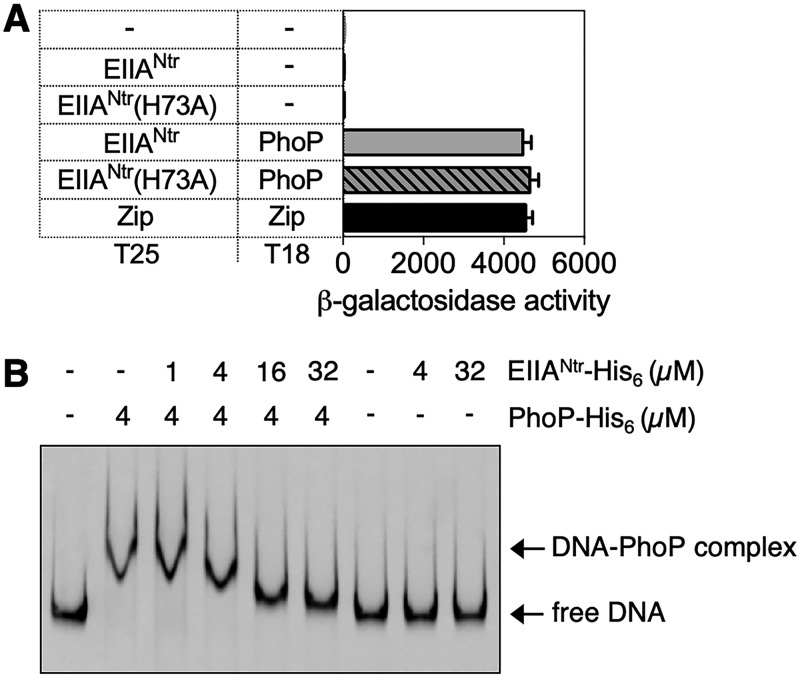
EIIA^Ntr^ inhibits PhoP binding to its target promoter by interacting with PhoP. (A) β-Galactosidase activities were determined from a *cya* mutant E. coli harboring the indicated plasmid combinations grown in LB containing 0.5 mM IPTG. The means and SDs from three independent experiments are shown. (B) *In vitro* binding of PhoP to the *pagD* promoter with or without EIIA^Ntr^. The *pagD* promoter DNA (80 fmol) was incubated with PhoP (4 µM) and EIIA^Ntr^ (1, 4, 16, and 32 µM) proteins. Representative results from at least three independent experiments are shown.

Given that EIIA^Ntr^ regulates other regulatory systems via protein-protein interaction (23, 24, 28, 32), we next investigated whether EIIA^Ntr^ interacts with the PhoP protein. We used the bacterial two-hybrid system, in which β-galactosidase levels are dependent on the proximity of fused proteins to fragments (i.e., T25 and T18) of the Bordetella pertussis adenylate cyclase in an Escherichia coli strain lacking its own adenylate cyclase (33). Coexpression of T25-EIIA^Ntr^ and T18-PhoP resulted in approximately 141-fold-higher levels of β-galactosidase activity than in strains expressing T25–EIIA^Ntr^ and T18 fragment or empty vectors (Fig. 4A), indicating that EIIA^Ntr^ interacts with PhoP. This activity was comparable to that from the positive-control strain harboring T25 and T18 fragments fused to the leucine zipper of the transcription factor GCN4 (Fig. 4A). Consistent with the observation that unphosphorylatable EIIA^Ntr^ (H73A) functions like the wild type in controlling the PhoP regulon (Fig. 3A), EIIA^Ntr^ (H73A) displayed an interaction with PhoP similar to that of the wild-type protein (Fig. 4A).

PhoP promotes transcription of the PhoP regulon by binding to DNA (34) when PhoP is activated by PhoQ-mediated phosphorylation under inducing conditions (35) or by reducing acetylation of PhoP (36). Thus, the interaction of EIIA^Ntr^ with PhoP could decrease PhoP activity by inhibiting the interaction of PhoP with the cognate kinase PhoQ (i.e., reducing phosphorylation), by promoting acetylation of PhoP, or by reducing deacetylation of PhoP. Alternatively, EIIA^Ntr^ could interfere with PhoP binding to DNA.

If EIIA^Ntr^ hampers PhoP phosphorylation by PhoQ, the lack of PhoQ should abolish the regulatory effects of EIIA^Ntr^ on the PhoP regulon. Because PhoP is not active in the absence of PhoQ, we investigated the function of EIIA^Ntr^ in a phoP* phoQ strain lacking PhoQ and expressing a PhoP variant that autophosphorylates from acetyl phosphate (35). EIIA^Ntr^ reduced *pagD* expression even in the absence of PhoQ (Fig. S5), indicating that the regulatory action of EIIA^Ntr^ is independent of PhoQ. We next examined whether acetylation of PhoP is responsible for EIIA^Ntr^-mediated regulation of PhoP target genes by mutating known acetylase (Pat) or deacetylase (CobB) (36). However, EIIA^Ntr^ displayed similar regulatory effects on *pagD* expression in the absence of Pat or CobB (Fig. S5).

10.1128/mBio.00291-19.5 FIG S5 EIIA^Ntr^ controls the PhoP-activated *pagD* gene expression independent of PhoQ and acetylation of PhoP. Fluorescence was determined from wild-type, *ptsN*, phoP* phoQ, phoP* phoQ ptsN, *cobB*, *cobB ptsN*, *pat*, and *pat ptsN Salmonella* strains harboring a plasmid with p*_pagD_*-*gfp* fusion. Bacteria were grown in acidified M9 medium. The means and SDs from three independent experiments are shown. Download FIG S5, PDF file, 0.2 MB. Copyright © 2019 Choi et al. 2019 Choi et al. This content is distributed under the terms of the Creative Commons Attribution 4.0 International license.

To test whether EIIA^Ntr^ inhibits PhoP’s DNA binding ability, a gel shift assay was conducted using purified PhoP and EIIA^Ntr^ proteins with the *phoP*-activated *pagD* promoter. Purified PhoP bound to the *pagD* promoter DNA and formed a complex with the probe DNA *in vitro* (Fig. 4B). EIIA^Ntr^ prevented PhoP from binding to the target DNA: the PhoP-DNA complex decreased to generate the unbound *pagD* promoter DNA when amounts of EIIA^Ntr^ increased (although an excess of EIIA^Ntr^ could not fully dissociate PhoP from DNA), and EIIA^Ntr^ alone did not form a complex with the DNA (Fig. 4B). However, addition of an EIIA^Ntr^ paralogue, EIIA^Glc^, did not alter binding of PhoP to DNA, indicating that it is specific to EIIA^Ntr^ (Fig. S6A). This inhibitory function of EIIA^Ntr^ is specific, as another regulatory protein, PmrA, bound to the *pbgP* promoter DNA regardless of EIIA^Ntr^ (Fig. S6B). Taken together, these data suggest that EIIA^Ntr^ reduces expression of the PhoP regulon by inhibiting PhoP binding to its target promoter DNA.

10.1128/mBio.00291-19.6 FIG S6 Regulatory effects of EIIA^Ntr^ on PhoP are specific. (A) *In vitro* binding of PhoP to the *pagD* promoter with or without EIIA^Glc^. The *pagD* promoter DNA (80 fmol) was incubated with PhoP (4 µM) and EIIA^Glc^ (1, 4, 16, and 32 µM). (B) *In vitro* binding of PmrA to the *pbgP* promoter with or without EIIA^Ntr^. The *pbgP* promoter DNA (80 fmol) was incubated with PmrA (4 µM) and EIIA^Ntr^ (1, 2, 4, 16, and 32 µM). Representative results from at least three independent experiments are shown. Download FIG S6, PDF file, 0.2 MB. Copyright © 2019 Choi et al. 2019 Choi et al. This content is distributed under the terms of the Creative Commons Attribution 4.0 International license.

### EIIA^Ntr^ delays activation of PhoP target genes inside macrophages.

**FIG 5:**
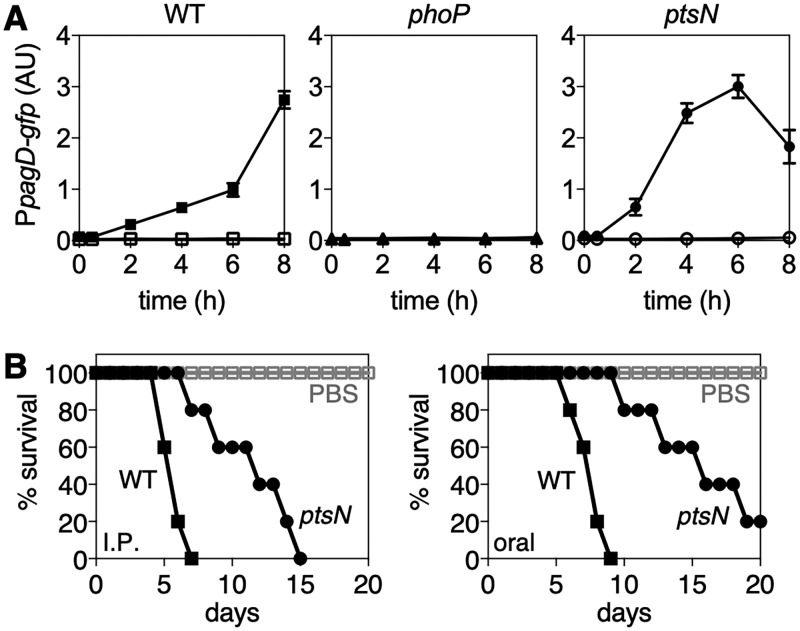
EIIA^Ntr^ delays activation of PhoP inside macrophages. (A) Macrophages were infected with the indicated *Salmonella* strains (wild type and *phoP* and *ptsN* mutants) harboring a plasmid containing a p*_pagD_-gfp* fusion (filled symbols) or a plasmid containing promoterless *gfp* (open symbols). Fluorescence was measured at the indicated time points after infection. The means and SDs from three independent experiments are shown. (B) Survival of BALB/c mice inoculated intraperitoneally with ∼10^2^ CFU (left) or orally with ∼10^6^ CFU (right) of wild-type and *ptsN* mutant *Salmonella*. Mice were monitored daily. Data are representative of those from two independent experiments.

We next examined if EIIA^Ntr^ could control the expression of PhoP-activated genes during infection. To evaluate this, macrophages were infected with wild-type and mutant *Salmonella* strains harboring a *gfp* fusion with the promoter of the PhoP-activated gene *pagD*, and fluorescence was measured. Activation of *pagD* expression inside macrophages was completely dependent on PhoP, because the *phoP* mutant was unable to produce any fluorescence, in contrast to the wild type (Fig. 5A). The *ptsN* mutant showed higher and earlier activation of *pagD* expression than the wild type (Fig. 5A), indicating that EIIA^Ntr^ inhibits PhoP activation inside macrophages. These results suggest that *Salmonella* delays expression of PhoP-activated genes inside macrophages via EIIA^Ntr^. The *ptsN* mutant also displayed accelerated activation of the *pagD* gene compared to that of the wild type in acidic pH (Fig. S7A). Consistent with a previous report (28), the lack of EIIA^Ntr^ rendered *Salmonella* virulence attenuated in mice inoculated via the intraperitoneal route (Fig. 5B, left). Defective virulence of the *ptsN* mutant *Salmonella* was also observed when mice were inoculated via the oral route (Fig. 5B, right). These results are in agreement that EIIA^Ntr^ controls the expression of various virulence genes, including the PhoP and SsrB regulons (Fig. 3) (28). And it is possible that the delayed virulence gene expression by EIIA^Ntr^ might be critical for *Salmonella* pathogenicity.

10.1128/mBio.00291-19.7 FIG S7 EIIA^Ntr^ inhibits expression of the *pagD* gene under PhoQ-inducing conditions. (A) *Salmonella* strains (wild type and the *ptsN* mutant) harboring a plasmid containing a p*_pagD_-gfp* fusion were grown in acidified M9 medium. Fluorescence was measured at the indicated time points after inoculation. (B and C) Fluorescence was measured from *Salmonella* strains (wild type and the *ptsN* mutant) harboring a plasmid containing a p*_pagD_-gfp* fusion. Strins were grown in M9 medium with high or low Mg^2+^ (2 mM or 10 µM) (A) or M9 medium with or without of 5 µg/ml of antimicrobial peptide C18G (B). The means and SDs from three independent experiments are shown. Download FIG S7, PDF file, 0.2 MB. Copyright © 2019 Choi et al. 2019 Choi et al. This content is distributed under the terms of the Creative Commons Attribution 4.0 International license.

## DISCUSSION

**FIG 6:**
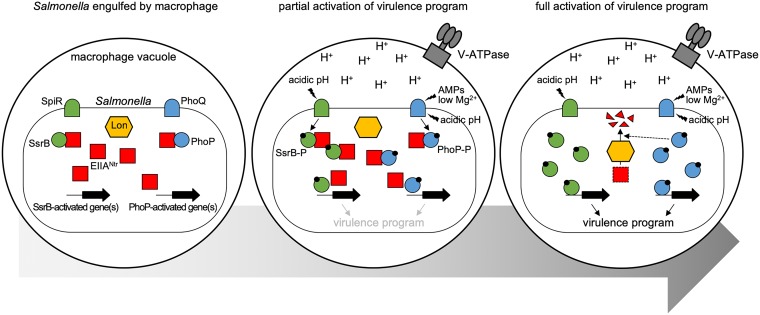
EIIA^Ntr^ delays activation of the virulence program by inhibiting virulence regulators. (Left) When *Salmonella* is only engulfed by macrophages, expression of virulence genes such as SPI-2 and PhoP-activated genes is “off” because there is no activating signal(s) and EIIA^Ntr^ inhibits them. (Middle) Upon phagosomal acidification via recruitment of host V-ATPase, the acidic pH activates pH sensors, such as PhoQ and SpiR, thereby activating SsrB and PhoP regulatory proteins. Several antimicrobial peptides (AMPs) also activates the sensor PhoQ. However, expression of virulence genes is only partially activated because of the inhibitory effects of EIIA^Ntr^. (Right) When PhoP activation reaches a certain level, EIIA^Ntr^ is degraded by Lon protease, thereby alleviating the inhibitory function of EIIA^Ntr^. This, in turn, allows further activation of both PhoP- and SsrB-activated virulence genes, thereby operating the virulence program.

In this study, we established that *Salmonella* employs EIIA^Ntr^ to delay the activation of its virulence program inside acidic phagosomes (Fig. 6). The master virulence regulator PhoP promotes the Lon-mediated degradation of EIIA^Ntr^ under acidic conditions (Fig. 1 and 2); this, in turn, favors the expression of PhoP-activated genes under acidic conditions (Fig. 3) and inside macrophages (Fig. 5A). Thus, the reduction of EIIA^Ntr^ amounts in the acidic phagosome allows delayed but robust activation of the *Salmonella* virulence program, including the PhoP/PhoQ system as well as SPI-2 genes (28) (Fig. 6), thereby enhancing its fitness inside the host (11, 28) (Fig. 5B).

Although nitrogen availability controls the phosphorylation status of EIIA^Ntr^ (37), total amounts of EIIA^Ntr^ remain unaltered *in vivo* (37) (Fig. 1D), suggesting that EIIA^Ntr^ phosphorylation status is important for its regulatory function. However, the role of EIIA^Ntr^ phosphorylation in its regulatory function is controversial: some EIIA^Ntr^ activities are dependent on its phosphorylation status (23, 24, 26, 27), whereas others are not (24, 25, 27, 28, 32, 38). EIIA^Ntr^ interacts with PhoP and decreases expression of PhoP-activated genes regardless of its phosphorylation status (Fig. 3A and Fig. 4A). Moreover, EIIA^Ntr^ accumulated in the *phoP* mutant of even in the absence of EI^Ntr^ (encoded by the *ptsP* gene), the phosphor donor for EIIA^Ntr^ (Fig. S8). Together with previous reports (24, 25, 27, 28, 32, 38), these findings indicate that EIIA^Ntr^ operates some functions regardless of its phosphorylation status, suggesting that it is important to understand how bacteria control cellular amounts of EIIA^Ntr^ protein.

10.1128/mBio.00291-19.8 FIG S8 PhoP reduces EIIA^Ntr^ amounts independent of EIIA^Ntr^'s phosphorylation status. Shown is Western blot analysis of crude extracts prepared from the *Salmonella ptsN*-*FLAG* wild type and isogenic mutants with *phoP*, *ptsP*, or *phoP ptsP* gene deletions. Strains were grown in acidified M9 medium. Representative results from at least three independent experiments are shown. Download FIG S8, PDF file, 0.2 MB. Copyright © 2019 Choi et al. 2019 Choi et al. This content is distributed under the terms of the Creative Commons Attribution 4.0 International license.

In this study, we established that *Salmonella* modulates EIIA^Ntr^ abundance via Lon-mediated degradation in a PhoP-dependent manner (Fig. 2). Moreover, we demonstrated that the *ptsN* gene has its own transcriptional start site, although the *ptsN* gene is considered a component of the *rpoN* operon (21). This raises the possibility that *Salmonella* may control expression of *ptsN* independent of *rpoN.* Indeed, a recent transcriptome sequencing (RNA-seq) study showed that nitrogen oxide shock reduces *ptsN* transcript levels but does not alter *rpoN* mRNA levels (39). Moreover, a recent study has shown that EIIA^Ntr^ is degraded by Lon in the absence of GlmS and *N*-acetylglucosamine, although this degradation was not observed in the wild type (27). Furthermore, EIIA^Ntr^ accumulates in the presence of acetylglucosamine in a degradation-independent manner (27). As a transcriptional regulator, PhoP probably induces an adapter-like protein that can alter degradability of EIIA^Ntr^ by Lon in a PhoP-dependent manner; rather, PhoP brings EIIA^Ntr^ to Lon. PhoP controls 9% of *Salmonella* genes (40) despite the fact that limited numbers of its direct targets are known. Given that EIIA^Ntr^ regulates 768 genes (Fig. S4), PhoP perhaps controls a subset of genes via EIIA^Ntr^.

Our findings now provide mechanisms for how EIIA^Ntr^ contributes to *Salmonella* virulence. EIIA^Ntr^ tunes the timing and extent of virulence regulatory systems’ activation inside host cells (Fig. 3A and Fig. 5A) (28), thereby enabling *Salmonella* to properly manage its virulence program. In addition, there are other biological processes regulated by EIIA^Ntr^, and they are potentially involved in bacterial virulence, which includes ppGpp accumulation, metabolism to produce amino sugars, and potassium uptake (23, 24, 26, 27). Thus, *Salmonella* probably changes various processes by altering EIIA^Ntr^ abundance during infection.

The function of EIIA^Ntr^ described here may explain the different behaviors of PhoP-regulated genes in *in vitro* cultures and inside macrophages; the expression of PhoP-activated genes reaches maximal levels 5 to ∼25 min after exposure to an environment that activates PhoQ (41), whereas it takes hours inside macrophages (16, 42) (Fig. 5A). Moreover, full activation of PhoP in acidic pH requires a PhoP-activated UgtL protein which amplifies the response of PhoQ to an acidic environment (43). Because EIIA^Ntr^ binds to and hampers SsrB (28), induction of SsrB would favor PhoP activation by reducing the number of EIIA^Ntr^ proteins interacting with PhoP. Moreover, inhibition of PhoP enables EIIA^Ntr^ to efficiently hamper the SsrB regulon given that PhoP transcriptionally activates SsrB (44) and that EIIA^Ntr^ hinders SsrB’s regulatory function (28).

Although the activation of the *pagD* gene inside macrophages was delayed in the wild type, maximal levels of activation were similar in the wild type and the *ptsN* mutant (Fig. 5A). In acidified defined media, however, the wild-type and the *ptsN* mutant strains did not show similar maximal levels of *pagD* expression (Fig. 3 and Fig. S7A). This might be due to difference in conditions that *Salmonella* experiences: acidic pH in defined media and complicated conditions inside acidic phagosomes. Moreover, PhoQ responds to multiple signals, including acidic pH, low Mg^2+^, antimicrobial peptides, and high osmolarity (45). EIIA^Ntr^ reduces *pagD* gene expression not only at acidic pH (Fig. 3 and Fig. S5) but also under conditions stimulating PhoQ, low Mg^2+^ (Fig. S7B), and antimicrobial peptide C18G (Fig. S7C). Thus, we want to note that other PhoQ-inducing signals and/or other components inside phagosomes potentially contributing to the activation of the PhoP/PhoQ system inside phagosomes probably play a role in modulating EIIA^Ntr^-mediated function during infection.

Pathogens possess virulence genes that enable them to cause disease in the host. The EIIA^Ntr^ gene can be defined as a virulence gene because it promotes *Salmonella* virulence in mice (28) (Fig. 5B). Paradoxically, EIIA^Ntr^ antagonizes the functions of other virulence regulatory systems, such as PhoP/PhoQ and SsrB/SpiR, although the deletion of them highly attenuates *Salmonella* pathogenicity (11, 46). This inhibition of virulence regulators by EIIA^Ntr^ delays the timing of their activation (Fig. 5A) and probably allows robust activation once the amount of active regulator(s) supersedes the inhibitory threshold created by EIIA^Ntr^ (Fig. 6).

Why does *Salmonella* limit activation of virulence regulatory systems via EIIA^Ntr^ during infection, although this may potentially decrease its pathogenicity? One possible explanation is that overactivation of those virulence systems might be harmful to *Salmonella* survival inside the host. Hyperconstitutive activation of the PhoP/PhoQ system actually attenuates *Salmonella* virulence in mice (20). Moreover, PhoP activates not only virulence factors but also antivirulence factors (47–49). Balancing those virulence and antivirulence factors is probably important to achieving optimal fitness inside the host. In addition, it is possible that retarding induction of the PhoP-activated virulence program may allow *Salmonella* to efficiently replicate and spread to other cells. Because PhoP-activated SPI-2 genes result in macrophage death (50, 51) and early activation of SPI-2 genes accelerates cell death (48), delayed activation of SPI-2 genes probably allows *Salmonella* sufficient time to replicate inside the host cell.

Furthermore, EIIA^Ntr^ may also help *Salmonella* efficiently turn off those systems when unnecessary (e.g., when *Salmonella* escapes from phagocytes). Efficient transition between the “on” and “off” states of virulence regulatory systems allows the bacterial virulence program to operate efficiently and saves energy by reducing unnecessary usage.

## MATERIALS AND METHODS

### Bacterial strains, plasmids, and growth conditions.

The Salmonella enterica serovar Typhimurium strains used in this study were derived from strain SL1344. The strains and plasmids used in this study are listed in Table S2A. Phage P22-mediated transduction was performed as described previously (52). All *Salmonella* strains were grown aerobically at 30 or 37°C in Luria-Bertani (LB) or M9 minimal medium at the desired pH and Mg^2+^ concentrations to mid- to late log phase unless specified. Antimicrobial peptide C18G was treated at 5 µg/ml for an hour. Antibiotics were used at the following concentrations: ampicillin, 50 μg/ml; chloramphenicol, 25 μg/ml; and kanamycin, 50 μg/ml. Primers used for the construction of bacterial strains and plasmids are listed in Table S2B.

10.1128/mBio.00291-19.10 TABLE S2 Bacterial strains, plasmids, and primers used in this study Download Table S2, PDF file, 0.1 MB. Copyright © 2019 Choi et al. 2019 Choi et al. This content is distributed under the terms of the Creative Commons Attribution 4.0 International license.

### Construction of mutant *Salmonella* strains.

To generate a *ptsN-FLAG* strain, a *cat* cassette was introduced in the 3′ end of the *ptsN* gene as follows: the *cat* fragment was amplified from pKD3 using primers ptsN-FLAG-F/ptsN-FLAG-R and then introduced into wild-type *Salmonella* (SL1344) harboring plasmid pKD46 as previously described (53). The *cat* cassette was removed with plasmid pCP20 (53).

To generate a *ptsP* mutant, a *cat* fragment was amplified from pKD3 using primers ptsP-Red-F/ptsP-Red-R and then introduced into wild-type *Salmonella* harboring plasmid pKD46 (53).

To generate a *phoP* mutant strain, a *kan* fragment was amplified from pKD13 using primers phoP-Red-F/phoP-Red-R and then introduced into wild-type *Salmonella* harboring plasmid pKD46 (53). Next, the *kan* cassette was removed with plasmid pCP20 (53).

To generate a *phoQ* mutant strain, a *kan* fragment was amplified from pKD13 using primers phoQ-Red-F/phoQ-Red-R and then introduced into wild-type *Salmonella* harboring plasmid pKD46 (53). Next, the *kan* cassette was removed with plasmid pCP20 (53).

To generate a *cobB* mutant strain, a *kan* fragment was amplified from pKD13 using primers cobB-P1-F-kan/cobB-P4-R-kan and then introduced into wild-type *Salmonella* harboring plasmid pKD46 (53).

To generate a *pat* mutant strain, a *kan* fragment was amplified from pKD13 using primers pat-P1-F-kan/pat-P4-R-kan and then introduced into wild-type *Salmonella* harboring plasmid pKD46 (53).

To generate a *clpXP* mutant, a *cat* fragment was amplified from pKD3 using primers clpP-Red-F/clpX-Red-R2 and then introduced into wild-type *Salmonella* harboring plasmid pKD46 (53). Next, the *kan* cassette was removed with plasmid pCP20 (53).

To generate a *lon* mutant, a *cat* fragment was amplified from pKD3 using primers lon-Red-F/lon-Red-R and then introduced into wild-type *Salmonella* harboring plasmid pKD46 (53). Next, the *kan* cassette was removed with plasmid pCP20 (53).

To generate a strain with the p*_ptsN_-lacZ* fusion in the normal chromosomal location, pCP20 was introduced into SR3203 (the *ptsN* mutant). Next, the *lacZ* fusion was generated with plasmid pCE70 (54).

To generate a strain with the p*_pagD_-lacZ* fusion in the normal chromosomal location, a *cat* fragment was amplified from pKD3 using primers pagD-1/pagD-2 and then introduced into wild-type *Salmonella* (SL1344) harboring plasmid pKD46 as previously described (53). The *cat* cassette was removed with plasmid pCP20 (53). Next, the *lacZ* fusion was generated with plasmid pCE70 (54).

### Construction of plasmids.

A plasmid expressing *ptsN-FLAG* was constructed as follows: the *ptsN-FLAG* fragment was amplified from *Salmonella* expressing *ptsN-FLAG* (SR4045) using primers ptsN-pF2/ptsN-pR2 and then introduced between the EcoRI and BamHI sites of pUHE21-2*lacI^q^* (55).

A plasmid expressing the *phoP* gene was constructed as follows: the *phoP* coding region was amplified from wild-type *Salmonella* (SL1344) using primers phoP-com-F2/phoP-com-R2 and then introduced between the EcoRI and BamHI sites of pUHE21-2*lacI^q^* (55).

A plasmid expressing *phoQ* gene was constructed as follows: the *phoQ* coding region was amplified from wild-type *Salmonella* (SL1344) using primers phoQ-com-F/phoQ-com-R and then introduced between the EcoRI and BamHI sites of pUHE21-2*lacI^q^* (55).

Plasmids expressing EIIA^Ntr^ variants [EIIA^Ntr^ (H73A) and EIIA^Ntr^ (H73E)] were constructed as follows: the pJJ14 plasmid was mutated using a QuikChange Lightning site-directed mutagenesis kit (Agilent Technologies) with primers ptsN_H73A-F/ptsN_H73A-R for H73A substitution and ptsN_H73A-F/ptsN_H73A-R for H73E substitution.

A plasmid expressing His_6_-tagged PhoP was constructed as follows: the *phoP* coding region was amplified from wild-type *Salmonella* (SL1344) using primers pPhoP-F/pPhoP-His6-R and then introduced between the BamHI and HindIII sites of pUHE21-2*lacI^q^* (55).

A plasmid expressing His_6_-tagged PmrA was constructed as follows: the *pmrA* coding region was amplified from wild-type *Salmonella* (SL1344) using primers pPmrA-F/pPmrA-His6-R and then introduced between the BamHI and HindIII sites of pUHE21-2*lacI^q^* (55).

Plasmids containing promoter fusions were constructed as follows: the *rpoN* and *ptsN* promoter regions were amplified from wild-type *Salmonella* (SL1344) using primers rpoN-pF1/rpoN-pR1 and ptsN-pF4/ptsN-pR4, respectively. Next, they were introduced between the EcoRI and BamHI sites of pRS415 (56). The *pagD* promoter region was amplified from wild-type *Salmonella* (SL1344) using primers PpagD-gfp-F/PpagD-gfp-R and then introduced between the EcoRI and BamHI sites of pFPV25 (57).

A plasmid expressing T18-PhoP fusion protein was constructed as follows: the *phoP* gene was amplified from wild-type *Salmonella* (SL1344) using primers phoP-F2/phoP-R1 and then introduced between the BamHI and EcoRI sites of pUT18C (58).

### Western blotting.

*Salmonella* strains expressing the EIIA^Ntr^-FLAG protein from its normal chromosomal location or under the control of a heterologous promoter were grown as described in “Bacterial strains, plasmids, and growth conditions,” above. Bacteria were collected by centrifugation, and cell lysates were prepared using B-PER solution (Pierce). Cell lysates were separated by 12% SDS-PAGE, and EIIA^Ntr^ and DnaK were detected using anti-FLAG (Sigma) and anti-DnaK (Abcam) antibodies, respectively. Blots were developed using anti-mouse IgG horseradish peroxidase-linked antibody with the ECL detection system (Amersham Biosciences).

### β-Galactosidase assay.

β-Galactosidase assays were carried out in triplicate, and the activity was determined as described previously (59).

### RNA isolation and quantitative RT (qRT)-PCR.

*Salmonella* strains were grown as described above, and total RNA was isolated using an RNeasy minikit (Qiagen). After DNase treatment of the isolated RNA, cDNA was synthesized using Omnitranscript reverse transcription reagents (Qiagen) and random hexamers (Invitrogen). Quantification of the cDNA was carried out using 2× iQ SYBR Green Supermix (Bio-Rad), and real-time amplification of the PCR products was performed using the iCycler iQ real-time detection system (Bio-Rad). The primers used for detection of the gene transcripts are listed in Table S2B . Data were normalized to the abundance of 16S rRNA expression levels.

### RNA or protein stability analyses.

To test RNA stability, bacterial cultures were treated with 0.1 mg/ml of rifampin to stop transcription, and samples were collected at the desired time points. Total RNA was isolated, and mRNA levels were determined by qRT-PCR as described above. To test the protein stability, bacterial cultures were treated with 0.2 mg/ml of chloramphenicol to stop protein synthesis, and samples were collected at the desired time points. Protein levels were analyzed using Western blot analysis.

### Bacterial two-hybrid assay.

E. coli BTH101 organisms harboring derivatives of plasmids pUT18 and pKT25 were grown overnight in LB broth containing ampicillin (100 µg/ml) and kanamycin (50 µg/ml), adding a 1:100 dilution to 1 ml of the same fresh medium containing 0.5 mM IPTG and employing shaking at 30°C overnight as previously described (33, 58).

### Purification of proteins.

His_6_-tagged PhoP, His_6_-tagged PmrA and His_6_-tagged EIIA^Ntr^ were expressed in E. coli BL21(DE3). Bacterial cells were grown in LB medium at 37°C until the optical density at 600 nm (OD_600_) reached 0.5, and the expression of those proteins was induced by addition of IPTG (0.5 M) followed by growth at 30°C for 5 h. Cells were harvested, washed, and suspended in buffer A (20 mM Tris [pH 8.0], 150 mM NaCl, and 20 mM imidazole). Then the cells were disrupted by sonication, and cell debris was removed by centrifugation at 20,000 × *g* at 4°C for 30 min. The supernatant was applied to a 1.5-ml nickel- nitrilotriacetic acid (Ni-NTA) agarose column equilibrated in buffer A, washed with a 25-column volume of the same buffer, and eluted using a gradient of buffer A and buffer B (20 mM Tris [pH 8.0], 150 mM NaCl, and 250 mM imidazole). The fractions were then collected and analyzed by SDS-PAGE, and selected fractions were dialyzed against buffer C (20 mM Tris [pH 8.0], 150 mM NaCl, and 10% glycerol).

### EMSA.

DNA fragments containing the promoter region of the *pagD* or *pbgP* gene were amplified by PCR using the primers EMSA-pagD-F/EMSA-pagD-R and EMSA-pbgP-F/EMSA-pbgP-R, respectively. Purified promoter DNA (80 fmol) was incubated with the desireded concentrations of purified PhoP-His_6_ or PmrA-His_6_ with EIIA^Ntr^-His_6_ or EIIA^Glc^-His_6_ at room temperature for 20 min in 15 μl of binding buffer (10 mM Tris [pH 7.5], 0.5 mM EDTA, 1 mM MgCl_2_, 0.5 mM dithiothreitol [DTT], and 50 mM NaCl) containing 5 ng/µl of poly(dI-dC). Samples were prepared by addition of 3 μl of 6× electrophoretic mobility shift assay (EMSA) gel loading solution and separated by electrophoresis using a 6% nondenaturing polyacrylamide gel. DNA staining was performed according to the manufacturer’s instructions (EMSA kit; E33075; Thermo Fisher Scientific).

### Macrophage infection assay.

The murine-derived macrophage line RAW264.7 was cultured in Dulbecco’s modified Eagle’s medium (DMEM; Life Technologies) supplemented with 10% heat-inactivated fetal bovine serum (FBS; Life Technologies) at 37°C with 5% CO_2_. Macrophages were seeded in 24-well tissue culture plates at 5 × 10^5^ per well 1 day before infection with *Salmonella*. Confluent monolayers were inoculated with bacterial cells that had been grown overnight in LB broth, washed with phosphate-buffered saline (PBS), and resuspended in 0.1 ml of prewarmed DMEM at a multiplicity of infection of 20. Following a 30-min incubation, the wells were washed three times with prewarmed PBS to remove extracellular bacteria and then incubated with prewarmed medium supplemented with 100 µg/ml of gentamicin for 1 h to kill extracellular bacteria. Next, the wells were washed three times with prewarmed PBS and incubated with prewarmed medium supplemented with 10 µg/ml of gentamicin. At the desired time points, the cells were washed three times with prewarmed PBS and subjected to the following procedures. For green fluorescent protein (GFP) assessment, washed cells were scraped with 200 µl of PBS and subjected to fluorescence measurements at 510 nm. For CFU measurements, washed cells were lysed with PBS containing 1% Triton X-100 and plated on LB agar plate at the proper dilutions.

### Mouse virulence assay.

Six-week-old female BALB/c mice were purchased from the Institute of Laboratory Animal Resources at Seoul National University. Five mice in each group were infected intraperitoneally or orally with 0.1 ml of PBS containing approximately 10^2^ or 10^6^ *Salmonella* cells grown in LB broth overnight, respectively. All animals were housed in temperature- and humidity-controlled rooms and maintained on a 12-h light/12-h dark cycle. All procedures complied with the regulations of the Institutional Animal Care and Use Committee of Seoul National University.

### Transcriptomic analysis.

RNA labeling, hybridization to the microarrays, scanning, and data analysis were performed at Macrogen. Triplicates of total RNAs from wild-type and *ptsN* mutant strains grown in acidified M9 medium (pH 5.8) were purified as described above and subjected to microarray using a CombiMatrix chip for the *Salmonella* Typhimurium SL1344 genome (12,396 probes covering 4,441 genes). Arrays were scanned using the Axon GenePix 4000B scanner (Molecular Devices LLC). Image analysis and feature extraction were performed using Axon GenePix Pro software (Molecular Devices). The data were analyzed using Avadis Prophetic software version 3.3 (Strand Genomics). Fold changes were calculated by comparing averaged normalized signal intensities in wild-type versus *ptsN* mutant *Salmonella*. The *t* test was performed in parallel with the use of a false-discovery rate correction for multiple testing (60). A *P* value of <0.05 was used to pinpoint significantly different expression levels of genes. A cutoff of a 2-fold change for up- or downregulated expression was chosen to define genes that were differentially expressed.

### Mapping of the transcription start site (primer extension assay).

Reverse transcription was conducted using ptsN-P1 and Superscript II (Invitrogen). The ladder was generated with a template DNA that was amplified using primers ptsN-PE-F/ptsN-PE-R and the genomic DNA of SL1344.

## References

[1] West AH, Stock AM 2001 Histidine kinases and response regulator proteins in two-component signaling systems. Trends Biochem Sci 26:369–376. doi:10.1016/S0968-0004(01)01852-7. 11406410

[2] Lukacs GL, Rotstein OD, Grinstein S 1991 Determinants of the phagosomal pH in macrophages. In situ assessment of vacuolar H(+)-ATPase activity, counterion conductance, and H^+^ “leak”. J Biol Chem 266:24540–24548. 1837024

[3] Jankowski A, Scott CC, Grinstein S 2002 Determinants of the phagosomal pH in neutrophils. J Biol Chem 277:6059–6066. doi:10.1074/jbc.M110059200. 11744729

[4] Weiss G, Schaible UE 2015 Macrophage defense mechanisms against intracellular bacteria. Immunol Rev 264:182–203. doi:10.1111/imr.12266. 25703560 PMC4368383

[5] Williamson LC, Neale EA 1994 Bafilomycin A1 inhibits the action of tetanus toxin in spinal cord neurons in cell culture. J Neurochem 63:2342–2345. 7964755 10.1046/j.1471-4159.1994.63062342.x

[6] Rathman M, Sjaastad MD, Falkow S 1996 Acidification of phagosomes containing *Salmonella* Typhimurium in murine macrophages. Infect Immun 64:2765–2773. 8698506 10.1128/iai.64.7.2765-2773.1996 PMC174137

[7] Porte F, Liautard JP, Kohler S 1999 Early acidification of phagosomes containing *Brucella suis* is essential for intracellular survival in murine macrophages. Infect Immun 67:4041–4047. 10417172 10.1128/iai.67.8.4041-4047.1999 PMC96697

[8] Chong A, Wehrly TD, Nair V, Fischer ER, Barker JR, Klose KE, Celli J 2008 The early phagosomal stage of *Francisella tularensis* determines optimal phagosomal escape and *Francisella* pathogenicity island protein expression. Infect Immun 76:5488–5499. doi:10.1128/IAI.00682-08. 18852245 PMC2583578

[9] Alpuche Aranda CM, Swanson JA, Loomis WP, Miller SI 1992 *Salmonella* Typhimurium activates virulence gene transcription within acidified macrophage phagosomes. Proc Natl Acad Sci U S A 89:10079–10083. doi:10.1073/pnas.89.21.10079. 1438196 PMC50281

[10] Fields PI, Groisman EA, Heffron F 1989 A *Salmonella* locus that controls resistance to microbicidal proteins from phagocytic cells. Science 243:1059–1062. doi:10.1126/science.2646710. 2646710

[11] Miller SI, Kukral AM, Mekalanos JJ 1989 A two-component regulatory system (*phoP phoQ*) controls *Salmonella* Typhimurium virulence. Proc Natl Acad Sci U S A 86:5054–5058. doi:10.1073/pnas.86.13.5054. 2544889 PMC297555

[12] Prost LR, Daley ME, Le Sage V, Bader MW, Le Moual H, Klevit RE, Miller SI 2007 Activation of the bacterial sensor kinase PhoQ by acidic pH. Mol Cell 26:165–174. doi:10.1016/j.molcel.2007.03.008. 17466620

[13] Choi J, Groisman EA 2016 Acidic pH sensing in the bacterial cytoplasm is required for *Salmonella* virulence. Mol Microbiol 101:1024–1038. doi:10.1111/mmi.13439. 27282333 PMC5015592

[14] Garcia Vescovi E, Soncini FC, Groisman EA 1996 Mg2+ as an extracellular signal: environmental regulation of *Salmonella* virulence. Cell 84:165–174. doi:10.1016/S0092-8674(00)81003-X. 8548821

[15] Bader MW, Sanowar S, Daley ME, Schneider AR, Cho U, Xu W, Klevit RE, Le Moual H, Miller SI 2005 Recognition of antimicrobial peptides by a bacterial sensor kinase. Cell 122:461–472. doi:10.1016/j.cell.2005.05.030. 16096064

[16] Martin-Orozco N, Touret N, Zaharik ML, Park E, Kopelman R, Miller S, Finlay BB, Gros P, Grinstein S 2006 Visualization of vacuolar acidification-induced transcription of genes of pathogens inside macrophages. Mol Biol Cell 17:498–510. doi:10.1091/mbc.e04-12-1096. 16251362 PMC1345685

[17] Núñez-Hernández C, Tierrez A, Ortega AD, Pucciarelli MG, Godoy M, Eisman B, Casadesús J, García-del Portillo F 2013 Genome expression analysis of nonproliferating intracellular *Salmonella enterica* serovar Typhimurium unravels an acid pH-dependent PhoP-PhoQ response essential for dormancy. Infect Immun 81:154–165. doi:10.1128/IAI.01080-12. 23090959 PMC3536153

[18] Puiac S, Negrea A, Richter-Dahlfors A, Plant L, Rhen M 2009 Omeprazole antagonizes virulence and inflammation in *Salmonella enterica*-infected RAW264.7 cells. Antimicrob Agents Chemother 53:2402–2409. doi:10.1128/AAC.01483-08. 19307359 PMC2687253

[19] Arpaia N, Godec J, Lau L, Sivick KE, McLaughlin LM, Jones MB, Dracheva T, Peterson SN, Monack DM, Barton GM 2011 TLR signaling is required for *Salmonella* Typhimurium virulence. Cell 144:675–688. doi:10.1016/j.cell.2011.01.031. 21376231 PMC3063366

[20] Miller SI, Mekalanos JJ 1990 Constitutive expression of the *phoP* regulon attenuates *Salmonella* virulence and survival within macrophages. J Bacteriol 172:2485–2490. doi:10.1128/jb.172.5.2485-2490.1990. 2185222 PMC208887

[21] Powell BS, Court DL, Inada T, Nakamura Y, Michotey V, Cui X, Reizer A, Saier MH Jr, Reizer J 1995 Novel proteins of the phosphotransferase system encoded within the *rpoN* operon of *Escherichia coli*. Enzyme IIA^Ntr^ affects growth on organic nitrogen and the conditional lethality of an *era*^ts^ mutant. J Biol Chem 270:4822–4839. doi:10.1074/jbc.270.9.4822. 7876255

[22] Reizer J, Reizer A, Merrick MJ, Plunkett G III, Rose DJ, Saier MH Jr. 1996 Novel phosphotransferase-encoding genes revealed by analysis of the *Escherichia coli* genome: a chimeric gene encoding an enzyme I homologue that possesses a putative sensory transduction domain. Gene 181:103–108. doi:10.1016/S0378-1119(96)00481-7. 8973315

[23] Lee CR, Cho SH, Yoon MJ, Peterkofsky A, Seok YJ 2007 *Escherichia coli* enzyme IIA^Ntr^ regulates the K^+^ transporter TrkA. Proc Natl Acad Sci U S A 104:4124–4129. doi:10.1073/pnas.0609897104. 17289841 PMC1794712

[24] Luttmann D, Heermann R, Zimmer B, Hillmann A, Rampp IS, Jung K, Gorke B 2009 Stimulation of the potassium sensor KdpD kinase activity by interaction with the phosphotransferase protein IIA(Ntr) in *Escherichia coli*. Mol Microbiol 72:978–994. doi:10.1111/j.1365-2958.2009.06704.x. 19400808

[25] Karstens K, Zschiedrich CP, Bowien B, Stulke J, Gorke B 2014 Phosphotransferase protein EIIA^Ntr^ interacts with SpoT, a key enzyme of the stringent response, in *Ralstonia eutropha* H16. Microbiology 160:711–722. doi:10.1099/mic.0.075226-0. 24515609

[26] Ronneau S, Petit K, De Bolle X, Hallez R 2016 Phosphotransferase-dependent accumulation of (p)ppGpp in response to glutamine deprivation in *Caulobacter crescentus*. Nat Commun 7:11423. doi:10.1038/ncomms11423. 27109061 PMC4848567

[27] Yoo W, Yoon H, Seok YJ, Lee CR, Lee HH, Ryu S 2016 Fine-tuning of amino sugar homeostasis by EIIA(Ntr) in *Salmonella* Typhimurium. Sci Rep 6:33055. doi:10.1038/srep33055. 27628932 PMC5024086

[28] Choi J, Shin D, Yoon H, Kim J, Lee CR, Kim M, Seok YJ, Ryu S 2010 *Salmonella* pathogenicity island 2 expression negatively controlled by EIIA(Ntr)-SsrB interaction is required for *Salmonella* virulence. Proc Natl Acad Sci U S A 107:20506–20511. doi:10.1073/pnas.1000759107. 21059960 PMC2996713

[29] Gur E, Biran D, Ron EZ 2011 Regulated proteolysis in Gram-negative bacteria—how and when? Nat Rev Microbiol 9:839–848. doi:10.1038/nrmicro2669. 22020261

[30] Tu X, Latifi T, Bougdour A, Gottesman S, Groisman EA 2006 The PhoP/PhoQ two-component system stabilizes the alternative sigma factor RpoS in *Salmonella enterica*. Proc Natl Acad Sci U S A 103:13503–13508. doi:10.1073/pnas.0606026103. 16938894 PMC1557385

[31] Flannagan RS, Cosio G, Grinstein S 2009 Antimicrobial mechanisms of phagocytes and bacterial evasion strategies. Nat Rev Microbiol 7:355–366. doi:10.1038/nrmicro2128. 19369951

[32] Luttmann D, Gopel Y, Gorke B 2012 The phosphotransferase protein EIIA(Ntr) modulates the phosphate starvation response through interaction with histidine kinase PhoR in *Escherichia coli*. Mol Microbiol 86:96–110. doi:10.1111/j.1365-2958.2012.08176.x. 22812494

[33] Karimova G, Pidoux J, Ullmann A, Ladant D 1998 A bacterial two-hybrid system based on a reconstituted signal transduction pathway. Proc Natl Acad Sci U S A 95:5752–5756. doi:10.1073/pnas.95.10.5752. 9576956 PMC20451

[34] Shin D, Groisman EA 2005 Signal-dependent binding of the response regulators PhoP and PmrA to their target promoters *in vivo*. J Biol Chem 280:4089–4094. doi:10.1074/jbc.M412741200. 15569664

[35] Chamnongpol S, Groisman EA 2000 Acetyl phosphate-dependent activation of a mutant PhoP response regulator that functions independently of its cognate sensor kinase. J Mol Biol 300:291–305. doi:10.1006/jmbi.2000.3848. 10873466

[36] Ren J, Sang Y, Tan Y, Tao J, Ni J, Liu S, Fan X, Zhao W, Lu J, Wu W, Yao YF 2016 Acetylation of lysine 201 Inhibits the DNA-binding ability of PhoP to regulate *Salmonella* virulence. PLoS Pathog 12:e1005458. doi:10.1371/journal.ppat.1005458. 26943369 PMC4778762

[37] Lee CR, Park YH, Kim M, Kim YR, Park S, Peterkofsky A, Seok YJ 2013 Reciprocal regulation of the autophosphorylation of enzyme I^Ntr^ by glutamine and alpha-ketoglutarate in *Escherichia coli*. Mol Microbiol 88:473–485. doi:10.1111/mmi.12196. 23517463 PMC3633653

[38] Sharma R, Shimada T, Mishra VK, Upreti S, Sardesai AA 2016 Growth inhibition by external potassium of Escherichia coli lacking PtsN (EIIA^Ntr^) is caused by potassium limitation mediated by YcgO. J Bacteriol 198:1868–1882. doi:10.1128/JB.01029-15. 27137496 PMC4907114

[39] Kroger C, Colgan A, Srikumar S, Handler K, Sivasankaran SK, Hammarlof DL, Canals R, Grissom JE, Conway T, Hokamp K, Hinton JC 2013 An infection-relevant transcriptomic compendium for *Salmonella enterica* serovar Typhimurium. Cell Host Microbe 14:683–695. doi:10.1016/j.chom.2013.11.010. 24331466

[40] Colgan AM, Kroger C, Diard M, Hardt WD, Puente JL, Sivasankaran SK, Hokamp K, Hinton JC 2016 The impact of 18 ancestral and horizontally-acquired regulatory proteins upon the transcriptome and sRNA landscape of *Salmonella enterica* serovar Typhimurium. PLoS Genet 12:e1006258. doi:10.1371/journal.pgen.1006258. 27564394 PMC5001712

[41] Zwir I, Yeo WS, Shin D, Latifi T, Huang H, Groisman EA 2014 Bacterial nucleoid-associated protein uncouples transcription levels from transcription timing. mBio 5:e01485-14. doi:10.1128/mBio.01485-14. 25293763 PMC4196223

[42] Westermann AJ, Forstner KU, Amman F, Barquist L, Chao Y, Schulte LN, Muller L, Reinhardt R, Stadler PF, Vogel J 2016 Dual RNA-seq unveils noncoding RNA functions in host-pathogen interactions. Nature 529:496–501. doi:10.1038/nature16547. 26789254

[43] Choi J, Groisman EA 2017 Activation of master virulence regulator PhoP in acidic pH requires the *Salmonella*-specific protein UgtL. Sci Signal 10:eaan6284. doi:10.1126/scisignal.aan6284. 28851823 PMC5966036

[44] Bijlsma JJ, Groisman EA 2005 The PhoP/PhoQ system controls the intramacrophage type three secretion system of *Salmonella enterica*. Mol Microbiol 57:85–96. doi:10.1111/j.1365-2958.2005.04668.x. 15948951

[45] Yuan J, Jin F, Glatter T, Sourjik V 2017 Osmosensing by the bacterial PhoQ/PhoP two-component system. Proc Natl Acad Sci U S A 114:E10792–E10798. doi:10.1073/pnas.1717272114. 29183977 PMC5740661

[46] Hensel M 2000 *Salmonella* pathogenicity island 2. Mol Microbiol 36:1015–1023. doi:10.1046/j.1365-2958.2000.01935.x. 10844687

[47] Mouslim C, Hilbert F, Huang H, Groisman EA 2002 Conflicting needs for a *Salmonella* hypervirulence gene in host and non-host environments. Mol Microbiol 45:1019–1027. doi:10.1046/j.1365-2958.2002.03070.x. 12180921

[48] Choi J, Groisman EA 2013 The lipopolysaccharide modification regulator PmrA limits *Salmonella* virulence by repressing the type three-secretion system Spi/Ssa. Proc Natl Acad Sci U S A 110:9499–9504. doi:10.1073/pnas.1303420110. 23690578 PMC3677452

[49] Pontes MH, Lee EJ, Choi J, Groisman EA 2015 *Salmonella* promotes virulence by repressing cellulose production. Proc Natl Acad Sci U S A 112:5183–5188. doi:10.1073/pnas.1500989112. 25848006 PMC4413311

[50] van der Velden AW, Lindgren SW, Worley MJ, Heffron F 2000 *Salmonella* pathogenicity island 1-independent induction of apoptosis in infected macrophages by *Salmonella enterica* serotype Typhimurium. Infect Immun 68:5702–5709. doi:10.1128/IAI.68.10.5702-5709.2000. 10992474 PMC101526

[51] Monack DM, Detweiler CS, Falkow S 2001 *Salmonella* pathogenicity island 2-dependent macrophage death is mediated in part by the host cysteine protease caspase-1. Cell Microbiol 3:825–837. doi:10.1046/j.1462-5822.2001.00162.x. 11736994

[52] Watanabe T, Ogata Y, Chan RK, Botstein D 1972 Specialized transduction of tetracycline resistance by phage P22 in *Salmonella* Typhimurium. I. Transduction of R factor 222 by phage P22. Virology 50:874–882. doi:10.1016/0042-6822(72)90441-2. 4565617

[53] Datsenko KA, Wanner BL 2000 One-step inactivation of chromosomal genes in *Escherichia coli* K-12 using PCR products. Proc Natl Acad Sci U S A 97:6640–6645. doi:10.1073/pnas.120163297. 10829079 PMC18686

[54] Merighi M, Ellermeier CD, Slauch JM, Gunn JS 2005 Resolvase-*in vivo* expression technology analysis of the *Salmonella enterica* serovar Typhimurium PhoP and PmrA regulons in BALB/c mice. J Bacteriol 187:7407–7416. doi:10.1128/JB.187.21.7407-7416.2005. 16237024 PMC1272988

[55] Soncini FC, Vescovi EG, Groisman EA 1995 Transcriptional autoregulation of the *Salmonella* Typhimurium *phoPQ* operon. J Bacteriol 177:4364–4371. doi:10.1128/jb.177.15.4364-4371.1995. 7543474 PMC177185

[56] Simons RW, Houman F, Kleckner N 1987 Improved single and multicopy *lac*-based cloning vectors for protein and operon fusions. Gene 53:85–96. doi:10.1016/0378-1119(87)90095-3. 3596251

[57] Valdivia RH, Falkow S 1996 Bacterial genetics by flow cytometry: rapid isolation of *Salmonella* Typhimurium acid-inducible promoters by differential fluorescence induction. Mol Microbiol 22:367–378. doi:10.1046/j.1365-2958.1996.00120.x. 8930920

[58] Karimova G, Ullmann A, Ladant D 2001 Protein-protein interaction between *Bacillus stearothermophilus* tyrosyl-tRNA synthetase subdomains revealed by a bacterial two-hybrid system. J Mol Microbiol Biotechnol 3:73–82. 11200232

[59] Miller JH 1972 Experiments in molecular genetics. Cold Spring Harbor Laboratory, Cold Spring Harbor, NY.

[60] Benjamini Y, Hochberg Y 1995 Controlling the false discovery rate—a practical and powerful approach to multiple testing. J R Stat Soc Series B Stat Methodol 57:289–300. doi:10.1111/j.2517-6161.1995.tb02031.x.

