# Supplementary material for: Programmed Delay of a Virulence Circuit Promotes Salmonella Pathogenicity
Source: mBio. 2019 Apr 9;10(2):e00291-19. doi: 10.1128/mBio.00291-19 (PMC6456747; doi:10.1128/mBio.00291-19)
Supplement: FIG S1 [file mBio.00291-19-sf001.pdf]

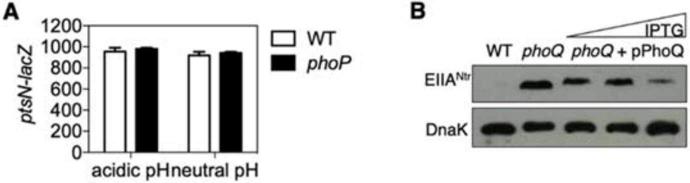

Fig. S1. The PhoP/PhoQ system reduces EIIA^{Ntr} abundance independent of its transcription. (**A**) β-galactosidase activities were determined from wild-type and *phoP Salmonella* with p_{ptsN}-*lacZ* fusion on its normal chromosomal location. Bacteria were grown M9 medium at acidic or neutral pH. The mean and SD from three independent experiments are shown. (**B**) Western blot analysis of crude extracts prepared from *Salmonella ptsN*-FLAG wild-type and isogenic *phoQ* mutant, or the *phoQ* mutant with a plasmid expressing PhoQ from heterologous promoter were grown in acidified M9 medium with or without IPTG (0, 10, and 100 μM from left to right). A representative of at least three independent experiments is shown.
