# Supplementary material for: Programmed Delay of a Virulence Circuit Promotes Salmonella Pathogenicity
Source: mBio. 2019 Apr 9;10(2):e00291-19. doi: 10.1128/mBio.00291-19 (PMC6456747; doi:10.1128/mBio.00291-19)
Supplement: FIG S2 [file mBio.00291-19-sf002.pdf]

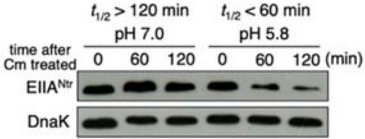

Fig. S2. EIIA^{Ntr} is degraded at acidic pH conditions. Western blot analysis of crude extracts prepared from *Salmonella* expressing *ptsN*-FLAG grown in M9 medium at pH 7.0 or 5.8 and its translation was stopped by adding 200 μg/ml chloramphenicol. Samples were collected at the indicated time points after treatment. $t_{1/2}$, half-life of EIIA^{Ntr}. A representative of at least three independent experiments is shown.
