# Supplementary material for: Programmed Delay of a Virulence Circuit Promotes Salmonella Pathogenicity
Source: mBio. 2019 Apr 9;10(2):e00291-19. doi: 10.1128/mBio.00291-19 (PMC6456747; doi:10.1128/mBio.00291-19)
Supplement: FIG S3 [file mBio.00291-19-sf003.pdf]

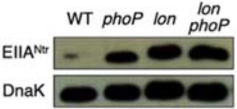

Fig. S3. PhoP decreases EIIA^{Ntr} amounts via Lon-dependent degradation. Western blot analysis of crude extracts prepared from *Salmonella ptsN-FLAG* wild-type and isogenic mutants with *phoP*, *lon*, or *phoP lon* gene deletions were grown in acidified M9 medium. A representative of at least three independent experiments is shown.
