# Supplementary material for: Programmed Delay of a Virulence Circuit Promotes Salmonella Pathogenicity
Source: mBio. 2019 Apr 9;10(2):e00291-19. doi: 10.1128/mBio.00291-19 (PMC6456747; doi:10.1128/mBio.00291-19)
Supplement: FIG S4 [file mBio.00291-19-sf004.pdf]

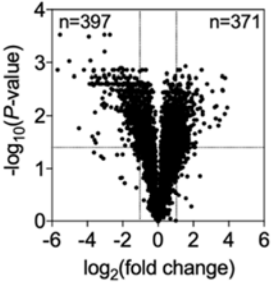

Fig. S4. Differentially expressed genes by EIIA^{Ntr} in acidic pH conditions. Fold-changes (*ptsN*/wild-type) of genes from DNA microarray were plotted. Dashed lines indicate 0.05 for *P*-value and 2 for fold change. Those sections above dashed lines indicate significantly regulated genes by EIIA^{Ntr} and "n" indicates number of genes in each section.
