# Supplementary material for: Programmed Delay of a Virulence Circuit Promotes Salmonella Pathogenicity
Source: mBio. 2019 Apr 9;10(2):e00291-19. doi: 10.1128/mBio.00291-19 (PMC6456747; doi:10.1128/mBio.00291-19)
Supplement: TABLE S1 [file mBio.00291-19-st001.pdf]

**Table S1. Genes regulated by EIIA$^{Ntr}$.** List of all genes from a microarray using wild-type and the *ptsN* mutant *Salmonella*. Genes displayed 2-fold or more changes are marked with red (increase in the *ptsN* mutant) and green (decrease in the *ptsN* mutant) colors.

| Gene | *P*-value (ptsN/WT) | Fold (ptsN/WT) | Probe Comment |
|---|---|---|---|
| STM0001_25_59 | 0.46 | 1.74 | 1251519lthrLlthr operon leader peptidel+ |
| STM0002_1688_1722 | 0.00 | -8.34 | 1251520lthrAlbifunctional aspartokinase I/homeserine dehydrogenase Il+ |
| STM0003_329_363 | 0.84 | -1.05 | 1251521lthrBlhomoserine kinasel+ |
| STM0004_1040_1074 | 0.04 | -1.83 | 1251522lthrClthreonine synthasel+ |
| STM0005_257_291 | 0.07 | 1.51 | 1251523lyaaAlhypothetical proteinl- |
| STM0006_784_818 | 0.03 | 1.43 | 1251524lyaaJlputative alanine/glycine transport proteinl- |
| STM0007_113_147 | 0.04 | 1.38 | 1251525ltalBltransaldolase Bl+ |
| STM0008_17_51 | 0.91 | -1.01 | 1251526lmogAlmolybdenum cofactor biosynthesis proteinl+ |
| STM0009_197_231 | 0.01 | 1.92 | 1251527lyaaHlhypothetical proteinl- |
| STM0010_641_675 | 0.36 | 1.07 | 1251528lhtgAlhypothetical proteinl- |
| STM0012_982_1016 | 0.06 | 1.40 | 1251530ldnaKlmolecular chaperone DnaKl+ |
| STM0013_797_831 | 0.02 | 2.14 | 1251531ldnaJlchaperone protein DnaJl+ |
| STM0014_673_707 | 0.04 | 1.16 | 1251532lSTM0014lputative transcriptional regulatorl+ |
| STM0017_385_419 | 0.03 | 1.23 | 1251535lSTM0017lhypothetical proteinl- |
| STM0018_1933_1967 | 0.02 | 1.76 | 1251536lSTM0018lputative exochitinasel+ |
| STM0019_2846_2880 | 0.20 | 1.09 | 1251537lSTM0019lputative hydroxymethyltransferasel+ |
| STM0020_241_275 | 0.01 | 1.45 | 1251538lSTM0020lputative cytoplasmic proteinl+ |
| STM0021_433_467 | 0.02 | 1.73 | 1251539lbcfAlfimbrial subunitl+ |
| STM0022_193_227 | 0.04 | 3.01 | 1251540lbcfBlfimbrial chaparonel+ |
| STM0024_233_267 | 0.94 | 1.01 | 1251542lbcfDlfimbrial subunitl+ |
| STM0025_81_115 | 0.01 | 2.19 | 1251543lbcfElfimbrial subunitl+ |
| STM0027_353_387 | 0.19 | -1.15 | 1251545lbcfGlfimbrial chaparonel+ |
| STM0028.1n_241_275 | 0.02 | 1.90 | 2673738lSTM0028.1nlhypothetical proteinl+ |
| STM0029_334_371 | 0.06 | -1.61 | 1251547lSTM0029lputative transcriptional regulatorl- |
| STM0030_214_248 | 0.03 | 1.92 | 1251548lSTM0030lputative transcriptional regulatorl+ |
| STM0031_217_251 | 0.03 | 1.51 | 1251549lSTM0031lputative transcriptional regulatorl- |
| STM0033_829_863 | 0.03 | 2.46 | 1251551lSTM0033lputative 5'-nucleotidasel- |
| STM0035_895_929 | 0.03 | -1.64 | 1251553lSTM0035lputative arylsulfatasel+ |
| STM0036_224_258 | 0.56 | -1.07 | 1251554lSTM0036lputative arylsulfatase regulatorl+ |
| STM0037_815_849 | 0.03 | 1.24 | 1251555lSTM0037lputative cytoplasmic proteinl+ |
| STM0039_232_266 | 0.39 | 1.07 | 1251557lnhaAlpH-dependent sodium/proton antiporterl+ |
| STM0040_361_395 | 0.46 | 1.09 | 1251558lnhaRltranscriptional activator NhaRl+ |
| STM0041_1377_1411 | 0.29 | 1.12 | 1251559lSTM0041lputative glycosyl hydrolasel- |
| STM0042_767_801 | 0.98 | -1.00 | 1251560lSTM0042lputative sodium galactoside symporterl- |

| | | | |
|---|---|---|---|
| STM0043_49_83 | 0.01 | -2.11 | 1251561\|rpsT\|30S ribosomal protein S20\|- |
| STM0044_170_207 | 0.06 | -1.39 | 1251562\|yaaY\|putative cytoplasmic protein\|+ |
| STM0045_553_587 | 0.99 | 1.00 | 1251563\|ribF\|bifunctional riboflavin kinase/FMN adenylyltransferase\|+ |
| STM0046_2362_2396 | 0.02 | -1.53 | 1251564\|ileS\|isoleucyl-tRNA synthetase\|+ |
| STM0047_33_67 | 0.06 | 1.19 | 1251565\|lspA\|lipoprotein signal peptidase\|+ |
| STM0048_9_43 | 0.07 | 1.11 | 1251566\|slpA\|FKBP-type peptidyl-prolyl cis-trans isomerase\|+ |
| STM0049_517_551 | 0.32 | -1.09 | 1251567\|ispH\|4-hydroxy-3-methylbut-2-enyl diphosphate reductase\|+ |
| STM0050_1100_1134 | 0.06 | 1.48 | 1251568\|STM0050\|putative nitrite reductase\|+ |
| STM0051_385_419 | 0.40 | 1.20 | 1251569\|rihC\|ribonucleoside hydrolase RihC\|+ |
| STM0052_617_651 | 0.00 | -1.52 | 1251570\|STM0052\|putative transcriptional regulator\|- |
| STM0053_749_783 | 0.07 | 2.27 | 1251571\|STM0053\|putative transcriptional regulator\|- |
| STM0054_370_404 | 0.03 | 1.43 | 1251572\|STM0054\|putative oxalacetate decarboxylase subunit beta\|- |
| STM0055_1194_1228 | 0.13 | 1.25 | 1251573\|STM0055\|pyruvate carboxylase subunit B\|- |
| STM0056_101_135 | 0.76 | 1.06 | 1251574\|STM0056\|oxaloacetate decarboxylase subunit gamma\|- |
| STM0057_1302_1336 | 0.04 | 2.72 | 1251575\|STM0057\|putative citrate-sodium symporter\|- |
| STM0060_129_163 | 0.26 | 1.55 | 1251578\|citE2\|putative citrate lyase beta chain\|+ |
| STM0061_1482_1516 | 0.92 | 1.01 | 1251579\|citF2\|putative citrate lyase alpha chain/citrate-ACP transferase\|+ |
| STM0062_89_123 | 0.14 | 1.22 | 1251580\|citX2\|putative cytoplasmic protein\|+ |
| STM0063_65_99 | 0.99 | -1.00 | 1251581\|citG2\|triphosphoribosyl-dephospho-CoA synthase\|+ |
| STM0064_289_323 | 0.04 | -1.38 | 1251582\|dapB\|dihydrodipicolinate reductase\|+ |
| STM0067_2485_2519 | 0.14 | -1.61 | 1251585\|carB\|carbamoyl phosphate synthase large subunit\|+ |
| STM0068_237_271 | 0.06 | 1.42 | 1251586\|caiF\|DNA-binding transcriptional activator CaiF\|+ |
| STM0069_209_243 | 0.95 | 1.00 | 1251587\|caiE\|carnitine racemase stimulation factor\|- |
| STM0070_449_483 | 0.82 | 1.02 | 1251588\|caiD\|carnitinyl-CoA dehydratase\|- |
| STM0071_1195_1229 | 0.44 | 1.27 | 1251589\|caiC\|putative crotonobetaine/carnitine-CoA ligase\|- |
| STM0072_731_765 | 0.37 | 1.17 | 1251590\|caiB\|crotonobetainyl-CoA:carnitine CoA-transferase\|- |
| STM0073_1104_1138 | 0.03 | 1.68 | 1251591\|caiA\|crotonobetainyl-CoA dehydrogenase\|- |
| STM0074_663_697 | 0.69 | -1.06 | 1251592\|caiT\|L-carnitine/gamma-butyrobetaine antiporter\|- |
| STM0075_449_483 | 0.03 | 2.30 | 1251593\|fixA\|putative electron transfer flavoprotein FixA\|+ |
| STM0078_3_37 | 0.20 | 1.54 | 1251596\|fixX\|putative ferredoxin\|+ |
| STM0079_726_760 | 0.18 | 1.25 | 1251597\|yaaU\|putative transport protein\|+ |
| STM0080_33_67 | 0.02 | 1.36 | 1251598\|STM0080\|putative outer membrane lipoprotein\|+ |
| STM0081_289_323 | 0.02 | 3.68 | 1251599\|STM0081\|putative secreted protein\|+ |
| STM0082_217_251 | 0.01 | 3.65 | 1251600\|STM0082\|putative secreted protein\|- |
| STM0084_1787_1821 | 0.04 | 1.61 | 1251602\|STM0084\|putative sulfatase\|+ |

| | | | |
|---|---|---|---|
| STM0085_433_467 | 0.98 | 1.00 | 1251603\|yabF\|glutathione-regulated potassium-efflux system ancillary protein KefF\|+ |
| STM0086_1440_1474 | 0.01 | 1.37 | 1251604\|kefC\|glutathione-regulated potassium-efflux system protein KefC\|+ |
| STM0087_345_379 | 0.04 | 1.89 | 1251605\|folA\|dihydrofolate reductase\|+ |
| STM0088_33_67 | 0.03 | -1.52 | 1251606\|apaH\|diadenosine tetraphosphatase\|- |
| STM0089_129_163 | 0.17 | 1.18 | 1251607\|apaG\|ApaG\|- |
| STM0090_641_675 | 0.19 | -1.10 | 1251608\|ksgA\|dimethyladenosine transferase\|- |
| STM0091_641_675 | 0.00 | -1.38 | 1251609\|pdxA\|4-hydroxythreonine-4-phosphate dehydrogenase\|- |
| STM0092_352_386 | 0.29 | -1.25 | 1251610\|surA\|peptidyl-prolyl cis-trans isomerase SurA\|- |
| STM0093_1874_1908 | 0.01 | -1.99 | 1251611\|imp\|organic solvent tolerance protein\|- |
| STM0094_353_387 | 0.03 | 1.26 | 1251612\|djlA\|Dna-J like membrane chaperone protein\|+ |
| STM0095_65_99 | 0.28 | 1.11 | 1251613\|rluA\|23S rRNA/tRNA pseudouridine synthase A\|- |
| STM0096_2436_2470 | 0.03 | -1.25 | 1251614\|hepA\|ATP-dependent helicase HepA\|- |
| STM0097_1625_1659 | 0.01 | 1.65 | 1251615\|polB\|DNA polymerase II\|- |
| STM0098_465_499 | 0.04 | 1.64 | 1251616\|STM0098\|putative secreted protein\|- |
| STM0100_2_36 | 0.01 | 2.25 | 1251618\|STM0100\|putative cytoplasmic protein\|+ |
| STM0101_226_260 | 0.82 | -1.04 | 1251619\|araD\|L-ribulose-5-phosphate 4-epimerase\|- |
| STM0102_1464_1498 | 0.02 | -1.47 | 1251620\|araA\|L-arabinose isomerase\|- |
| STM0103_871_905 | 0.01 | -1.59 | 1251621\|araB\|ribulokinase\|- |
| STM0104_217_251 | 0.65 | -1.06 | 1251622\|araC\|DNA-binding transcriptional regulator AraC\|+ |
| STM0105_321_355 | 0.10 | -1.48 | 1251623\|yabI\|hypothetical protein\|+ |
| STM0106_345_379 | 0.04 | -1.55 | 1251624\|thiQ\|thiamin transporter ATP-binding subunit\|- |
| STM0107_1348_1382 | 0.18 | -1.26 | 1251625\|thiP\|thiamin transporter membrane protein\|- |
| STM0108_49_83 | 0.62 | 1.28 | 1251626\|tbpA\|thiamin transporter substrate binding subunit\|- |
| STM0109_868_902 | 0.60 | -1.04 | 1251627\|yabN\|putative periplasmic binding protein\|- |
| STM0110_49_83 | 0.12 | 1.41 | 1251628\|leuD\|isopropylmalate isomerase small subunit\|- |
| STM0111_438_472 | 0.98 | 1.00 | 1251629\|leuC\|isopropylmalate isomerase large subunit\|- |
| STM0112_605_639 | 0.20 | -1.35 | 1251630\|leuB\|3-isopropylmalate dehydrogenase\|- |
| STM0113_1469_1503 | 0.09 | 2.11 | 1251631\|leuA\|2-isopropylmalate synthase\|- |
| STM0114_11_45 | 0.62 | 1.37 | 1251632\|leuL\|leu operon leader peptide\|- |
| STM0115_641_675 | 0.66 | -1.08 | 1251633\|leuO\|leucine transcriptional activator\|+ |
| STM0116_855_889 | 0.79 | 1.04 | 1251634\|ilvI\|acetolactate synthase 3 catalytic subunit\|+ |
| STM0117_417_451 | 0.02 | -2.16 | 1251635\|ilvH\|acetolactate synthase 3 regulatory subunit\|+ |
| STM0118_214_248 | 0.04 | -1.27 | 1251636\|fruR\|DNA-binding transcriptional regulator FruR\|+ |
| STM0119_17_51 | 0.86 | -1.03 | 1251637\|yabB\|hypothetical protein\|+ |
| STM0120_273_307 | 0.01 | -1.94 | 1251638\|mraW\|S-adenosyl-methyltransferase MraW\|+ |

| | | | |
|---|---|---|---|
| STM0121_34_68 | 0.02 | -1.61 | 1251639\|ftsL\|cell division protein FtsL\|+ |
| STM0122_1280_1314 | 0.77 | 1.04 | 1251640\|ftsI\|division specific transpeptidase\|+ |
| STM0123_617_651 | 0.01 | -2.12 | 1251641\|murE\|UDP-N-acetylmuramoylalanyl-D-glutamate--2 6-diaminopimelate ligase\|+ |
| STM0124_456_490 | 0.02 | -1.83 | 1251642\|murF\|UDP-N-acetylmuramoyl-tripeptide--D-alanyl-D-alanine ligase\|+ |
| STM0125_308_342 | 0.00 | -1.51 | 1251643\|mraY\|phospho-N-acetylmuramoyl-pentapeptide-transferase\|+ |
| STM0126_606_640 | 0.04 | -1.69 | 1251644\|murD\|UDP-N-acetylmuramoyl-L-alanyl-D-glutamate synthetase\|+ |
| STM0127_694_728 | 0.03 | -1.28 | 1251645\|ftsW\|essential cell division gene\|+ |
| STM0128_165_199 | 0.09 | -1.18 | 1251646\|murG\|N-acetylglucosaminyl transferase\|+ |
| STM0129_589_623 | 0.01 | -2.34 | 1251647\|murC\|UDP-N-acetylmuramate--L-alanine ligase\|+ |
| STM0130_609_643 | 0.00 | -1.83 | 1251648\|ddl\|D-alanine--D-alanine ligase\|+ |
| STM0131_449_483 | 0.02 | -1.47 | 1251649\|ftsQ\|cell division protein FtsQ\|+ |
| STM0132_792_826 | 0.00 | -1.81 | 1251650\|ftsA\|cell division protein FtsA\|+ |
| STM0133_361_395 | 0.26 | -1.21 | 1251651\|ftsZ\|cell division protein FtsZ\|+ |
| STM0134_473_507 | 0.10 | -1.19 | 1251652\|lpxC\|UDP-3-O-[3-hydroxymyristoyl] N-acetylglucosamine deacetylase\|+ |
| STM0135_425_459 | 0.11 | 1.63 | 1251653\|yacA\|SecA regulator SecM\|+ |
| STM0136_2347_2381 | 0.01 | -1.80 | 1251654\|secA\|preprotein translocase subunit SecA\|+ |
| STM0137_351_389 | 0.05 | -1.74 | 1251655\|mutT\|nucleoside triphosphate pyrophosphohydrolase  marked preference for dGTP\|+ |
| STM0137.1N_7_41 | 0.97 | -1.00 | 2673762\|STM0137.1N\|-\|- |
| STM0138_89_123 | 0.01 | 1.46 | 1251656\|yacG\|zinc-binding protein\|- |
| STM0139_513_547 | 0.57 | -1.04 | 1251657\|yacF\|hypothetical protein\|- |
| STM0140_513_547 | 0.34 | -1.14 | 1251658\|coaE\|dephospho-CoA kinase\|- |
| STM0141_477_511 | 0.86 | 1.03 | 1251659\|guaC\|guanosine 5'-monophosphate oxidoreductase\|+ |
| STM0142_652_686 | 0.35 | 1.19 | 1251660\|hofC\|type IV pilin biogenesis protein\|- |
| STM0144_65_99 | 0.83 | 1.05 | 1251662\|ppdD\|putative major pilin subunit\|- |
| STM0145_49_83 | 0.58 | -1.03 | 1251663\|nadC\|quinolinate phosphoribosyltransferase\|- |
| STM0146_193_227 | 0.32 | 1.08 | 1251664\|ampD\|N-acetyl-anhydromuranmyl-L-alanine amidase\|+ |
| STM0147_545_579 | 0.01 | 1.37 | 1251665\|ampE\|regulatory protein AmpE\|+ |
| STM0148_737_771 | 0.83 | -1.02 | 1251666\|STM0148\|putative cytoplasmic protein\|- |
| STM0149_472_506 | 0.93 | 1.01 | 1251667\|STM0149\|Na+/galactoside symporter\|- |
| STM0150_503_537 | 0.01 | -2.94 | 1251668\|aroP\|aromatic amino acid transporter\|- |
| STM0151_161_195 | 0.00 | 5.54 | 1251669\|pdhR\|transcriptional regulator PdhR\|+ |
| STM0152_1793_1827 | 0.01 | 2.52 | 1251670\|aceE\|pyruvate dehydrogenase subunit E1\|+ |
| STM0153_1275_1309 | 0.03 | 1.99 | 1251671\|aceF\|dihydrolipoamide acetyltransferase\|+ |

| | | | |
|---|---|---|---|
| STM0154_1034_1068 | 0.03 | 1.59 | 1251672\|lpdA\|dihydrolipoamide dehydrogenase\|+ |
| STM0155_27_61 | 0.03 | 1.35 | 1251673\|STM0155\|putative outer membrane protein\|+ |
| STM0156_681_715 | 0.95 | 1.01 | 1251674\|STM0156\|putative periplasmic protein\|- |
| STM0157_1474_1508 | 0.12 | 1.57 | 1251675\|yacH\|putative outer membrane protein\|- |
| STM0158_2239_2273 | 0.05 | -1.47 | 1251676\|acnB\|bifunctional aconitate hydratase 2/2-methylisocitrate dehydratase\|+ |
| STM0159_321_355 | 0.11 | 1.25 | 1251677\|STM0159\|putative restriction endonuclease\|- |
| STM0160_117_151 | 0.04 | 1.27 | 1251678\|yacL\|hypothetical protein\|+ |
| STM0161_705_739 | 0.06 | 1.93 | 1251679\|kdgT\|2-keto-3-deoxygluconate permease\|+ |
| STM0163_545_579 | 0.01 | -1.45 | 1251681\|pdxA\|4-hydroxythreonine-4-phosphate dehydrogenase 2\|+ |
| STM0164_97_131 | 0.10 | 1.19 | 1251682\|STM0164\|putative transcriptional regulator\|+ |
| STM0165_321_355 | 0.12 | -1.59 | 1251683\|speD\|S-adenosylmethionine decarboxylase\|- |
| STM0166_337_371 | 0.43 | 1.43 | 1251684\|speE\|spermidine synthase\|- |
| STM0168_1268_1302 | 0.04 | -1.96 | 1251686\|cueO\|multicopper oxidase\|+ |
| STM0169_1680_1714 | 0.30 | 1.27 | 1251687\|gcd\|glucose dehydrogenase\|- |
| STM0170_65_99 | 0.91 | -1.02 | 1251688\|hpt\|hypoxanthine-guanine phosphoribosyltransferase\|+ |
| STM0171_225_259 | 0.43 | -1.11 | 1251689\|yadF\|carbonic anhydrase\|- |
| STM0172_289_323 | 0.03 | 1.22 | 1251690\|yadG\|putative ABC-type multidrug transport system ATPase component\|+ |
| STM0173_609_643 | 0.08 | -1.13 | 1251691\|yadH\|putative transport protein\|+ |
| STM0174_801_835 | 0.11 | 1.48 | 1251692\|stiH\|putative fimbrial protein precurosr\|- |
| STM0175_1948_1982 | 0.65 | 1.09 | 1251693\|stiC\|putativie fimbrial usher\|- |
| STM0176_625_659 | 0.04 | 1.47 | 1251694\|stiB\|putative fimbrial chaparone\|- |
| STM0177_213_247 | 0.06 | 1.51 | 1251695\|stiA\|putative fimbrial subunit\|- |
| STM0178_294_328 | 0.07 | 1.38 | 1251696\|yadI\|putative PTS enzyme\|+ |
| STM0179_279_313 | 0.01 | -2.01 | 1251697\|yadE\|putative xylanase/chitin deacetylase\|+ |
| STM0180_145_179 | 0.77 | -1.07 | 1251698\|panD\|aspartate alpha-decarboxylase\|- |
| STM0181_321_355 | 0.99 | 1.01 | 1251699\|panC\|pantoate--beta-alanine ligase\|- |
| STM0182_229_263 | 0.11 | -1.21 | 1251700\|panB\|3-methyl-2-oxobutanoate hydroxymethyltransferase\|- |
| STM0183_377_411 | 0.29 | -1.18 | 1251701\|folK\|2-amino-4-hydroxy-6-hydroxymethyldihyropteridine pyrophosphokinase\|- |
| STM0184_524_558 | 0.03 | -2.08 | 1251702\|pcnB\|poly(A) polymerase I\|- |
| STM0185_513_547 | 0.25 | -1.28 | 1251703\|yadB\|glutamyl-Q tRNA(Asp) synthetase\|- |
| STM0186_17_51 | 0.42 | -1.13 | 1251704\|dksA\|DnaK transcriptional regulator DksA\|- |
| STM0187_251_285 | 0.09 | 1.16 | 1251705\|sfsA\|sugar fermentation stimulation protein A\|- |
| STM0188_211_245 | 0.00 | 1.52 | 1251706\|ligT\|2'-5' RNA ligase\|- |

| | | | |
|---|---|---|---|
| STM0189_1572_1606 | 0.01 | -1.88 | 1251707\|hrpB\|ATP-dependent RNA helicase HrpB\|+ |
| STM0190_1780_1814 | 0.92 | 1.00 | 1251708\|mrcB\|penicillin-binding protein 1b\|+ |
| STM0191_1959_1993 | 0.02 | -3.06 | 1251709\|fhuA\|ferrichrome outer membrane transporter\|+ |
| STM0192_729_763 | 0.22 | -1.41 | 1251710\|fhuC\|iron-hydroxamate transporter ATP-binding subunit\|+ |
| STM0193_249_283 | 0.01 | 2.61 | 1251711\|fhuD\|iron-hydroxamate transporter substrate-binding subunit\|+ |
| STM0194_2011_2045 | 0.05 | 2.35 | 1251712\|fhuB\|iron-hydroxamate transporter permease subunit\|+ |
| STM0195_65_99 | 0.91 | -1.05 | 1251713\|stfA\|putative fimbrial subunit\|+ |
| STM0198_305_339 | 0.02 | 2.62 | 1251716\|stfE\|putative minor fimbrial subunit\|+ |
| STM0199_117_151 | 0.50 | -1.28 | 1251717\|stfF\|putative minor fimbrial subunit\|+ |
| STM0200_335_369 | 0.18 | 1.17 | 1251718\|stfG\|putative minor fimbrial subunit\|+ |
| STM0201_705_739 | 0.04 | 2.15 | 1251719\|STM0201\|putative outer membrane protein\|+ |
| STM0202_698_732 | 0.04 | 1.56 | 1251720\|hemL\|glutamate-1-semialdehyde aminotransferase\|- |
| STM0203_943_977 | 0.01 | -1.65 | 1251721\|yadQ\|chloride channel protein\|+ |
| STM0204.S_273_307 | 0.01 | 1.90 | 1251722\|yadR\|hypothetical protein\|+ |
| STM0205_449_483 | 0.57 | 1.06 | 1251723\|yadS\|hypothetical protein\|- |
| STM0206_270_304 | 0.30 | 2.07 | 1251724\|btuF\|vitamin B12-transporter protein BtuF\|- |
| STM0207_289_323 | 0.06 | -1.43 | 1251725\|pfs\|5'-methylthioadenosine/S-adenosylhomocysteine nucleosidase\|- |
| STM0208_1351_1385 | 0.04 | 1.63 | 1251726\|dgt\|deoxyguanosinetriphosphate triphosphohydrolase\|+ |
| STM0209_1101_1135 | 0.04 | 2.06 | 1251727\|htrA\|serine endoprotease\|+ |
| STM0210_639_673 | 0.00 | -3.24 | 1251728\|cdaR\|carbohydrate diacid transcriptional activator CdaR\|+ |
| STM0211_289_323 | 0.01 | -2.60 | 1251729\|yaeH\|putative cytoplasmic protein\|- |
| STM0212_879_913 | 0.23 | 1.08 | 1251730\|STM0212\|putative inner membrane protein\|+ |
| STM0213_641_675 | 0.78 | 1.03 | 1251731\|dapD\|2 3 4 5-tetrahydropyridine-2-carboxylate N-succinyltransferase\|- |
| STM0214_2634_2668 | 0.14 | 1.22 | 1251732\|glnD\|PII uridylyl-transferase\|- |
| STM0215_593_627 | 0.04 | -1.34 | 1251733\|map\|methionine aminopeptidase\|- |
| STM0216_609_643 | 0.06 | -1.55 | 1251734\|rpsB\|30S ribosomal protein S2\|+ |
| STM0217_689_723 | 0.04 | -2.28 | 1251735\|tsf\|elongation factor Ts\|+ |
| STM0218_65_99 | 0.02 | -2.03 | 1251736\|pyrH\|uridylate kinase\|+ |
| STM0219_241_275 | 0.05 | -1.31 | 1251737\|frr\|ribosome recycling factor\|+ |
| STM0220_478_512 | 0.66 | -1.06 | 1251738\|dxr\|1-deoxy-D-xylulose 5-phosphate reductoisomerase\|+ |
| STM0221_389_423 | 0.01 | -2.10 | 1251739\|uppS\|undecaprenyl pyrophosphate synthase\|+ |
| STM0222_705_739 | 0.07 | -1.27 | 1251740\|cdsA\|CDP-diglyceride synthase\|+ |
| STM0223_482_516 | 0.01 | -1.72 | 1251741\|yaeL\|zinc metallopeptidase\|+ |
| STM0224_1416_1450 | 0.02 | -1.51 | 1251742\|yaeT\|outer membrane protein assembly factor YaeT\|+ |
| STM0225_97_131 | 0.03 | -1.53 | 1251743\|hlpA\|periplasmic chaperone\|+ |
| STM0226_363_397 | 0.74 | -1.05 | 1251744\|lpxD\|UDP-3-O-[3-hydroxymyristoyl] glucosamine N-acyltransferase\|+ |

| | | | |
|---|---|---|---|
| STM0227_217_251 | 0.07 | -1.49 | 1251745\|fabZ\|(3R)-hydroxymyristoyl-(acyl carrier protein) dehydratase\|+ |
| STM0228_385_419 | 0.15 | -1.25 | 1251746\|lpxA\|UDP-N-acetylglucosamine acyltransferase\|+ |
| STM0229_214_248 | 0.08 | 1.26 | 1251747\|lpxB\|lipid-A-disaccharide synthase\|+ |
| STM0230_393_427 | 0.02 | -1.57 | 1251748\|rnhB\|ribonuclease HII\|+ |
| STM0232_553_587 | 0.07 | -1.52 | 1251750\|accA\|acetyl-CoA carboxylase carboxyltransferase subunit alpha\|+ |
| STM0233_1213_1247 | 0.23 | 1.17 | 1251751\|STM0233\|putative endochitinase\|+ |
| STM0234_1303_1337 | 0.12 | 1.30 | 1251752\|ldcC\|lysine decarboxylase 2\|+ |
| STM0235_138_172 | 0.37 | 1.11 | 1251753\|yaeR\|hypothetical protein\|+ |
| STM0236_1078_1112 | 0.12 | 1.43 | 1251754\|tilS\|tRNA(Ile)-lysidine synthetase\|+ |
| STM0237_33_67 | 0.17 | 1.31 | 1251755\|rof\|Rho-binding antiterminator\|- |
| STM0238_177_211 | 0.21 | 1.32 | 1251756\|yaeP\|hypothetical protein\|- |
| STM0239_373_407 | 0.41 | -1.19 | 1251757\|yaeQ\|putative cytoplasmic protein\|+ |
| STM0240_9_43 | 0.03 | -1.39 | 1251758\|yaeJ\|hypothetical protein\|+ |
| STM0241_49_83 | 0.01 | 3.01 | 1251759\|cutF\|pseudo\|+ |
| STM0242_1184_1218 | 0.01 | -2.53 | 1251760\|proS\|prolyl-tRNA synthetase\|- |
| STM0243_313_347 | 0.02 | -1.53 | 1251761\|yaeB\|putative regulatory protein\|- |
| STM0244_185_219 | 0.44 | 1.15 | 1251762\|rcsF\|outer membrane lipoprotein\|- |
| STM0245_289_323 | 0.01 | 2.55 | 1251763\|metQ\|DL-methionine transporter substrate-binding subunit\|- |
| STM0246_329_363 | 0.01 | 4.10 | 1251764\|yaeE\|DL-methionine transporter permease subunit\|- |
| STM0247_609_643 | 0.01 | 4.53 | 1251765\|metN\|DL-methionine transporter ATP-binding subunit\|- |
| STM0248_49_83 | 0.24 | 1.38 | 1251766\|yaeD\|D D-heptose 1 7-bisphosphate phosphatase\|+ |
| STM0250_36_70 | 0.02 | -2.30 | 1251768\|ileV\|tRNA\|+ |
| STM0255_257_291 | 0.12 | 1.34 | 1251773\|dkgB\|2 5-diketo-D-gluconate reductase B\|+ |
| STM0256_129_163 | 0.43 | -1.11 | 1251774\|yafC\|putative transcriptional regulator\|- |
| STM0257_1009_1043 | 1.00 | 1.00 | 1251775\|STM0257\|putative drug efflux protein\|+ |
| STM0258_225_259 | 0.02 | 1.17 | 1251776\|yafD\|hypothetical protein\|+ |
| STM0259_385_419 | 0.06 | -1.39 | 1251777\|yafE\|putative methyltransferase\|+ |
| STM0260_369_403 | 0.00 | -2.65 | 1251778\|dniR\|predicted membrane-bound lytic murein transglycosylase D\|- |
| STM0261_521_555 | 0.07 | 1.84 | 1251779\|gloB\|predicted hydroxyacylglutathione hydrolase\|- |
| STM0262_257_291 | 0.09 | 1.17 | 1251780\|yafS\|putative SAM-dependent methyltransferase\|+ |
| STM0263_129_163 | 0.30 | 1.08 | 1251781\|rnhA\|ribonuclease H\|- |
| STM0264_49_83 | 0.62 | -1.04 | 1251782\|dnaQ\|DNA polymerase III subunit epsilon\|+ |
| STM0266_185_219 | 0.08 | -1.18 | 1251784\|STM0266\|putative cytoplasmic protein\|- |
| STM0268_1301_1335 | 0.17 | -1.20 | 1251786\|STM0268\|putative cytoplasmic protein\|- |
| STM0270_625_659 | 0.75 | 1.10 | 1251788\|STM0270\|putative cytoplasmic protein\|- |
| STM0271_129_163 | 0.18 | 1.16 | 1251789\|STM0271\|putative cytoplasmic protein\|- |

| | | | |
|---|---|---|---|
| STM0273_273_307 | 0.05 | 1.40 | 1251791lSTM0273lputative cytoplasmic proteinl+ |
| STM0274_894_928 | 0.05 | 1.54 | 1251792lSTM0274lputative cytoplasmic proteinl+ |
| STM0275.s_73_107 | 0.03 | 1.65 | 1251794lSTM0275.slputative cytoplasmic proteinl+ |
| STM0277_161_195 | 0.02 | 1.68 | 1251796lSTM0277lputative cytoplasmic proteinl+ |
| STM0278_127_161 | 0.42 | 1.25 | 1251797lSTM0278lputative periplasmic proteinl+ |
| STM0280_81_115 | 0.07 | 1.40 | 1251799lSTM0280lputative outer membrane lipoproteinl+ |
| STM0281_345_379 | 0.26 | 1.15 | 1251800lSTM0281lputative cytoplasmic proteinl+ |
| STM0283_117_151 | 0.00 | 2.35 | 1251802lSTM0283lputative inner membrane proteinl+ |
| STM0284_58_95 | 0.05 | 1.77 | 1251803lSTM0284lputative Shiga-like toxin A subunitl+ |
| STM0285_3479_3513 | 0.75 | -1.12 | 1251804lSTM0285lputative inner membrane proteinl+ |
| STM0286_85_120 | 0.03 | -1.44 | 1251805lSTM0286lputative cytoplasmic proteinl+ |
| STM0288_241_275 | 0.03 | 1.12 | 1251807lSTM0288lputative cytoplasmic proteinl+ |
| STM0289_1831_1865 | 0.01 | 2.37 | 1251808lSTM0289lputative cytoplasmic proteinl+ |
| STM0290_113_150 | 0.05 | 2.29 | 1251809lSTM0290lputative cytoplasmic proteinl+ |
| STM0291_3968_4002 | 0.36 | 1.16 | 1251810lSTM0291lputative RHS-like proteinl+ |
| STM0292_359_397 | 0.15 | 1.46 | 1251811lSTM0292lputative RHS-like proteinl+ |
| STM0293_57_95 | 0.36 | 1.14 | 1251812lSTM0293lputative cytoplasmic proteinl+ |
| STM0294_61_98 | 0.00 | 2.15 | 1251813lSTM0294lputative cytoplasmic proteinl+ |
| STM0294.1N_28_62 | 0.03 | -1.41 | 2673758lSTM0294.1Nlhypothetical proteinl- |
| STM0295_99_137 | 0.03 | 1.51 | 1251814lSTM0295lputative cytoplasmic proteinl+ |
| STM0299_465_500 | 0.16 | 1.31 | 1251818lsafAlputative outer membrane proteinl+ |
| STM0300_393_427 | 0.46 | 1.09 | 1251819lsafBlputative fimbrial assembly chaparonel+ |
| STM0301_1912_1946 | 0.40 | 1.10 | 1251820lsafClputative fimbrial usherl+ |
| STM0302_181_215 | 0.58 | -1.15 | 1251821lsafDlputative fimbrial subunitl+ |
| STM0303_321_355 | 0.13 | -1.55 | 1251822lybeJlputative xylanase/chitin deacetylasel+ |
| STM0304_161_195 | 0.23 | -1.27 | 1251823lsinRltranscriptional regulatorl+ |
| STM0305_161_195 | 0.81 | 1.07 | 1251824lSTM0305lputative cytoplasmic proteinl+ |
| STM0306_401_435 | 0.01 | 2.25 | 1251825lSTM0306lSapA-like proteinl- |
| STM0307_229_263 | 0.01 | 3.42 | 1251826lSTM0307lVirG-like proteinl- |
| STM0308_417_451 | 0.02 | 1.34 | 1251827lyafVlhypothetical proteinl- |
| STM0309_1830_1864 | 0.93 | 1.01 | 1251828lfadElacyl-CoA dehydrogenasel- |
| STM0310_353_387 | 0.03 | -1.13 | 1251829lgmhAlphosphoheptose isomerasel+ |
| STM0311_321_355 | 0.63 | 1.05 | 1251830lyafJlputative glutamine amidotransferasel+ |
| STM0312_273_307 | 0.00 | -4.13 | 1251831lyafKlputative periplasmic proteinl- |
| STM0313_761_795 | 0.07 | 1.21 | 1251832ldinPlDNA polymerase IVl+ |
| STM0314_1038_1072 | 0.09 | 1.68 | 1251833lSTM0314lpseudol+ |

| | | | |
|---|---|---|---|
| STM0315_289_323 | 0.28 | -1.17 | 1251834lprfHlpeptide chain release factor-like proteinl+ |
| STM0316_1387_1421 | 0.03 | 1.50 | 1251835lpepDlaminoacyl-histidine dipeptidasel- |
| STM0317_137_171 | 0.05 | -1.95 | 1251836lgptlxanthine-guanine phosphoribosyltransferasel+ |
| STM0318_374_408 | 0.56 | -1.04 | 1251837lfrsAlfermentation/respiration switch proteinl+ |
| STM0319_241_275 | 0.04 | 1.58 | 1251838lcrllDNA-binding transcriptional regulator Crll+ |
| STM0320_190_224 | 0.00 | 2.72 | 1251839lphoElouter membrane phosphoporin protein El- |
| STM0321_361_395 | 0.09 | -1.50 | 1251840lproBlgamma-glutamyl kinasel+ |
| STM0322_348_382 | 0.74 | -1.22 | 1251841lproAlgamma-glutamyl phosphate reductasel+ |
| STM0325_579_613 | 0.01 | 1.52 | 1251844lSTM0325lputative truncated IS3 transposasel- |
| STM0327_129_163 | 0.00 | -2.33 | 1251846lSTM0327lputative cytoplasmic proteinl+ |
| STM0328.s_435_469 | 0.04 | 1.33 | 1251847lSTM0328.slputative permeasel+ |
| STM0329_431_465 | 0.04 | -1.35 | 1251848lSTM0329lisopropylmalate isomerase large subunitl+ |
| STM0330_337_371 | 0.86 | -1.02 | 1251849lSTM0330lputative 3-isopropylmalate isomerasel+ |
| STM0332_609_643 | 0.11 | 1.54 | 1251851lSTM0332lputative hydrolase/acyltransferasel+ |
| STM0333_769_803 | 0.25 | -1.12 | 1251852lSTM0333lputative transcriptional regulatorl+ |
| STM0335_19_54 | 0.96 | 1.02 | 1251854lSTM0335lputative outer membrane proteinl+ |
| STM0336_177_211 | 0.02 | 1.49 | 1251855lstbElputative fimbrial chaparonel- |
| STM0338_1787_1821 | 0.94 | -1.01 | 1251857lstbClputative fimbrial usherl- |
| STM0339_193_227 | 0.04 | 2.35 | 1251858lstbBlputative fimbrial chaperonel- |
| STM0340_283_317 | 0.41 | 1.26 | 1251859lstbAlputative fimbrial major subunitl- |
| STM0341_275_309 | 0.02 | 2.96 | 1251860lSTM0341lputative inner membrane proteinl+ |
| STM0342_49_83 | 0.47 | -1.09 | 1251861lSTM0342lputative periplasmic proteinl+ |
| STM0343_1128_1162 | 0.15 | 1.51 | 1251862lSTM0343lhypothetical proteinl+ |
| STM0344_369_403 | 0.16 | 1.81 | 1251863lSTM0344lputative response regulatorl+ |
| STM0345_385_419 | 0.91 | -1.04 | 1251864lSTM0345lputative inner membrane proteinl+ |
| STM0346_337_371 | 0.07 | 2.93 | 1251865lSTM0346lputative outer membrane proteinl+ |
| STM0347_249_283 | 0.01 | 1.93 | 1251866lSTM0347lputative response regulatorl+ |
| STM0348_45_79 | 0.03 | 3.27 | 1251867lSTM0348lputative inner membrane proteinl+ |
| STM0349_65_99 | 0.16 | 1.38 | 1251868lSTM0349lputative outer membrane lipoproteinl+ |
| STM0350.S_1307_1341 | 0.65 | 1.08 | 1251869lSTM0350.Slouter membrane efflux-like proteinl- |
| STM0351_2377_2411 | 0.20 | 1.43 | 1251870lSTM0351lputative cation efflux system proteinl- |
| STM0352.S_607_641 | 0.15 | 1.71 | 1251871lSTM0352.Slputative cation efflux pumpl- |
| STM0353_1658_1692 | 0.03 | -1.45 | 1251872lSTM0353lputative cation transport ATPasel+ |
| STM0354_365_399 | 0.95 | -1.01 | 1251873lSTM0354lputative transcriptional regulatorl+ |
| STM0355_62_96 | 0.04 | -1.41 | 1251874lSTM0355lputative copper chaperonel+ |
| STM0356_479_513 | 0.00 | -2.40 | 1251875lSTM0356lputative inner membrane proteinl+ |

| | | | |
|---|---|---|---|
| STM0357_1088_1122 | 0.02 | 1.48 | 1251876lmodlDNA methylasel+ |
| STM0359_2_40 | 0.02 | 2.25 | 1251878lSTM0359lputative cytoplasmic proteinl+ |
| STM0360_949_983 | 0.40 | 1.24 | 1251879lSTM0360lcytochrome BD2 subunit Il+ |
| STM0361_16_50 | 0.89 | 1.03 | 1251880lSTM0361lcytochrome BD2 subunit IIl+ |
| STM0362_65_99 | 0.72 | 1.09 | 1251881lSTM0362lputative cytoplasmic proteinl+ |
| STM0363_118_152 | 0.00 | 2.39 | 1251882lSTM0363lputative transcriptional regulatorl+ |
| STM0364_1398_1432 | 0.88 | 1.04 | 1251883lfoxAlferrioxamine receptorl+ |
| STM0365_4_38 | 0.00 | -4.16 | 1251884lyahNlputative transport proteinl- |
| STM0366_95_129 | 0.32 | -1.24 | 1251885lyahOlhypothetical proteinl+ |
| STM0367_1107_1141 | 0.00 | -4.26 | 1251886lprpRlprp operon regulatorl- |
| STM0368_65_99 | 0.09 | -1.26 | 1251887lprpBl2-methylisocitrate lyasel+ |
| STM0369_471_505 | 0.46 | 1.44 | 1251888lprpClmethylcitrate synthasel+ |
| STM0370_1029_1063 | 0.06 | 1.41 | 1251889lprpDl2-methylcitrate dehydratasel+ |
| STM0372_553_587 | 0.02 | 1.63 | 1251891lhemBldelta-aminolevulinic acid dehydratasel- |
| STM0373_2770_2804 | 0.82 | 1.02 | 1251892lyaiUlflagellar proteinl+ |
| STM0374_257_291 | 0.01 | 2.24 | 1251893lyaiVlputative DNA-binding transcriptional regulatorl+ |
| STM0375_132_166 | 0.08 | -1.29 | 1251894lampHlbeta-lactamase/D-alanine carboxypeptidasel- |
| STM0376_1150_1184 | 0.13 | 1.40 | 1251895lsbmAltransport proteinl+ |
| STM0377_1008_1042 | 0.04 | 1.26 | 1251896lyaiWlputative outer membrane lipoproteinl+ |
| STM0378_165_199 | 0.00 | 4.41 | 1251897lyaiYlputative inner membrane proteinl- |
| STM0379_125_159 | 0.00 | -2.93 | 1251898lyaiZlputative inner membrane proteinl+ |
| STM0380_256_290 | 0.01 | -1.64 | 1251899lddllD-alanyl-alanine synthetase Al- |
| STM0381_625_659 | 0.29 | 1.23 | 1251900lSTM0381lputative inner membrane proteinl+ |
| STM0382_213_247 | 0.02 | 1.31 | 1251901lSTM0382lputative permeasel+ |
| STM0383_197_231 | 0.03 | 1.93 | 1251902lyaiBlhypothetical proteinl+ |
| STM0384_129_163 | 0.01 | 4.71 | 1251903lpsiFlhypothetical proteinl+ |
| STM0385_1010_1044 | 0.33 | -1.11 | 1251904lyaiClhypothetical proteinl+ |
| STM0386_33_67 | 0.54 | -1.04 | 1251905lproClpyrroline-5-carboxylate reductasel- |
| STM0387_161_195 | 0.77 | -1.04 | 1251906lyaiIlhypothetical proteinl+ |
| STM0388_257_291 | 0.03 | -3.18 | 1251907laroLlshikimate kinase IIl+ |
| STM0389_13_47 | 0.18 | -1.49 | 1251908lyaiAlhypothetical proteinl+ |
| STM0390_25_59 | 0.01 | -2.92 | 1251909laroMlhypothetical proteinl+ |
| STM0391_153_187 | 0.72 | 1.09 | 1251910lyaiElhypothetical proteinl+ |
| STM0392_657_691 | 0.51 | -1.18 | 1251911lrdgClrecombination associated proteinl- |
| STM0393_289_323 | 0.13 | -1.32 | 1251912lyajFlfructokinasel+ |
| STM0394_1038_1072 | 0.02 | 1.53 | 1251913laraJlprotein AraJl- |

| | | | |
|---|---|---|---|
| STM0395_2702_2736 | 0.17 | -1.29 | 1251914lsbcClexonuclease subunit SbcCl- |
| STM0396_844_878 | 0.43 | 1.11 | 1251915lsbcDlexonuclease subunit SbcDl- |
| STM0397_513_547 | 0.67 | -1.09 | 1251916lphoBlDNA-binding response regulator in two-component regulatory system with PhoR (or CreC)l+ |
| STM0398_1097_1131 | 0.23 | -1.38 | 1251917lphoRlphosphate regulon sensor proteinl+ |
| STM0399_977_1011 | 0.18 | 1.23 | 1251918lbrnQlbranched-chain amino acid transporterl+ |
| STM0400_884_918 | 0.38 | -1.05 | 1251919lproYlputative proline transporterl+ |
| STM0401_1251_1285 | 0.96 | -1.01 | 1251920lmalZlmaltodextrin glucosidasel+ |
| STM0402_385_419 | 0.05 | 1.84 | 1251921lSTM0402lputative thiol-alkyl hydroperoxide reductasel- |
| STM0403_129_163 | 0.30 | 1.22 | 1251922lyajBlacyl carrier protein phosphodiesterasel- |
| STM0404_578_612 | 0.04 | -2.22 | 1251923lqueAlS-adenosylmethionine:tRNA ribosyltransferase-isomerasel+ |
| STM0405_961_995 | 0.55 | -1.08 | 1251924ltgtlqueuine tRNA-ribosyltransferasel+ |
| STM0406_273_307 | 0.02 | -2.43 | 1251925lyajClpreprotein translocase subunit YajCl+ |
| STM0407_1041_1075 | 0.01 | -2.92 | 1251926lsecDlpreprotein translocase subunit SecDl+ |
| STM0408_609_643 | 0.00 | -2.04 | 1251927lsecFlpreprotein translocase subunit SecFl+ |
| STM0409_65_99 | 0.24 | 1.19 | 1251928lSTM0409lhypothetical proteinl+ |
| STM0410_81_115 | 0.00 | -1.37 | 1251929lSTM0410lputative regulatory proteinl+ |
| STM0411_289_323 | 0.08 | 1.54 | 1251930lyajDlhypothetical proteinl+ |
| STM0412_93_127 | 0.39 | 1.16 | 2673775lSTM0412l-l- |
| STM0413_257_291 | 0.00 | -4.46 | 1251932ltsxlnucleoside channell- |
| STM0414_325_359 | 0.02 | 1.28 | 1251933lyajIlhypothetical proteinl- |
| STM0415_97_131 | 0.01 | 2.50 | 1251934lybaDlhypothetical proteinl+ |
| STM0416_873_907 | 0.19 | -1.18 | 1251935lribDlbifunctional diaminohydroxyphosphoribosylaminopyrimidine deaminase/5-amino-6-(5-phosphoribosylamino)uracil reductasel+ |
| STM0417_353_387 | 0.94 | -1.02 | 1251936lribHlriboflavin synthase subunit betal+ |
| STM0418_121_155 | 0.29 | 1.17 | 1251937lnusBltranscription antitermination protein NusBl+ |
| STM0419_849_883 | 0.17 | -1.32 | 1251938lthiLlthiamine monophosphate kinasel+ |
| STM0420_225_259 | 0.07 | -1.33 | 1251939lpgpAlphosphatidylglycerophosphatase Al+ |
| STM0421_917_951 | 0.07 | -1.83 | 1251940lyajOlputative oxidoreductasel- |
| STM0422_1200_1234 | 0.10 | -1.29 | 1251941ldxsl1-deoxy-D-xylulose-5-phosphate synthasel- |
| STM0423_225_259 | 0.03 | -1.24 | 1251942lispAlgeranyltranstransferasel- |
| STM0424_33_67 | 0.02 | -1.34 | 1251943lxseBlexodeoxyribonuclease VII small subunitl- |
| STM0425_482_516 | 0.06 | -1.48 | 1251944lthiIlthiamine biosynthesis protein ThiIl+ |
| STM0426_433_467 | 0.05 | 1.30 | 1251945lphnVl2-aminoethylphosphonate transporterl- |
| STM0427_375_409 | 0.49 | -1.22 | 1251946lphnUl2-aminoethylphosphonate transporterl- |
| STM0428_719_753 | 0.26 | 1.14 | 1251947lphnTl2-aminoethylphosphonate transporterl- |

| | | | |
|---|---|---|---|
| STM0430_513_547 | 0.21 | 1.17 | 1251949lphnRl2-aminoethylphosphonate transport proteinl- |
| STM0431_281_315 | 0.73 | 1.06 | 1251950lphnWl2-aminoethylphosphonate transportl+ |
| STM0432_257_291 | 0.90 | -1.01 | 1251951lphnXl2-aminoethylphosphonate transportl+ |
| STM0433_129_163 | 0.01 | 2.71 | 1251952lthiJlhypothetical proteinl- |
| STM0434_769_803 | 0.02 | 1.73 | 1251953lapbAl2-dehydropantoate 2-reductasel- |
| STM0435_97_131 | 0.02 | -1.78 | 1251954lyajQlnucleotide-binding proteinl+ |
| STM0436.S_1006_1040 | 0.03 | 1.60 | 1251955lyajRlputative transport proteinl- |
| STM0436A_89_123 | 0.10 | 1.25 | 1251956lSTM0436Al-l- |
| STM0438_769_803 | 0.02 | 2.14 | 1251958lSTM0438ltetratricopeptide repeat proteinl+ |
| STM0439_849_883 | 0.03 | 2.38 | 1251959lcyoElprotoheme IX farnesyltransferasel- |
| STM0440_61_95 | 0.06 | -1.76 | 1251960lcyoDlcytochrome o ubiquinol oxidase subunit IVl- |
| STM0441_229_263 | 0.00 | -2.53 | 1251961lcyoClcytochrome o ubiquinol oxidase subunit IIIl- |
| STM0442_1393_1427 | 0.01 | -1.47 | 1251962lcyoBlcytochrome o ubiquinol oxidase subunit Il- |
| STM0443_129_163 | 0.00 | -2.84 | 1251963lcyoAlcytochrome o ubiquinol oxidase subunit IIl- |
| STM0444_1333_1367 | 0.03 | 1.22 | 1251964lampGlmuropeptide transporterl- |
| STM0445_293_327 | 0.03 | -1.81 | 1251965lyajGlhypothetical proteinl- |
| STM0446_257_291 | 0.11 | 1.50 | 1251966lbolAltranscriptional regulator BolAl+ |
| STM0447_1036_1070 | 0.19 | 1.54 | 1251967ltigltrigger factorl+ |
| STM0448_145_179 | 0.00 | 1.31 | 1251968lclpPlATP-dependent Clp protease proteolytic subunitl+ |
| STM0449_273_307 | 0.06 | -1.18 | 1251969lclpXlATP-dependent protease ATP-binding subunitl+ |
| STM0450_1708_1742 | 0.01 | 2.33 | 1251970llonlDNA-binding ATP-dependent protease Lal+ |
| STM0451_225_259 | 0.00 | -4.14 | 1251971lhupBltranscriptional regulator HU subunit betal+ |
| STM0452_1577_1611 | 0.02 | 1.80 | 1251972lcypDlpeptidyl-prolyl cis-trans isomerase (rotamase D)l+ |
| STM0453_141_175 | 0.12 | 1.20 | 1251973lybaVlputative DNA uptake proteinl+ |
| STM0454_189_223 | 0.00 | -3.79 | 1251974lybaWlputative esterasel+ |
| STM0455_609_643 | 0.03 | 1.83 | 1251975lybaXlqueuosine biosynthesis protein QueCl- |
| STM0456_1422_1456 | 0.00 | -2.92 | 1251976lybaElputative ABC transporter periplasmic binding proteinl- |
| STM0457_721_755 | 0.42 | 1.07 | 1251977lcoflputative hydrolasel+ |
| STM0458_953_987 | 0.06 | 1.91 | 1251978lSTM0458lputative cysteine synthase/cystathionine beta-synthasel- |
| STM0459_353_387 | 0.07 | 1.46 | 1251979lybaOlputative transcriptional regulatorl+ |
| STM0460_1342_1376 | 0.46 | 1.14 | 1251980lmdlAlputative multidrug transporter membrane\ATP-binding componentsl+ |
| STM0461_1071_1105 | 0.26 | -1.14 | 1251981lmdlBlputative multidrug transporter membrane\ATP-binding componentsl+ |
| STM0461.1n_377_411 | 0.14 | 1.53 | 2673746lSTM0461.1nlhypothetical proteinl- |
| STM0462_171_205 | 0.01 | 1.81 | 1251982lglnKlnitrogen regulatory protein P-II 2l+ |

| | | | |
|---|---|---|---|
| STM0463_1184_1218 | 0.03 | 1.40 | 1251983lamtBlammonium transporterl+ |
| STM0464_225_259 | 0.02 | 1.70 | 1251984ltesBlacyl-CoA thioesterase IIl- |
| STM0465_17_51 | 0.05 | 1.67 | 1251985lybaYlhypothetical proteinl+ |
| STM0466_342_376 | 0.51 | -1.17 | 1251986lybaZlputative methyltransferasel- |
| STM0467_17_51 | 0.62 | 1.56 | 1251987lffslmisc_RNAl+ |
| STM0468_656_690 | 0.18 | -1.27 | 1251988lylaBlhypothetical proteinl- |
| STM0469_5_39 | 0.03 | -12.11 | 1251989lrpmE2l50S ribosomal protein L31 type Bl+ |
| STM0470_49_83 | 0.05 | -4.97 | 1251990lrpmJl50S ribosomal protein L36l+ |
| STM0471_289_323 | 0.00 | 2.54 | 1251991lylaClputative inner membrane proteinl- |
| STM0472_73_107 | 0.66 | 1.04 | 1251992lmaalmaltose O-acetyltransferasel- |
| STM0473_125_159 | 0.05 | 1.61 | 1251993lhhalhemolysin expression-modulating proteinl- |
| STM0474_145_179 | 0.00 | 1.99 | 1251994lybaJlhypothetical proteinl- |
| STM0475_2623_2657 | 0.19 | 1.13 | 1251995lacrBlacridine efflux pumpl- |
| STM0476_211_245 | 0.10 | 1.33 | 1251996lacrAlacridine efflux pumpl- |
| STM0477_185_221 | 0.84 | -1.02 | 1251997lacrRlDNA-binding transcriptional repressorl+ |
| STM0478_2876_2910 | 0.28 | -1.19 | 1251998laefAlpotassium efflux protein KefAl+ |
| STM0479_585_619 | 0.05 | 1.58 | 1251999lSTM0479lputative transposasel- |
| STM0480_25_59 | 0.05 | 1.21 | 1252000lybaMlhypothetical proteinl- |
| STM0481_97_131 | 0.88 | 1.05 | 1252001lpriClprimosomal replication protein N''l- |
| STM0482_129_163 | 0.44 | -1.14 | 1252002lybaNlhypothetical proteinl+ |
| STM0483_289_323 | 0.02 | -1.62 | 1252003laptladenine phosphoribosyltransferasel+ |
| STM0484_1046_1080 | 0.54 | -1.07 | 1252004ldnaXlDNA polymerase III subunits gamma and taul+ |
| STM0485_5_40 | 0.87 | 1.02 | 1252005lybaBlhypothetical proteinl+ |
| STM0486_197_231 | 0.60 | 1.31 | 1252006lrecRlrecombination protein RecRl+ |
| STM0487.S_1420_1454 | 0.96 | 1.01 | 1252007lhtpGlheat shock protein 90l+ |
| STM0488_129_163 | 0.01 | -3.73 | 1252008ladkladenylate kinasel+ |
| STM0489_321_355 | 0.21 | 1.12 | 1252009lhemHlferrochelatasel+ |
| STM0490_673_707 | 0.01 | -2.25 | 1252010laeslacetyl esterasel- |
| STM0491_1106_1140 | 0.00 | -1.59 | 1252011lgsklinosine-guanosine kinasel+ |
| STM0492_878_912 | 0.00 | 1.76 | 1252012lybaLlputative cation:proton antiport proteinl- |
| STM0493_238_272 | 0.59 | -1.12 | 1252013lfsrlputative transport proteinl- |
| STM0494_1110_1144 | 0.03 | -1.91 | 1252014lushAlbifunctional UDP-sugar hydrolase/5'-nucleotidase periplasmic precursorl+ |
| STM0495_113_147 | 0.05 | 1.26 | 1252015lybaKlhypothetical proteinl- |
| STM0496_641_675 | 0.37 | 1.28 | 1252016lybaPlputative cytoplasmic proteinl- |
| STM0497_205_239 | 0.03 | 2.41 | 1252017lSTM0497lputative periplasmic proteinl- |

| | | | |
|---|---|---|---|
| STM0498_2447_2481 | 0.15 | -1.79 | 1252018\|copA\|copper transporter\|- |
| STM0499_353_387 | 0.01 | 2.11 | 1252019\|cueR\|DNA-binding transcriptional activator of copper-responsive regulon genes\|+ |
| STM0500_113_147 | 0.01 | 1.78 | 1252020\|ybbJ\|hypothetical protein\|- |
| STM0502_609_643 | 0.22 | -1.19 | 1252022\|ybbL\|putative ABC transporter ATP-binding protein YbbL\|+ |
| STM0503_177_211 | 0.07 | 1.27 | 1252023\|ybbM\|putative transport protein\|+ |
| STM0504_665_699 | 0.29 | 1.32 | 1252024\|ybbN\|putative thioredoxin protein\|- |
| STM0505_129_163 | 0.16 | -1.12 | 1252025\|ybbO\|short chain dehydrogenase\|- |
| STM0506_433_467 | 0.07 | -1.46 | 1252026\|tesA\|multifunctional acyl-CoA thioesterase I and protease I and lysophospholipase L1\|- |
| STM0507_73_107 | 0.00 | 2.24 | 1252027\|ybbA\|putative ABC transporter ATP-binding protein YbbA\|+ |
| STM0508_1752_1786 | 0.02 | 1.39 | 1252028\|ybbP\|putative inner membrane protein\|+ |
| STM0509_935_969 | 0.79 | -1.06 | 1252029\|STM0509\|putative outer membrane protein\|+ |
| STM0510_492_526 | 0.06 | -1.43 | 1252030\|sfbA\|putative ABC-type transport system ATPase component\|+ |
| STM0511_850_884 | 0.13 | -1.23 | 1252031\|sfbB\|putative ABC-type transport system ATPase component\|+ |
| STM0512_385_419 | 0.01 | -2.26 | 1252032\|sfbC\|putative ABC transporter permease component\|+ |
| STM0513_300_334 | 0.01 | 1.64 | 1252033\|ybbB\|tRNA 2-selenouridine synthase\|- |
| STM0516_641_675 | 0.21 | 1.15 | 1252036\|allR\|DNA-binding transcriptional repressor\|+ |
| STM0518_305_339 | 0.26 | -1.47 | 1252038\|gip\|hydroxypyruvate isomerase\|+ |
| STM0522_987_1021 | 0.26 | 1.29 | 1252042\|allP\|allantoin permease\|+ |
| STM0523_363_397 | 0.39 | -1.30 | 1252043\|allB\|allantoinase\|+ |
| STM0525_1078_1112 | 0.03 | 2.82 | 1252045\|glxK\|glycerate kinase II\|+ |
| STM0526_161_195 | 0.58 | 1.18 | 1252046\|ylbA\|hypothetical protein\|- |
| STM0528_195_229 | 0.15 | 1.88 | 1252048\|allD\|ureidoglycolate dehydrogenase\|- |
| STM0529_1162_1196 | 0.05 | 1.18 | 1252049\|fdrA\|membrane protein FdrA\|+ |
| STM0531_389_423 | 0.69 | -1.04 | 1252051\|ylbF\|putative cytoplasmic protein\|+ |
| STM0533_853_887 | 0.01 | -2.73 | 1252053\|purK\|phosphoribosylaminoimidazole carboxylase ATPase subunit\|- |
| STM0534_169_203 | 0.01 | -4.50 | 1252054\|purE\|phosphoribosylaminoimidazole carboxylase catalytic subunit\|- |
| STM0535_321_355 | 0.96 | 1.01 | 1252055\|lpxH\|UDP-2 3-diacylglucosamine hydrolase\|- |
| STM0536_361_395 | 0.09 | -1.70 | 1252056\|ppiB\|peptidyl-prolyl cis-trans isomerase B (rotamase B)\|- |
| STM0537_515_549 | 0.07 | 1.25 | 1252057\|cysS\|cysteinyl-tRNA synthetase\|+ |
| STM0538_257_291 | 0.83 | -1.05 | 1252058\|STM0538\|putative outer membrane protein\|- |
| STM0539_377_411 | 0.16 | -1.18 | 1252059\|STM0539\|putative inner membrane protein\|- |
| STM0540_149_183 | 0.64 | 1.04 | 1252060\|ybcI\|putative membrane-bound metal-dependent hydrolase\|- |
| STM0541_152_187 | 0.05 | 1.25 | 1252061\|ybcJ\|hypothetical protein\|- |

| | | | |
|---|---|---|---|
| STM0542_609_643 | 0.07 | 1.26 | 1252062\|folD\|bifunctional 5 10-methylene-tetrahydrofolate dehydrogenase/ 5 10-methylene-tetrahydrofolate cyclohydrolase\|- |
| STM0543_65_99 | 0.07 | -2.16 | 1252063\|fimA\|fimbrin\|+ |
| STM0545_218_252 | 0.02 | 2.45 | 1252065\|fimC\|periplasmic chaperone\|+ |
| STM0547_969_1003 | 0.04 | -1.32 | 1252067\|fimH\|minor fimbrial subunit\|+ |
| STM0550_41_75 | 0.13 | 1.37 | 1252070\|fimY\|putative regulatory protein\|- |
| STM0552_167_201 | 0.02 | 1.39 | 1252072\|fimW\|putative fimbrial protein\|- |
| STM0554_17_51 | 0.69 | 1.17 | 1252074\|STM0554\|integrase\|- |
| STM0555_337_371 | 0.64 | 1.15 | 2673757\|STM0555\|pseudo\|- |
| STM0558_665_699 | 0.05 | 1.76 | 1252078\|yfdH\|putative glycosyltransferase\|- |
| STM0559_257_291 | 0.71 | 1.14 | 1252079\|rfbI\|putative glycosyl translocase\|- |
| STM05559.T1_9_43 | 0.03 | 1.91 | 2673761\|STM05559.T1\|tRNA\|+ |
| STM0560_5_39 | 0.42 | 1.22 | 1252080\|STM0560\|pseudo\|- |
| STM0561_43_77 | 0.03 | 1.21 | 1252081\|STM0561\|sensor kinase\|- |
| STM0562_169_203 | 0.04 | -1.26 | 2673759\|STM0562\|-\|+ |
| STM0563_241_275 | 0.00 | 1.77 | 1252083\|STM0563\|putative transcriptional regulator\|- |
| STM0564_583_617 | 0.95 | 1.01 | 1252084\|STM0564\|pyridine nucleotide-disulfide oxidoreductase\|+ |
| STM0565_65_99 | 0.76 | 1.07 | 1252085\|STM0565\|putative periplasmic protein\|+ |
| STM0567_321_355 | 0.05 | 1.45 | 1252087\|STM0567\|putative DNA repair ATPase\|- |
| STM0568_1292_1326 | 0.94 | -1.01 | 1252088\|pheP\|phenylalanine transporter\|+ |
| STM0569_1017_1051 | 0.02 | -1.37 | 1252089\|ybdG\|hypothetical protein\|- |
| STM0570_1388_1422 | 0.15 | -1.16 | 1252090\|apeE\|outer membrane esterase\|+ |
| STM0571_1731_1765 | 0.12 | 1.50 | 1252091\|STM0571\|putative inner membrane protein\|+ |
| STM0572_849_883 | 0.16 | -1.24 | 1252092\|STM0572\|putative phosphosugar isomerase\|- |
| STM0573_813_847 | 0.03 | 2.22 | 1252093\|STM0573\|putative inner membrane protein\|- |
| STM0575_113_147 | 0.14 | 1.52 | 1252095\|STM0575\|putative inner membrane protein\|- |
| STM0576_113_147 | 0.01 | 2.33 | 1252096\|STM0576\|putative PTS system mannose-specific enzyme IIAB\|- |
| STM0577_33_67 | 0.41 | 1.10 | 1252097\|STM0577\|putative PTS system mannose-specific enzyme IIAB\|- |
| STM0578_417_451 | 0.16 | -1.48 | 1252098\|nfnB\|dihydropteridine reductase\|- |
| STM0579_117_151 | 0.33 | 1.08 | 1252099\|ybdF\|hypothetical protein\|- |
| STM0580_345_379 | 0.01 | -1.92 | 1252100\|STM0580\|putative regulatory protein\|- |
| STM0581_61_95 | 0.25 | 1.08 | 1252101\|STM0581\|putative regulatory protein\|+ |
| STM0582_3_37 | 0.68 | -1.11 | 1252102\|ybdJ\|putative inner membrane protein\|- |
| STM0583_696_730 | 0.03 | -1.29 | 1252103\|ybdK\|putative cytoplasmic protein\|- |
| STM0584_513_547 | 0.72 | -1.15 | 1252104\|entD\|phosphopantetheinyltransferase component of enterobactin synthase multienzyme complex\|- |

| | | | |
|---|---|---|---|
| STM0585_2025_2059 | 0.18 | -1.44 | 1252105\|fepA\|outer membrane ferric enterobactin receptor precursor\|- |
| STM0588_3318_3352 | 0.20 | -1.37 | 1252108\|entF\|enterobactin synthase subunit F\|+ |
| STM0589_394_428 | 0.06 | 1.97 | 1252109\|fepE\|ferric enterobactin/enterochelin transporter\|+ |
| STM0590_721_755 | 0.40 | -1.17 | 1252110\|fepC\|iron-enterobactin transporter ATP-binding protein\|- |
| STM0591_369_403 | 0.40 | -1.13 | 1252111\|fepG\|iron-enterobactin transporter permease\|- |
| STM0592_889_923 | 0.24 | -1.22 | 1252112\|fepD\|iron-enterobactin transporter membrane protein\|- |
| STM0593_310_344 | 0.87 | -1.03 | 1252113\|ybdA\|enterobactin exporter EntS\|+ |
| STM0594_225_259 | 0.16 | -1.33 | 1252114\|fepB\|iron-enterobactin transporter periplasmic binding protein\|- |
| STM0595_241_275 | 0.36 | 1.19 | 1252115\|entC\|isochorismate synthase\|+ |
| STM0596_992_1026 | 0.97 | -1.01 | 1252116\|entE\|enterobactin synthase subunit E\|+ |
| STM0597_769_803 | 0.01 | 1.42 | 1252117\|entB\|2 3-dihydro-2 3-dihydroxybenzoate synthetase\|+ |
| STM0598_705_739 | 0.62 | -1.07 | 1252118\|entA\|2 3-dihydroxybenzoate-2 3-dehydrogenase\|+ |
| STM0599_187_221 | 0.23 | -1.18 | 1252119\|ybdB\|hypothetical protein\|+ |
| STM0600_1299_1333 | 0.00 | -9.62 | 1252120\|cstA\|carbon starvation protein\|+ |
| STM0601_67_101 | 0.00 | -5.90 | 1252121\|ybdD\|putative cytoplasmic protein\|+ |
| STM0602_938_972 | 0.01 | 15.08 | 1252122\|ybdH\|putative glycerol dehydrogenase\|- |
| STM0603_254_288 | 0.01 | 9.24 | 1252123\|ybdL\|putative aminotransferase\|+ |
| STM0604_257_291 | 0.43 | 1.20 | 1252124\|ybdM\|putative transcriptional regulator\|- |
| STM0605_773_807 | 0.07 | 2.22 | 1252125\|ybdN\|putative 3'-phosphoadenosine 5'-phosphosulfate sulfotransferase\|- |
| STM0606_681_715 | 0.02 | 2.18 | 1252126\|ybdO\|putative transcriptional regulator\|- |
| STM0607_417_451 | 0.37 | -1.11 | 1252127\|dsbG\|disulfide isomerase/thiol-disulfide oxidase\|- |
| STM0608_65_99 | 0.99 | -1.00 | 1252128\|ahpC\|alkyl hydroperoxide reductase subunit C\|+ |
| STM0609_935_969 | 0.15 | 1.38 | 1252129\|ahpF\|alkyl hydroperoxide reductase F52a subunit\|+ |
| STM0610_161_195 | 0.26 | 1.56 | 1252130\|STM0610\|putative anaerobic dehydrogenase component\|+ |
| STM0611_2113_2147 | 0.33 | -1.11 | 1252131\|STM0611\|putative oxidoreductase protein\|+ |
| STM0612_201_235 | 0.19 | -1.28 | 1252132\|STM0612\|putative hydrogenase protein\|+ |
| STM0613_725_759 | 0.20 | -1.14 | 1252133\|STM0613\|putative hydrogenase protein\|+ |
| STM0614_209_243 | 0.05 | 1.66 | 1252134\|ybdQ\|putative universal stress protein\|- |
| STM0615_816_850 | 0.03 | -1.37 | 1252135\|ybdR\|putative dehydrogenase\|+ |
| STM0616_25_59 | 0.03 | -1.63 | 1252136\|rnk\|nucleoside diphosphate kinase regulator\|- |
| STM0617_497_531 | 0.01 | 2.83 | 1252137\|rna\|ribonuclease I\|- |
| STM0618_1121_1155 | 0.53 | -1.39 | 1252138\|citT\|citrate/succinate transport antiport protein\|- |
| STM0619_785_819 | 0.47 | -1.14 | 1252139\|citG\|triphosphoribosyl-dephospho-CoA synthase\|- |
| STM0620_481_515 | 0.11 | -1.21 | 1252140\|citX\|2'-(5''-triphosphoribosyl)-3'-dephospho-CoA:apo-citrate lyase\|- |
| STM0622_193_227 | 0.03 | -1.25 | 1252142\|citE\|citrate lyase beta chain\|- |

| | | | |
|---|---|---|---|
| STM0624_798_832 | 0.49 | 1.07 | 1252144lcitClcitrate lyase synthetasel- |
| STM0625_711_745 | 0.01 | -1.59 | 1252145ldpiBlsensory histidine kinasel+ |
| STM0626_465_499 | 0.02 | -1.42 | 1252146ldpiAlDNA-binding response regulator in two-component regulatory system with citAl+ |
| STM0628_369_403 | 0.02 | 1.33 | 1252148lpagPlpalmitoyl transferase for Lipid Al+ |
| STM0629_145_179 | 0.01 | -2.83 | 1252149lcspElcold shock protein El+ |
| STM0630_49_83 | 0.26 | -1.16 | 1252150lccrBlcamphor resistance protein CrcBl- |
| STM0631_433_467 | 0.11 | 1.21 | 1252151lybeMlputative hydrolasel+ |
| STM0632_91_125 | 0.07 | 1.64 | 1252152ltatEltwin arginine translocase protein El+ |
| STM0633_617_651 | 0.19 | -1.23 | 1252153llipAllipoyl synthasel- |
| STM0634_257_291 | 0.04 | -1.68 | 1252154lybeFlputative DNA-binding transcriptional regulatorl- |
| STM0635.S_273_307 | 0.00 | 2.02 | 1252155llipBllipoyltransferasel- |
| STM0636_81_115 | 0.09 | -1.26 | 1252156lybeDlhypothetical proteinl- |
| STM0638_643_677 | 0.05 | -1.29 | 1252158lrlpAlrare lipoprotein Al- |
| STM0639_426_460 | 0.71 | 1.08 | 1252159lmrdBlcell wall shape-determining proteinl- |
| STM0640_1223_1257 | 0.01 | 2.46 | 1252160lmrdAlpenicillin-binding protein 2l- |
| STM0641_45_79 | 0.31 | 1.20 | 1252161lybeAlhypothetical proteinl- |
| STM0642_129_163 | 0.01 | 2.54 | 1252162lybeBlhypothetical proteinl- |
| STM0643_229_263 | 0.20 | 1.12 | 1252163lcobClalpha ribazole-5'-P phosphatasel- |
| STM0644_240_274 | 0.02 | -2.54 | 1252164lcobDlthreonine-phosphate decarboxylasel+ |
| STM0645_65_99 | 0.00 | -1.71 | 1252165lnadDlnicotinic acid mononucleotide adenyltransferasel- |
| STM0646_145_179 | 0.67 | -1.07 | 1252166lholAlDNA polymerase III subunit deltal- |
| STM0647_329_363 | 0.02 | -1.68 | 1252167lrlpBlLPS-assembly lipoprotein RplBl- |
| STM0648_1720_1754 | 0.47 | -1.17 | 1252168lleuSlleucyl-tRNA synthetasel- |
| STM0649.S_101_135 | 0.07 | 1.44 | 1252169lSTM0649.Slputative hydrolasel+ |
| STM0651_305_339 | 0.07 | 1.45 | 1252171lSTM0651l2-keto-3-deoxygluconate permeasel+ |
| STM0652_1442_1476 | 0.49 | -1.24 | 1252172lSTM0652lputative sigma-54 dependent transcriptional regulatorl+ |
| STM0653_5_39 | 0.01 | -1.84 | 1252173lybeLlhypothetical proteinl+ |
| STM0654_833_867 | 0.05 | 2.28 | 1252174lybeQltetratricopeptide repeat proteinl- |
| STM0655_289_323 | 0.20 | -1.17 | 1252175lybeRlputative cytoplasmic proteinl+ |
| STM0656_755_789 | 0.14 | -1.37 | 1252176lybeSlputative molecular chaperonel+ |
| STM0658_730_764 | 0.13 | -1.15 | 1252178lybeVlputative molecular chaperonel+ |
| STM0660_832_866 | 0.83 | 1.09 | 1252180lSTM0660lputative cytoplasmic proteinl- |
| STM0661_401_435 | 0.02 | -2.14 | 1252181lrihAlribonucleoside hydrolase 1l- |
| STM0662_513_547 | 0.02 | -1.99 | 1252182lgltLlglutamate/aspartate transporterl- |
| STM0663_321_355 | 0.01 | -1.59 | 1252183lgltKlglutamate/aspartate transporterl- |

| | | | |
|---|---|---|---|
| STM0664_641_675 | 0.01 | -1.35 | 1252184\|gltJ\|glutamate/aspartate transporter\|- |
| STM0665_289_323 | 0.60 | -1.06 | 1252185\|gltI\|glutamate and aspartate transporter subunit\|- |
| STM0666_1212_1246 | 0.00 | 1.27 | 1252186\|lnt\|apolipoprotein N-acyltransferase\|- |
| STM0667_129_163 | 0.04 | -1.11 | 1252187\|ybeX\|putative transport protein\|- |
| STM0668_401_435 | 0.32 | 1.18 | 1252188\|ybeY\|hypothetical protein\|- |
| STM0669_727_761 | 0.45 | -1.07 | 1252189\|phoL\|putative phosphate starvation-inducible protein\|- |
| STM0670_714_748 | 0.16 | 1.14 | 1252190\|miaB\|rRNA modification protein\|- |
| STM0671_705_739 | 0.33 | -1.16 | 1252191\|ubiF\|2-octaprenyl-3-methyl-6-methoxy-1 4-benzoquinol hydroxylase\|+ |
| STM0672_273_307 | 0.37 | 1.24 | 1252192\|STM0672\|putative inner membrane protein\|- |
| STM0675/STM0679_2_36 | 0.02 | -1.76 | 1252195/1252199\|metU/metT\|tRNA\|- |
| STM0678_9_43 | 0.01 | -2.65 | 1252198\|leuW\|tRNA\|- |
| STM0680_1114_1148 | 0.01 | 1.70 | 1252200\|asnB\|asparagine synthetase B\|- |
| STM0681_9_43 | 0.11 | -1.14 | 1252201\|nagD\|UMP phosphatase\|- |
| STM0682_862_896 | 0.05 | 1.63 | 1252202\|nagC\|N-acetylglucosamine operon transcriptional repressor\|- |
| STM0683_924_958 | 0.30 | -1.15 | 1252203\|nagA\|N-acetylglucosamine-6-phosphate deacetylase\|- |
| STM0684_689_723 | 0.07 | -1.09 | 1252204\|nagB\|glucosamine-6-phosphate deaminase\|- |
| STM0685_1498_1532 | 0.00 | -2.26 | 1252205\|nagE\|N-acetyl glucosamine specific PTS system components IIABC\|+ |
| STM0686_1245_1279 | 0.89 | -1.04 | 1252206\|glnS\|glutaminyl-tRNA synthetase\|+ |
| STM0687_728_762 | 0.03 | -1.65 | 1252207\|ybfM\|putative outer membrane protein\|+ |
| STM0689_498_532 | 0.31 | 1.20 | 1252209\|citA\|citrate-proton symporter\|- |
| STM0691_1197_1231 | 0.07 | 1.46 | 1252211\|STM0691\|tricarballylate dehydrogenase\|- |
| STM0692_297_331 | 0.57 | -1.05 | 1252212\|STM0692\|putative transcriptional regulator\|- |
| STM0693_257_291 | 0.28 | 1.14 | 1252213\|fur\|ferric uptake regulator\|- |
| STM0694_385_419 | 0.06 | 1.35 | 1252214\|fldA\|flavodoxin FldA\|- |
| STM0695_13_47 | 0.08 | -1.32 | 1252215\|ybfE\|LexA regulated protein\|- |
| STM0696_137_171 | 0.70 | -1.05 | 1252216\|ybfF\|hypothetical protein\|- |
| STM0697_257_291 | 0.22 | -1.17 | 1252217\|seqA\|replication initiation regulator SeqA\|+ |
| STM0698_642_676 | 0.02 | -2.02 | 1252218\|pgm\|phosphoglucomutase\|+ |
| STM0699_305_339 | 0.27 | -1.13 | 1252219\|STM0699\|putative cytoplasmic protein\|- |
| STM0700_897_931 | 0.23 | -1.16 | 1252220\|potE\|putrescine/ornithine antiporter\|- |
| STM0702_129_163 | 0.26 | 1.09 | 1252222\|kdpE\|DNA-binding response regulator in two-component regulatory system with KdpD\|- |
| STM0703_2262_2296 | 0.08 | -1.31 | 1252223\|kdpD\|sensor protein KdpD\|- |
| STM0704_227_261 | 0.06 | 1.18 | 1252224\|kdpC\|potassium-transporting ATPase subunit C\|- |
| STM0705_1642_1676 | 0.47 | 1.13 | 1252225\|kdpB\|potassium-transporting ATPase subunit B\|- |

| | | | |
|---|---|---|---|
| STM0707_48_86 | 0.01 | 1.93 | 1252227\|STM0707\|putative outer membrane protein\|- |
| STM0708_141_175 | 0.00 | 2.34 | 1252228\|ybfA\|putative periplasmic protein\|+ |
| STM0709_551_585 | 0.20 | -1.22 | 1252229\|phrB\|deoxyribodipyrimidine photolyase\|+ |
| STM0710_595_629 | 0.02 | 2.53 | 1252230\|ybgH\|putative POT family transport protein\|- |
| STM0711_169_203 | 0.13 | -1.24 | 1252231\|ybgI\|putative hydrolase-oxidase\|+ |
| STM0712_17_51 | 0.66 | -1.17 | 1252232\|ybgJ\|putative carboxylase\|+ |
| STM0713_593_627 | 0.19 | -1.26 | 1252233\|ybgK\|putative carboxylase\|+ |
| STM0714_385_419 | 0.13 | -1.23 | 1252234\|ybgL\|hypothetical protein\|+ |
| STM0715_33_67 | 0.02 | 1.68 | 1252235\|STM0715\|putative cytoplasmic protein\|+ |
| STM0716_493_527 | 0.03 | 2.82 | 1252236\|STM0716\|putative phage integrase\|+ |
| STM0717_301_335 | 0.83 | 1.02 | 1252237\|STM0717\|putative inner membrane protein\|+ |
| STM0718_513_547 | 0.41 | 1.04 | 1252238\|STM0718\|putative cytoplasmic protein\|+ |
| STM0719_602_636 | 0.02 | 2.31 | 1252239\|STM0719\|putative UDP-galactopyranose mutase\|+ |
| STM0721_775_809 | 0.51 | 1.19 | 1252241\|STM0721\|putative glycosyl transferase\|+ |
| STM0722_137_171 | 0.05 | 2.13 | 1252242\|STM0722\|putative ABC transporter permease protein\|+ |
| STM0723_161_195 | 0.20 | 1.69 | 1252243\|STM0723\|putative ABC-type polysaccharide/polyol phosphate transport system ATPase component\|+ |
| STM0725_33_67 | 0.04 | 2.91 | 1252245\|STM0725\|putative glycosyltransferase\|+ |
| STM0726_1673_1707 | 0.02 | 2.23 | 1252246\|STM0726\|putative glycosyl transferase\|+ |
| STM0727_100_135 | 0.04 | -1.89 | 1252247\|STM0727\|putative cytoplasmic protein\|+ |
| STM0728_545_579 | 0.94 | 1.01 | 1252248\|nei\|endonuclease VIII\|+ |
| STM0729_960_994 | 0.14 | 1.16 | 1252249\|abrB\|putative transport protein\|- |
| STM0730_349_383 | 0.00 | -4.30 | 1252250\|gltA\|type II citrate synthase\|- |
| STM0731_49_83 | 0.11 | -1.58 | 1252251\|STM0731\|putative inner membrane protein\|+ |
| STM0732_109_143 | 0.00 | -14.72 | 1252252\|sdhC\|succinate dehydrogenase cytochrome b556 large membrane subunit\|+ |
| STM0733_49_83 | 0.00 | -5.92 | 1252253\|sdhD\|succinate dehydrogenase cytochrome b556 small membrane subunit\|+ |
| STM0734_1664_1698 | 0.00 | -7.14 | 1252254\|sdhA\|succinate dehydrogenase flavoprotein subunit\|+ |
| STM0735_669_703 | 0.00 | -10.77 | 1252255\|sdhB\|succinate dehydrogenase iron-sulfur subunit\|+ |
| STM0736_1867_1901 | 0.00 | -8.11 | 1252256\|kgd\|alpha-ketoglutarate decarboxylase\|+ |
| STM0737_754_788 | 0.00 | -4.99 | 1252257\|sucB\|dihydrolipoamide acetyltransferase\|+ |
| STM0738_936_970 | 0.00 | -12.44 | 1252258\|sucC\|succinyl-CoA synthetase subunit beta\|+ |
| STM0739_65_99 | 0.00 | -8.47 | 1252259\|sucD\|succinyl-CoA synthetase subunit alpha\|+ |
| STM0740_586_620 | 0.87 | -1.03 | 1252260\|cydA\|cytochrome d terminal oxidase polypeptide subunit I\|+ |
| STM0741_701_735 | 0.34 | 1.14 | 1252261\|cydB\|cytochrome d terminal oxidase polypeptide subunit II\|+ |

| | | | |
|---|---|---|---|
| STM0742_9_43 | 0.97 | 1.01 | 1252262\|ybgT\|putative outer membrane lipoprotein\|+ |
| STM0743_9_43 | 0.07 | 1.30 | 1252263\|ybgE\|hypothetical protein\|+ |
| STM0744_361_395 | 0.01 | -1.40 | 1252264\|ybgC\|acyl-CoA thioester hydrolase YbgC\|+ |
| STM0745_169_203 | 0.04 | -2.03 | 1252265\|tolQ\|colicin uptake protein TolQ\|+ |
| STM0746_193_227 | 0.14 | -1.46 | 1252266\|tolR\|colicin uptake protein TolR\|+ |
| STM0747_1001_1035 | 0.89 | -1.02 | 1252267\|tolA\|cell envelope integrity inner membrane protein TolA\|+ |
| STM0748_806_840 | 0.01 | -1.80 | 1252268\|tolB\|translocation protein TolB\|+ |
| STM0749_129_163 | 0.02 | -2.58 | 1252269\|pal\|peptidoglycan-associated outer membrane lipoprotein\|+ |
| STM0750_193_227 | 0.15 | -1.30 | 1252270\|ybgF\|hypothetical protein\|+ |
| STM0756_397_431 | 0.05 | 1.57 | 1252276\|nadA\|quinolinate synthetase\|+ |
| STM0757_17_51 | 0.04 | 1.21 | 1252277\|pnuC\|nucleoside/purine/pyrimidine transporter\|+ |
| STM0758_833_867 | 0.33 | 1.17 | 1252278\|ybgR\|zinc transporter ZitB\|- |
| STM0759_225_259 | 0.04 | 2.05 | 1252279\|ybgS\|hypothetical protein\|- |
| STM0760_166_200 | 0.01 | -1.32 | 1252280\|aroG\|phospho-2-dehydro-3-heoxyheptonate aldolase\|+ |
| STM0761_353_387 | 0.17 | 1.13 | 1252281\|STM0761\|fumarate hydratase\|- |
| STM0762_577_611 | 0.65 | -1.07 | 1252282\|STM0762\|fumarate hydratase\|- |
| STM0763.s_769_803 | 0.13 | -1.27 | 1252283\|STM0763.s\|transcriptional regulator\|+ |
| STM0764_769_803 | 0.03 | 2.34 | 1252284\|STM0764\|transcriptional regulator\|- |
| STM0767_1309_1343 | 0.03 | 1.70 | 1252287\|dcoA\|pseudo\|+ |
| STM0769_65_99 | 0.37 | -1.20 | 1252289\|STM0769\|putative cytoplasmic protein\|+ |
| STM0770_793_827 | 0.08 | 1.23 | 1252290\|STM0770\|putative ABC transport protein\|+ |
| STM0772_385_419 | 0.59 | -1.09 | 1252292\|gpmA\|phosphoglyceromutase\|- |
| STM0773_698_732 | 0.87 | -1.03 | 1252293\|galM\|aldose 1-epimerase\|- |
| STM0774_918_952 | 0.59 | 1.02 | 1252294\|galK\|galactokinase\|- |
| STM0775_688_722 | 0.76 | 1.04 | 1252295\|galT\|galactose-1-phosphate uridylyltransferase\|- |
| STM0776_18_52 | 0.35 | -1.12 | 1252296\|galE\|UDP-galactose-4-epimerase\|- |
| STM0777_801_835 | 0.02 | 2.41 | 1252297\|STM0777\|putative inner membrane protein\|- |
| STM0778_757_791 | 0.14 | -1.26 | 1252298\|modF\|putative molybdenum transport ATP-binding protein ModF\|- |
| STM0779_113_147 | 0.54 | 1.09 | 1252299\|modE\|DNA-binding transcriptional dual regulator\|- |
| STM0780_21_55 | 0.11 | 1.72 | 1252300\|STM0780\|hypothetical protein\|+ |
| STM0781_545_579 | 0.02 | 1.90 | 1252301\|modA\|molybdate transporter periplasmic protein\|+ |
| STM0782_593_627 | 0.00 | -1.53 | 1252302\|modB\|molybdate ABC transporter permease protein\|+ |
| STM0783_180_214 | 0.38 | -1.10 | 1252303\|modC\|molybdate transporter ATP-binding protein\|+ |
| STM0784_321_355 | 0.21 | -1.25 | 1252304\|ybhA\|predicted hydrolase\|- |
| STM0785_449_483 | 0.45 | 1.15 | 1252305\|ybhE\|6-phosphogluconolactonase\|+ |
| STM0786_1181_1215 | 0.01 | -3.38 | 1252306\|ybhC\|putative pectinesterase\|- |

| | | | |
|---|---|---|---|
| STM0787_1137_1171 | 0.00 | -2.95 | 1252307\|hutI\|imidazolonepropionase\|+ |
| STM0788_785_819 | 0.01 | -1.72 | 1252308\|hutG\|formimidoylglutamase\|+ |
| STM0789_129_163 | 0.00 | -1.58 | 1252309\|hutC\|histidine utilization repressor\|+ |
| STM0790_798_832 | 0.01 | -3.32 | 1252310\|hutU\|pseudo\|+ |
| STM0792_345_379 | 0.02 | 2.44 | 1252312\|ybhB\|predicted kinase inhibitor\|- |
| STM0793_291_325 | 0.92 | -1.03 | 1252313\|bioA\|adenosylmethionine--8-amino-7-oxononanoate transaminase\|- |
| STM0794_490_524 | 0.14 | 1.60 | 1252314\|bioB\|biotin synthetase\|+ |
| STM0795_271_305 | 0.45 | -1.17 | 1252315\|bioF\|8-amino-7-oxononanoate synthase\|+ |
| STM0796_360_394 | 0.07 | 1.57 | 1252316\|bioC\|predicted methyltransferase\|+ |
| STM0797_73_107 | 0.13 | -1.72 | 1252317\|bioD\|dithiobiotin synthetase\|+ |
| STM0798_1343_1377 | 0.68 | -1.05 | 1252318\|uvrB\|excinuclease ABC subunit B\|+ |
| STM0799.1N_97_131 | 0.02 | 2.06 | 2673729\|STM0799.1N\|-\|+ |
| STM0800_2067_2101 | 0.03 | 1.43 | 1252320\|slrP\|leucine-rich repeat protein\|+ |
| STM0801_385_419 | 0.03 | -1.52 | 1252321\|ybhK\|putative cytoplasmic protein\|- |
| STM0802_81_115 | 0.03 | -1.43 | 1252322\|moaA\|molybdenum cofactor biosynthesis protein A\|+ |
| STM0803_65_99 | 0.30 | -1.11 | 1252323\|moaB\|molybdopterin biosynthetic protein B\|+ |
| STM0804_361_395 | 0.01 | -1.45 | 1252324\|moaC\|molybdenum cofactor biosynthesis protein C\|+ |
| STM0805_33_67 | 0.07 | -1.35 | 1252325\|moaD\|molybdopterin synthase small subunit\|+ |
| STM0806_97_131 | 0.24 | -1.16 | 1252326\|MoaE\|molybdopterin synthase large subunit\|+ |
| STM0807_257_291 | 0.26 | -1.11 | 1252327\|ybhL\|putative permease\|+ |
| STM0808_545_579 | 0.02 | 1.44 | 1252328\|ybhM\|putative integral membrane protein\|+ |
| STM0809_410_444 | 0.18 | 1.38 | 1252329\|STM0809\|putative inner membrane protein\|+ |
| STM0810_145_179 | 0.15 | 1.58 | 1252330\|STM0810\|putative inner membrane protein\|+ |
| STM0811_241_275 | 0.01 | 1.64 | 1252331\|ybhN\|hypothetical protein\|- |
| STM0812_1011_1045 | 0.01 | 1.65 | 1252332\|ybhO\|cardiolipin synthase 2\|- |
| STM0814_63_97 | 0.00 | -3.76 | 1252334\|ybhQ\|putative inner membrane protein\|+ |
| STM0815_236_270 | 0.01 | -1.51 | 1252335\|ybhR\|putative transport protein\|- |
| STM0816_388_422 | 0.02 | 1.40 | 1252336\|ybhS\|putative transport protein\|- |
| STM0817_1578_1612 | 0.64 | -1.06 | 1252337\|ybhF\|putative ABC-type multidrug transport system ATPase component\|- |
| STM0819_553_587 | 0.46 | -1.07 | 1252339\|ybiH\|putative DNA-binding transcriptional regulator\|- |
| STM0820_1134_1168 | 0.06 | -1.30 | 1252340\|rhlE\|ATP-dependent RNA helicase RhlE\|+ |
| STM0821_1514_1548 | 0.13 | 1.64 | 1252341\|dinG\|ATP-dependent DNA helicase DinG\|+ |
| STM0822_673_707 | 0.08 | 1.33 | 1252342\|ybiB\|hypothetical protein\|+ |
| STM0823_9_43 | 0.02 | 2.69 | 1252343\|ybiJ\|hypothetical protein\|- |
| STM0825_109_143 | 0.24 | 1.51 | 1252344\|ybiI\|hypothetical protein\|- |

| | | | |
|---|---|---|---|
| STM0826_385_419 | 0.65 | 1.05 | 1252345\|ybiN\|putative SAM-dependent methyltransferase\|+ |
| STM0827_1992_2026 | 0.33 | -1.14 | 1252346\|ybiO\|predicted mechanosensitive channel\|- |
| STM0828_65_99 | 0.04 | -1.21 | 1252347\|glnQ\|glutamine ABC transporter ATP-binding protein\|- |
| STM0829_257_291 | 0.04 | 1.13 | 1252348\|glnP\|glutamine ABC transporter permease protein\|- |
| STM0830_481_515 | 0.04 | 1.66 | 1252349\|glnH\|glutamine ABC transporter periplasmic protein\|- |
| STM0831_369_403 | 0.01 | 2.76 | 1252350\|dps\|DNA starvation/stationary phase protection protein Dps\|- |
| STM0832_561_595 | 0.40 | -1.10 | 1252351\|ybiF\|threonine and homoserine efflux system\|- |
| STM0833_393_427 | 0.01 | 5.22 | 1252352\|ompX\|outer membrane protein X\|+ |
| STM0835_97_131 | 0.04 | -1.25 | 1252354\|STM0835\|manganese transport regulator MntR\|+ |
| STM0836_114_148 | 0.37 | 1.10 | 1252355\|ybiR\|putative transporter\|+ |
| STM0838_1362_1396 | 0.01 | -1.96 | 1252357\|ybiT\|putative ABC transporter ATPase component\|+ |
| STM0841_667_701 | 0.04 | -1.38 | 1252360\|ybiU\|putative cytoplasmic protein\|- |
| STM0842_553_587 | 0.86 | 1.03 | 1252361\|ybiV(1)\|putative hydrolase\|+ |
| STM0844_449_483 | 0.05 | 2.06 | 1252363\|pflE\|putative pyruvate formate lyase activating enzyme\|- |
| STM0845_193_227 | 0.20 | 1.37 | 1252364\|moeB\|molybdopterin biosynthesis protein MoeB\|- |
| STM0846_931_965 | 0.71 | 1.06 | 1252365\|moeA\|molybdopterin biosynthesis protein MoeA\|- |
| STM0847_577_611 | 0.12 | -1.66 | 1252366\|ybiK\|L-asparaginase\|+ |
| STM0848_1333_1367 | 0.02 | -1.75 | 1252367\|yliA\|glutathione transporter ATP-binding protein\|+ |
| STM0849_828_862 | 0.05 | -1.60 | 1252368\|yliB\|putative ABC transporter periplasmic binding protein\|+ |
| STM0850_677_711 | 0.24 | -1.24 | 1252369\|yliC\|putative ABC transporter periplasmic binding protein\|+ |
| STM0851_465_499 | 0.02 | -2.26 | 1252370\|yliD\|putative ABC transporter inner membrane component\|+ |
| STM0852_1223_1257 | 0.03 | -2.04 | 1252371\|yliG\|putative FeS oxidoreductase\|- |
| STM0853_273_307 | 0.02 | 1.45 | 1252372\|bssR\|biofilm formation regulatory protein BssR\|+ |
| STM0854_221_255 | 0.02 | 1.51 | 1252373\|STM0854\|putative cytoplasmic protein\|+ |
| STM0855_787_821 | 0.10 | 2.21 | 1252374\|STM0855\|putative electron transfer protein beta subunit\|+ |
| STM0856_569_603 | 0.26 | 1.14 | 1252375\|STM0856\|putative electron transfer protein alpha subunit\|+ |
| STM0857_949_983 | 0.89 | 1.01 | 1252376\|STM0857\|putative acyl-CoA dehydrogenase\|+ |
| STM0858_991_1025 | 0.15 | 1.50 | 1252377\|STM0858\|putative dehydrogenase\|+ |
| STM0859_481_515 | 0.05 | 2.08 | 1252378\|STM0859\|putative transcriptional regulator\|- |
| STM0860_285_321 | 0.01 | 3.93 | 1252379\|STM0860\|putative inner membrane protein\|- |
| STM0861_593_627 | 0.32 | 1.23 | 1252380\|yliI\|-\|+ |
| STM0862_90_124 | 0.69 | 1.03 | 1252381\|yliJ\|putative glutathione S-transferase\|- |
| STM0863_556_590 | 0.03 | 1.80 | 1252382\|dacC\|D-alanyl-D-alanine carboxypeptidase fraction C\|+ |
| STM0864_345_379 | 0.77 | 1.08 | 1252383\|deoR\|DNA-binding transcriptional repressor\|- |
| STM0865_49_83 | 0.11 | 1.42 | 1252384\|ybjG\|undecaprenyl pyrophosphate phosphatase\|- |
| STM0866_810_844 | 0.41 | -1.12 | 1252385\|mdfA\|multidrug translocase\|+ |

| | | | |
|---|---|---|---|
| STM0867_145_179 | 0.00 | 1.51 | 1252386lSTM0867lputative hydrolasel- |
| STM0868_213_247 | 0.07 | -1.18 | 1252387lSTM0868lputative transport protein/regulatorl- |
| STM0869_129_163 | 0.49 | 1.11 | 1252388lSTM0869lputative regulatory proteinl+ |
| STM0870_879_913 | 0.03 | -1.10 | 1252389lSTM0870lhypothetical proteinl- |
| STM0871_225_259 | 0.04 | 1.61 | 1252390lybjMlputative inner membrane proteinl+ |
| STM0872_73_107 | 0.02 | 1.63 | 1252391lgrxAlglutaredoxin 1l- |
| STM0873_21_55 | 0.78 | -1.04 | 1252392lybjClpredicted inner membrane proteinl+ |
| STM0874_577_611 | 0.57 | 1.06 | 1252393lmdaAlnitroreductase Al+ |
| STM0876_321_355 | 0.27 | -1.11 | 1252395lybjNlputative cytoplasmic proteinl+ |
| STM0877_626_660 | 0.00 | -2.43 | 1252396lpotFlputrescine transporter subunit: periplasmic-binding component of ABC superfamilyl+ |
| STM0878_1039_1073 | 0.00 | -2.36 | 1252397lpotGlputrescine transporter ATP-binding subunitl+ |
| STM0879_209_243 | 0.20 | -1.16 | 1252398lpotHlputrescine transporter subunit: membrane component of ABC superfamilyl+ |
| STM0880_753_787 | 0.01 | -1.84 | 1252399lpotIlputrescine transporter subunit: membrane component of ABC superfamilyl+ |
| STM0881_353_387 | 0.09 | 1.20 | 1252400lybjOlputative inner membrane proteinl+ |
| STM0882_756_790 | 0.03 | -1.33 | 1252401lrumBl23S rRNA metyluridine methyltransferasel+ |
| STM0884_761_795 | 0.78 | -1.04 | 1252402lulaAlascorbate-specific PTS system enzyme IICl+ |
| STM0885_185_219 | 0.03 | 1.39 | 1252403lSTM0885lputative inner membrane proteinl+ |
| STM0886_745_779 | 0.04 | 1.37 | 1252404lSTM0886lputative sulfatasel+ |
| STM0887_129_163 | 0.01 | 1.46 | 1252405lartJlarginine transport system componentl- |
| STM0888_153_187 | 0.01 | -2.06 | 1252406lartMlarginine transporter permease subunit ArtMl- |
| STM0889_609_643 | 0.01 | -1.61 | 1252407lartQlarginine transporter permease subunit ArtQl- |
| STM0890_433_467 | 0.00 | -3.46 | 1252408lartIlarginine transport systeml- |
| STM0891_513_547 | 0.01 | -1.77 | 1252409lartPlarginine transporter ATP-binding subunitl- |
| STM0892_401_435 | 0.99 | 1.00 | 1252410lybjPlpredicted lipoproteinl- |
| STM0894_109_143 | 0.02 | -2.04 | 1252412lSTM0894lputative excisionasel- |
| STM0897_349_383 | 0.04 | -1.33 | 1252415lSTM0897lhypothetical proteinl- |
| STM0900_1129_1163 | 0.05 | -2.03 | 1252419lSTM0900lputative helicasel+ |
| STM0901_865_899 | 0.37 | -1.18 | 1252420lSTM0901lputative phage DNA primasel+ |
| STM0906_73_107 | 0.05 | 2.10 | 1252425lSTM0906lhypothetical proteinl+ |
| STM0907_73_107 | 0.25 | 1.33 | 1252426lSTM0907lputative chitinasel+ |
| STM0908_53_88 | 0.09 | 1.61 | 1252427lSTM0908lhypothetical proteinl+ |
| STM0909_321_355 | 0.14 | -1.52 | 1252428lSTM0909lhypothetical proteinl+ |
| STM0910_1302_1336 | 0.15 | -1.27 | 1252429lSTM0910lhypothetical proteinl+ |

| | | | |
|---|---|---|---|
| STM0912_1908_1942 | 0.59 | -1.18 | 1252431lSTM0912lATP-dependent proteasel+ |
| STM0913.2N_198_232 | 0.35 | -1.23 | 2673767lSTM0913.2Nlhypothetical proteinl+ |
| STM0914_429_463 | 0.90 | -1.02 | 1252433lSTM0914lputative phage tail componentl+ |
| STM0915_296_331 | 0.02 | -1.33 | 1252434lSTM0915lhypothetical proteinl+ |
| STM0916.1N_365_399 | 0.50 | -1.05 | 2673747lSTM0916.1Nlhypothetical proteinl+ |
| STM0921_249_283 | 0.45 | -1.09 | 1252440lSTM0921lputative minor tail proteinl+ |
| STM0922_533_567 | 0.03 | -1.60 | 1252441lSTM0922lputative phage tail assembly proteinl+ |
| STM0928_608_642 | 0.11 | -1.57 | 1252447lnanHlneuraminidasel- |
| STM0930_33_67 | 0.15 | 1.14 | 1252449lorfBlhypothetical proteinl+ |
| STM0931_321_355 | 0.34 | -1.06 | 1252450lybjRlputative aminidasel+ |
| STM0932_399_433 | 0.01 | -1.45 | 1252451lSTM0932lputative nucleoside-diphosphate-sugar epimerasel- |
| STM0933_467_501 | 0.09 | 1.16 | 1252452lybjTlputative nucleoside-diphosphate-sugar epimerasel- |
| STM0934_3_37 | 0.03 | 1.51 | 1252453lltaAlL-allo-threonine aldolase  PLP-dependentl- |
| STM0935_1024_1058 | 0.09 | 1.41 | 1252454lpoxBlpyruvate dehydrogenasel- |
| STM0936_545_579 | 0.02 | -1.66 | 1252455lhcrlHCP oxidoreductase  NADH-dependentl- |
| STM0938_769_803 | 0.03 | -1.18 | 1252457lybjElputative inner membrane proteinl- |
| STM0939_916_950 | 0.01 | -1.55 | 1252458lybjDlhypothetical proteinl+ |
| STM0940_193_227 | 0.30 | 1.16 | 1252459lybjXlVirK-like proteinl- |
| STM0941_1016_1050 | 0.02 | 1.43 | 1252460lybjYlmacrolide transporter subunit MacAl+ |
| STM0942_1428_1462 | 0.04 | 1.61 | 1252461lybjZlmacrolide transporter ATP-binding /permease proteinl+ |
| STM0943_105_139 | 0.00 | -6.01 | 1252462lcspDlstress response proteinl- |
| STM0944_248_282 | 0.08 | -1.47 | 1252463lclpSlATP-dependent Clp protease adaptor protein ClpSl+ |
| STM0945_1406_1440 | 0.11 | -1.45 | 1252464lclpAlATP-dependent Clp protease ATP-binding subunitl+ |
| STM0946/STM2471/STM303 | 0.07 | -1.17 | 1252465/1253993/1254555/1255002/1255837ltnpA_1/tnpA_3/tnpA_4/ tnpA_5/tnpA_6ltransposasel+ |
| STM0947_193_227 | 0.27 | 1.24 | 1252466lSTM0947lputative integrasel- |
| STM0948_129_163 | 0.08 | -1.57 | 1252467lSTM0948lputative cytoplasmic proteinl- |
| STM0949_8_42 | 0.71 | 1.49 | 1252468lserWltRNAl- |
| STM0950_401_435 | 0.06 | -1.72 | 1252469lSTM0950lSlsAl- |
| STM0952_833_867 | 0.00 | 1.67 | 1252471lSTM0952lputative transcriptional regulatorl+ |
| STM0953_65_99 | 0.32 | 1.10 | 1252472linfAltranslation initiation factor IF-1l- |
| STM0954_49_83 | 0.73 | -1.10 | 1252473lSTM0954lputative inner membrane proteinl- |
| STM0955_353_387 | 0.61 | -1.04 | 1252474laatlleucyl/phenylalanyl-tRNA--protein transferasel- |
| STM0956_1379_1413 | 0.02 | -1.43 | 1252475lcydClcysteine/glutathione ABC transporter membrane/ATP-binding componentl- |

| | | | |
|---|---|---|---|
| STM0957_1632_1666 | 0.80 | 1.04 | 1252476lcydDlcysteine/glutathione ABC transporter membrane/ATP-binding componentl- |
| STM0958_705_739 | 0.88 | 1.03 | 1252477ltrxBlthioredoxin reductasel- |
| STM0959_353_387 | 0.02 | -1.90 | 1252478llrplleucine-responsive transcriptional regulatorl+ |
| STM0960_3841_3875 | 0.02 | -2.04 | 1252479lftsKlDNA translocase FtsKl+ |
| STM0961_321_355 | 0.05 | -1.31 | 1252480llolAlouter-membrane lipoprotein carrier proteinl+ |
| STM0962_1185_1219 | 0.01 | -1.32 | 1252481lycaJlrecombination factor protein RarAl+ |
| STM0963_550_584 | 0.16 | 1.37 | 1252482lserSlseryl-tRNA synthetasel+ |
| STM0964_2342_2376 | 0.99 | -1.00 | 1252483ldmsAlanaerobic dimethyl sulfoxide reductase subunit Al+ |
| STM0965_583_617 | 0.86 | -1.01 | 1252484ldmsBlanaerobic dimethyl sulfoxide reductase subunit Bl+ |
| STM0966_417_451 | 0.00 | 2.35 | 1252485ldmsClanaerobic dimethyl sulfoxide reductase subunit Cl+ |
| STM0969_1127_1161 | 0.02 | 1.14 | 1252487lycaMlputative amino-acid transporterl+ |
| STM0970_161_195 | 0.01 | 2.26 | 1252488lpflAlpyruvate formate lyase-activating enzyme 1l- |
| STM0972_721_760 | 0.02 | -1.40 | 1252490lSTM0972lSopD-like proteinl+ |
| STM0973_2244_2278 | 0.83 | -1.03 | 1252491lpflBlpyruvate formate lyase Il- |
| STM0974_497_531 | 0.04 | -1.48 | 1252492lfocAlformate transporterl- |
| STM0975_1482_1516 | 0.06 | -1.35 | 1252493lycaOlputative cytoplasmic proteinl- |
| STM0976_317_351 | 0.17 | -1.22 | 1252494lycaPlputative inner membrane proteinl+ |
| STM0977_442_476 | 0.20 | 1.30 | 1252495lserClphosphoserine aminotransferasel+ |
| STM0978_1053_1087 | 0.68 | -1.13 | 1252496laroAl3-phosphoshikimate 1-carboxyvinyltransferasel+ |
| STM0979_641_675 | 0.01 | 1.56 | 1252497lycaLlputative Zn-dependent proteasel+ |
| STM0980_137_171 | 0.03 | -1.80 | 1252498lcmklcytidylate kinasel+ |
| STM0981_851_885 | 0.02 | -1.79 | 1252499lrpsAl30S ribosomal protein S1l+ |
| STM0982_232_266 | 0.56 | 1.11 | 1252500lihfBlintegration host factor subunit betal+ |
| STM0984_1134_1168 | 0.02 | -1.81 | 1252502lmsbAllipid transporter ATP-binding/permease proteinl+ |
| STM0985_417_451 | 0.04 | -1.62 | 1252503llpxKltetraacyldisaccharide 4'-kinasel+ |
| STM0986_898_932 | 0.03 | -1.28 | 1252504lycaQlputative cytoplasmic proteinl+ |
| STM0987_83_117 | 0.24 | 1.21 | 1252505lycaRlhypothetical proteinl+ |
| STM0988_277_311 | 0.03 | 1.54 | 1252506lkdsBl3-deoxy-manno-octulosonate cytidylyltransferasel+ |
| STM0989_561_595 | 0.22 | -1.13 | 1252507lSTM0989lhypothetical proteinl+ |
| STM0990_132_167 | 0.02 | -1.81 | 1252508lycbClhypothetical proteinl- |
| STM0991_201_235 | 0.03 | -1.48 | 1252509lsmtAlputative metallothionein SmtAl+ |
| STM0992_372_406 | 0.03 | -1.51 | 1252510lmukFlcondesin subunit Fl+ |
| STM0993_321_355 | 0.01 | -1.96 | 1252511lmukElcondesin subunit El+ |
| STM0994_3804_3838 | 0.00 | -3.30 | 1252512lmukBlcell division protein MukBl+ |
| STM0995_1099_1133 | 0.00 | 1.91 | 1252513lycbBlhypothetical proteinl+ |

| | | | |
|---|---|---|---|
| STM0996_109_143 | 0.04 | -1.66 | 1252514lycbKlputative outer membrane proteinl+ |
| STM0997_417_451 | 0.81 | -1.05 | 1252515lycbLlputative metallo-beta-lactamasel+ |
| STM0998_944_978 | 0.75 | -1.08 | 1252516laspClaromatic amino acid aminotransferasel- |
| STM0999_285_319 | 0.01 | -2.61 | 1252517lompFlouter membrane protein F precursorl- |
| STM1000_1338_1372 | 0.02 | 1.82 | 1252518lasnClasparaginyl-tRNA synthetasel- |
| STM1001_281_315 | 0.00 | -1.87 | 1252519lSTM1001lputative leucine response regulatorl- |
| STM1003_694_728 | 0.02 | -1.97 | 1252521lSTM1003lputative transcriptional regulatorl+ |
| STM1004_476_510 | 0.06 | -1.11 | 1252522lpncBlnicotinate phosphoribosyltransferasel- |
| STM1005_1062_1096 | 0.12 | 1.44 | 1252523lSTM1005lintegrasel- |
| STM1006_145_179 | 0.10 | 1.91 | 1252524lSTM1006lexcisionasel- |
| STM1009/STM2632_2759_2 | 0.00 | 1.91 | 1252527/1254155lSTM1009/STM2632lexodeoxyribonucleasel- |
| STM1010_127_161 | 0.10 | 1.35 | 1252528lSTM1010lhypothetical proteinl- |
| STM1010.1n_122_156 | 0.25 | 1.37 | 2673727lSTM1010.1nlhypothetical proteinl- |
| STM1011_144_178 | 0.01 | 1.95 | 1252529lSTM1011lhypothetical proteinl- |
| STM1012/STM2628_67_101 | 0.12 | 1.37 | 1252530/1254151lSTM1012/STM2628lprobable regulatory proteinl- |
| STM1013/STM2627_57_91 | 0.04 | 1.84 | 1252531/1254150lSTM1013/STM2627lprobable regulatory proteinl+ |
| STM1014/STM2626_257_29 | 0.03 | -1.24 | 1252532/1254149lSTM1014/STM2626lprobable regulatory proteinl+ |
| STM1015/STM2625_577_61 | 0.50 | 1.05 | 1252533/1254148lSTM1015/STM2625lputative replication proteinl+ |
| STM1016/STM2624_193_22 | 0.01 | -1.47 | 1252534/1254147lSTM1016/STM2624lhypothetical proteinl+ |
| STM1017/STM2623_129_16 | 0.03 | -1.29 | 1252535/1254146lSTM1017/STM2623lhypothetical proteinl+ |
| STM1018_60_99 | 0.02 | 1.44 | 1252536lSTM1018lhypothetical proteinl+ |
| STM1020_159_194 | 0.02 | 1.54 | 1252538lSTM1020lhypothetical proteinl+ |
| STM1021_432_466 | 0.01 | 2.68 | 1252539lSTM1021lhypothetical proteinl+ |
| STM1021.1n_77_111 | 0.02 | 1.19 | 2673724lSTM1021.1nlhypothetical proteinl+ |
| STM1022_673_707 | 0.96 | -1.00 | 1252540lSTM1022lputative molecular chaperonel+ |
| STM1023_225_259 | 0.16 | -1.32 | 1252541lSTM1023lhypothetical proteinl+ |
| STM1024_5_39 | 0.18 | 1.49 | 1252542lSTM1024lhypothetical proteinl+ |
| STM1025_385_419 | 0.04 | 2.05 | 1252543lSTM1025lhypothetical proteinl- |
| STM1026_166_200 | 0.09 | 1.35 | 1252544lSTM1026lhypothetical proteinl- |
| STM1027_80_114 | 0.06 | 1.96 | 1252545lSTM1027lhypothetical proteinl+ |
| STM1028_141_175 | 0.05 | 1.20 | 1252546lSTM1028llysozymel+ |
| STM1029_83_117 | 0.07 | 1.61 | 1252547lSTM1029lhypothetical proteinl+ |
| STM1030_145_179 | 0.87 | -1.02 | 1252548lSTM1030lhypothetical proteinl+ |
| STM1031_1332_1366 | 0.01 | -1.41 | 1252549lSTM1031lhypothetical proteinl+ |
| STM1032_603_637 | 0.01 | -1.64 | 1252550lSTM1032lhypothetical proteinl+ |
| STM1033_1083_1117 | 0.43 | -1.08 | 1252551lSTM1033lClp protease-like proteinl+ |

| | | | |
|---|---|---|---|
| STM1034_49_83 | 0.04 | 1.77 | 1252552lSTM1034lputative RecA/RadA recombinasel+ |
| STM1035_33_67 | 0.11 | 1.61 | 1252553lSTM1035lATP-binding sugar transporter-like proteinl+ |
| STM1036_249_283 | 0.74 | -1.07 | 1252554lSTM1036lprobable minor tail proteinl+ |
| STM1037_366_400 | 0.02 | -1.11 | 1252555lSTM1037lprobable minor tail proteinl+ |
| STM1038_9_43 | 0.08 | -1.20 | 1252556lSTM1038lprobable major tail proteinl+ |
| STM1039_49_83 | 0.48 | 1.13 | 1252557lSTM1039lprobable minor tail proteinl+ |
| STM1040_229_263 | 0.03 | -1.72 | 1252558lSTM1040lprobable minor tail proteinl+ |
| STM1041_2677_2711 | 0.17 | -1.20 | 1252559lSTM1041lprobable minor tail proteinl+ |
| STM1042_226_260 | 0.03 | 1.67 | 1252560lSTM1042lprobable minor tail proteinl+ |
| STM1043_191_225 | 0.65 | 1.04 | 1252561lSTM1043lattachment/invasion proteinl+ |
| STM1044_429_463 | 0.00 | 3.26 | 1252562lsodClsuperoxide dismutase precursorl- |
| STM1045_301_335 | 0.04 | -1.39 | 1252563lSTM1045lprobable minor tail proteinl+ |
| STM1046_225_259 | 0.02 | -1.44 | 1252564lSTM1046lprobable tail assembly proteinl+ |
| STM1047_341_375 | 0.10 | -1.15 | 1252565lSTM1047lprobable tail assembly proteinl+ |
| STM1048_2217_2251 | 0.18 | 1.25 | 1252566lSTM1048lhost specificity protein Jl+ |
| STM1048.1N_717_751 | 0.66 | 1.08 | 2673720lSTM1048.1Nlhypothetical proteinl+ |
| STM1049_1520_1554 | 0.20 | 1.13 | 1252567lSTM1049lprobable tail fiber proteinl+ |
| STM1050_329_364 | 0.03 | 1.82 | 1252568lSTM1050ltail fiber assembly like-proteinl+ |
| STM1051_433_467 | 0.00 | 3.37 | 1252569lssellsecreted effector proteinl+ |
| STM1052_121_155 | 0.02 | 3.47 | 1252570lSTM1052lpseudol+ |
| STM1053_129_163 | 0.04 | 1.28 | 1252571lSTM1053lhypothetical proteinl- |
| STM1054_228_263 | 0.02 | 1.64 | 1252572lSTM1054lhypothetical proteinl- |
| STM1055_105_139 | 0.07 | 1.39 | 1252573lSTM1055lhypothetical proteinl- |
| STM1056_57_91 | 0.01 | 1.34 | 1252574lSTM1056lMsgA-like proteinl- |
| STM1057_2446_2480 | 0.13 | 1.26 | 1252575lpepNlaminopeptidase Nl+ |
| STM1058_68_102 | 0.06 | -1.28 | 1252576lpyrDldihydroorotate dehydrogenase 2l+ |
| STM1059_345_379 | 0.91 | -1.02 | 1252577lycbWlputative cytoplasmic proteinl+ |
| STM1060_895_929 | 0.01 | 1.38 | 1252578lSTM1060lputative iron-sulfur proteinl- |
| STM1061_1366_1400 | 0.07 | 1.50 | 1252579lycbYlputative N6-adenine-specific DNA methylasel+ |
| STM1062_1229_1263 | 0.03 | -1.51 | 1252580luuplABC transporter ATPase componentl+ |
| STM1063_1183_1217 | 0.08 | 1.90 | 1252581lpqiAlparaquat-inducible protein Al+ |
| STM1064_1522_1556 | 0.04 | 2.65 | 1252582lpqiBlparaquat-inducible protein Bl+ |
| STM1065_73_107 | 0.03 | -1.18 | 1252583lymbAlputative outer membrane proteinl+ |
| STM1066_81_115 | 0.20 | 1.30 | 1252584lrmflribosome modulation factorl+ |
| STM1067_449_483 | 0.01 | -2.29 | 1252585lfabAl3-hydroxydecanoyl-(acyl carrier protein) dehydratasel- |
| STM1068_1594_1628 | 0.02 | -1.17 | 1252586llonHlputative proteasel- |

| | | | |
|---|---|---|---|
| STM1069_281_315 | 0.10 | 1.32 | 1252587lycbGlhypothetical proteinl+ |
| STM1071_401_435 | 0.22 | 1.28 | 1252589lsulAlSOS cell division inhibitorl- |
| STM1072_113_147 | 0.00 | -2.26 | 1252590lyccRlputative DNA transformation proteinl+ |
| STM1073_1427_1461 | 0.92 | -1.01 | 1252591lyccSlputative transporterl- |
| STM1074_289_323 | 0.13 | 1.45 | 1252592lyccFlhypothetical proteinl- |
| STM1075_1280_1314 | 0.12 | 1.46 | 1252593lhelDlDNA helicase IVl+ |
| STM1076_321_355 | 0.02 | -1.70 | 1252594lmgsAlmethylglyoxal synthasel- |
| STM1077_497_531 | 0.98 | 1.00 | 1252595lyccTlhypothetical proteinl- |
| STM1078_119_153 | 0.84 | 1.03 | 1252596lSTM1078lputative cytoplasmic proteinl+ |
| STM1079_81_115 | 0.16 | 1.24 | 1252597lyccVlputative inner membrane proteinl- |
| STM1080_853_887 | 0.28 | 1.25 | 1252598lyccWlputative SAM-dependent methyltransferasel- |
| STM1081_257_291 | 0.08 | 1.83 | 1252599lSTM1081lputative outer membrane proteinl+ |
| STM1082_457_491 | 0.89 | -1.02 | 1252600lSTM1082lbacterial regulatory proteinl+ |
| STM1083_241_275 | 0.14 | 1.11 | 1252601lyccXlputative phosphohydrolasel+ |
| STM1084_235_269 | 0.29 | -1.15 | 1252602lyccKlsulfurtransferase TusEl- |
| STM1085_561_595 | 0.00 | 8.43 | 1252603lyccAlhypothetical proteinl- |
| STM1086_35_69 | 0.75 | 1.49 | 1252604lserTltRNAl+ |
| STM1087_5_39 | 0.07 | 1.33 | 1252605lpipAlpathogenicity island-encoded protein Al- |
| STM1089_259_293 | 0.08 | 1.19 | 1252607lSTM1089lputative inner membrane proteinl+ |
| STM1090_129_163 | 0.02 | -1.46 | 1252608lpipClpathogenicity island-encoded protein Cl- |
| STM1092_125_159 | 0.05 | -1.76 | 1252610lorfXlputative cytoplasmic proteinl+ |
| STM1093_37_71 | 0.00 | -7.71 | 1252611lSTM1093lputative cytoplasmic proteinl- |
| STM1095_1262_1296 | 0.23 | 1.21 | 1252613lcopSlcopper resistance proteinl- |
| STM1096_357_391 | 0.68 | 1.11 | 1252614lcopRltranscriptional regulatory protein YedWl- |
| STM1097_157_191 | 0.02 | 1.76 | 1252615lSTM1097lhypothetical proteinl+ |
| STM1098_353_387 | 0.13 | 1.23 | 1252616lhpaCl4-hydroxyphenylacetate catabolisml- |
| STM1100_225_259 | 0.02 | 1.85 | 1252618lhpaRl4-hydroxyphenylacetate catabolisml- |
| STM1106_209_243 | 0.03 | -1.61 | 1252624lhpaIl4-hydroxyphenylacetate catabolisml+ |
| STM1107_962_996 | 0.02 | -1.91 | 1252625lhpaXl4-hydroxyphenylacetate catabolisml+ |
| STM1108_769_803 | 0.02 | 1.98 | 1252626lhpaAl4-hydroxyphenylacetate catabolisml+ |
| STM1109_577_611 | 0.96 | -1.00 | 1252627lSTM1109lputative periplasmic proteinl+ |
| STM1110_33_67 | 0.09 | 2.63 | 1252628lSTM1110lhypothetical proteinl- |
| STM1111_97_131 | 0.05 | -1.46 | 1252629lyccDlchaperone-modulator protein CbpMl- |
| STM1112_193_227 | 0.14 | -1.16 | 1252630lcbpAlcurved DNA-binding protein CbpAl- |
| STM1113_41_76 | 0.60 | 1.07 | 1252631lscsAlsuppression of copper sensitivity protein Al+ |
| STM1114_1016_1050 | 0.74 | 1.12 | 1252632lscsBlsuppression of copper sensitivity proteinl+ |

| | | | |
|---|---|---|---|
| STM1116_53_87 | 0.06 | -1.18 | 1252634lscsDlsuppression of copper sensitivity proteinl+ |
| STM1117_307_341 | 0.00 | -7.81 | 1252635lagplglucose-1-phosphatase/inositol phosphatasel+ |
| STM1118_129_163 | 0.41 | 1.13 | 1252636lyccJlhypothetical proteinl- |
| STM1119_81_115 | 0.28 | -1.15 | 1252637lwraBlTrpR binding protein WrbAl- |
| STM1120_19_53 | 0.08 | 1.53 | 1252638lycdFlpseudol+ |
| STM1121_95_129 | 0.02 | 3.35 | 1252639lymdFlputative cytoplasmic proteinl+ |
| STM1122_481_515 | 0.10 | -1.46 | 1252640lycdClputative transcriptional repressorl+ |
| STM1123_33_67 | 0.01 | -2.86 | 1252641lSTM1123lputative periplasmic proteinl- |
| STM1124_3228_3262 | 0.05 | -2.49 | 2673752lputAl-l- |
| STM1125_798_832 | 0.01 | -1.89 | 1252643lputPlmajor sodium/proline symporterl+ |
| STM1126_449_483 | 0.01 | -1.43 | 2673763lphoHl-l+ |
| STM1127_129_163 | 0.00 | 1.88 | 1252645lSTM1127lputative transcriptional regulatorl- |
| STM1128_1298_1332 | 0.45 | -1.10 | 1252646lSTM1128lputative sodium/glucose cotransporterl- |
| STM1129_33_67 | 0.08 | -1.18 | 1252647lSTM1129lN-acetylmannosamine-6-phosphate 2-epimerasel- |
| STM1130_706_740 | 0.03 | 1.99 | 1252648lSTM1130lputative inner membrane proteinl+ |
| STM1131_57_91 | 0.66 | -1.10 | 1252649lSTM1131lputative outer membrane proteinl+ |
| STM1132_482_516 | 0.89 | 1.02 | 1252650lSTM1132lputative sialic acid transporterl+ |
| STM1133_108_142 | 0.18 | 1.18 | 1252651lSTM1133lputative dehydrogenasel+ |
| STM1134_12_46 | 0.73 | 1.44 | 1252652lserXltRNAl- |
| STM1135_769_803 | 0.01 | 1.74 | 1252653lycdWlputative oxidoreductasel+ |
| STM1136_33_67 | 0.46 | -1.07 | 1252654lycdXlputative hydrolasel+ |
| STM1137_81_115 | 0.51 | -1.08 | 1252655lycdYlputative oxidoreductase componentl+ |
| STM1138_189_223 | 0.02 | 1.84 | 1252656lycdZlputative inner membrane proteinl+ |
| STM1139_353_387 | 0.56 | 1.03 | 1252657lcsgGlputative curli operon transcriptional regulatorl- |
| STM1141_94_128 | 0.01 | -1.45 | 1252659lcsgElcurli assembly protein CsgEl- |
| STM1142_345_379 | 0.04 | -1.38 | 1252660lcsgDlDNA-binding transcriptional activator in two-component regulatory systeml- |
| STM1143_129_163 | 0.13 | -1.31 | 1252661lcsgBlcurlin minor subunitl+ |
| STM1144_209_243 | 0.01 | 2.41 | 1252662lcsgAlcryptic curlin major subunitl+ |
| STM1145_81_115 | 0.03 | 3.41 | 1252663lcsgClputative curli production protein precursorl+ |
| STM1146_241_275 | 0.03 | 2.47 | 1252664lymdAlputative periplasmic proteinl+ |
| STM1147_209_243 | 0.02 | 1.60 | 1252665lSTM1147lhypothetical proteinl+ |
| STM1148.S_1129_1163 | 0.35 | 1.10 | 1252666lymdClputative phospholipasel+ |
| STM1149_808_842 | 0.67 | -1.05 | 1252667lmdoClglucans biosynthesis proteinl- |
| STM1150_793_827 | 0.17 | -1.21 | 1252668lmdoGlglucan biosynthesis protein Gl+ |
| STM1151_2089_2123 | 0.05 | -1.18 | 1252669lmdoHlglucosyltransferase MdoHl+ |

| | | | |
|---|---|---|---|
| STM1152_129_163 | 0.19 | -1.29 | 1252670\|yceK\|predicted lipoprotein\|+ |
| STM1153_65_99 | 0.01 | -2.02 | 1252671\|msyB\|hypothetical protein\|- |
| STM1154_760_794 | 0.76 | 1.04 | 1252672\|yceE\|drug efflux system protein MdtG\|- |
| STM1155_457_491 | 0.31 | 1.07 | 1252673\|htrB\|lipid A biosynthesis lauroyl acyltransferase\|- |
| STM1156_758_792 | 0.03 | -2.18 | 1252674\|yceA\|hypothetical protein\|+ |
| STM1157_353_387 | 0.33 | -1.11 | 1252675\|yceI\|hypothetical protein\|- |
| STM1158_97_131 | 0.09 | 1.21 | 1252676\|STM1158\|putative inner membrane protein\|- |
| STM1159_61_95 | 0.04 | 1.46 | 1252677\|yceO\|putative inner membrane protein\|- |
| STM1160_504_538 | 0.87 | 1.04 | 1252678\|solA\|N-methyltryptophan oxidase\|- |
| STM1161.S_53_87 | 0.85 | 1.01 | 1252679\|bssS\|biofilm formation regulatory protein BssS\|- |
| STM1162_7_41 | 0.54 | 1.05 | 1252680\|dinI\|DNA damage-inducible protein I\|- |
| STM1163_208_242 | 0.02 | -1.66 | 1252681\|pyrC\|dihydroorotase\|- |
| STM1164_37_71 | 0.04 | 1.52 | 1252682\|yceB\|predicted lipoprotein\|- |
| STM1165_457_491 | 0.54 | -1.15 | 1252683\|grxB\|glutaredoxin 2\|- |
| STM1166_690_724 | 0.04 | -1.31 | 1252684\|yceL\|multidrug resistance protein MdtH\|- |
| STM1167_401_435 | 0.00 | -2.08 | 1252685\|rimJ\|ribosomal-protein-S5-alanine N-acetyltransferase\|+ |
| STM1168_385_419 | 0.17 | -1.44 | 1252686\|yceH\|hypothetical protein\|+ |
| STM1169_65_99 | 0.09 | 1.31 | 1252687\|mviM\|putative virulence protein\|+ |
| STM1170_1152_1186 | 0.62 | -1.10 | 1252688\|mviN\|putative virulence protein\|+ |
| STM1171_13_47 | 0.41 | 1.21 | 1252689\|flgN\|putative FlgK/FlgL export chaperone\|- |
| STM1172_257_291 | 0.11 | 1.48 | 1252690\|flgM\|anti-sigma28 factor FlgM\|- |
| STM1173_129_163 | 0.95 | 1.02 | 1252691\|flgA\|flagellar basal body P-ring biosynthesis protein FlgA\|- |
| STM1175_289_323 | 0.49 | 1.20 | 1252693\|flgC\|flagellar basal body rod protein FlgC\|+ |
| STM1181_163_197 | 0.74 | -1.04 | 1252699\|flgI\|flagellar basal body P-ring protein\|+ |
| STM1183_1239_1273 | 0.01 | -2.29 | 1252701\|flgK\|flagellar hook-associated protein FlgK\|+ |
| STM1184_225_259 | 0.95 | 1.01 | 1252702\|flgL\|flagellar hook-associated protein FlgL\|+ |
| STM1185_2877_2911 | 0.02 | -1.70 | 1252703\|rne\|ribonuclease E\|- |
| STM1186_121_155 | 0.16 | 1.38 | 1252704\|STM1186\|pseudo\|+ |
| STM1187_737_771 | 0.08 | -1.26 | 1252705\|rluC\|23S rRNA pseudouridylate synthase C\|+ |
| STM1188_333_367 | 0.20 | -1.43 | 1252706\|STM1188\|putative inner membrane lipoprotein\|+ |
| STM1189_391_425 | 0.09 | -1.54 | 1252707\|maf\|Maf-like protein\|- |
| STM1190_225_259 | 0.02 | -2.30 | 1252708\|yceD\|hypothetical protein\|+ |
| STM1191_139_173 | 0.06 | -2.19 | 1252709\|rpmF\|50S ribosomal protein L32\|+ |
| STM1192_633_667 | 0.64 | 1.11 | 1252710\|plsX\|fatty acid/phospholipid synthesis protein\|+ |
| STM1193_289_323 | 0.28 | 1.17 | 1252711\|fabH\|3-oxoacyl-(acyl carrier protein) synthase III\|+ |
| STM1194_673_707 | 0.03 | 1.29 | 1252712\|fabD\|acyl carrier protein S-malonyltransferase\|+ |

| | | | |
|---|---|---|---|
| STM1195_257_291 | 0.14 | -1.38 | 1252713\|fabG\|3-ketoacyl-(acyl-carrier-protein) reductase\|+ |
| STM1196_202_236 | 0.54 | -1.09 | 1252714\|acpP\|acyl carrier protein\|+ |
| STM1197_1075_1109 | 0.68 | -1.08 | 1252715\|fabF\|3-oxoacyl-(acyl carrier protein) synthase II\|+ |
| STM1198_65_99 | 0.01 | 1.44 | 1252716\|pabC\|4-amino-4-deoxychorismate lyase\|+ |
| STM1199_760_794 | 0.13 | 1.42 | 1252717\|yceG\|hypothetical protein\|+ |
| STM1200_161_195 | 0.20 | -1.25 | 1252718\|tdk\|thymidylate kinase\|+ |
| STM1201_646_680 | 0.99 | 1.00 | 1252719\|holB\|DNA polymerase III subunit delta'\|+ |
| STM1202_353_387 | 0.02 | 1.46 | 1252720\|ycfH\|predicted metallodependent hydrolase\|+ |
| STM1203_1011_1045 | 0.02 | -1.81 | 1252721\|ptsG\|glucose-specific PTS system IIBC components\|+ |
| STM1204_2008_2042 | 0.01 | 2.07 | 1252722\|fhuE\|ferric-rhodotorulic acid outer membrane transporter\|- |
| STM1205_225_259 | 0.03 | -1.31 | 1252723\|ycfF\|purine nucleoside phosphoramidase\|+ |
| STM1206_173_207 | 0.40 | -1.16 | 1252724\|ycfL\|putative outer membrane lipoprotein\|+ |
| STM1207_593_627 | 0.02 | -1.41 | 1252725\|ycfM\|putative outer membrane lipoprotein\|+ |
| STM1209_91_125 | 0.12 | -1.15 | 1252727\|nagZ\|beta-hexosaminidase\|+ |
| STM1210_65_99 | 0.13 | -1.31 | 1252728\|ycfP\|hypothetical protein\|+ |
| STM1211_1170_1204 | 0.01 | 2.26 | 1252729\|ndh\|respiratory NADH dehydrogenase 2\|+ |
| STM1212_65_99 | 0.62 | 1.05 | 1252730\|ycfJ\|hypothetical protein\|+ |
| STM1213_63_97 | 0.00 | 2.42 | 1252731\|ycfQ\|putative transcriptional repressor\|- |
| STM1214_21_55 | 0.00 | 12.69 | 1252732\|ycfR\|putative outer membrane protein\|+ |
| STM1215_673_707 | 0.02 | 1.44 | 1252733\|ycfS\|putative periplasmic protein\|- |
| STM1216_2640_2674 | 0.79 | 1.02 | 1252734\|mfd\|transcription-repair coupling factor\|- |
| STM1217.S_537_571 | 0.03 | -1.35 | 1252735\|ycfU\|outer membrane-specific lipoprotein transporter subunit LolC\|+ |
| STM1218_49_83 | 0.00 | -1.61 | 1252736\|lolD\|lipoprotein transporter ATP-binding subunit\|+ |
| STM1219_758_792 | 0.02 | -2.12 | 1252737\|ycfW\|outer membrane-specific lipoprotein transporter subunit LolE\|+ |
| STM1220_449_483 | 0.00 | -2.06 | 1252738\|ycfX\|N-acetyl-D-glucosamine kinase\|+ |
| STM1221_3_37 | 0.24 | -1.16 | 1252739\|cobB\|NAD-dependent deacetylase\|+ |
| STM1222_912_946 | 0.10 | -2.13 | 1252740\|potD\|spermidine/putrescine ABC transporter periplasmic substrate-binding protein\|- |
| STM1223_145_179 | 0.62 | 1.11 | 1252741\|potC\|spermidine/putrescine ABC transporter membrane protein\|- |
| STM1224_796_830 | 0.06 | 1.78 | 1252742\|sifA\|secreted effector protein\|- |
| STM1225_649_683 | 0.37 | -1.07 | 1252743\|potB\|spermidine/putrescine ABC transporter membrane protein\|- |
| STM1226_650_684 | 0.19 | 1.21 | 1252744\|potA\|putrescine/spermidine ABC transporter ATPase protein\|- |
| STM1227_519_553 | 0.01 | -2.34 | 1252745\|pepT\|peptidase T\|+ |
| STM1228_432_466 | 0.88 | -1.04 | 1252746\|STM1228\|putative periplasmic protein\|+ |
| STM1229_667_701 | 0.03 | -1.28 | 1252747\|ycfD\|putative cytoplasmic protein\|- |
| STM1230_1009_1043 | 0.19 | 1.43 | 1252748\|phoQ\|sensor protein PhoQ\|- |

| | | | |
|---|---|---|---|
| STM1231_65_99 | 0.01 | 2.37 | 1252749lphoPlDNA-binding response regulator in two-component regulatory system with PhoQl- |
| STM1232_1332_1366 | 0.01 | -2.16 | 1252750lpurBladenylosuccinate lyasel- |
| STM1233_313_347 | 0.55 | -1.12 | 1252751lycfClhypothetical proteinl- |
| STM1234.S_940_974 | 0.02 | -2.11 | 1252752ltrmUltRNA (5-methylaminomethyl-2-thiouridylate)- |
| STM1235_290_325 | 0.90 | -1.02 | 1252753lymfBlputative MutT-like proteinl- |
| STM1236_33_67 | 0.48 | -1.06 | 1252754lSTM1236lputative periplasmic proteinl- |
| STM1237_305_339 | 0.07 | -1.30 | 1252755lymfClputative ribosomal large subunit pseudouridine synthasel- |
| STM1238_908_942 | 0.02 | -2.11 | 1252756licdAlisocitrate dehydrogenasel+ |
| STM1239_502_536 | 0.91 | -1.02 | 1252757lSTM1239lputative cytoplasmic proteinl+ |
| STM1240_49_83 | 0.07 | 1.96 | 1252758lenvFlputative envelope lipoproteinl- |
| STM1241.1N_42_76 | 0.84 | 1.02 | 2673773lSTM1241.1Nl-l+ |
| STM1241_17_51 | 0.16 | 1.46 | 1252759lmsgAlmacrophage survival proteinl- |
| STM1242_353_387 | 0.01 | 1.79 | 1252760lenvElputative envelope proteinl- |
| STM1244_201_235 | 0.04 | 3.16 | 1252762lpagDlvirulence protein PAGD precursorl- |
| STM1246_464_498 | 0.01 | 9.88 | 1252764lpagClvirulence membrane protein PAGC precursorl+ |
| STM1247_35_69 | 0.56 | -1.44 | 1252765lSTM1247ltRNAl+ |
| STM1248_117_151 | 0.00 | -3.38 | 1252766lSTM1248lpseudol+ |
| STM1249_165_199 | 0.71 | -1.04 | 1252767lSTM1249lputative periplasmic proteinl- |
| STM1250_145_179 | 0.07 | 1.63 | 1252768lSTM1250lputative cytoplasmic proteinl+ |
| STM1251_145_179 | 0.03 | 2.97 | 1252769lSTM1251lputative molecular chaperonel+ |
| STM1252_973_1007 | 0.04 | 2.13 | 1252770lSTM1252lputative cytoplasmic proteinl+ |
| STM1253_21_55 | 0.14 | 1.50 | 1252771lSTM1253lputative inner membrane proteinl- |
| STM1254_65_99 | 0.16 | 1.54 | 1252772lSTM1254lputative outer membrane lipoproteinl- |
| STM1256_385_419 | 0.48 | 1.06 | 1252774lSTM1256lputative ABC transporter proteinl+ |
| STM1257_737_771 | 0.24 | -1.07 | 1252775lSTM1257lputative ABC transporter proteinl+ |
| STM1259_65_99 | 0.25 | -1.19 | 1252777lSTM1259lputative ABC-type transport system ATPase componentl+ |
| STM1260_327_361 | 0.03 | 1.36 | 1252778lSTM1260lputative inner membrane proteinl- |
| STM1261_115_149 | 0.26 | -1.18 | 1252779lSTM1261lputative cytoplasmic proteinl+ |
| STM1262_3_37 | 0.09 | -1.43 | 1252780lSTM1262ltRNAl+ |
| STM1263_609_643 | 0.02 | -4.52 | 1252781lSTM1263lhypothetical proteinl+ |
| STM1264_65_99 | 0.03 | -1.45 | 1252782laadAlaminoglycoside adenyltransferasel+ |
| STM1265_501_535 | 0.99 | 1.00 | 1252783lSTM1265lputative response regulatorl+ |
| STM1266_641_675 | 0.32 | -1.13 | 1252784lSTM1266lputative transcriptional regulatorl+ |
| STM1267_97_131 | 0.03 | -2.34 | 1252785lSTM1267lputative cytoplasmic proteinl- |
| STM1269_353_387 | 0.07 | 1.37 | 1252787lSTM1269lchorismate mutasel+ |

| | | | |
|---|---|---|---|
| STM1270_353_389 | 0.01 | -1.18 | 1252788\|yeaS\|leucine export protein LeuE\|+ |
| STM1271_65_99 | 0.01 | 1.25 | 1252789\|yeaR\|putative cytoplasmic protein\|+ |
| STM1272_21_55 | 0.03 | 1.27 | 1252790\|yoaG\|putative cytoplasmic protein\|+ |
| STM1273_449_483 | 0.11 | 1.90 | 1252791\|STM1273\|putative nitric oxide reductase\|+ |
| STM1274_113_147 | 0.99 | -1.00 | 1252792\|yeaQ\|hypothetical protein\|+ |
| STM1275_193_227 | 0.01 | 2.27 | 1252793\|yaoF\|putative hemolysin\|+ |
| STM1276_129_163 | 0.02 | 1.28 | 1252794\|STM1276\|putative periplasmic protein\|+ |
| STM1277_273_307 | 0.03 | 1.54 | 1252795\|yeaO\|putative cytoplasmic protein\|- |
| STM1278_346_380 | 0.15 | -1.34 | 1252796\|yeaN\|putative amino acid/amine transport protein\|- |
| STM1279_689_723 | 0.00 | 1.81 | 1252797\|yeaM\|putative regulatory protein\|+ |
| STM1280_145_179 | 0.28 | -1.10 | 1252798\|yeaL\|putative inner membrane protein\|- |
| STM1281_5_39 | 0.02 | 1.78 | 1252799\|STM1281\|putative inner membrane protein\|+ |
| STM1282_73_107 | 0.01 | 2.88 | 1252800\|yeaK\|putative cytoplasmic protein\|- |
| STM1283_1295_1329 | 0.09 | 1.32 | 1252801\|yeaJ\|putative methyl-accepting chemotaxis protein\|- |
| STM1285_1416_1450 | 0.60 | -1.06 | 1252803\|yeaG\|putative serine protein kinase\|- |
| STM1286_673_707 | 0.01 | -2.54 | 1252804\|mipA\|MltA-interacting protein A\|+ |
| STM1287_358_392 | 0.13 | 1.26 | 1252805\|STM1287\|arylsulfatase regulator\|+ |
| STM1288_609_643 | 0.48 | -1.12 | 1252806\|STM1288\|putative aldehyde reductase\|+ |
| STM1289_609_643 | 0.96 | -1.00 | 1252807\|yeaD\|aldose 1-epimerase\|- |
| STM1290_801_835 | 0.01 | -1.82 | 1252808\|gapA\|glyceraldehyde-3-phosphate dehydrogenase\|- |
| STM1291_289_323 | 0.00 | -3.16 | 1252809\|yeaA\|methionine sulfoxide reductase B\|+ |
| STM1292_161_195 | 0.01 | -3.25 | 1252810\|yeaC\|putative cytoplasmic protein\|+ |
| STM1293_513_547 | 0.17 | 1.32 | 1252811\|pncA\|nicotinamidase/pyrazinamidase\|- |
| STM1294_34_68 | 0.10 | -1.28 | 1252812\|ansA\|cytoplasmic asparaginase I\|- |
| STM1295_1634_1668 | 0.13 | -1.28 | 1252813\|sppA\|protease 4\|- |
| STM1296_365_399 | 0.42 | 1.17 | 1252814\|ydjA\|putative oxidoreductase\|+ |
| STM1297_661_695 | 0.07 | 1.45 | 1252815\|selD\|selenophosphate synthetase\|+ |
| STM1298_1783_1817 | 0.29 | 1.20 | 1252816\|topB\|DNA topoisomerase III\|+ |
| STM1299_665_699 | 0.02 | -2.22 | 1252817\|gdhA\|glutamate dehydrogenase\|- |
| STM1300_145_179 | 0.03 | 2.13 | 1252818\|STM1300\|putative periplasmic protein\|+ |
| STM1301_273_307 | 0.15 | -1.35 | 1252819\|STM1301\|pyrimidine (deoxy)nucleoside triphosphate pyrophosphohydrolase\|- |
| STM1302_641_675 | 0.22 | 1.23 | 1252820\|xthA\|exonuclease III\|- |
| STM1303_484_518 | 0.20 | -1.14 | 1252821\|argD\|bifunctional succinylornithine transaminase/acetylornithine transaminase\|+ |
| STM1304_932_966 | 0.00 | -1.47 | 1252822\|astA\|arginine succinyltransferase\|+ |

| | | | |
|---|---|---|---|
| STM1305_1184_1218 | 0.03 | -1.29 | 1252823lastDlsuccinylglutamic semialdehyde dehydrogenasel+ |
| STM1308_241_275 | 0.15 | 1.27 | 1252826lspylperiplasmic proteinl+ |
| STM1309_869_903 | 0.01 | 1.81 | 1252827lSTM1309lendonuclease of nucleotide excision repairl- |
| STM1310_537_571 | 0.06 | 1.55 | 1252828lnadElNAD synthetasel- |
| STM1311_177_211 | 0.02 | 2.03 | 1252829losmElDNA-binding transcriptional activatorl+ |
| STM1312_241_275 | 0.06 | 1.49 | 1252830lcelAlN N'-diacetylchitobiose-specific PTS system transporter subunit IIBl+ |
| STM1314_137_171 | 0.00 | -1.46 | 1252832lcelClN N'-diacetylchitobiose-specific PTS system transporter subunit IIAl+ |
| STM1315_105_139 | 0.00 | -1.49 | 1252833lcelDlDNA-binding transcriptional dual regulatorl+ |
| STM1316_637_671 | 0.75 | -1.12 | 1252834lcelFlphospho-beta-glucosidase/cellobiose-6-phosphate hydrolasel+ |
| STM1317_321_355 | 0.30 | -1.09 | 1252835lcelGlhypothetical proteinl+ |
| STM1318_1662_1696 | 0.05 | 1.93 | 1252836lkatElhydroperoxidase IIl- |
| STM1319_20_56 | 0.02 | 1.25 | 1252837lcedAlcell division modulatorl+ |
| STM1320_537_571 | 0.13 | -1.53 | 1252838lydjNlkinase/transporter-like proteinl- |
| STM1321_105_139 | 0.02 | 1.53 | 1252839lydjMlhypothetical proteinl- |
| STM1322_145_179 | 0.15 | 1.10 | 1252840lyniClpredicted hydrolasel- |
| STM1323_407_441 | 0.01 | -1.83 | 1252841lyniBlputative regulatory proteinl+ |
| STM1324_769_803 | 0.01 | -1.83 | 1252842lSTM1324lputative cytoplasmic proteinl- |
| STM1325_225_259 | 0.30 | 1.14 | 1252843lydiZlputative cytoplasmic proteinl- |
| STM1326_433_467 | 0.13 | 1.30 | 1252844lpfkBl6-phosphofructokinase 2l- |
| STM1327_721_755 | 0.75 | 1.15 | 1252845lydiYlputative outer membrane proteinl+ |
| STM1328_905_939 | 0.00 | 2.95 | 1252846lSTM1328lputative outer membrane proteinl- |
| STM1332_931_965 | 0.03 | -1.50 | 1252850lrfclO-antigen polymerasel- |
| STM1333_1522_1556 | 0.29 | -1.21 | 1252851lthrSlthreonyl-tRNA synthetasel+ |
| STM1334.c_417_451 | 0.74 | -1.08 | 1252852linfCltranslation initiation factor IF-3l+ |
| STM1335_109_144 | 0.02 | -2.23 | 1252853lrpmIl50S ribosomal protein L35l+ |
| STM1336_129_163 | 0.01 | -2.18 | 1252854lrplTl50S ribosomal protein L20l+ |
| STM1337_585_619 | 0.05 | 1.69 | 1252855lpheSlphenylalanyl-tRNA synthetase subunit alphal+ |
| STM1338_1933_1967 | 0.00 | -1.48 | 1252856lpheTlphenylalanyl-tRNA synthetase subunit betal+ |
| STM1339_205_239 | 0.01 | 1.70 | 1252857lihfAlintegration host factor subunit alphal+ |
| STM1340_577_611 | 0.60 | -1.13 | 1252858lbtuClvtamin B12-transporter permeasel+ |
| STM1341_397_431 | 0.00 | -1.67 | 1252859lbtuElpredicted glutathione peroxidasel+ |
| STM1342_609_643 | 0.03 | 1.28 | 1252860lbtuDlvitamin B12-transporter ATPasel+ |
| STM1343_97_131 | 0.17 | -1.19 | 1252861lnlpCllipoproteinl+ |
| STM1344_529_563 | 0.06 | 1.44 | 1252862lydiVlhypothetical proteinl+ |

| | | | |
|---|---|---|---|
| STM1345_828_862 | 0.07 | 1.25 | 1252863lydiUlhypothetical proteinl+ |
| STM1346_129_163 | 0.65 | -1.15 | 1252864lydiElhypothetical proteinl- |
| STM1347_336_370 | 0.06 | -1.41 | 1252865laroHlphospho-2-dehydro-3-deoxyheptonate aldolasel- |
| STM1348_513_547 | 0.24 | -1.09 | 1252866lydiAlhypothetical proteinl- |
| STM1349_1444_1478 | 0.01 | -1.51 | 1252867lppslphosphoenolpyruvate synthasel+ |
| STM1350_1442_1476 | 0.06 | 1.20 | 1252868lydiDlshort chain acyl-CoA synthetasel- |
| STM1351_137_171 | 0.08 | 1.58 | 1252869lydiTlputative ferredoxinl- |
| STM1352_816_850 | 0.81 | -1.02 | 1252870lydiSlflavoproteinl- |
| STM1355_257_291 | 0.20 | 1.50 | 1252873lydiPlputative transcriptional regulatorl+ |
| STM1356_345_379 | 0.04 | 1.62 | 1252874lydiOlputative acyl-CoA dehydrogenasel- |
| STM1357.S_725_759 | 0.58 | -1.13 | 1252875lydiFlputative acetyl-CoA/acetoacetyl-CoA transferase beta subunitl- |
| STM1358_449_483 | 0.46 | 1.13 | 1252876laroDl3-dehydroquinate dehydratasel- |
| STM1362_325_359 | 0.00 | -3.10 | 1252880lydiLlputative cytoplasmic proteinl- |
| STM1363_49_83 | 0.07 | 2.01 | 1252881lrprAlmisc_RNAl- |
| STM1364_712_746 | 0.04 | 1.18 | 1252882lydiKlpredicted inner membrane proteinl- |
| STM1365_2362_2396 | 0.20 | -1.29 | 1252883lydiJlputative oxidasel+ |
| STM1366_185_219 | 0.16 | 1.21 | 1252884lSTM1366lhypothetical proteinl+ |
| STM1367_11_49 | 0.21 | -1.36 | 1252885lydiHlputative cytoplasmic proteinl+ |
| STM1369_137_171 | 0.01 | 1.83 | 1252887lsufAliron-sulfur cluster assembly scaffold proteinl+ |
| STM1370_1001_1035 | 0.01 | 2.17 | 1252888lsufBlcysteine desulfurase activator complex subunit SufBl+ |
| STM1371_513_547 | 0.04 | 1.32 | 1252889lsufClcysteine desulfurase ATPase componentl+ |
| STM1373_478_512 | 0.00 | 1.47 | 1252891lsufSlselenocysteine lyasel+ |
| STM1375_195_229 | 0.32 | -1.09 | 1252893lynhGlhypothetical proteinl+ |
| STM1377_103_137 | 0.12 | 1.40 | 1252895llpplmurein lipoproteinl- |
| STM1378_622_656 | 0.09 | -1.32 | 1252896lpykFlpyruvate kinasel- |
| STM1379_377_411 | 0.00 | -3.03 | 1252897lorf48lputative amino acid permeasel- |
| STM1380_545_579 | 0.01 | -2.60 | 1252898lorf32lputative proline iminopeptidasel- |
| STM1381_325_359 | 0.00 | -2.85 | 1252899lorf245lputative cytoplasmic proteinl- |
| STM1382_676_710 | 0.53 | -1.08 | 1252900lorf408lputative regulatory proteinl- |
| STM1383_2440_2474 | 0.05 | 2.68 | 1252901lttrAltetrathionate reductase complex subunit Al- |
| STM1384_432_466 | 0.92 | 1.01 | 1252902lttrCltetrathionate reductase complex subunit Cl- |
| STM1387_161_195 | 0.01 | -1.61 | 1252905lttrRlresponse regulatorl+ |
| STM1388_177_211 | 0.09 | -1.47 | 1252906lorf70lhypothetical proteinl+ |
| STM1389_449_483 | 0.05 | -1.52 | 1252907lorf319lputative inner membrane proteinl- |
| STM1390_129_163 | 0.02 | 1.81 | 1252908lorf242lputative regulatory proteinl- |
| STM1391_129_163 | 0.04 | 1.58 | 1252909lssrBltranscriptional activatorl- |

| | | | |
|---|---|---|---|
| STM1394_559_593 | 0.29 | 1.13 | 1252912\|ssaC\|outer membrane secretin precursor\|+ |
| STM1395_757_791 | 0.30 | 1.22 | 1252913\|ssaD\|virulence protein\|+ |
| STM1396_153_187 | 0.18 | 1.55 | 1252914\|ssaE\|secretion system effector\|+ |
| STM1397_89_123 | 0.03 | 2.46 | 1252915\|sseA\|secretion system chaperone protein\|+ |
| STM1398_409_443 | 0.90 | 1.62 | 1252916\|sseB\|translocation machinery component\|+ |
| STM1399_385_419 | 0.31 | 1.13 | 1252917\|sscA\|secretion system chaparone\|+ |
| STM1400_1172_1206 | 0.04 | 1.58 | 1252918\|sseC\|translocation machinery component\|+ |
| STM1401_281_315 | 0.34 | 1.42 | 1252919\|sseD\|translocation machinery component\|+ |
| STM1402_9_43 | 0.34 | 1.46 | 1252920\|sseE\|secreted effector protein\|+ |
| STM1403_274_308 | 0.05 | 1.39 | 1252921\|sscB\|secretion system chaparone\|+ |
| STM1404_593_627 | 0.01 | 1.40 | 1252922\|sseF\|secreted effector protein\|+ |
| STM1405_577_611 | 0.15 | 1.38 | 1252923\|sseG\|secreted effector protein\|+ |
| STM1406_93_127 | 0.53 | 3.69 | 1252924\|ssaG\|type III secretion system apparatus protein\|+ |
| STM1407_2_36 | 0.01 | 2.44 | 1252925\|ssaH\|type III secretion system apparatus protein\|+ |
| STM1408_161_195 | 0.08 | 1.37 | 1252926\|ssaI\|type III secretion system apparatus protein\|+ |
| STM1409_49_83 | 0.16 | 1.50 | 1252927\|ssaJ\|needle complex inner membrane lipoprotein\|+ |
| STM1410_109_143 | 0.01 | 1.85 | 1252928\|STM1410\|putative cytoplasmic protein\|+ |
| STM1411_513_547 | 0.05 | 1.39 | 1252929\|ssaK\|type III secretion system apparatus protein\|+ |
| STM1412_658_692 | 0.05 | 2.02 | 1252930\|ssaL\|type III secretion system apparatus protein\|+ |
| STM1414_1087_1121 | 0.98 | 2.00 | 1252932\|ssaV\|secretion system apparatus protein SsaV\|+ |
| STM1416_257_291 | 0.05 | 1.55 | 1252934\|ssaO\|type III secretion system apparatus protein\|+ |
| STM1417_166_200 | 0.10 | 1.41 | 1252935\|ssaP\|type III secretion system apparatus protein\|+ |
| STM1418_529_563 | 0.18 | 1.62 | 1252936\|ssaQ\|type III secretion system protein\|+ |
| STM1419_273_307 | 0.37 | 1.58 | 1252937\|ssaR\|type III secretion system protein\|+ |
| STM1420_189_223 | 0.33 | 1.22 | 1252938\|ssaS\|type III secretion system apparatus protein\|+ |
| STM1421_305_339 | 0.01 | 1.38 | 1252939\|ssaT\|type III secretion system apparatus protein\|+ |
| STM1423_39_73 | 0.86 | 1.24 | 1252941\|valW\|tRNA\|- |
| STM1424_38_72 | 0.58 | -1.42 | 1252942\|valV\|tRNA\|- |
| STM1425_823_857 | 0.02 | -2.46 | 1252943\|ydhE\|multidrug efflux protein\|- |
| STM1426_529_563 | 0.68 | 1.08 | 1252944\|ribE\|riboflavin synthase subunit alpha\|+ |
| STM1427_1030_1064 | 0.04 | -1.68 | 1252945\|cfa\|cyclopropane fatty acyl phospholipid synthase\|- |
| STM1428_335_369 | 0.15 | 2.15 | 1252946\|ydhC\|inner membrane transport protein YdhC\|- |
| STM1429_769_803 | 0.01 | 1.21 | 1252947\|ydhB\|putative DNA-binding transcriptional regulator\|+ |
| STM1430_699_733 | 0.06 | -1.21 | 1252948\|purR\|DNA-binding transcriptional repressor PurR\|- |
| STM1431_61_95 | 0.25 | 1.31 | 1252949\|sodB\|superoxide dismutase\|- |
| STM1432_713_747 | 0.20 | -1.19 | 1252950\|ydhO\|putative cell wall-associated hydrolase\|- |

| | | | |
|---|---|---|---|
| STM1433_165_199 | 0.55 | 1.16 | 1252951lydhDlhypothetical proteinl+ |
| STM1434_225_259 | 0.44 | -1.10 | 1252952lrntlribonuclease TIl- |
| STM1435_321_355 | 0.06 | 1.47 | 1252953lgloAlglyoxalase IIl- |
| STM1436_995_1029 | 0.24 | -1.17 | 1252954lnemAlN-ethylmaleimide reductase  FMN-linkedl- |
| STM1437_561_595 | 0.13 | -1.12 | 1252955lydhMlputative transcriptional repressorl- |
| STM1438_29_63 | 0.05 | 1.79 | 1252956lydhLlputative oxidoreductasel+ |
| STM1439_593_627 | 0.01 | -2.18 | 1252957lydhFlputative aldo/keto reductasel+ |
| STM1440_105_139 | 0.00 | -2.06 | 1252958lsodClsuperoxide dismutasel+ |
| STM1441_1421_1455 | 0.79 | -1.22 | 1252959lSTM1441lputative inner membrane proteinl- |
| STM1442_97_131 | 0.03 | -1.61 | 1252960lydhJlputative multidrug resistance efflux pumpl- |
| STM1443_121_155 | 0.49 | -1.12 | 1252961lydhIlputative inner membrane proteinl- |
| STM1444_129_163 | 0.03 | 1.49 | 1252962lslyAltranscriptional regulator SlyAl+ |
| STM1445_389_423 | 0.05 | 1.47 | 1252963lslyBlputative outer membrane lipoproteinl- |
| STM1446_1083_1117 | 0.31 | -1.16 | 1252964lanmKlanhydro-N-acetylmuramic acid kinasel+ |
| STM1447_113_147 | 0.53 | 1.08 | 1252965lydhAlhypothetical proteinl+ |
| STM1448_177_211 | 0.05 | -1.35 | 1252966lpdxHlpyridoxamine 5'-phosphate oxidasel+ |
| STM1449_1204_1238 | 0.02 | -1.21 | 1252967ltyrSltyrosyl-tRNA synthetasel+ |
| STM1450_129_163 | 0.13 | -1.37 | 1252968lpdxYlpyridoxamine kinasel+ |
| STM1451_465_499 | 0.02 | -2.57 | 1252969lgstlglutathionine S-transferasel- |
| STM1452_667_701 | 0.02 | -1.75 | 1252970ltppBlputative tripeptide transporter permeasel- |
| STM1453_537_571 | 0.05 | 1.51 | 1252971lnthlendonuclease IIIl- |
| STM1454_257_291 | 0.02 | 1.47 | 1252972lydgQlSoxR-reducing system protein RsxEl- |
| STM1455_49_83 | 0.01 | -2.47 | 1252973lydgPlelectron transport complex protein RnfGl- |
| STM1456_524_558 | 0.00 | -2.31 | 1252974lrnfDlelectron transport complex protein RnfDl- |
| STM1457_1225_1259 | 0.13 | -1.34 | 1252975lSTM1457lelectron transport complex protein RnfCl- |
| STM1458_417_451 | 0.01 | -1.87 | 1252976lydgMlelectron transport complex protein RnfBl- |
| STM1459_321_355 | 0.04 | -1.41 | 1252977lSTM1459lNa(+)-translocating NADH-quinone reductase subunit El- |
| STM1460_81_115 | 0.07 | -1.33 | 1252978lydgKlputative inner membrane proteinl- |
| STM1461.S_161_195 | 0.01 | 1.39 | 1252979lydgTloriC-binding nucleoid-associated proteinl- |
| STM1462.S_714_748 | 0.05 | 1.41 | 1252980lydgJlputative oxidoreductasel+ |
| STM1463_267_301 | 0.47 | 1.18 | 1252981laddladenosine deaminasel- |
| STM1464_37_71 | 0.00 | -1.21 | 1252982lmalYlpseudol- |
| STM1465_145_179 | 0.01 | -1.28 | 1252983lmalXlpseudol- |
| STM1466_1230_1264 | 0.02 | 1.21 | 1252984lydgAlputative periplasmic proteinl- |
| STM1467_1073_1107 | 0.16 | -1.19 | 1252985lmanAlmannose-6-phosphate isomerasel- |
| STM1468_1080_1114 | 0.00 | -8.24 | 1252986lfumAlfumarase Al+ |

| | | | |
|---|---|---|---|
| STM1469_469_503 | 0.00 | -3.45 | 1252987\|fumC\|fumarate hydratase\|+ |
| STM1471_1007_1041 | 0.01 | -1.92 | 1252989\|rstB\|sensor protein RstB\|- |
| STM1473_647_681 | 0.05 | 1.28 | 1252991\|ompN\|outer membrane protein N precursor\|- |
| STM1474_188_222 | 0.27 | 1.14 | 1252992\|STM1474\|pseudo\|+ |
| STM1475_131_167 | 0.56 | 1.07 | 1252993\|rstA\|DNA-binding response regulator in two-component regulatory system with RstB\|- |
| STM1476_171_205 | 0.05 | -1.72 | 1252994\|ydgC\|putative inner membrane protein\|+ |
| STM1477_1280_1314 | 0.06 | 1.37 | 1252995\|ydgI\|putative amino acid transporter\|- |
| STM1478_865_899 | 0.22 | -1.29 | 1252996\|ydgH\|putative periplasmic protein\|- |
| STM1479_739_773 | 0.52 | 1.11 | 1252997\|pntA\|NAD(P) transhydrogenase subunit alpha\|+ |
| STM1480_1126_1160 | 0.09 | -1.26 | 1252998\|pntB\|pyridine nucleotide transhydrogenase\|+ |
| STM1481_292_326 | 0.40 | 1.09 | 1252999\|tqsA\|putative transport protein\|- |
| STM1483_285_319 | 0.25 | 1.13 | 1253001\|ydgE\|multidrug efflux system protein MdtI\|+ |
| STM1484_432_466 | 0.01 | 1.45 | 1253002\|STM1484\|putative protease\|- |
| STM1486_1155_1189 | 0.03 | 1.92 | 1253004\|ynfM\|putative transport protein\|- |
| STM1487_417_451 | 0.02 | -1.23 | 1253005\|ynfL\|putative transcriptional regulator\|+ |
| STM1488_830_864 | 0.30 | 1.10 | 1253006\|mlc\|pts operon transcriptional repressor\|+ |
| STM1489_65_99 | 0.02 | -1.96 | 1253007\|bioD\|putative dithiobiotin synthetase\|+ |
| STM1490_575_609 | 0.95 | -1.02 | 1253008\|STM1490\|putative voltage-gated ClC-type chloride channel ClcB\|- |
| STM1491_966_1000 | 0.09 | -1.19 | 1253009\|STM1491\|proline/glycine betaine transport systems\|- |
| STM1492_289_323 | 0.31 | 1.07 | 1253010\|STM1492\|putative ABC transporter permease component\|- |
| STM1494_97_131 | 0.40 | 1.11 | 1253012\|STM1494\|putative transport system permease component\|- |
| STM1495_65_99 | 0.37 | 1.05 | 1253013\|ynfI\|twin-argninine leader-binding protein DmsD\|- |
| STM1496_577_611 | 0.18 | 1.15 | 1253014\|STM1496\|putative dimethylsulfoxide reductase\|- |
| STM1497_92_126 | 0.47 | 1.56 | 1253015\|STM1497\|putative dimethyl sulphoxide reductase\|- |
| STM1498_1597_1631 | 0.03 | 2.10 | 1253016\|STM1498\|putative dimethyl sulphoxide reductase\|- |
| STM1499_2211_2245 | 0.27 | 1.52 | 1253017\|STM1499\|putative dimethyl sulphoxide reductase chain A1\|- |
| STM1500_100_134 | 0.03 | 1.46 | 1253018\|ynfD\|putative outer membrane protein\|- |
| STM1501_33_67 | 0.06 | 1.22 | 1253019\|ynfC\|hypothetical protein\|+ |
| STM1502_409_443 | 0.80 | 1.04 | 1253020\|speG\|spermidine N1-acetyltransferase\|- |
| STM1503_97_131 | 0.02 | 1.35 | 1253021\|ynfB\|putative periplasmic protein\|- |
| STM1504_273_308 | 0.07 | -1.12 | 1253022\|ynfA\|hypothetical protein\|+ |
| STM1505_1144_1178 | 0.05 | 2.67 | 1253023\|rspA\|putative dehydratase\|+ |
| STM1506_789_823 | 0.23 | -1.13 | 1253024\|rspB\|putative dehydrogenase\|+ |
| STM1509_145_179 | 0.06 | -1.62 | 1253027\|ydfZ\|putative cytoplasmic protein\|- |
| STM1510_65_99 | 0.29 | 1.19 | 1253028\|ydfH\|putative regulatory protein\|- |

| | | | |
|---|---|---|---|
| STM1511_65_99 | 0.33 | -1.11 | 1253029\|ydfG\|L-allo-threonine dehydrogenase  NAD(P)-binding\|- |
| STM1512_1828_1862 | 0.04 | -1.24 | 1253030\|dcp\|dipeptidyl carboxypeptidase II\|+ |
| STM1513_145_179 | 0.09 | 1.24 | 1253031\|STM1513\|putative cytoplasmic protein\|+ |
| STM1514_4_41 | 0.05 | 2.69 | 1253032\|ydeJ\|competence damage-inducible protein A\|- |
| STM1515_193_227 | 0.01 | 1.30 | 1253033\|ydeI\|hypothetical protein\|+ |
| STM1516_797_831 | 0.43 | 1.19 | 1253034\|ydeE\|putative MFS-type transporter YdeE\|- |
| STM1517_401_435 | 0.69 | 1.12 | 1253035\|ydeD\|O-acetylserine/cysteine export protein\|+ |
| STM1519.S_225_259 | 0.01 | -2.10 | 1253037\|marA\|DNA-binding transcriptional activator MarA\|- |
| STM1520_257_291 | 0.01 | 1.92 | 1253038\|marR\|DNA-binding transcriptional repressor MarR\|- |
| STM1521_337_371 | 0.01 | 1.63 | 1253039\|marC\|multiple drug resistance protein MarC\|+ |
| STM1522_1056_1090 | 0.04 | 1.49 | 1253040\|ydeA\|sugar efflux transporter\|- |
| STM1523_817_851 | 0.15 | 1.27 | 1253041\|yneJ\|putative transcriptional regulator\|- |
| STM1524_726_760 | 0.03 | 1.46 | 1253042\|yneI\|putative succinate-semialdehyde dehydrogenase\|+ |
| STM1525_81_115 | 0.29 | 1.11 | 1253043\|yneH\|glutaminase\|+ |
| STM1527_601_635 | 0.01 | 1.56 | 1253045\|STM1527\|putative inner membrane protein\|+ |
| STM1528_401_435 | 0.02 | 2.44 | 1253046\|STM1528\|putative outer membrane protein\|- |
| STM1531_289_323 | 0.07 | 1.69 | 1253049\|STM1531\|putative hydrogenase\|- |
| STM1532_545_579 | 0.08 | 1.14 | 1253050\|STM1532\|putative dehydrogenase protein\|- |
| STM1533_575_609 | 0.12 | -1.27 | 1253051\|STM1533\|putative hydrogenase\|- |
| STM1534_369_403 | 0.62 | -1.03 | 1253052\|STM1534\|putative hydrogenase\|- |
| STM1535_65_99 | 0.01 | 1.14 | 1253053\|STM1535\|putative hydrogenase protein\|- |
| STM1536_321_355 | 0.00 | -1.52 | 1253054\|STM1536\|putative hydrogenase maturation protease\|- |
| STM1537_33_67 | 0.06 | 2.35 | 1253055\|STM1537\|putative Ni/Fe hydrogenase 1 b-type cytochrome subunit\|- |
| STM1539_515_549 | 0.00 | 2.14 | 1253057\|STM1539\|putative hydrogenase-1 small subunit\|- |
| STM1540_221_255 | 0.00 | 5.67 | 1253058\|STM1540\|putative hydrolase\|+ |
| STM1541_385_419 | 0.02 | 1.48 | 1253059\|STM1541\|putative regulatory protein\|+ |
| STM1542_899_933 | 0.83 | -1.03 | 1253060\|STM1542\|putative zinc-binding dehydrogenase\|- |
| STM1543_1192_1226 | 0.17 | 1.28 | 1253061\|STM1543\|putative transport protein\|- |
| STM1544_1486_1520 | 0.01 | 3.53 | 1253062\|pqaA\|PhoPQ-regulated protein\|- |
| STM1545_979_1013 | 0.23 | -1.12 | 1253063\|STM1545\|putative multidrug efflux protein\|- |
| STM1548.s_809_843 | 0.01 | 3.06 | 1253066\|STM1548.s\|putative S-adenosylmethionine/tRNA-ribosyltransferase-isomerase\|+ |
| STM1549_52_86 | 0.03 | -1.32 | 1253067\|STM1549\|putative translation initiation inhibitor\|- |
| STM1550_105_139 | 0.06 | 1.25 | 1253068\|STM1550\|putative cytoplasmic protein\|- |
| STM1551_177_211 | 0.05 | 1.50 | 1253069\|STM1551\|putative cytoplasmic protein\|- |
| STM1551.1n_273_307 | 0.72 | -1.03 | 2673754\|STM1551.1n\|hypothetical protein\|+ |

| | | | |
|---|---|---|---|
| STM1552_729_763 | 0.52 | 1.07 | 1253070\|STM1552\|putative cytoplasmic protein\|+ |
| STM1553_561_595 | 0.04 | 1.34 | 1253071\|STM1553\|pseudo\|- |
| STM1554_788_822 | 0.62 | 1.11 | 1253072\|STM1554\|putative coiled-coil protein\|- |
| STM1557_620_654 | 0.33 | -1.30 | 1253075\|STM1557\|putative aminotransferase\|+ |
| STM1558_1845_1879 | 0.02 | -1.44 | 1253076\|STM1558\|putative glycosyl hydrolase\|- |
| STM1559_1690_1724 | 0.02 | -1.56 | 1253077\|STM1559\|putative glycosyl hydrolase\|- |
| STM1560_850_884 | 0.10 | 1.11 | 1253078\|STM1560\|putative alpha amylase\|- |
| STM1561_249_283 | 0.05 | 1.77 | 1253079\|STM1561\|putative lipoprotein\|- |
| STM1562_45_79 | 0.03 | 3.31 | 1253080\|hdeB\|acid-resistance protein\|- |
| STM1563_225_259 | 0.03 | 1.67 | 1253081\|osmC\|putative envelope protein\|- |
| STM1564_121_155 | 0.01 | 1.74 | 1253082\|yddX\|biofilm-dependent modulation protein\|+ |
| STM1565_5_39 | 0.73 | 1.05 | 1253083\|rpsV\|30S ribosomal subunit S22\|+ |
| STM1566_1499_1533 | 0.08 | -1.51 | 1253084\|sfcA\|NAD-linked malate dehydrogenase\|+ |
| STM1567_332_366 | 0.10 | 1.43 | 1253085\|adhP\|alcohol dehydrogenase\|+ |
| STM1568_113_147 | 0.07 | 1.63 | 1253086\|fdnI\|formate dehydrogenase-N subunit gamma\|- |
| STM1569_705_739 | 0.31 | 1.11 | 1253087\|fdnH\|formate dehydrogenase-N beta subunit\|- |
| STM1571_225_259 | 0.08 | 1.41 | 1253089\|yddG\|hypothetical protein\|+ |
| STM1572_730_764 | 0.01 | -2.26 | 1253090\|nmpC\|putative outer membrane porin precursor\|+ |
| STM1573.Sc_289_323 | 0.02 | -1.54 | 1253091\|STM1573.Sc\|putative cytoplasmic protein\|+ |
| STM1574_649_683 | 0.04 | 1.28 | 1253092\|smvA\|methyl viologen resistance\|- |
| STM1575_377_411 | 0.02 | 1.79 | 1253093\|STM1575\|putative transcriptional regulator\|+ |
| STM1576_982_1016 | 0.07 | -1.42 | 1253094\|narU\|nitrate extrusion protein\|+ |
| STM1577_3606_3640 | 0.11 | 1.50 | 1253095\|narZ\|nitrate reductase 2 alpha subunit\|+ |
| STM1578_1282_1316 | 0.01 | -2.67 | 1253096\|narY\|nitrate reductase 2 beta subunit\|+ |
| STM1579_65_99 | 0.01 | -2.03 | 1253097\|narW\|nitrate reductase 2 delta subunit\|+ |
| STM1580_129_163 | 0.00 | -3.40 | 1253098\|narV\|nitrate reductase 2 gamma subunit\|+ |
| STM1581_321_355 | 0.61 | 1.06 | 1253099\|yddE\|hypothetical protein\|+ |
| STM1582_681_715 | 0.54 | 1.07 | 1253100\|nhoA\|putative arylamine N-acetyltransferase\|- |
| STM1583_129_163 | 0.00 | 2.60 | 1253101\|STM1583\|putative cytoplasmic protein\|- |
| STM1584_1311_1345 | 0.08 | 1.17 | 1253102\|ansP\|L-asparagine transport protein\|+ |
| STM1585_65_99 | 0.02 | 1.99 | 1253103\|STM1585\|putative outer membrane lipoprotein\|- |
| STM1586_575_609 | 0.05 | -1.52 | 1253104\|STM1586\|putative periplasmic protein\|- |
| STM1587_1506_1540 | 0.01 | 1.28 | 1253105\|yncD\|putative outer membrane receptor\|+ |
| STM1588_449_483 | 0.60 | -1.09 | 1253106\|yncC\|putative DNA-binding transcriptional regulator\|- |
| STM1589_296_330 | 0.02 | -1.14 | 1253107\|yncB\|putative NADP-dependent oxidoreductase\|- |
| STM1590_385_419 | 0.80 | 1.04 | 1253108\|yncA\|putative acyltransferase\|+ |

| | | | |
|---|---|---|---|
| STM1591_86_121 | 0.04 | -1.14 | 1253109\|ydcZ\|putative inner membrane protein\|+ |
| STM1592_149_183 | 0.07 | 1.33 | 1253110\|ydcY\|putative cytoplasmic protein\|- |
| STM1593_321_355 | 0.03 | -1.18 | 1253111\|srfA\|putative virulence protein\|+ |
| STM1594_2047_2081 | 0.11 | -1.23 | 1253112\|srfB\|putative virulence protein\|+ |
| STM1595_1418_1452 | 0.22 | -1.14 | 1253113\|srfC\|putative virulence protein\|+ |
| STM1596_67_102 | 0.25 | -1.11 | 1253114\|ydcX\|putative inner membrane protein\|- |
| STM1597_874_908 | 0.00 | -2.11 | 1253115\|ydcW\|putative aldehyde dehydrogenase\|- |
| STM1598_530_564 | 0.02 | 1.33 | 1253116\|ydcR\|putative regulatory protein\|- |
| STM1599_289_323 | 0.03 | 2.49 | 1253117\|pdgL\|periplasmic dipeptidase precursor\|- |
| STM1601_70_107 | 0.05 | 1.68 | 1253119\|ugtL\|hypothetical protein\|- |
| STM1602_461_495 | 0.05 | 1.65 | 1253120\|sifB\|secreted effector protein\|+ |
| STM1603_115_149 | 0.02 | -1.85 | 1253121\|yncJ\|putative periplasmic protein\|+ |
| STM1604_1798_1832 | 0.07 | 1.63 | 1253122\|ydcP\|putative collagenase\|- |
| STM1605_242_276 | 0.01 | 1.68 | 1253123\|ydcN\|putative repressor\|- |
| STM1606_452_486 | 0.11 | -1.27 | 1253124\|STM1606\|putative benzoate membrane transport protein\|+ |
| STM1607_617_651 | 0.30 | -1.11 | 1253125\|STM1607\|putative outer membrane lipoprotein\|- |
| STM1608_129_163 | 0.19 | -1.24 | 1253126\|tehB\|tellurite resistance protein TehB\|- |
| STM1609_55_89 | 0.15 | -1.13 | 1253127\|tehA\|potassium-tellurite ethidium and proflavin transporter\|- |
| STM1611_337_371 | 0.04 | 1.64 | 1253129\|rimL\|ribosomal-protein-L7/L12-serine acetyltransferase\|- |
| STM1613_17_51 | 0.02 | -2.43 | 1253131\|STM1613\|putative PTS system enzymeIIB component\|+ |
| STM1614_667_701 | 0.01 | -1.97 | 1253132\|STM1614\|putative PTS system enzyme IIC component\|+ |
| STM1615_385_419 | 0.05 | -1.41 | 1253133\|STM1615\|putative nucleoside triphosphatase\|+ |
| STM1617_577_611 | 0.01 | -1.83 | 1253135\|STM1617\|ribulose-phosphate 3-epimerase\|+ |
| STM1618_129_163 | 0.01 | -1.47 | 1253136\|STM1618\|putative transcriptional repressor of sgc operon\|+ |
| STM1619_401_435 | 0.44 | -1.13 | 1253137\|STM1619\|cryptic aminoglycoside resistance gene\|+ |
| STM1620_1036_1070 | 0.60 | -1.04 | 1253138\|STM1620\|putative oxidase\|- |
| STM1621_137_171 | 0.52 | -1.08 | 1253139\|STM1621\|putative periplasmic protein\|- |
| STM1622_1617_1651 | 0.02 | -1.63 | 1253140\|mdoD\|glucan biosynthesis protein D\|- |
| STM1623_1294_1328 | 0.04 | -2.02 | 1253141\|STM1623\|putative carboxylesterase\|- |
| STM1624_665_699 | 0.00 | -5.65 | 1253142\|STM1624\|putative cytoplasmic protein\|- |
| STM1625_833_867 | 0.00 | -11.25 | 1253143\|ydcI\|putative transcriptional regulator\|+ |
| STM1626_1427_1461 | 0.00 | -2.50 | 1253144\|trg\|methyl-accepting chemotaxis protein III\|- |
| STM1627_248_282 | 0.03 | 1.34 | 1253145\|STM1627\|alcohol dehydrogenase class III\|- |
| STM1628_85_119 | 0.39 | 1.13 | 1253146\|STM1628\|putative cytoplasmic protein\|- |
| STM1630_145_179 | 0.01 | 3.85 | 1253148\|STM1630\|putative inner membrane protein\|- |
| STM1631_1124_1158 | 0.01 | 3.76 | 1253149\|sseJ\|secreted effector protein\|+ |

| | | | |
|---|---|---|---|
| STM1632_209_243 | 0.01 | 1.94 | 1253150lSTM1632lputative inner membrane proteinl- |
| STM1633_417_451 | 0.01 | 1.82 | 1253151lSTM1633lputative periplasmic binding proteinl+ |
| STM1634_273_307 | 0.51 | 1.08 | 1253152lSTM1634lputative ABC transporter permease componentl+ |
| STM1635_513_547 | 0.66 | 1.04 | 1253153lSTM1635lputative ABC-type polar amino acid transport system ATPase componentl+ |
| STM1636_281_315 | 0.09 | -1.32 | 1253154lSTM1636lputative ABC-type transport system membrane componentl+ |
| STM1637_1192_1226 | 0.20 | -1.30 | 1253155lSTM1637lputative inner membrane proteinl+ |
| STM1638_321_355 | 0.18 | -1.28 | 1253156lSTM1638lputative SAM-dependent methyltransferasel+ |
| STM1639_301_335 | 0.01 | -2.18 | 1253157lcybBlcytochrome b561l- |
| STM1640_545_579 | 0.99 | -1.00 | 1253158lydcFlputative inner membrane proteinl- |
| STM1641_3295_3329 | 0.05 | 1.14 | 1253159lhrpAlATP-dependent RNA helicase HrpAl- |
| STM1642_31_65 | 0.02 | 1.23 | 1253160lacpDlazoreductasel+ |
| STM1643_33_67 | 0.07 | 1.35 | 1253161lSTM1643lputative inner membrane proteinl- |
| STM1644_185_219 | 0.04 | 1.23 | 1253162lydbLlputative periplasmic proteinl- |
| STM1645_81_115 | 0.00 | 1.43 | 1253163lynbElputative outer membrane lipoproteinl- |
| STM1646_1958_1992 | 0.05 | 1.72 | 1253164lydbHlhypothetical proteinl- |
| STM1647_497_531 | 0.04 | 1.47 | 1253165lldhAlD-lactate dehydrogenasel+ |
| STM1648_9_43 | 0.54 | 1.07 | 1253166lhslJlheat-inducible proteinl+ |
| STM1649_65_99 | 0.06 | -1.38 | 1253167lSTM1649lputative cytoplasmic proteinl- |
| STM1651_2910_2944 | 0.04 | 1.54 | 1253169lnifJlputative pyruvate-flavodoxin oxidoreductasel+ |
| STM1652_337_371 | 0.00 | -3.45 | 1253170lynaFlputative universal stress proteinl+ |
| STM1653_273_307 | 0.05 | 1.22 | 1253171lSTM1653lputative membrane transporter of cationsl- |
| STM1654_897_931 | 0.02 | -1.44 | 1253172lydaOlC32 tRNA thiolasel+ |
| STM1656_289_323 | 0.05 | -1.14 | 1253174lzntBlzinc transporterl- |
| STM1657_1068_1102 | 0.16 | -1.20 | 1253175lSTM1657lputative methyl-accepting chemotaxis proteinl- |
| STM1658_513_547 | 0.03 | 1.69 | 1253176lydaLlhypothetical proteinl- |
| STM1659_129_163 | 0.03 | 1.65 | 1253177logtlO-6-alkylguanine-DNA:cysteine-protein methyltransferasel+ |
| STM1660.S_577_611 | 0.11 | -1.20 | 1253178lfnrlfumarate/nitrate reduction transcriptional regulatorl+ |
| STM1661_897_931 | 0.01 | 1.64 | 1253179lydaAluniversal stress protein UspEl+ |
| STM1662_137_171 | 0.39 | -1.11 | 1253180lynaJlputative inner membrane proteinl- |
| STM1663_49_83 | 0.83 | -1.05 | 1253181lynaIlputative integral membrane proteinl+ |
| STM1664_513_547 | 0.36 | -1.08 | 1253182lSTM1664lputative transcriptional regulatorl- |
| STM1665_513_547 | 0.03 | 2.09 | 1253183lSTM1665lputative cytoplasmic proteinl+ |
| STM1666_161_195 | 0.01 | -1.37 | 1253184lSTM1666lpseudol+ |
| STM1667_105_139 | 0.04 | 1.89 | 1253185lSTM1667lputative thiol peroxidasel- |

| | | | |
|---|---|---|---|
| STM1668_145_179 | 0.82 | -1.03 | 1253186lSTM1668lhypothetical proteinl- |
| STM1670_273_307 | 0.09 | 1.53 | 1253188lSTM1670lputative lipoproteinl- |
| STM1671_291_325 | 0.02 | 2.38 | 1253189lSTM1671lputative regulatory proteinl+ |
| STM1672_564_598 | 0.03 | 1.70 | 2673745lSTM1672l-l+ |
| STM1673_65_99 | 0.16 | -1.14 | 1253191lSTM1673lputative outer membrane lipoproteinl- |
| STM1675_213_247 | 0.12 | 1.27 | 1253193lSTM1675loxidoreductasel+ |
| STM1676_417_451 | 0.03 | 1.37 | 1253194lSTM1676lputative aldo/keto reductasel- |
| STM1677_481_515 | 0.11 | 1.43 | 1253195lSTM1677lputative transcriptional regulatorl- |
| STM1678_369_403 | 0.07 | 2.18 | 1253196lSTM1678lputative 2'-hydroxyisoflavone reductasel+ |
| STM1679_671_705 | 0.15 | 1.23 | 1253197lmppAlperiplasmic murein tripeptide transport proteinl- |
| STM1680_193_227 | 0.78 | -1.05 | 1253198lycjIlmurein peptide amidase Al+ |
| STM1681_65_99 | 0.04 | -1.24 | 1253199lycjGlputative chloromuconate cycloisomerasel- |
| STM1682_21_55 | 0.62 | 1.04 | 1253200ltpxlthiol peroxidasel+ |
| STM1683_1119_1153 | 0.11 | 1.30 | 1253201ltyrRlDNA-binding transcriptional dual regulator  tyrosine-bindingl- |
| STM1685_469_503 | 0.02 | -2.04 | 1253203lycjXlputative ATPasel- |
| STM1686_33_67 | 0.00 | -3.00 | 1253204lpspElthiosulfate:cyanide sulfurtransferase (rhodanese)l- |
| STM1687_37_71 | 0.57 | 1.06 | 1253205lpspDlperipheral inner membrane phage-shock proteinl- |
| STM1688_81_115 | 0.45 | -1.16 | 1253206lpspClDNA-binding transcriptional activatorl- |
| STM1689_125_159 | 0.06 | 1.38 | 1253207lpspBlphage shock protein Bl- |
| STM1690_15_49 | 0.13 | 1.34 | 1253208lpspAlphage shock proteinl- |
| STM1691_897_931 | 0.08 | 1.34 | 1253209lpspFlphage shock protein operon transcriptional activatorl+ |
| STM1692_907_941 | 0.03 | 1.33 | 1253210lsapAlpeptide transport proteinl+ |
| STM1693_801_835 | 0.02 | -1.73 | 1253211lsapBlpeptide transport proteinl+ |
| STM1694_769_803 | 0.20 | -1.11 | 1253212lsapClpeptide transport proteinl+ |
| STM1695_385_419 | 0.64 | -1.08 | 1253213lsapDlpeptide transport proteinl+ |
| STM1696_417_451 | 0.03 | -1.40 | 1253214lsapFlpeptide transport proteinl+ |
| STM1697_513_547 | 0.04 | -1.41 | 1253215lSTM1697lhypothetical proteinl- |
| STM1699_89_123 | 0.01 | 2.27 | 1253218lycjElputative cytoplasmic proteinl+ |
| STM1700_585_619 | 0.12 | 1.52 | 1253219lfabIlenoyl-(acyl carrier protein) reductasel+ |
| STM1701_465_499 | 0.23 | -2.33 | 1253220lyciWlputative cytoplasmic proteinl+ |
| STM1702_1016_1050 | 0.10 | -1.49 | 1253221lrnblexoribonuclease IIl+ |
| STM1704_33_67 | 0.16 | -1.22 | 1253223lyciTlputative regulatory proteinl+ |
| STM1705_133_167 | 0.11 | 1.83 | 1253224losmBllipoproteinl+ |
| STM1706_121_155 | 0.03 | 1.97 | 1253225lyciHltranslation initiation factor Sui1l- |
| STM1707_641_675 | 0.91 | -1.02 | 1253226lpyrFlOMP decarboxylase; OMPDCase; OMPdecasel- |
| STM1708_1131_1165 | 0.03 | 1.99 | 1253227lyciMlhypothetical proteinl- |

| | | | |
|---|---|---|---|
| STM1709_61_95 | 0.01 | -1.12 | 1253228|yciS|putative inner membrane protein|- |
| STM1710_41_75 | 0.18 | 1.24 | 1253229|pgpB|phosphatidylglycerophosphatase B|- |
| STM1711_481_515 | 0.43 | -1.14 | 1253230|ribA|GTP cyclohydrolase II|+ |
| STM1712_1885_1919 | 0.02 | -2.00 | 1253231|acnA|aconitate hydratase|- |
| STM1713_641_675 | 0.77 | 1.09 | 1253232|cysB|transcriptional regulator CysB|- |
| STM1714_1599_1633 | 0.12 | -1.41 | 1253233|topA|DNA topoisomerase I|- |
| STM1715_5_39 | 0.99 | 1.00 | 1253234|yciN|hypothetical protein|+ |
| STM1716_880_914 | 0.08 | 1.12 | 1253235|sohB|predicted inner membrane peptidase|- |
| STM1717_545_579 | 0.24 | -1.26 | 1253236|yciK|short chain dehydrogenase|+ |
| STM1718_489_523 | 0.00 | -3.21 | 1253237|btuR|cob(I)yrinic acid a c-diamide adenosyltransferase|+ |
| STM1719_385_419 | 0.10 | -1.32 | 1253238|yciL|23S rRNA pseudouridylate synthase B|- |
| STM1720_529_563 | 0.04 | 1.44 | 1253239|yciO|hypothetical protein|- |
| STM1721_529_563 | 0.67 | 1.09 | 1253240|trpH|hypothetical protein|- |
| STM1722_9_43 | 0.21 | -1.28 | 1253241|trpL|trp operon leader peptide|+ |
| STM1723_1468_1502 | 0.18 | -1.24 | 1253242|trpE|anthranilate synthase component I|+ |
| STM1724_853_887 | 0.06 | 1.18 | 1253243|trpD|bifunctional indole-3-glycerol-phosphate synthase/anthranilate phosphoribosyltransferase|+ |
| STM1725_360_394 | 0.14 | 1.19 | 1253244|trpC|bifunctional indole-3-glycerol phosphate synthase/phosphoribosylanthranilate isomerase|+ |
| STM1726_387_421 | 0.74 | -1.07 | 1253245|trpB|tryptophan synthase subunit beta|+ |
| STM1727_289_323 | 0.53 | -1.12 | 1253246|trpA|tryptophan synthase subunit alpha|+ |
| STM1728_9_43 | 0.02 | 2.76 | 1253247|yciG|putative cytoplasmic protein|+ |
| STM1729_151_185 | 0.02 | 1.78 | 1253248|yciF|putative cytoplasmic protein|+ |
| STM1730_65_99 | 0.03 | 2.10 | 1253249|yciE|putative cytoplasmic protein|+ |
| STM1731_385_419 | 0.36 | 1.13 | 1253250|STM1731|putative catalase|+ |
| STM1732_161_195 | 0.00 | -9.02 | 1253251|ompW|outer membrane protein W|- |
| STM1733_225_259 | 0.24 | 1.36 | 1253252|STM1733|putative ferredoxin|+ |
| STM1734_577_611 | 0.08 | 1.20 | 1253253|yciC|hypothetical protein|+ |
| STM1735_385_419 | 0.04 | 1.34 | 1253254|yciB|intracellular septation protein A|+ |
| STM1736_101_135 | 0.71 | 1.09 | 1253255|yciA|predicted hydrolase|+ |
| STM1737_49_83 | 0.07 | -1.51 | 1253256|tonB|membrane spanning protein in TonB-ExbB-ExbD complex; transduces proton motive force of the cytoplasmic membrane to outer membrane transporters; involved in the transport of ron-siderophore complexes vitamin B12 and colicins|- |
| STM1738_190_224 | 0.05 | -1.29 | 1253257|yciI|YciI-like protein|+ |
| STM1739_590_624 | 0.11 | -1.19 | 1253258|cls|cardiolipin synthetase|+ |

| | | | |
|---|---|---|---|
| STM1740_308_342 | 0.16 | -1.27 | 2673771lyciUl-l+ |
| STM1741_545_579 | 0.51 | -1.03 | 1253260lSTM1741lputative voltage-gated potassium channell- |
| STM1742_710_744 | 0.01 | -2.48 | 1253261loppFloligopeptide transport proteinl- |
| STM1743_777_811 | 0.00 | -3.08 | 1253262loppDloligopeptide transporter ATP-binding componentl- |
| STM1744_625_659 | 0.01 | -2.10 | 1253263loppCloligopeptide transport proteinl- |
| STM1745_641_675 | 0.01 | -2.61 | 1253264loppBloligopeptide transporter permeasel- |
| STM1746.S_745_779 | 0.00 | -3.83 | 1253265loppAloligopeptide transport proteinl- |
| STM1748_259_293 | 0.08 | 2.53 | 1253267lychElhypothetical proteinl- |
| STM1749_1680_1714 | 0.00 | -6.81 | 1253268ladhEliron-dependent alcohol dehydrogenasel+ |
| STM1750_17_51 | 0.01 | -1.37 | 1253269ltdklthymidine kinasel- |
| STM1751_17_51 | 0.14 | -1.39 | 1253270lhnslglobal DNA-binding transcriptional dual regulator H-NSl+ |
| STM1752_641_675 | 0.79 | -1.01 | 1253271lgalUlUTP--glucose-1-phosphate uridylyltransferase subunit GalUl- |
| STM1753_911_945 | 0.31 | 1.11 | 1253272lhnrlresponse regulator of RpoSl- |
| STM1754_641_675 | 0.11 | 1.21 | 1253273lychKlhypothetical proteinl- |
| STM1755_65_99 | 0.12 | 1.17 | 1253274lychJlhypothetical proteinl+ |
| STM1756_385_419 | 0.77 | -1.04 | 1253275lpurUlformyltetrahydrofolate deformylasel+ |
| STM1758_49_83 | 0.67 | -1.07 | 1253277lrtTlmisc_RNAl+ |
| STM1760_563_597 | 0.13 | -1.30 | 1253279lSTM1760ltetratricopeptide repeat proteinl- |
| STM1761_641_675 | 0.95 | -1.01 | 1253280lnarIlnitrate reductase 1 gamma subunitl- |
| STM1762_337_371 | 0.85 | -1.02 | 1253281lnarJlnitrate reductase 1 delta subunitl- |
| STM1763_1121_1155 | 0.02 | -1.46 | 1253282lnarHlnitrate reductase 1 beta subunitl- |
| STM1764_3689_3723 | 0.08 | 1.85 | 1253283lnarGlnitrate reductase 1 alpha subunitl- |
| STM1766_830_864 | 0.03 | -1.30 | 1253285lnarXlnitrate/nitrite sensor protein NarXl+ |
| STM1767_241_275 | 0.04 | -1.44 | 1253286lnarLlDNA-binding response regulator in two-component regulatory system with NarX (or NarQ)l+ |
| STM1768_1018_1052 | 0.91 | -1.01 | 1253287lychPlpredicted invasinl- |
| STM1769_275_309 | 0.54 | 1.07 | 1253288lychNlputative sulfur reduction proteinl+ |
| STM1770_3_40 | 0.07 | -1.25 | 1253289lchaBlcation transport regulatorl- |
| STM1771_1006_1040 | 0.10 | 1.55 | 1253290lchaAlcalcium/sodium:proton antiporterl+ |
| STM1772_97_131 | 0.37 | -1.18 | 1253291lkdsAl2-dehydro-3-deoxyphosphooctonate aldolasel- |
| STM1773_641_675 | 0.03 | 1.65 | 1253292lychAlputative transcriptional regulatorl- |
| STM1774_129_163 | 0.97 | 1.02 | 1253293lsirClputative transcriptional regulatorl- |
| STM1775_481_515 | 0.05 | -1.39 | 1253294lhemKlN5-glutamine S-adenosyl-L-methionine-dependent methyltransferasel- |
| STM1776_980_1014 | 0.03 | -1.43 | 1253295lprfAlpeptide chain release factor 1l- |
| STM1777_1058_1092 | 0.05 | -1.33 | 1253296lhemAlglutamyl-tRNA reductasel- |

| | | | |
|---|---|---|---|
| STM1778_17_51 | 0.24 | 1.10 | 1253297\|lolB\|outer membrane lipoprotein LolB\|+ |
| STM1779_265_299 | 0.10 | -1.39 | 1253298\|ipk\|4-diphosphocytidyl-2-C-methyl-D-erythritol kinase\|+ |
| STM1780_897_931 | 0.06 | -1.69 | 1253299\|prsA\|ribose-phosphate pyrophosphokinase\|+ |
| STM1781_1511_1545 | 0.05 | -2.12 | 1253300\|ychM\|putative sulfate transporter YchM\|+ |
| STM1782_201_235 | 0.00 | -9.98 | 1253301\|ychH\|predicted inner membrane protein\|- |
| STM1783.S_385_419 | 0.04 | 2.09 | 1253302\|pth\|peptidyl-tRNA hydrolase\|+ |
| STM1784_861_895 | 0.79 | -1.03 | 1253303\|ychF\|translation-associated GTPase\|+ |
| STM1785_779_813 | 0.02 | 1.77 | 1253304\|STM1785\|putative cytoplasmic protein\|- |
| STM1787_795_829 | 0.91 | -1.04 | 1253306\|STM1787\|hydrogenase 1 large subunit\|+ |
| STM1788_545_579 | 0.12 | -1.23 | 1253307\|STM1788\|hydrogenase 1 b-type cytochrome subunit\|+ |
| STM1789_377_411 | 0.04 | -1.38 | 1253308\|STM1789\|hydrogenase 1 maturation protease\|+ |
| STM1791_65_99 | 0.27 | 1.41 | 1253310\|STM1791\|putative hydrogenase-1 protein\|+ |
| STM1792_594_628 | 0.02 | 1.24 | 1253311\|STM1792\|putative cytochrome oxidase subunit II\|+ |
| STM1793_522_556 | 0.79 | -1.02 | 1253312\|STM1793\|putative cytochrome oxidase subunit III\|+ |
| STM1794_22_56 | 0.07 | 1.47 | 1253313\|STM1794\|putative periplasmic protein\|+ |
| STM1795_1111_1145 | 0.00 | -8.53 | 1253314\|STM1795\|putative glutamic dehyrogenase-like protein\|+ |
| STM1796_1386_1420 | 0.54 | 1.09 | 1253315\|treA\|trehalase\|+ |
| STM1797_145_179 | 0.20 | 1.35 | 1253316\|ymgE\|putative transglycosylase-associated protein\|- |
| STM1798_249_283 | 0.94 | -1.02 | 1253317\|ycgR\|putative inner membrane protein\|+ |
| STM1799_209_243 | 0.12 | 1.14 | 1253318\|emtA\|membrane-bound lytic murein transglycosylase E\|- |
| STM1800_289_323 | 0.14 | 1.12 | 1253319\|ldcA\|L D-carboxypeptidase A\|+ |
| STM1801_1031_1065 | 0.02 | -1.40 | 1253320\|ycgO\|potassium/proton antiporter\|+ |
| STM1803_940_974 | 0.00 | -12.53 | 1253322\|dadA\|D-amino acid dehydrogenase small subunit\|- |
| STM1804.S_1232_1266 | 0.51 | 1.14 | 1253323\|ycgB\|hypothetical protein\|+ |
| STM1805_513_547 | 0.01 | -1.46 | 1253324\|fadR\|fatty acid metabolism regulator\|- |
| STM1806_1442_1476 | 0.01 | -1.26 | 1253325\|nhaB\|sodium/proton antiporter\|+ |
| STM1807_209_243 | 0.44 | -1.10 | 1253326\|dsbB\|disulfide bond formation protein B\|+ |
| STM1808_137_171 | 0.03 | 2.11 | 1253327\|STM1808\|putative cytoplasmic protein\|+ |
| STM1809_119_154 | 0.01 | 1.93 | 1253328\|STM1809\|putative cytoplasmic protein\|- |
| STM1810_6_41 | 0.02 | 2.51 | 1253329\|STM1810\|putative cytoplasmic protein\|- |
| STM1811_129_163 | 0.01 | 1.67 | 1253330\|ycgN\|hypothetical protein\|- |
| STM1812_257_291 | 0.12 | 1.37 | 1253331\|ycgM\|predicted isomerase/hydrolase\|- |
| STM1813_45_81 | 0.02 | 1.88 | 1253332\|ycgL\|putative cytoplasmic protein\|- |
| STM1814_177_211 | 0.03 | -1.67 | 1253333\|minC\|septum formation inhibitor\|+ |
| STM1815_705_739 | 0.02 | -2.17 | 1253334\|minD\|cell division inhibitor MinD\|+ |
| STM1816_161_195 | 0.78 | 1.01 | 1253335\|minE\|cell division topological specificity factor MinE\|+ |

| | | | |
|---|---|---|---|
| STM1817_897_931 | 0.03 | 1.45 | 1253336\|rnd\|ribonuclease D\|- |
| STM1818_879_913 | 0.00 | -6.82 | 1253337\|fadD\|long-chain-fatty-acid--CoA ligase\|- |
| STM1819_65_99 | 0.03 | -1.33 | 1253338\|slp\|putative outer membrane protein\|- |
| STM1820_198_232 | 0.01 | -2.14 | 1253339\|yeaZ\|putative molecular chaperone\|- |
| STM1821_1072_1106 | 0.07 | -1.16 | 1253340\|yoaA\|putative DNA helicase\|- |
| STM1822_17_51 | 0.09 | -1.40 | 1253341\|yoaB\|putative translation initiation inhibitor\|+ |
| STM1823_65_99 | 0.07 | 1.38 | 1253342\|yoaH\|hypothetical protein\|- |
| STM1824_558_592 | 0.09 | -1.32 | 1253343\|pabB\|para-aminobenzoate synthase component I\|+ |
| STM1825_49_83 | 0.21 | -1.37 | 1253344\|yeaB\|hypothetical protein\|+ |
| STM1826_910_944 | 0.05 | 1.26 | 1253345\|sdaA\|L-serine deaminase I/L-threonine deaminase I\|+ |
| STM1827.S_739_773 | 0.41 | 1.13 | 1253346\|STM1827.S\|putative diguanylate cyclase/phosphodiesterase\|+ |
| STM1828_1358_1392 | 0.07 | -1.41 | 1253347\|yoaE\|putative inner membrane protein\|- |
| STM1829_157_191 | 0.10 | 2.44 | 1253348\|STM1829\|putative cytoplasmic protein\|- |
| STM1830_257_291 | 0.01 | -2.83 | 1253349\|manX\|mannose-specific enzyme IIAB\|+ |
| STM1831_321_355 | 0.01 | -2.46 | 1253350\|manY\|mannose-specific enzyme IIC\|+ |
| STM1832_481_515 | 0.01 | -2.73 | 1253351\|manZ\|mannose-specific PTS system protein IID\|+ |
| STM1833_353_387 | 0.12 | 1.69 | 1253352\|STM1833\|hypothetical protein\|+ |
| STM1834_289_323 | 0.05 | -1.36 | 1253353\|yebN\|hypothetical protein\|+ |
| STM1835_433_467 | 0.02 | -1.80 | 1253354\|rrmA\|23S rRNA methyltransferase A\|- |
| STM1836_875_909 | 0.01 | 1.15 | 1253355\|STM1836\|putative penicillin-binding protein 3\|- |
| STM1837_145_179 | 0.12 | -1.53 | 1253356\|cspC\|cold shock-like protein cspC\|- |
| STM1838_25_59 | 0.01 | -2.16 | 1253357\|yobF\|putative cytoplasmic protein\|- |
| STM1839_123_160 | 0.01 | 3.78 | 1253358\|STM1839\|hypothetical protein\|- |
| STM1840_57_91 | 0.01 | 1.70 | 1253359\|yobG\|hypothetical protein\|- |
| STM1841_153_187 | 0.02 | 2.96 | 1253360\|STM1841\|hypothetical protein\|+ |
| STM1842_385_419 | 0.38 | 1.13 | 1253361\|kdgR\|putative transcriptional repressor\|- |
| STM1843_967_1001 | 0.04 | -1.30 | 1253362\|STM1843\|putative transport protein\|+ |
| STM1844_641_675 | 0.27 | 1.38 | 1253363\|htpX\|heat shock protein HtpX\|- |
| STM1845_1242_1276 | 0.03 | -1.66 | 1253364\|prc\|carboxy-terminal protease\|- |
| STM1846_624_658 | 0.01 | -2.17 | 1253365\|proQ\|putative solute/DNA competence effector\|- |
| STM1847_173_207 | 0.04 | -1.23 | 1253366\|yebR\|putative nucleotide-binding protein\|- |
| STM1848_493_527 | 0.29 | -1.17 | 1253367\|yebS\|putative inner membrane protein\|+ |
| STM1849_2281_2315 | 0.77 | 1.23 | 1253368\|STM1849\|putative inner membrane protein\|+ |
| STM1850_1097_1131 | 0.47 | -1.13 | 1253369\|yebU\|rRNA (cytosine-C(5)-)-methyltransferase RsmF\|+ |
| STM1851_30_65 | 0.03 | 2.36 | 1253370\|STM1851\|putative cytoplasmic protein\|+ |
| STM1852_89_123 | 0.34 | -1.19 | 1253371\|yebW\|putative inner membrane lipoprotein\|+ |

| | | | |
|---|---|---|---|
| STM1853_417_451 | 0.13 | 2.01 | 1253372lpphAlserine/threonine protein phosphatase 1l- |
| STM1854_77_111 | 0.01 | 2.55 | 1253373lSTM1854lputative inner membrane proteinl- |
| STM1856.1N_321_355 | 0.03 | 2.75 | 2673765lSTM1856.1Nlhypothetical proteinl+ |
| STM1857_129_163 | 0.00 | -4.54 | 1253376lSTM1857lputative acetyltransferasel- |
| STM1859_81_115 | 0.01 | 1.37 | 1253378lSTM1859lputative cytoplasmic proteinl+ |
| STM1861_73_107 | 0.02 | 2.76 | 1253380lSTM1861l-l+ |
| STM1862_65_99 | 0.01 | 3.18 | 1253381lpagOlintegral membrane proteinl+ |
| STM1863_73_107 | 0.00 | 2.61 | 1253382lSTM1863lputative inner membrane proteinl+ |
| STM1864_225_259 | 0.00 | 2.93 | 1253383lSTM1864lputative inner membrane proteinl+ |
| STM1865_37_71 | 0.01 | 5.28 | 1253384lSTM1865l-l- |
| STM1866_119_153 | 0.02 | 3.63 | 1253385lSTM1866lpseudol- |
| STM1867_44_82 | 0.03 | 4.40 | 1253386lpagKlPagKl+ |
| STM1868_553_587 | 0.02 | 2.54 | 1253387lmig-3lphage-tail assembly-like proteinl- |
| STM1868.1N_38_72 | 0.05 | 2.55 | 2673772lSTM1868.1Nlhypothetical proteinl- |
| STM1868A_75_109 | 0.79 | -1.06 | 1253388lSTM1868Allytic enzymel+ |
| STM1869_385_419 | 0.07 | 1.21 | 1253389lSTM1869lphage-tail assembly-like proteinl- |
| STM1869A_17_51 | 0.25 | 1.15 | 1253390lSTM1869Alhypothetical proteinl- |
| STM1870_217_251 | 0.02 | 2.53 | 1253391lSTM1870lRecE-like proteinl+ |
| STM1871_593_627 | 0.03 | 1.30 | 1253392lSTM1871l-l+ |
| STM1872_51_85 | 0.10 | 1.26 | 1253393lSTM1872lputative cytoplasmic proteinl- |
| STM1873_241_275 | 0.02 | 1.35 | 1253394lSTM1873lhypothetical proteinl- |
| STM1874_641_675 | 0.04 | 1.62 | 1253395lSTM1874lputative inner membrane proteinl- |
| STM1875_289_323 | 0.02 | 1.81 | 1253396lyobAlhypothetical proteinl- |
| STM1876_179_213 | 0.54 | -1.07 | 1253397lholElDNA polymerase III subunit thetal+ |
| STM1877_617_651 | 0.35 | -1.18 | 1253398lSTM1877lputative amidohydrolasel+ |
| STM1878_257_291 | 0.08 | -1.24 | 1253399lexoXlexodeoxyribonuclease Xl+ |
| STM1879_1341_1375 | 0.02 | 1.51 | 1253400lptrBlprotease 2l- |
| STM1880_121_155 | 0.01 | 2.22 | 1253401lyebElputative inner membrane proteinl- |
| STM1881_49_83 | 0.01 | 1.63 | 1253402lyebFlputative periplasmic proteinl- |
| STM1882_90_124 | 0.01 | 2.43 | 1253403lyebGlDNA damage-inducible protein YebGl- |
| STM1884_497_531 | 0.40 | 1.09 | 1253405ledalketo-hydroxyglutarate-aldolase/keto-deoxy-phosphogluconate aldolasel- |
| STM1885_1517_1551 | 0.76 | 1.04 | 1253406leddlphosphogluconate dehydratasel- |
| STM1886_1213_1247 | 0.48 | 1.10 | 1253407lzwflglucose-6-phosphate 1-dehydrogenasel- |
| STM1887_513_547 | 0.04 | 1.46 | 1253408lyebKlDNA-binding transcriptional regulator HexRl+ |
| STM1888_1340_1374 | 0.09 | -1.53 | 1253409lpykAlpyruvate kinasel+ |

| | | | |
|---|---|---|---|
| STM1889_545_579 | 0.07 | -1.25 | 1253410lmsbBllipid A biosynthesis (KDO)2-(lauroyl)-lipid IVA acyltransferasel- |
| STM1890_1025_1059 | 0.00 | -2.87 | 1253411lyebAlhypothetical proteinl- |
| STM1891_81_115 | 0.02 | -10.53 | 1253412lznuAlhigh-affinity zinc transporter periplasmic componentl- |
| STM1892.S_225_259 | 0.02 | -2.25 | 1253413lznuClhigh-affinity zinc transporter ATPasel+ |
| STM1893_513_547 | 0.04 | -3.65 | 1253414lznuBlhigh-affinity zinc transporter membrane componentl+ |
| STM1895_523_557 | 0.07 | 1.37 | 1253416lruvAlHolliday junction DNA helicase motor proteinl- |
| STM1896_410_444 | 0.03 | 2.62 | 1253417lSTM1896lputative cytoplasmic proteinl+ |
| STM1897_397_431 | 0.37 | 1.14 | 1253418lyebBlhypothetical proteinl+ |
| STM1898_105_139 | 0.04 | -1.21 | 1253419lruvClHolliday junction resolvasel- |
| STM1900_209_243 | 0.02 | -1.56 | 1253421lntpAldATP pyrophosphohydrolasel- |
| STM1901_854_888 | 0.01 | -2.41 | 1253422laspSlaspartyl-tRNA synthetasel- |
| STM1902_385_419 | 0.01 | 2.44 | 1253423lyecDlhypothetical proteinl+ |
| STM1903_753_787 | 0.01 | 1.79 | 1253424lyecElhypothetical proteinl+ |
| STM1904_209_243 | 0.09 | -1.07 | 1253425lyecNlputative inner membrane proteinl+ |
| STM1905_513_547 | 0.16 | -1.35 | 1253426lyecOlputative SAM-dependent methyltransferasel+ |
| STM1906_641_675 | 0.80 | -1.04 | 1253427lyecPlputative enzymel+ |
| STM1907_137_171 | 0.01 | 1.46 | 1253428lcutClcopper homeostasis protein CutCl- |
| STM1908_97_131 | 0.02 | -1.65 | 1253429lyecMlhypothetical proteinl- |
| STM1909_991_1025 | 0.57 | -1.14 | 1253430largSlarginyl-tRNA synthetasel+ |
| STM1910_1225_1259 | 0.05 | 1.98 | 1253431lSTM1910lputative penicillin-binding proteinl+ |
| STM1911_845_879 | 0.69 | 1.03 | 1253432lSTM1911lputative cytoplasmic proteinl- |
| STM1912_357_391 | 0.90 | 1.01 | 1253433lflhElflagellar proteinl- |
| STM1913_1208_1242 | 0.71 | -1.11 | 1253434lflhAlflagellar biosynthesis protein Al- |
| STM1914_793_827 | 0.08 | 2.17 | 1253435lflhBlflagellar biosynthesis protein FlhBl- |
| STM1915_353_387 | 0.08 | -1.47 | 1253436lcheZlchemotaxis regulator CheZl- |
| STM1916_317_351 | 0.05 | -1.35 | 1253437lcheYlchemotaxis regulator transmitting signal to flagellar motor componentl- |
| STM1917_539_573 | 0.02 | -1.45 | 1253438lcheBlchemotaxis-specific methylesterasel- |
| STM1919_663_697 | 0.02 | -1.77 | 1253440lcheMlmethyl accepting chemotaxis protein IIl- |
| STM1920_225_259 | 0.01 | -2.39 | 1253441lcheWlpurine-binding chemotaxis proteinl- |
| STM1921_1401_1435 | 0.01 | -2.16 | 1253442lcheAlchemotaxis protein CheAl- |
| STM1922_537_571 | 0.02 | -1.86 | 1253443lmotBlflagellar motor protein MotBl- |
| STM1923_21_55 | 0.15 | -1.33 | 1253444lmotAlflagellar motor protein MotAl- |
| STM1924.S_41_75 | 0.00 | -3.13 | 1253445lflhClranscriptional activator FlhCl- |
| STM1925_209_243 | 0.00 | -3.34 | 1253446lflhDltranscriptional activator FlhDl- |
| STM1927_97_131 | 0.03 | -1.80 | 1253448lyecGluniversal stress protein UspCl+ |

| | | | |
|---|---|---|---|
| STM1928_903_937 | 0.45 | -1.13 | 1253449lotsAltrehalose-6-phosphate synthasel- |
| STM1929_257_291 | 0.18 | 1.38 | 1253450lotsBltrehalose-6-phosphate phosphatasel- |
| STM1930_81_115 | 0.41 | -1.26 | 1253451lSTM1930lpseudol- |
| STM1932_457_491 | 0.00 | 2.09 | 1253453lftnBlferritin-like proteinl+ |
| STM1933_153_187 | 0.28 | -1.17 | 1253454lSTM1933lhypothetical proteinl- |
| STM1934_225_259 | 0.06 | 1.26 | 1253455lSTM1934lputative outer membrane lipoproteinl+ |
| STM1935_161_195 | 0.06 | 1.51 | 1253456lftnlferritinl+ |
| STM1936_102_137 | 0.09 | -1.36 | 1253457lyecHlputative cytoplasmic proteinl- |
| STM1937_1149_1183 | 0.55 | -1.11 | 1253458ltyrPltyrosine-specific transport proteinl+ |
| STM1938_97_131 | 0.00 | -2.98 | 1253459lyecAlhypothetical proteinl- |
| STM1939_26_60 | 0.02 | 3.40 | 1253460lSTM1939lputative glucose-6-phosphate dehydrogenasel- |
| STM1940_1049_1083 | 0.02 | 1.48 | 1253461lSTM1940lputative cell wall-associated hydrolasel- |
| STM1941_141_175 | 0.00 | 2.33 | 1253462lSTM1941lputative inner membrane proteinl- |
| STM1942_5_40 | 0.01 | -2.11 | 1253463lleuZltRNAl- |
| STM1943_5_39 | 0.02 | -2.12 | 1253464lcysTltRNAl- |
| STM1945_417_451 | 0.70 | -1.03 | 1253466lpgsAlphosphatidylglycerophosphate synthetasel- |
| STM1946_1090_1124 | 0.14 | -1.14 | 1253467luvrClexcinuclease ABC subunit Cl- |
| STM1947_337_371 | 0.13 | 1.07 | 1253468luvrYlresponse regulatorl- |
| STM1949_35_70 | 0.02 | 1.53 | 1253470lyecFlhypothetical proteinl+ |
| STM1950_81_115 | 0.20 | -1.24 | 1253471lsdiAlDNA-binding transcriptional activatorl- |
| STM1951_529_563 | 0.48 | -1.14 | 1253472lyecClputative amino-acid ABC transporter ATP-binding protein YecCl- |
| STM1952_33_67 | 0.34 | -1.12 | 1253473lyecSlputative ABC-type amino acid transporter permease |
| STM1953_65_99 | 0.76 | -1.05 | 1253474lyedOlD-cysteine desulfhydrasel- |
| STM1954_357_391 | 0.09 | 2.04 | 1253475lfliYlcystine transporter subunitl- |
| STM1955_337_371 | 0.70 | 1.02 | 1253476lfliZlprotein FliZl- |
| STM1956_161_195 | 0.07 | -1.18 | 1253477lfliAlflagellar biosynthesis sigma factorl- |
| STM1957_251_285 | 0.03 | -1.43 | 1253478ltnpA_2ltransposase for IS200l- |
| STM1958_847_881 | 0.51 | -1.10 | 1253479lfliBllysine-N-methylasel- |
| STM1959_545_579 | 0.03 | -2.40 | 1253480lfliClflagellinl- |
| STM1960_949_983 | 0.01 | -2.49 | 1253481lfliDlflagellar capping proteinl+ |
| STM1961_273_307 | 0.01 | -1.67 | 1253482lfliSlflagellar protein FliSl+ |
| STM1962_65_99 | 0.04 | -1.54 | 1253483lfliTlflagellar biosynthesis protein FliTl+ |
| STM1963_1154_1188 | 0.53 | -1.02 | 1253484lamyAlcytoplasmic alpha-amylasel+ |
| STM1964_241_275 | 0.27 | 1.25 | 1253485lyedDlhypothetical proteinl- |
| STM1965_555_589 | 0.01 | -1.72 | 1253486lyedElpredicted inner membrane proteinl+ |
| STM1966_161_195 | 0.28 | -1.13 | 1253487lyedFlhypothetical proteinl+ |

| | | | |
|---|---|---|---|
| STM1968_279_313 | 0.01 | -1.45 | 1253489\|fliE\|flagellar hook-basal body protein FliE\|- |
| STM1969_748_782 | 0.11 | 1.16 | 1253490\|fliF\|flagellar MS-ring protein\|+ |
| STM1970_97_131 | 0.86 | -1.01 | 1253491\|fliG\|flagellar motor switch protein G\|+ |
| STM1971_69_103 | 0.37 | -1.10 | 1253492\|fliH\|flagellar assembly protein H\|+ |
| STM1974_283_317 | 0.12 | -1.21 | 1253495\|fliK\|flagellar hook-length control protein\|+ |
| STM1975_177_211 | 0.02 | 1.64 | 1253496\|fliL\|flagellar basal body-associated protein FliL\|+ |
| STM1977_281_315 | 0.23 | 1.12 | 1253498\|fliN\|flagellar motor switch protein FliN\|+ |
| STM1978_209_243 | 0.03 | 1.32 | 1253499\|fliO\|flagellar biosynthesis protein FliO\|+ |
| STM1979_321_355 | 0.00 | 1.62 | 1253500\|fliP\|flagellar biosynthesis protein FliP\|+ |
| STM1980_137_171 | 0.02 | 3.35 | 1253501\|fliQ\|flagellar biosynthesis protein FliQ\|+ |
| STM1981_241_275 | 0.70 | 1.03 | 1253502\|fliR\|flagellar biosynthesis protein FliR\|+ |
| STM1982_313_347 | 0.01 | 2.26 | 1253503\|rcsA\|colanic acid capsular biosynthesis activation protein A\|+ |
| STM1983_123_157 | 0.04 | 1.78 | 1253504\|dsrB\|hypothetical protein\|- |
| STM1984_129_163 | 0.91 | -1.07 | 1253505\|yodD\|putative cytoplasmic protein\|+ |
| STM1986_209_243 | 0.02 | 1.53 | 1253507\|yedP\|mannosyl-3-phosphoglycerate phosphatase\|+ |
| STM1987_746_780 | 0.39 | -1.10 | 1253508\|STM1987\|putative inner membrane protein\|- |
| STM1988.S_89_123 | 0.25 | -1.29 | 1253509\|STM1988.S\|putative cytoplasmic protein\|- |
| STM1989_641_675 | 0.04 | -1.69 | 1253510\|yedI\|hypothetical protein\|- |
| STM1990_641_675 | 0.27 | -1.11 | 1253511\|yedA\|hypothetical protein\|+ |
| STM1991_113_147 | 0.06 | 1.19 | 1253512\|vsr\|DNA mismatch endonuclease\|- |
| STM1992_1264_1298 | 0.03 | -1.37 | 1253513\|dcm\|DNA cytosine methylase\|- |
| STM1993_481_515 | 0.02 | -1.54 | 1253514\|yedJ\|hypothetical protein\|- |
| STM1994_263_297 | 0.39 | 1.30 | 1253515\|STM1994\|putative inner membrane protein\|- |
| STM1995_558_592 | 0.28 | 1.20 | 1253516\|ompS\|putative porin\|+ |
| STM1996_178_212 | 0.07 | 1.67 | 1253517\|cspB\|putative cold-shock protein\|- |
| STM1997_398_432 | 0.00 | 1.44 | 1253518\|umuC\|DNA polymerase V subunit UmuC\|- |
| STM1998_225_259 | 0.02 | 1.87 | 1253519\|umuD\|DNA polymerase V subunit UmuD\|- |
| STM1999_106_140 | 0.05 | 1.88 | 1253520\|STM1999\|putative cytoplasmic protein\|- |
| STM2000_31_65 | 0.05 | 1.39 | 1253521\|serU\|tRNA\|- |
| STM2001_402_437 | 0.00 | -4.61 | 1253522\|yeeI\|putative inner membrane protein\|+ |
| STM2003_57_91 | 0.27 | 1.28 | 1253524\|STM2003\|pseudo\|+ |
| STM2006_685_719 | 0.37 | 1.06 | 2673718\|STM2006\|-\|+ |
| STM2007_1048_1082 | 0.43 | -1.05 | 1253528\|STM2007\|tetratricopeptide repeat protein\|+ |
| STM2008_1324_1358 | 0.09 | 1.68 | 1253529\|STM2008\|putative periplasmic protein\|+ |
| STM2011_17_51 | 0.01 | -1.16 | 1253532\|STM2011\|putative cytoplasmic protein\|- |
| STM2011.1n_133_167 | 0.15 | 1.82 | 2673719\|STM2011.1n\|hypothetical protein\|+ |

| | | | |
|---|---|---|---|
| STM2013_971_1005 | 0.13 | -1.17 | 1253534lyeeOlpredicted multidrug efflux systeml- |
| STM2015_705_739 | 0.03 | 1.78 | 1253536lerfKlhypothetical proteinl- |
| STM2016_840_874 | 0.03 | -1.37 | 1253537lcobTlnicotinate-nucleotide--dimethylbenzimidazole phosphoribosyltransferasel- |
| STM2018_449_483 | 0.05 | -1.28 | 1253539lcobUladenosylcobinamide kinase/adenosylcobinamide-phosphate guanylyltransferasel- |
| STM2019_1418_1452 | 0.01 | -2.01 | 1253540lcbiPlcobyric acid synthasel- |
| STM2020_17_51 | 0.05 | -1.44 | 1253541lcbiOlvitamin B12 biosynthetic proteinl- |
| STM2021_329_363 | 0.01 | -2.71 | 1253542lcbiQlvitamin B12 biosynthetic proteinl- |
| STM2022_61_95 | 0.01 | -2.95 | 1253543lcbiNlcobalt transport protein CbiNl- |
| STM2024_289_323 | 0.00 | -2.70 | 1253545lcbiLlcobalt-precorrin-2 C(20)-methyltransferasel- |
| STM2025_289_323 | 0.54 | -1.27 | 1253546lcbiKlvitamin B12 biosynthetic proteinl- |
| STM2026_193_227 | 0.06 | -1.74 | 1253547lcbiJlcobalt-precorrin-6x reductasel- |
| STM2027_685_719 | 0.00 | -2.62 | 1253548lcbiHlprecorrin-3B C17-methyltransferasel- |
| STM2028_1021_1055 | 0.00 | -3.78 | 1253549lcbiGlcobalamin biosynthesis protein CbiGl- |
| STM2029_97_131 | 0.00 | -6.63 | 1253550lcbiFlvitamin B12 biosynthetic proteinl- |
| STM2030_437_471 | 0.00 | -3.87 | 1253551lcbiTlcobalt-precorrin-6Y C(15)-methyltransferasel- |
| STM2032_813_847 | 0.00 | -2.03 | 1253553lcbiDlcobalt-precorrin-6A synthasel- |
| STM2033_593_627 | 0.01 | -3.18 | 1253554lcbiClprecorrin-8X methylmutasel- |
| STM2034_705_739 | 0.05 | -1.40 | 1253555lcobDlcobalamin biosynthesis proteinl- |
| STM2035_381_415 | 0.06 | 1.84 | 1253556lcbiAlcobyrinic acid a c-diamide synthasel- |
| STM2036_313_347 | 0.97 | 1.01 | 1253557lpocRltranscriptional regulatorl- |
| STM2037_397_431 | 0.06 | -1.22 | 1253558lpduFlpropanediol diffusion facilitatorl- |
| STM2038_69_103 | 0.09 | 1.29 | 1253559lpduAlpolyhedral body proteinl+ |
| STM2042_17_51 | 0.15 | 1.14 | 1253563lpduElpropanediol dehydratase small subunitl+ |
| STM2043_1314_1348 | 0.04 | 2.19 | 1253564lpduGlpropanediol dehydratase reactivation proteinl+ |
| STM2045_61_95 | 0.01 | 4.03 | 1253566lpduJlpolyhedral body proteinl+ |
| STM2047_17_51 | 0.02 | 2.33 | 1253568lpduLlpropanediol utilization proteinl+ |
| STM2048_9_43 | 0.04 | 1.55 | 1253569lpduMlpropanediol utilization proteinl+ |
| STM2049_23_57 | 0.25 | -1.33 | 1253570lpduNlpolyhedral body proteinl+ |
| STM2051_1164_1198 | 0.15 | 1.35 | 1253572lpduPlCoA-dependent propionaldehyde dehydrogenasel+ |
| STM2055_141_175 | 0.90 | -1.02 | 1253576lpduUlpolyhedral body proteinl+ |
| STM2056_401_435 | 0.94 | -1.01 | 1253577lpduVlpropanediol utilization proteinl+ |
| STM2057_216_250 | 0.91 | -1.01 | 1253578lpduWlpropionate kinasel+ |
| STM2058_785_819 | 0.05 | 2.13 | 1253579lpduXlpropanediol utilization proteinl+ |
| STM2059_161_195 | 0.73 | 1.06 | 1253580lyeeXlhypothetical proteinl- |

| | | | |
|---|---|---|---|
| STM2060_604_638 | 0.37 | 1.11 | 1253581\|yeeA\|putative inner membrane protein\|- |
| STM2061_225_259 | 0.01 | -2.68 | 1253582\|sbmC\|DNA gyrase inhibitor\|- |
| STM2062_1118_1152 | 0.01 | 2.41 | 1253583\|dacD\|D-alanyl-D-alanine carboxypeptidase\|- |
| STM2063_265_299 | 0.37 | 1.07 | 1253584\|phsC\|thiosulfate reductase cytochrome B subunit\|- |
| STM2064_97_131 | 0.14 | 1.21 | 1253585\|phsB\|thiosulfate reductase electron transport protein\|- |
| STM2066_2198_2232 | 0.03 | 1.40 | 1253587\|sopA\|secreted effector protein\|+ |
| STM2067_768_802 | 0.12 | -1.25 | 1253588\|sbcB\|exonuclease I\|+ |
| STM2069_129_163 | 0.01 | 1.48 | 1253590\|yeeY\|putative transcriptional regulator\|- |
| STM2070_9_43 | 0.29 | -1.09 | 1253591\|yeeZ\|putative dehydratase\|- |
| STM2071_129_163 | 0.27 | -1.15 | 1253592\|hisG\|ATP phosphoribosyltransferase\|+ |
| STM2072_946_980 | 0.27 | 1.08 | 1253593\|hisD\|histidinol dehydrogenase\|+ |
| STM2073_861_895 | 0.14 | -1.36 | 1253594\|hisC\|histidinol-phosphate aminotransferase\|+ |
| STM2074_453_487 | 0.27 | -1.11 | 1253595\|hisB\|imidazole glycerol-phosphate dehydratase/histidinol phosphatase\|+ |
| STM2075_161_195 | 0.93 | 1.01 | 1253596\|hisH\|imidazole glycerol phosphate synthase subunit HisH\|+ |
| STM2076_497_531 | 0.33 | 1.17 | 1253597\|hisA\|1-(5-phosphoribosyl)-5-[(5-phosphoribosylamino)methylideneamino] imidazole-4-carboxamide isomerase\|+ |
| STM2077_65_99 | 0.44 | 1.14 | 1253598\|hisF\|imidazole glycerol phosphate synthase subunit HisF\|+ |
| STM2078_129_163 | 0.68 | 1.04 | 1253599\|hisI\|bifunctional phosphoribosyl-AMP cyclohydrolase/phosphoribosyl-ATP pyrophosphatase protein\|+ |
| STM2080_696_730 | 0.01 | 2.48 | 1253601\|udg\|UDP-glucose/GDP-mannose dehydrogenase\|- |
| STM2081_824_858 | 0.21 | -1.25 | 1253602\|gnd\|6-phosphogluconate dehydrogenase\|- |
| STM2082_928_962 | 0.01 | -1.45 | 1253603\|rfbP\|undecaprenol-phosphate galactosephosphotransferase/O-antigen transferase\|- |
| STM2083_1023_1057 | 0.98 | -1.00 | 1253604\|rfbK\|phosphomannomutase\|- |
| STM2084_1193_1227 | 0.03 | -1.54 | 1253605\|rfbM\|mannose-1-phosphate guanylyltransferase\|- |
| STM2085_321_355 | 0.09 | 1.29 | 1253606\|rfbN\|rhamnosyl transferase\|- |
| STM2086_71_105 | 0.73 | 1.08 | 1253607\|rfbU\|mannosyl transferase\|- |
| STM2087_363_399 | 0.15 | 1.31 | 1253608\|rfbV\|abequosyltransferase\|- |
| STM2088_845_881 | 0.04 | 2.22 | 1253609\|rfbX\|putative O-antigen transferase\|- |
| STM2089_556_590 | 0.26 | -1.16 | 1253610\|rfbJ\|CDP-abequose synthase\|- |
| STM2090_779_813 | 0.11 | -1.19 | 1253611\|rfbH\|CDP-6-deoxy-D-xylo-4-hexulose-3-dehydrase\|- |
| STM2091_593_627 | 0.01 | -1.68 | 1253612\|rfbG\|CDP glucose 4 6-dehydratase\|- |
| STM2092_385_419 | 0.03 | -1.86 | 1253613\|rfbF\|glucose-1-phosphate cytidylyltransferase\|- |
| STM2093_81_115 | 0.02 | -1.87 | 1253614\|rfbI\|CDP-6-deoxy-delta-3 4-glucoseen reductase\|- |
| STM2094_513_547 | 0.03 | -1.55 | 1253615\|rfbC\|dTDP-4 deoxyrhamnose 3 5 epimerase\|- |

| | | | |
|---|---|---|---|
| STM2095_145_179 | 0.04 | -1.27 | 1253616lrfbAldTDP-glucose pyrophosphorylasel- |
| STM2096_129_163 | 0.09 | -1.23 | 1253617lrfbDldTDP-4-dehydrorhamnose reductasel- |
| STM2097_247_281 | 0.04 | 1.63 | 1253618lrfbBldTDP-glucose 4 6 dehydratasel- |
| STM2098_17_51 | 0.02 | -1.94 | 1253619lgalFlUTP--glucose-1-phosphate uridylyltransferase subunit GalFl- |
| STM2099_693_727 | 0.01 | 1.99 | 1253620lwcaMlputative colanic acid biosynthetic proteinl- |
| STM2100_478_512 | 0.08 | 1.91 | 1253621lwcaLlputative glycosyl transferasel- |
| STM2101_634_668 | 0.09 | -1.18 | 1253622lwcaKlputative pyruvyl transferasel- |
| STM2102_792_826 | 0.22 | 1.22 | 1253623lwzxClcolanic acid exporterl- |
| STM2103_780_814 | 0.39 | 1.10 | 1253624lwcaJlputative UDP-glucose lipid carrier transferasel- |
| STM2104_932_966 | 0.02 | -1.48 | 1253625lcpsGlphosphomannomutasel- |
| STM2106_513_547 | 0.03 | 2.41 | 1253627lwcaIlpredicted glycosyl transferasel- |
| STM2107_326_360 | 0.63 | 1.03 | 1253628lwcaHlGDP-mannose mannosyl hydrolasel- |
| STM2108_481_515 | 0.18 | 1.15 | 1253629lwcaGlGDP-fucose synthetasel- |
| STM2109_283_317 | 0.06 | 1.85 | 1253630lgmdlGDP-D-mannose dehydratasel- |
| STM2111_153_187 | 0.21 | 1.22 | 1253632lwcaElpredicted glycosyl transferasel- |
| STM2112_936_970 | 0.01 | 1.31 | 1253633lwcaDlputative colanic acid polymerasel- |
| STM2113_219_253 | 0.91 | -1.02 | 1253634lwcaClpredicted glycosyl transferasel- |
| STM2114_193_227 | 0.17 | 1.47 | 1253635lwcaBlputative colanic acid biosynthesis acetyltransferase WcaBl- |
| STM2115_249_283 | 0.15 | 1.18 | 1253636lwcaAlpredicted glycosyl transferasel- |
| STM2116_1673_1707 | 0.01 | 1.31 | 1253637lwzcltyrosine kinasel- |
| STM2117_209_243 | 0.29 | 1.61 | 1253638lwzbltyrosine phosphatasel- |
| STM2118_429_463 | 0.13 | 1.41 | 1253639lwzalputative outer membrane polysaccharide export proteinl- |
| STM2119_886_920 | 0.86 | 1.02 | 1253640lyegHlputative inner membrane proteinl+ |
| STM2120_1498_1532 | 0.01 | -1.86 | 1253641lasmAlputative assembly proteinl- |
| STM2121_145_179 | 0.03 | -1.94 | 1253642ldcdldeoxycytidine triphosphate deaminasel- |
| STM2122_257_291 | 0.04 | -1.41 | 1253643ludkluridine kinasel- |
| STM2123_2600_2634 | 0.07 | -1.21 | 1253644lyegElputative diguanylate cyclase/phosphodiesterasel+ |
| STM2127_2252_2286 | 0.02 | 2.58 | 1253648lyegNlmultidrug efflux system subunit MdtBl+ |
| STM2128_2146_2180 | 0.12 | -1.21 | 1253649lyegOlmultidrug efflux system subunit MdtCl+ |
| STM2129_958_992 | 0.40 | 1.09 | 1253650lyegBlmultidrug efflux system protein MdtEl+ |
| STM2130_661_695 | 0.04 | 1.53 | 1253651lbaeSlsignal transduction histidine-protein kinase BaeSl+ |
| STM2131_113_147 | 0.34 | 1.08 | 1253652lbaeRlDNA-binding response regulator in two-component regulatory system with BaeSl+ |
| STM2133_705_739 | 0.07 | 1.72 | 1253654lSTM2133lputative cytoplasmic proteinl+ |
| STM2134_185_219 | 0.05 | 2.44 | 1253655lSTM2134lputative inner membrane proteinl+ |
| STM2135_1481_1515 | 0.04 | 1.29 | 1253656lSTM2135lputative inner membrane proteinl+ |

| | | | |
|---|---|---|---|
| STM2136_747_781 | 0.01 | -1.63 | 1253657\|yegQ\|putative protease\|+ |
| STM2137_884_918 | 0.68 | -1.07 | 1253658\|STM2137\|putative cytoplasmic protein\|+ |
| STM2138_104_142 | 0.47 | -1.13 | 1253659\|STM2138\|putative cytoplasmic protein\|- |
| STM2139_189_223 | 0.02 | 1.52 | 1253660\|STM2139\|putative inner membrane protein\|- |
| STM2139.2n_49_83 | 0.69 | 1.05 | 2673733\|STM2139.2n\|hypothetical protein\|- |
| STM2140_49_83 | 0.93 | -1.02 | 1253661\|yegS\|lipid kinase\|+ |
| STM2141_118_152 | 0.77 | -1.05 | 1253662\|fbaB\|fructose-bisphosphate aldolase\|- |
| STM2142_433_467 | 0.03 | 1.41 | 1253663\|yegT\|putative transport protein\|+ |
| STM2143_6_40 | 0.14 | 1.42 | 1253664\|yegU\|putative glycohydrolase\|+ |
| STM2144_529_563 | 0.20 | 1.16 | 1253665\|yegV\|putative sugar kinase\|+ |
| STM2145_537_571 | 0.01 | 1.69 | 1253666\|yegW\|putative regulatory protein\|- |
| STM2146_385_419 | 0.00 | 1.89 | 1253667\|thiD\|phosphomethylpyrimidine kinase\|- |
| STM2148_137_171 | 0.01 | 2.49 | 1253669\|STM2148\|putative periplasmic protein\|+ |
| STM2149_289_323 | 0.07 | -1.24 | 1253670\|stcD\|putative outer membrane lipoprotein\|- |
| STM2150_1891_1925 | 0.02 | 1.44 | 1253671\|stcC\|putative outer membrane protein\|- |
| STM2151_105_139 | 0.02 | 2.33 | 1253672\|stcB\|putative periplasmic chaperone protein\|- |
| STM2152_345_379 | 0.04 | 1.80 | 1253673\|stcA\|putative fimbrial-like protein\|- |
| STM2153_201_235 | 0.00 | 6.82 | 1253674\|yehE\|putative outer membrane protein\|- |
| STM2154_639_673 | 0.32 | -1.14 | 1253675\|mrp\|putative ATPase\|- |
| STM2155_1467_1501 | 0.00 | -2.19 | 1253676\|metG\|methionyl-tRNA synthetase\|+ |
| STM2156_213_247 | 0.06 | 2.13 | 1253677\|yehR\|putative lipoprotein\|+ |
| STM2156A_321_355 | 0.15 | 1.38 | 1253678\|STM2156A\|hypothetical protein\|+ |
| STM2157_321_355 | 0.03 | -1.42 | 1253679\|yehS\|putative cytoplasmic protein\|- |
| STM2158_121_155 | 0.01 | -2.24 | 1253680\|yehT\|putative two-component response-regulatory protein YehT\|- |
| STM2159_1327_1361 | 0.04 | -1.74 | 1253681\|yehU\|putative sensor kinase\|- |
| STM2160_209_243 | 0.03 | -1.61 | 1253682\|yehV\|putative transcriptional repressor\|+ |
| STM2161_25_59 | 0.44 | -1.09 | 1253683\|STM2161\|putative inner membrane protein\|+ |
| STM2162_601_635 | 0.02 | 1.80 | 1253684\|yehW\|putative ABC-type proline/glycine betaine transport system permease component\|- |
| STM2163_321_355 | 0.02 | -2.03 | 1253685\|yehX\|putative ABC-type proline/glycine betaine transport system ATPase component\|- |
| STM2164_1067_1101 | 0.02 | -1.23 | 1253686\|yehY\|putative ABC-type proline/glycine betaine transport system permease component\|- |
| STM2165_81_115 | 0.26 | -1.13 | 1253687\|yehZ\|putative transport protein\|- |
| STM2166_2227_2261 | 0.48 | 1.05 | 1253688\|bglX\|periplasmic beta-D-glucoside glucohydrolase\|- |
| STM2167_876_910 | 0.26 | 1.14 | 1253689\|dld\|D-lactate dehydrogenase\|+ |

| | | | |
|---|---|---|---|
| STM2168_817_851 | 0.02 | 1.59 | 1253690lpbpGlD-alanyl-D-alanine endopeptidasel- |
| STM2169_161_195 | 0.04 | 1.70 | 1253691lyohClputative transport proteinl- |
| STM2170_201_235 | 0.75 | -1.04 | 1253692lyohDlhypothetical proteinl+ |
| STM2171_273_307 | 0.08 | -1.34 | 1253693lyohFlacetoin dehydrogenasel- |
| STM2172_646_680 | 0.02 | 1.43 | 1253694lyohGlmultidrug resistance outer membrane protein MdtQl- |
| STM2174_785_819 | 0.76 | -1.03 | 1253696lyohIltRNA-dihydrouridine synthase Cl- |
| STM2176_529_563 | 0.31 | -1.19 | 1253698lSTM2176lputative glutathione S-transferasel- |
| STM2177_649_683 | 0.02 | -1.67 | 1253699lSTM2177lputative flutathione S-transferasel- |
| STM2178_327_361 | 0.05 | 1.95 | 1253700lSTM2178lputative 1 2-dioxygenasel- |
| STM2179_824_858 | 0.10 | -1.42 | 1253701lSTM2179lputative sugar transporterl- |
| STM2181_337_371 | 0.00 | 3.65 | 1253703lyohJlhypothetical proteinl+ |
| STM2182_289_323 | 0.07 | 1.54 | 1253704lyohKlpredicted inner membrane proteinl+ |
| STM2183_849_883 | 0.01 | 2.58 | 1253705lcddlcytidine deaminasel+ |
| STM2184_465_499 | 0.05 | 1.53 | 1253706lsanAlhypothetical proteinl+ |
| STM2185_121_155 | 0.35 | -1.04 | 1253707lb2145lputative inner membrane proteinl+ |
| STM2186_507_541 | 0.02 | -1.86 | 1253708lSTM2186lputative oxidoreductasel+ |
| STM2187_1037_1071 | 0.02 | -1.59 | 1253709lyeiAldihydropyrimidine dehydrogenasel+ |
| STM2188_524_558 | 0.01 | -2.53 | 1253710lmglClbeta-methylgalactoside transporter inner membrane componentl- |
| STM2189_1034_1068 | 0.03 | -2.03 | 1253711lmglAlgalactose/methyl galaxtoside transporter ATP-binding proteinl- |
| STM2190_817_851 | 0.00 | -10.12 | 1253712lmglBlgalactose transport proteinl- |
| STM2191_856_890 | 0.00 | -4.75 | 1253713lgalSlDNA-binding transcriptional repressorl- |
| STM2192_400_434 | 0.65 | 1.04 | 1253714lyeiBlhypothetical proteinl- |
| STM2193_513_547 | 0.03 | -1.52 | 1253715lfolElGTP cyclohydrolase Il- |
| STM2194_643_677 | 0.18 | 1.24 | 1253716lyeiGlputative esterasel+ |
| STM2195_25_59 | 0.05 | 1.44 | 1253717lSTM2195lputative transcriptional regulatorl- |
| STM2196_1265_1299 | 0.03 | 1.65 | 1253718lSTM2196lputative L-serine dehydratasel- |
| STM2197_633_667 | 0.04 | 2.68 | 1253719lSTM2197lputative phosphoserine phosphatasel+ |
| STM2198_356_390 | 0.49 | 1.13 | 1253720lSTM2198lputative regulatory proteinl+ |
| STM2199_1185_1219 | 0.08 | -1.29 | 1253721lcirAlferric iron-catecholate outer membrane transporterl- |
| STM2200_1119_1153 | 0.01 | -1.73 | 1253722llysPllysine transporterl- |
| STM2201_257_291 | 0.50 | -1.21 | 1253723lyeiElputative DNA-binding transcriptional regulatorl- |
| STM2202_307_341 | 0.38 | -1.17 | 1253724lyeiHlputative inner membrane proteinl+ |
| STM2203_785_819 | 0.02 | 1.58 | 1253725lnfolendonuclease IVl+ |
| STM2204_1246_1280 | 0.03 | 1.29 | 1253726lfruAlfructose-specific PTS system IIBC componentl- |
| STM2205_385_419 | 0.37 | -1.09 | 1253727lfruKl1-phosphofructokinasel- |

| | | | |
|---|---|---|---|
| STM2206_692_726 | 0.03 | -1.63 | 1253728\|fruF\|fructose-specific PTS IIA/HPr components\|- |
| STM2208_536_570 | 0.50 | -1.07 | 1253730\|STM2208\|putative inner membrane protein\|- |
| STM2209.1c_93_127 | 0.04 | 1.29 | 2673743\|STM2209.1c\|hypothetical protein\|- |
| STM2212_513_547 | 0.04 | -1.54 | 1253734\|yeiR\|putative cobalamin biosynthetic protein\|+ |
| STM2213_289_323 | 0.01 | -1.61 | 1253735\|yeiU\|putative permease\|+ |
| STM2214_457_491 | 0.00 | -2.98 | 1253736\|spr\|putative lipoprotein\|+ |
| STM2215_942_976 | 0.01 | 1.90 | 1253737\|rtn\|hypothetical protein\|+ |
| STM2216_1655_1689 | 0.02 | -1.71 | 1253738\|yejA\|putative ABC transporter periplasmic binding protein\|+ |
| STM2217_752_786 | 0.02 | 2.73 | 1253739\|yejB\|putative ABC-type dipeptide/oligopeptide/nickel transport system permease\|+ |
| STM2218_731_765 | 0.03 | 1.24 | 1253740\|yejE\|putative ABC-type dipeptide/oligopeptide/nickel transport system permease component\|+ |
| STM2219_1359_1393 | 0.01 | 1.75 | 1253741\|yejF\|putative ABC-type transport system ATPase component\|+ |
| STM2220_17_51 | 0.04 | -1.46 | 1253742\|yejG\|hypothetical protein\|- |
| STM2221_960_994 | 0.09 | 1.49 | 1253743\|bcr\|bicyclomycin/multidrug efflux system\|- |
| STM2222_193_227 | 0.83 | 1.02 | 1253744\|rsuA\|16S rRNA pseudouridylate synthase A\|- |
| STM2223_954_988 | 0.16 | 1.07 | 1253745\|yejH\|putative ATP-dependent helicase\|+ |
| STM2224_69_103 | 0.03 | -1.92 | 1253746\|rplY\|50S ribosomal protein L25\|+ |
| STM2225_273_307 | 0.01 | -2.03 | 1253747\|STM2225\|putative inner membrane protein\|- |
| STM2226_265_299 | 0.02 | -2.18 | 1253748\|yejK\|nucleoid-associated protein NdpA\|- |
| STM2227_117_151 | 0.04 | -1.27 | 1253749\|yejL\|putative cytoplasmic protein\|+ |
| STM2228_794_828 | 0.04 | -1.26 | 1253750\|yejM\|putative hydrolase\|+ |
| STM2230.1c_121_155 | 0.82 | -1.04 | 1253752\|STM2230.1c\|DNA polymerase V subunit\|- |
| STM2232_1399_1433 | 0.02 | 2.02 | 1253754\|oafA\|O-antigen acetylase\|+ |
| STM2233_53_87 | 0.01 | 4.10 | 1253755\|STM2233\|putative cytoplasmic protein\|- |
| STM2235_339_373 | 0.00 | 4.46 | 1253757\|STM2235\|hypothetical protein\|- |
| STM2236_65_99 | 0.01 | 3.42 | 1253758\|STM2236\|hypothetical protein\|- |
| STM2237_65_99 | 0.02 | 2.64 | 1253759\|STM2237\|putative inner membrane protein\|- |
| STM2238_369_403 | 0.23 | 1.30 | 1253760\|STM2238\|hypothetical protein\|+ |
| STM2239_113_147 | 0.02 | 2.52 | 1253761\|STM2239\|putative phage antiterminator\|- |
| STM2240_457_491 | 0.02 | 2.38 | 1253762\|STM2240\|putative cytoplasmic protein\|- |
| STM2241_1920_1954 | 0.32 | 2.25 | 1253763\|sspH2\|leucine-rich repeat protein\|- |
| STM2242_13_47 | 0.06 | 1.79 | 1253764\|STM2242\|putative phage tail fiber protein\|- |
| STM2243_673_707 | 0.25 | 1.81 | 1253765\|STM2243\|putative tail fiber protein of phage\|- |
| STM2244_87_122 | 0.03 | 3.07 | 1253766\|STM2244\|virulence protein\|+ |
| STM2245_289_323 | 0.00 | 3.00 | 1253767\|STM2245\|putative outer membrane protein\|- |

| | | | |
|---|---|---|---|
| STM2246_305_339 | 0.37 | -1.15 | 1253768\|narP\|DNA-binding response regulator in two-component regulatory system with NarQ or NarX\|+ |
| STM2247_884_918 | 0.63 | 1.03 | 1253769\|ccmH\|putative heme lyase subunit\|- |
| STM2248/STM3813_355_38 | 0.00 | 1.22 | 1253770/1255340\|ccmG/ccmG\|heme lyase/disulfide oxidoreductase\|- |
| STM2249/STM3814_1805_1 | 0.11 | -1.23 | 1253771/1255341\|ccmF/ccmF\|cytochrome c-type biogenesis protein\|- |
| STM2252/STM3817_497_53 | 0.92 | -1.01 | 1253774/1255344\|ccmC/ccmC\|heme exporter protein\|- |
| STM2253/STM3818_543_57 | 0.01 | -1.71 | 1253775/1255345\|ccmB/ccmB\|heme exporter protein\|- |
| STM2255_9_43 | 0.58 | 1.07 | 1253777\|napC\|cytochrome c-type protein NapC\|- |
| STM2256_389_423 | 0.83 | -1.07 | 1253778\|napB\|diheme cytochrome c550\|- |
| STM2257_73_107 | 0.35 | -1.15 | 1253779\|napH\|quinol dehydrogenase membrane component\|- |
| STM2258_445_479 | 0.02 | 1.44 | 1253780\|napG\|quinol dehydrogenase periplasmic component\|- |
| STM2259_2368_2402 | 0.05 | -1.26 | 1253781\|napA\|periplasmic nitrate reductase\|- |
| STM2261_353_387 | 0.73 | -1.11 | 1253783\|napF\|ferredoxin-type protein\|- |
| STM2262_193_227 | 0.00 | 2.67 | 1253784\|eco\|ecotin precursor\|+ |
| STM2263_693_727 | 0.13 | -1.29 | 1253785\|yojI\|multidrug transporter membrane component/ATP-binding component\|- |
| STM2264_281_315 | 0.22 | 1.09 | 1253786\|alkB\|DNA repair system protein\|- |
| STM2266_846_880 | 0.05 | -1.16 | 1253788\|apbE\|thiamine biosynthesis lipoprotein ApbE\|- |
| STM2267_746_780 | 0.02 | -2.67 | 1253789\|ompC\|outer membrane porin protein C\|- |
| STM2268_17_51 | 0.90 | -1.01 | 1253790\|micF\|misc_RNA\|- |
| STM2269_2567_2601 | 0.22 | -1.28 | 1253791\|yojN\|phosphotransfer intermediate protein in two-component regulatory system with RcsBC\|+ |
| STM2270_129_163 | 0.06 | -1.48 | 1253792\|rcsB\|DNA-binding response regulator in two-component regulatory system with RcsC and YojN\|+ |
| STM2271_2288_2322 | 0.09 | 1.39 | 1253793\|rcsC\|hybrid sensory kinase in two-component regulatory system with RcsB and YojN\|- |
| STM2272_2350_2384 | 0.02 | -2.41 | 1253794\|gyrA\|DNA gyrase subunit A\|- |
| STM2273_468_502 | 0.26 | 1.32 | 1253795\|STM2273\|putative dehydratase\|- |
| STM2274_1087_1121 | 0.17 | 2.01 | 1253796\|STM2274\|putative permease\|- |
| STM2275_497_531 | 0.00 | 2.38 | 1253797\|STM2275\|putative regulatory protein\|+ |
| STM2276_673_707 | 0.03 | 2.05 | 1253798\|ubiG\|3-demethylubiquinone-9 3-methyltransferase\|+ |
| STM2277_2087_2121 | 0.35 | -1.23 | 1253799\|nrdA\|ribonucleotide-diphosphate reductase subunit alpha\|+ |
| STM2278_452_486 | 0.03 | 1.40 | 1253800\|nrdB\|ribonucleotide-diphosphate reductase subunit beta\|+ |
| STM2279_145_179 | 0.09 | -1.61 | 1253801\|yfaE\|2Fe-2S ferredoxin YfaE\|+ |
| STM2280_272_306 | 0.03 | 1.15 | 1253802\|STM2280\|putative permease\|- |
| STM2281_129_163 | 0.22 | 1.44 | 1253803\|STM2281\|putative transcriptional regulator\|+ |

| | | | |
|---|---|---|---|
| STM2282_840_874 | 0.01 | -11.80 | 1253804\|glpQ\|glycerophosphodiester phosphodiesterase\|- |
| STM2283_1080_1114 | 0.21 | -1.43 | 1253805\|glpT\|sn-glycerol-3-phosphate transporter\|- |
| STM2284_758_792 | 0.00 | -14.13 | 1253806\|glpA\|sn-glycerol-3-phosphate dehydrogenase subunit A\|+ |
| STM2285_517_551 | 0.00 | -8.86 | 1253807\|glpB\|anaerobic glycerol-3-phosphate dehydrogenase subunit B\|+ |
| STM2286_368_402 | 0.00 | -6.04 | 1253808\|glpC\|sn-glycerol-3-phosphate dehydrogenase subunit C\|+ |
| STM2287_817_851 | 0.14 | 3.14 | 1253809\|sseL\|deubiquitinase\|+ |
| STM2288_34_68 | 0.01 | -1.68 | 1253810\|STM2288\|putative cytoplasmic protein\|- |
| STM2289_577_611 | 0.02 | -1.94 | 1253811\|STM2289\|putative aldolase\|- |
| STM2290_867_901 | 0.05 | -1.41 | 1253812\|yfaV\|putative transport protein\|- |
| STM2291_347_381 | 0.02 | -2.36 | 1253813\|yfaW\|putative galactonate dehydratase\|- |
| STM2292_193_227 | 0.00 | -2.30 | 1253814\|yfaX\|putative transcriptional regulator\|- |
| STM2293_806_840 | 0.28 | 1.10 | 1253815\|STM2293\|competence damage-inducible protein A\|- |
| STM2294_17_51 | 0.03 | 2.31 | 1253816\|yfaZ\|putative inner membrane protein\|- |
| STM2295_205_239 | 0.13 | -1.21 | 1253817\|yfaO\|putative NTP pyrophosphohydrolase\|+ |
| STM2296_513_547 | 0.19 | 1.43 | 1253818\|ais\|aluminum-inducible protein\|- |
| STM2297_533_567 | 0.03 | 1.52 | 1253819\|yfbE\|UDP-4-amino-4-deoxy-L-arabinose--oxoglutarate aminotransferase\|+ |
| STM2298_833_867 | 0.02 | 1.49 | 1253820\|pmrF\|undecaprenyl phosphate 4-deoxy-4-formamido-L-arabinose transferase\|+ |
| STM2299_1816_1850 | 0.02 | 1.86 | 1253821\|yfbG\|bifunctional UDP-glucuronic acid decarboxylase/UDP-4-amino-4-deoxy-L-arabinose formyltransferase\|+ |
| STM2300_577_611 | 0.13 | 1.14 | 1253822\|STM2300\|putative cytoplasmic protein\|+ |
| STM2301_1416_1450 | 0.02 | -1.54 | 1253823\|arnT\|4-amino-4-deoxy-L-arabinose transferase\|+ |
| STM2302_181_215 | 0.87 | -1.02 | 1253824\|STM2302\|putative inner membrane protein\|+ |
| STM2303_269_303 | 0.00 | 1.43 | 1253825\|STM2303\|hypothetical protein\|+ |
| STM2304_105_139 | 0.01 | 4.17 | 1253826\|pmrD\|polymyxin resistance protein B\|- |
| STM2305_1081_1115 | 0.01 | -1.65 | 1253827\|menE\|O-succinylbenzoic acid--CoA ligase\|- |
| STM2306_561_595 | 0.10 | 1.14 | 1253828\|menC\|O-succinylbenzoate synthase\|- |
| STM2307_129_163 | 0.05 | -1.19 | 1253829\|menB\|naphthoate synthase\|- |
| STM2308_113_147 | 0.03 | -2.26 | 1253830\|yfbB\|acyl-CoA thioester hydrolase YfbB\|- |
| STM2309_896_930 | 0.03 | -1.64 | 1253831\|menD\|2-succinyl-6-hydroxy-2 4-cyclohexadiene-1-carboxylic acid synthase/2-oxoglutarate decarboxylase\|- |
| STM2311_169_203 | 0.81 | -1.03 | 1253833\|elaB\|hypothetical protein\|- |
| STM2312_81_115 | 0.62 | -1.04 | 1253834\|elaA\|hypothetical protein\|- |
| STM2313_433_467 | 0.01 | -1.49 | 1253835\|elaC\|ribonuclease Z\|+ |
| STM2314_67_101 | 0.43 | -1.08 | 1253836\|STM2314\|putative chemotaxis signal transduction protein\|+ |

| | | | |
|---|---|---|---|
| STM2315_1263_1297 | 0.01 | 1.62 | 1253837\|yfbK\|hypothetical protein\|- |
| STM2316.S_971_1005 | 0.01 | -2.18 | 1253838\|nuoN\|NADH dehydrogenase subunit N\|- |
| STM2317_915_949 | 0.01 | -1.41 | 1253839\|nuoM\|NADH dehydrogenase subunit M\|- |
| STM2318_1639_1673 | 0.01 | -1.73 | 1253840\|nuoL\|NADH dehydrogenase subunit L\|- |
| STM2319_163_197 | 0.01 | -2.01 | 1253841\|nuoK\|NADH dehydrogenase subunit K\|- |
| STM2320_65_99 | 0.00 | -2.24 | 1253842\|nuoJ\|NADH dehydrogenase subunit J\|- |
| STM2321_441_475 | 0.06 | -1.45 | 1253843\|nuoI\|NADH dehydrogenase subunit I\|- |
| STM2322_929_963 | 0.01 | -1.57 | 1253844\|nuoH\|NADH dehydrogenase subunit H\|- |
| STM2323.S_2568_2602 | 0.00 | -2.06 | 1253845\|nuoG\|NADH dehydrogenase subunit G\|- |
| STM2324_1155_1189 | 0.67 | 1.03 | 1253846\|nuoF\|NADH dehydrogenase I subunit F\|- |
| STM2325_213_247 | 0.98 | -1.00 | 1253847\|nuoE\|NADH dehydrogenase subunit E\|- |
| STM2326_820_854 | 0.60 | -1.08 | 1253848\|nuoC\|bifunctional NADH:ubiquinone oxidoreductase subunit C/D\|- |
| STM2327_417_451 | 0.11 | -1.25 | 1253849\|nuoB\|NADH dehydrogenase subunit B\|- |
| STM2328_129_163 | 0.05 | -1.26 | 1253850\|nuoA\|NADH dehydrogenase subunit A\|- |
| STM2329_41_75 | 0.13 | 2.53 | 1253851\|STM2329\|putative cytoplasmic protein\|- |
| STM2330_385_419 | 0.01 | 2.23 | 1253852\|lrhA\|NADH dehydrogenase transcriptional repressor\|- |
| STM2331_232_266 | 0.29 | 1.12 | 1253853\|yfbQ\|aminotransferase AlaT\|+ |
| STM2332_193_227 | 0.06 | 1.98 | 1253854\|STM2332\|hypothetical protein\|+ |
| STM2333_988_1022 | 0.77 | -1.03 | 1253855\|yfbS\|putative response regulator\|- |
| STM2334_33_67 | 0.27 | 1.24 | 1253856\|yfbT\|putative phosphatase\|- |
| STM2335_297_331 | 0.03 | 1.54 | 1253857\|yfbU\|hypothetical protein\|- |
| STM2336_257_291 | 0.43 | 1.05 | 1253858\|STM2336\|hypothetical protein\|- |
| STM2337_460_494 | 0.02 | 1.47 | 1253859\|ackA\|acetate kinase\|+ |
| STM2338_1626_1660 | 0.37 | 1.24 | 1253860\|pta\|phosphate acetyltransferase\|+ |
| STM2340_673_707 | 0.02 | -2.02 | 1253862\|STM2340\|putative transketolase\|- |
| STM2341_225_259 | 0.00 | -4.17 | 1253863\|STM2341\|putative transketolase\|- |
| STM2342_527_561 | 0.01 | -3.77 | 1253864\|ulaA\|ascorbate-specific PTS system enzyme IIC\|- |
| STM2344_265_299 | 0.02 | -1.85 | 1253866\|STM2344\|putative phosphotransferase system enzyme II A component\|- |
| STM2345_21_55 | 0.05 | 1.32 | 1253867\|STM2345\|putative transcriptional regulator\|+ |
| STM2346_9_43 | 0.01 | -1.51 | 1253868\|STM2346\|putative NTP pyrophosphohydrolase\|- |
| STM2347_97_131 | 0.08 | -1.33 | 1253869\|yfcE\|phosphodiesterase\|- |
| STM2348_345_379 | 0.02 | 2.95 | 1253870\|yfcF\|putative glutathione S-transferase\|- |
| STM2349_385_419 | 0.17 | 1.24 | 1253871\|yfcG\|putative glutathione S-transferase\|+ |
| STM2350_385_419 | 0.07 | 1.23 | 1253872\|yfcH\|putative sugar nucleotide epimerase\|+ |
| STM2351_353_387 | 0.00 | -1.28 | 1253873\|hisP\|histidine/lysine/arginine/ornithine transporter subunit\|- |

| | | | |
|---|---|---|---|
| STM2352_577_611 | 0.30 | 1.20 | 1253874\|hisM\|histidine/lysine/arginine/ornithine transport protein\|- |
| STM2353_209_243 | 0.01 | -1.51 | 1253875\|hisQ\|histidine/lysine/arginine/ornithine transport protein\|- |
| STM2354_81_115 | 0.00 | -3.61 | 1253876\|hisJ\|histidine transport protein\|- |
| STM2355_269_303 | 0.03 | -2.08 | 1253877\|argT\|lysine/arginine/ornithine transport protein\|- |
| STM2356_377_411 | 0.79 | 1.03 | 1253878\|ubiX\|3-octaprenyl-4-hydroxybenzoate carboxy-lyase\|- |
| STM2357_870_904 | 0.00 | -1.41 | 1253879\|STM2357\|putative amino acid transporter\|- |
| STM2358_617_651 | 0.90 | 1.02 | 1253880\|STM2358\|putative cytoplasmic protein\|- |
| STM2359_1079_1113 | 0.02 | 1.70 | 1253881\|STM2359\|putative amino acid transporter\|- |
| STM2360_871_905 | 0.02 | 1.60 | 1253882\|STM2360\|putative diaminopimelate decarboxylase\|- |
| STM2361_629_663 | 0.48 | -1.32 | 1253883\|STM2361\|putative regulatory protein\|+ |
| STM2362_1031_1065 | 0.01 | -3.34 | 1253884\|purF\|amidophosphoribosyltransferase\|- |
| STM2363_133_167 | 0.03 | -2.25 | 1253885\|cvpA\|colicin V production protein\|- |
| STM2364_273_307 | 0.19 | 1.14 | 1253886\|dedD\|hypothetical protein\|- |
| STM2365_1038_1072 | 0.04 | -1.66 | 1253887\|folC\|bifunctional folylpolyglutamate synthase/ dihydrofolate synthase\|- |
| STM2366_129_163 | 0.04 | -1.32 | 1253888\|accD\|acetyl-CoA carboxylase subunit beta\|- |
| STM2367_257_291 | 0.09 | 1.28 | 1253889\|dedA\|hypothetical protein\|- |
| STM2368_561_595 | 0.59 | -1.04 | 1253890\|truA\|tRNA pseudouridine synthase A\|- |
| STM2369_527_561 | 0.17 | -1.28 | 1253891\|usg\|hypothetical protein\|- |
| STM2371_723_757 | 0.01 | -1.80 | 1253893\|flk\|predicted flagella assembly protein\|+ |
| STM2372_332_366 | 0.01 | -1.96 | 1253894\|STM2372\|hypothetical protein\|- |
| STM2373_65_99 | 0.40 | 1.44 | 1253895\|STM2373\|putative cytoplasmic protein\|- |
| STM2374_65_99 | 0.02 | 2.92 | 1253896\|STM2374\|putative regulatory protein\|+ |
| STM2376_177_211 | 0.60 | -1.02 | 1253898\|STM2376\|putative periplasmic protein\|+ |
| STM2377_257_291 | 0.02 | 2.01 | 1253899\|STM2377\|putative inner membrane protein\|- |
| STM2378_504_538 | 0.12 | -1.14 | 1253900\|fabB\|3-oxoacyl-(acyl carrier protein) synthase I\|- |
| STM2379_1610_1644 | 0.01 | 1.66 | 1253901\|mnmC\|5-methylaminomethyl-2-thiouridine methyltransferase\|+ |
| STM2380_33_67 | 0.29 | -1.29 | 1253902\|yfcL\|putative cytoplasmic protein\|- |
| STM2381_129_163 | 0.46 | -1.09 | 1253903\|yfcM\|putative cytoplasmic protein\|- |
| STM2382_513_547 | 0.40 | -1.09 | 1253904\|yfcA\|hypothetical protein\|- |
| STM2383_417_451 | 0.10 | -1.13 | 1253905\|mepA\|penicillin-insensitive murein endopeptidase\|- |
| STM2384_487_521 | 0.02 | 1.74 | 1253906\|aroC\|chorismate synthase\|- |
| STM2385_129_163 | 0.07 | -1.51 | 1253907\|yfcB\|N5-glutamine S-adenosyl-L-methionine-dependent methyltransferase\|- |
| STM2386_193_227 | 0.04 | 1.95 | 1253908\|yfcN\|hypothetical protein\|+ |
| STM2387_321_355 | 0.02 | 1.90 | 1253909\|sixA\|phosphohistidine phosphatase\|- |

| | | | |
|---|---|---|---|
| STM2388_1853_1887 | 0.63 | 1.06 | 1253910lfadJlmultifunctional fatty acid oxidation complex subunit alphal- |
| STM2390_177_211 | 0.00 | 3.95 | 1253912lyfcZlputative cytoplasmic proteinl- |
| STM2391_1051_1085 | 0.00 | -26.78 | 1253913lfadLllong-chain fatty acid outer membrane transporterl+ |
| STM2392_641_675 | 0.28 | -1.14 | 1253914lvacJllipoprotein precursorl- |
| STM2393_593_627 | 0.28 | 1.18 | 1253915lyfdClhypothetical proteinl+ |
| STM2394_31_65 | 0.43 | 1.52 | 1253916largWltRNAl+ |
| STM2395_353_387 | 0.01 | 2.81 | 1253917lpgtElouter membrane proteasel- |
| STM2396_569_603 | 0.00 | -2.53 | 1253918lpgtAlactivatorl- |
| STM2397_1968_2002 | 0.00 | -2.50 | 1253919lpgtBlphosphoglycerate transport system sensor proteinl- |
| STM2398_915_949 | 0.05 | -1.34 | 1253920lpgtClphosphoglycerate transport regulatory protein precursorl- |
| STM2399_1065_1099 | 0.00 | -2.72 | 1253921lpgtPltransporterl+ |
| STM2400_189_223 | 0.69 | 1.04 | 1253922lSTM2400lputative inner membrane proteinl- |
| STM2401_699_733 | 0.03 | 1.71 | 1253923lddgllipid A biosynthesis palmitoleoyl acyltransferasel+ |
| STM2402_720_754 | 0.10 | -1.49 | 1253924lyfdZlaminotransferasel- |
| STM2403_129_163 | 0.78 | -1.03 | 1253925lglklglucokinasel- |
| STM2404_1101_1135 | 0.01 | -1.11 | 1253926lSTM2404lhypothetical proteinl+ |
| STM2405_862_896 | 0.04 | 1.44 | 1253927lSTM2405lindolepyruvate decarboxylasel- |
| STM2406_881_915 | 0.01 | 1.74 | 1253928lSTM2406lputative oxidoreductasel+ |
| STM2407_97_131 | 0.31 | 1.09 | 1253929lypeClputative periplasmic proteinl+ |
| STM2408_691_725 | 0.07 | -1.39 | 1253930lmntHlmanganese transport protein MntHl- |
| STM2409_908_942 | 0.00 | -4.12 | 1253931lnupClnucleoside transportl+ |
| STM2410_1575_1609 | 0.16 | -1.13 | 1253932lyfeAlhypothetical proteinl- |
| STM2413_33_67 | 0.04 | -2.50 | 1253935lyfeClputative negative regulatorl+ |
| STM2414_137_171 | 0.00 | -1.87 | 1253936lyfeDlputative negative regulatorl+ |
| STM2415_593_627 | 0.71 | -1.06 | 1253937lgltXlglutamyl-tRNA synthetasel- |
| STM2420_161_195 | 0.51 | 1.12 | 1253942lxapRlDNA-binding transcriptional activatorl- |
| STM2420.1n_8_46 | 0.69 | 1.48 | 2673749lSTM2420.1nlhypothetical proteinl- |
| STM2421_514_548 | 0.11 | 1.59 | 1253943lxapBlxanthosine permeasel- |
| STM2423_321_355 | 0.08 | 1.56 | 1253945lyfeNlhypothetical proteinl+ |
| STM2424_577_611 | 0.13 | 1.73 | 1253946lyfeRlputative transcriptional regulatorl- |
| STM2425_17_51 | 0.61 | 1.07 | 1253947lyfeHlputative Na+-dependent transporterl+ |
| STM2426_24_58 | 0.03 | -1.23 | 1253948lSTM2426lputative cytoplasmic proteinl- |
| STM2427_1753_1787 | 0.79 | -1.07 | 1253949lligAlNAD-dependent DNA ligase LigAl- |
| STM2428_97_131 | 0.05 | 1.25 | 1253950lzipAlcell division protein ZipAl- |
| STM2429_649_683 | 0.94 | 1.01 | 1253951lcysZlputative sulfate transport protein CysZl+ |
| STM2430_265_299 | 0.28 | 1.59 | 1253952lcysKlcysteine synthase Al+ |

| | | | |
|---|---|---|---|
| STM2431_209_243 | 0.34 | 1.21 | 1253953lptsHlphosphohistidinoprotein-hexose phosphotransferase component of PTS system (Hpr)l+ |
| STM2432_1657_1691 | 0.01 | 1.53 | 1253954lptsIlphosphoenolpyruvate-protein phosphotransferasel+ |
| STM2433_241_275 | 0.27 | 1.27 | 1253955lcrrlglucose-specific PTS system componentl+ |
| STM2434_123_157 | 0.02 | 1.90 | 1253956lSTM2434lputative cytoplasmic proteinl- |
| STM2435_321_355 | 0.24 | 1.45 | 1253957lpdxKlpyridoxal kinasel- |
| STM2436_422_456 | 0.94 | 1.02 | 1253958lptsJlputative regulatory proteinl+ |
| STM2437_129_163 | 0.01 | 1.62 | 1253959lyfeJlglutamine amidotransferasel+ |
| STM2438_141_175 | 0.02 | 2.15 | 1253960lyfeKlhypothetical proteinl+ |
| STM2439_89_123 | 0.64 | 1.15 | 1253961lyfeLlputative membrane carboxypeptidasel+ |
| STM2440_849_883 | 0.06 | -1.84 | 1253962lcysMlcysteine synthase Bl- |
| STM2441_163_197 | 0.91 | -1.05 | 1253963lcysAlsulfate/thiosulfate transporter subunitl- |
| STM2442_105_139 | 0.59 | 1.27 | 1253964lcysWlsulfate/thiosulfate transporter permease subunitl- |
| STM2443_425_459 | 0.15 | 1.93 | 1253965lcysUlsulfate/thiosulfate transporter subunitl- |
| STM2444_338_372 | 0.18 | 1.93 | 1253966lcysPlthiosulfate transporter subunitl- |
| STM2445_513_547 | 0.00 | -8.21 | 1253967lucpAlshort chain dehydrogenasel- |
| STM2446_113_147 | 0.16 | -1.28 | 1253968lSTM2446lputative iron-dependent peroxidasel- |
| STM2447_417_451 | 0.02 | 1.85 | 1253969lSTM2447lhypothetical proteinl- |
| STM2448_385_419 | 0.04 | 1.34 | 1253970lyfeZlputative inner membrane proteinl- |
| STM2449.S_105_139 | 0.09 | 1.46 | 1253971lSTM2449.Slputative acetyltransferasel- |
| STM2450_705_739 | 0.13 | 1.45 | 1253972lamiAlN-acetylmuramoyl-l-alanine amidase IIl+ |
| STM2451_617_651 | 0.10 | 1.78 | 1253973lhemFlcoproporphyrinogen III oxidasel+ |
| STM2453_983_1017 | 0.72 | 1.03 | 1253975lSTM2453lputative cytoplasmic proteinl+ |
| STM2454_118_152 | 0.01 | -1.97 | 1253976leutRltranscriptional regulator EutRl- |
| STM2455_81_115 | 0.01 | -1.76 | 1253977leutKlputative carboxysome structural proteinl- |
| STM2456_545_579 | 0.01 | -1.80 | 1253978leutLlputative carboxysome structural proteinl- |
| STM2457_301_335 | 0.69 | 1.14 | 1253979leutClethanolamine ammonia-lyase small subunitl- |
| STM2458_1067_1101 | 0.18 | -1.09 | 1253980leutBlethanolamine ammonia-lyase heavy chainl- |
| STM2459_597_631 | 0.61 | 1.07 | 1253981leutAlreactivating factor for ethanolamine ammonia lyasel- |
| STM2460_1092_1126 | 0.40 | 1.11 | 1253982leutHlputative transport proteinl- |
| STM2461_933_967 | 0.19 | 1.12 | 1253983leutGlputative transport proteinl- |
| STM2462_345_379 | 0.05 | -1.35 | 1253984leutJlethanolamine utilization proteinl- |
| STM2464_233_267 | 0.06 | -1.10 | 1253986leutNlputative detox proteinl- |
| STM2465_37_71 | 0.03 | -1.44 | 1253987leutMlputative detox proteinl- |
| STM2466_346_380 | 0.06 | -1.68 | 1253988leutDlphosphotransacetylasel- |
| STM2468_497_531 | 0.17 | -1.42 | 1253990leutQlputative ethanolamine utilization proteinl- |

| | | | |
|---|---|---|---|
| STM2469_233_267 | 0.02 | 1.85 | 1253991\|eutP\|putative ethanolamine utilization protein\|- |
| STM2470_177_211 | 0.11 | 1.19 | 1253992\|eutS\|putative carboxysome structural protein\|- |
| STM2472_2177_2211 | 0.00 | -3.24 | 1253994\|maeB\|malic enzyme\|- |
| STM2473_321_355 | 0.04 | 1.57 | 1253995\|talA\|transaldolase A\|+ |
| STM2474_1642_1676 | 0.14 | -1.22 | 1253996\|tktB\|transketolase\|+ |
| STM2475_161_195 | 0.01 | 1.98 | 1253997\|STM2475\|putative cytoplasmic protein\|- |
| STM2476_557_591 | 0.13 | 2.04 | 1253998\|ypfG\|putative periplasmic protein\|- |
| STM2477_257_291 | 0.56 | 1.12 | 1253999\|yffH\|putative pyrophosphohydrolase\|- |
| STM2478_588_622 | 0.09 | -1.26 | 1254000\|STM2478\|hypothetical protein\|+ |
| STM2479_1379_1413 | 0.08 | 1.88 | 1254001\|aegA\|putative oxidoreductase Fe-S binding subunit\|- |
| STM2480_846_880 | 0.14 | -1.52 | 1254002\|narQ\|nitrate/nitrite sensor protein NarQ\|+ |
| STM2481_2307_2341 | 0.03 | -1.49 | 1254003\|acrD\|aminoglycoside/multidrug efflux system\|+ |
| STM2482_241_275 | 0.50 | 1.03 | 1254004\|yffB\|hypothetical protein\|+ |
| STM2483_721_755 | 0.06 | -1.41 | 1254005\|dapE\|succinyl-diaminopimelate desuccinylase\|+ |
| STM2484_41_76 | 0.05 | -1.48 | 1254006\|STM2484\|putative inner membrane protein\|+ |
| STM2485_1256_1290 | 0.04 | -1.24 | 1254007\|ypfI\|putative acetyltransferase\|- |
| STM2486_529_563 | 0.12 | 1.28 | 1254008\|STM2486\|putative inner membrane protein\|- |
| STM2487_65_99 | 0.02 | -4.24 | 1254009\|purC\|phosphoribosylaminoimidazole-succinocarboxamide synthase\|- |
| STM2488_692_726 | 0.24 | -1.12 | 1254010\|nlpB\|lipoprotein\|- |
| STM2489_257_291 | 0.66 | -1.02 | 1254011\|dapA\|dihydrodipicolinate synthase\|- |
| STM2490_161_195 | 0.04 | -1.21 | 1254012\|gcvR\|glycine cleavage system transcriptional repressor\|+ |
| STM2491_21_55 | 0.03 | 1.80 | 1254013\|bcp\|thioredoxin-dependent thiol peroxidase\|+ |
| STM2492_161_195 | 0.00 | -1.46 | 1254014\|STM2492\|putative glycerate kinase\|- |
| STM2493_997_1031 | 0.01 | -1.51 | 1254015\|perM\|putative permease\|- |
| STM2494_849_883 | 0.21 | 1.28 | 1254016\|STM2494\|hypothetical protein\|+ |
| STM2495_249_283 | 0.62 | -1.08 | 1254017\|yfgD\|putative arsenate reductase\|+ |
| STM2496_257_291 | 0.63 | -1.05 | 1254018\|yfgE\|DNA replication initiation factor\|- |
| STM2497_803_837 | 0.95 | -1.01 | 1254019\|uraA\|uracil transporter\|- |
| STM2498_65_99 | 0.01 | 1.71 | 1254020\|upp\|uracil phosphoribosyltransferase\|- |
| STM2499.S_790_824 | 0.00 | -4.88 | 1254021\|purM\|phosphoribosylaminoimidazole synthetase\|+ |
| STM2500_481_515 | 0.01 | -2.41 | 1254022\|purN\|phosphoribosylglycinamide formyltransferase\|+ |
| STM2501_1132_1166 | 0.04 | 1.39 | 1254023\|ppk\|polyphosphate kinase\|+ |
| STM2502_607_641 | 0.54 | -1.05 | 1254024\|ppx\|exopolyphosphatase\|+ |
| STM2506_34_68 | 0.01 | 2.03 | 1254028\|STM2506\|putative inner membrane protein\|+ |
| STM2508_257_291 | 0.05 | 1.21 | 1254030\|STM2508\|putative cytoplasmic protein\|+ |
| STM2509_25_59 | 0.01 | 1.95 | 1254031\|STM2509\|putative transposase\|- |

| | | | |
|---|---|---|---|
| STM2510_1379_1413 | 0.17 | -1.36 | 1254032lguaAlbifunctional GMP synthase/glutamine amidotransferase proteinl- |
| STM2511_1236_1270 | 0.05 | -4.01 | 1254033lguaBlinositol-5-monophosphate dehydrogenasel- |
| STM2512_959_993 | 0.04 | 2.21 | 1254034lxseAlexodeoxyribonuclease VII large subunitl+ |
| STM2513_5569_5603 | 0.08 | -1.23 | 1254035lshdAlAIDA autotransporter-like proteinl- |
| STM2514_6885_6919 | 0.51 | -1.20 | 1254036lratBlputative outer membrane proteinl- |
| STM2515_4983_5017 | 0.10 | 1.23 | 1254037lratAlputative outer membrane proteinl- |
| STM2518_65_99 | 0.09 | 1.23 | 1254040lyfgJlputative cytoplasmic proteinl- |
| STM2519_1306_1340 | 0.93 | -1.02 | 1254041lengAlGTP-binding protein EngAl- |
| STM2520_780_814 | 0.06 | -1.63 | 1254042lyfgLlouter membrane protein assembly complex subunit YfgLl- |
| STM2521_553_587 | 0.90 | -1.02 | 1254043lyfgMlputative inner membrane proteinl- |
| STM2522_404_438 | 0.21 | -1.23 | 1254044lhisSlhistidyl-tRNA synthetasel- |
| STM2523_120_154 | 0.81 | -1.05 | 1254045lispGl4-hydroxy-3-methylbut-2-en-1-yl diphosphate synthasel- |
| STM2524_934_968 | 0.07 | -1.25 | 1254046lyfgAlhypothetical proteinl- |
| STM2525_1096_1130 | 0.03 | -2.09 | 1254047lyfgBlhypothetical proteinl- |
| STM2526_321_355 | 0.00 | -7.48 | 1254048lndklnucleoside diphosphate kinasel- |
| STM2527_177_211 | 0.83 | -1.03 | 1254049lSTM2527lputative polyferredoxinl- |
| STM2528_577_611 | 0.03 | -1.44 | 1254050lSTM2528lputative dimethylsulfoxide reductasel- |
| STM2529_489_523 | 0.03 | 1.32 | 1254051lSTM2529lputative anaerobic dimethylsulfoxide reductasel- |
| STM2530_2079_2113 | 0.11 | -1.21 | 1254052lSTM2530lputative anaerobic dimethylsulfoxide reductasel- |
| STM2531_1445_1479 | 0.02 | -1.62 | 1254053lpbpClpenicillin-binding protein 1Cl- |
| STM2532_4096_4130 | 0.00 | -2.75 | 1254054lSTM2532lputative inner membrane lipoproteinl- |
| STM2533_609_643 | 0.17 | -1.46 | 1254055lsseAl3-mercaptopyruvate sulfurtransferasel+ |
| STM2534_513_547 | 0.05 | 2.77 | 1254056lSTM2534lputative cytoplasmic proteinl+ |
| STM2535_385_419 | 0.38 | -1.22 | 1254057lsseBlenhanced serine sensitivity proteinl- |
| STM2536_301_335 | 0.71 | -1.05 | 1254058lpepBlaminopeptidase Bl- |
| STM2537_65_99 | 0.03 | 3.31 | 1254059lyfhJlhypothetical proteinl- |
| STM2538_297_331 | 0.04 | 4.09 | 1254060lfdxlelectron carrer proteinl- |
| STM2539_1060_1094 | 0.03 | 3.82 | 1254061lhscAlchaperone protein HscAl- |
| STM2540_273_307 | 0.04 | 4.00 | 1254062lhscBlco-chaperone HscBl- |
| STM2541_169_203 | 0.04 | 3.10 | 1254063liscAliron-sulfur cluster assembly proteinl- |
| STM2542_57_91 | 0.02 | 7.57 | 1254064lnifUlscaffold proteinl- |
| STM2543_1112_1146 | 0.04 | 4.25 | 1254065lnifSlcysteine desulfurasel- |
| STM2544_17_51 | 0.01 | 5.74 | 1254066lyfhPlDNA-binding transcriptional repressorl- |
| STM2545_385_419 | 0.06 | 1.55 | 1254067lSTM2545lputative rRNA methylasel- |
| STM2546_673_707 | 0.04 | -1.63 | 1254068lsuhBlinositol monophosphatasel+ |
| STM2547_385_419 | 0.10 | 1.15 | 1254069lSTM2547lputative hydrolasel+ |

| | | | |
|---|---|---|---|
| STM2548_717_751 | 0.02 | 1.70 | 1254070lasrAlanaerobic sulfide reductasel+ |
| STM2549_577_611 | 0.47 | 1.12 | 1254071lasrBlanaerobic sulfite reductase subunit Bl+ |
| STM2550_271_305 | 0.06 | 1.41 | 1254072lasrClanaerobic sulfide reductasel+ |
| STM2551_689_723 | 0.06 | -1.57 | 1254073lSTM2551lputative inner membrane proteinl- |
| STM2552_353_387 | 0.49 | -1.08 | 1254074lSTM2552lhypothetical proteinl- |
| STM2553_855_889 | 0.00 | -4.52 | 1254075lcsiElstationary phase inducible protein CsiEl+ |
| STM2554_157_191 | 0.02 | -1.35 | 1254076lhcaTlputative 3-phenylpropionic acid transporterl- |
| STM2555_1063_1097 | 0.02 | -1.67 | 1254077lglyAlserine hydroxymethyltransferasel- |
| STM2556_264_298 | 0.00 | -3.03 | 1254078lhmpAlnitric oxide dioxygenasel+ |
| STM2557_1458_1492 | 0.17 | 1.36 | 1254079lcadClDNA-binding transcriptional activatorl+ |
| STM2558_1117_1151 | 0.92 | 1.01 | 1254080lcadBlpredicted lysine/cadaverine transporterl+ |
| STM2559_1498_1532 | 0.00 | 2.84 | 1254081lcadAllysine decarboxylase 1l+ |
| STM2561_209_243 | 0.67 | -1.09 | 1254083lglnBlregulatory protein P-IIl- |
| STM2562_467_501 | 0.36 | -1.14 | 1254084lyfhAlputative transcriptional regulatorl- |
| STM2563_689_723 | 0.91 | -1.03 | 1254085lyfhGlhypothetical proteinl- |
| STM2564_1196_1230 | 0.01 | 1.30 | 1254086lyfhKlputative sensor kinasel- |
| STM2566_97_131 | 0.03 | 1.73 | 1254088lSTM2566lputative periplasmic proteinl+ |
| STM2567_802_836 | 0.01 | 1.57 | 1254089lyfhDlputative transglycosylasel+ |
| STM2568_257_291 | 0.50 | -1.08 | 1254090lyfhCltRNA-specific adenosine deaminasel- |
| STM2569_289_323 | 0.03 | 1.59 | 1254091lyfhBlhypothetical proteinl- |
| STM2570_877_911 | 0.02 | 1.51 | 1254092lSTM2570lputative phosphotransferase system IIB componentl- |
| STM2571_17_51 | 0.12 | -1.23 | 1254093lmurQlN-acetylmuramic acid-6-phosphate etherasel- |
| STM2572_225_259 | 0.02 | 1.80 | 1254094lyfhHlputative DNA-binding transcriptional regulatorl+ |
| STM2573_577_611 | 0.11 | -1.25 | 1254095lSTM2573l2-dehydropantoate 2-reductasel- |
| STM2574_582_616 | 0.14 | 1.32 | 1254096lSTM2574lputative permeasel- |
| STM2575_641_675 | 0.37 | 1.07 | 1254097lSTM2575lputative transcriptional regulatorl+ |
| STM2576_97_131 | 0.01 | -1.74 | 1254098lyfhLlputative ferredoxinl+ |
| STM2577_17_51 | 0.70 | 1.07 | 1254099lacpSl4'-phosphopantetheinyl transferasel- |
| STM2578_385_419 | 0.36 | 1.16 | 1254100lpdxJlpyridoxal phosphate biosynthetic protein PdxJl- |
| STM2579_521_555 | 0.13 | 1.07 | 1254101lrecOlDNA repair protein RecOl- |
| STM2580_17_51 | 0.24 | -1.14 | 1254102leralGTP-binding protein Eral- |
| STM2581_337_371 | 0.57 | -1.07 | 1254103lrnclribonuclease IIIl- |
| STM2582_545_579 | 0.01 | 1.34 | 1254104llepBlsignal peptidase Il- |
| STM2583_1249_1283 | 0.27 | -1.17 | 1254105llepAlGTP-binding protein LepAl- |
| STM2584_1339_1374 | 0.01 | 2.66 | 1254106lgogBlleucine-rich repeat proteinl+ |
| STM2585_489_523 | 0.01 | 5.76 | 1254107lSTM2585ltransposase-like proteinl+ |

| | | | |
|---|---|---|---|
| STM2585A_148_183 | 0.00 | 3.72 | 1254108lSTM2585AlPagK-like proteinl+ |
| STM2586_436_471 | 0.07 | 2.39 | 1254109lSTM2586lphage tail assembly-like proteinl- |
| STM2587_105_139 | 0.08 | 1.59 | 1254110lSTM2587lphage tail assembly-like proteinl- |
| STM2589_2890_2924 | 0.04 | 1.41 | 1254112lSTM2589lhost specificity protein-J-likel- |
| STM2590_177_211 | 0.06 | 1.69 | 1254113lSTM2590ltail assembly protein I-likel- |
| STM2591_293_327 | 0.10 | 1.42 | 1254114lSTM2591ltail assembly protein K-likel- |
| STM2592_385_419 | 0.05 | 1.78 | 1254115lSTM2592lphage tail component L-like proteinl- |
| STM2593_113_147 | 0.06 | 1.52 | 1254116lSTM2593lphage tail component M-like proteinl- |
| STM2594_2615_2649 | 0.09 | 1.79 | 1254117lSTM2594lphage tail component H-like proteinl- |
| STM2595_161_195 | 0.02 | 2.87 | 1254118lSTM2595lminor tail-like proteinl- |
| STM2596_92_126 | 0.26 | 1.55 | 1254119lSTM2596lminor tail-like proteinl- |
| STM2597_375_409 | 0.02 | 2.37 | 1254120lSTM2597lmajor tail-like proteinl- |
| STM2598_129_163 | 0.02 | 2.37 | 1254121lSTM2598lhypothetical proteinl- |
| STM2599_612_646 | 0.03 | 1.85 | 1254122lSTM2599lputative virulence proteinl+ |
| STM2600_321_355 | 0.02 | 2.39 | 1254123lSTM2600lminor tail protein Z-likel- |
| STM2601_220_254 | 0.02 | 2.21 | 1254124lSTM2601lminor capsid protein FIIl- |
| STM2602_193_227 | 0.05 | 2.21 | 1254125lSTM2602lDNA packaging-like proteinl- |
| STM2603_606_640 | 0.03 | 2.39 | 1254126lSTM2603lphage head-like proteinl- |
| STM2604_7_41 | 0.07 | 1.82 | 1254127lSTM2604lphage head-like proteinl- |
| STM2605_674_708 | 0.27 | 1.47 | 1254128lSTM2605lhead-tail preconnector-like proteinl- |
| STM2606_1446_1480 | 0.05 | 1.82 | 1254129lSTM2606lhead-tail preconnector-like proteinl- |
| STM2607_81_115 | 0.01 | 1.55 | 1254130lSTM2607lhead-to-tail joining-like proteinl- |
| STM2608_1749_1783 | 0.05 | 2.01 | 1254131lSTM2608lterminase-like large proteinl- |
| STM2610_201_235 | 0.54 | 1.12 | 1254133lSTM2610lhypothetical proteinl+ |
| STM2611.S_78_113 | 0.02 | 2.01 | 1254134lSTM2611.Slendopeptidase-like proteinl- |
| STM2612_357_391 | 0.16 | 1.35 | 1254135lSTM2612lmorphogenesis-like proteinl- |
| STM2613_88_122 | 0.02 | 2.82 | 1254136lSTM2613lhypothetical proteinl- |
| STM2614_99_133 | 0.08 | 1.30 | 1254137lSTM2614lhypothetical proteinl+ |
| STM2615_25_59 | 0.01 | 2.47 | 1254138lSTM2615ltRNAl- |
| STM2616_441_475 | 0.33 | -1.15 | 1254139lSTM2616lantirepressor-like proteinl- |
| STM2617_289_323 | 0.07 | 1.79 | 1254140lSTM2617lantiterminator-like proteinl- |
| STM2618_73_107 | 0.11 | 1.61 | 1254141lSTM2618lhypothetical proteinl- |
| STM2619_439_473 | 0.03 | 2.61 | 1254142lSTM2619lhypothetical proteinl- |
| STM2620_532_566 | 0.69 | 1.07 | 1254143lSTM2620lhypothetical proteinl- |
| STM2622_56_95 | 0.04 | 1.43 | 1254145lSTM2622lhypothetical proteinl- |
| STM2629_148_183 | 0.02 | 2.00 | 1254152lSTM2629lhypothetical proteinl+ |

| | | | |
|---|---|---|---|
| STM2630_108_142 | 0.12 | 1.30 | 1254153lSTM2630lhypothetical proteinl+ |
| STM2631_180_214 | 0.03 | 1.26 | 1254154lSTM2631lhypothetical proteinl+ |
| STM2635_81_115 | 0.05 | 1.86 | 1254158lSTM2635lexcisionase-like proteinl+ |
| STM2636_1031_1065 | 0.40 | -1.03 | 1254159lSTM2636lintegrase-like proteinl- |
| STM2637_121_155 | 0.01 | 2.36 | 1254160lrseClSoxR reducing system protein RseCl- |
| STM2638_769_803 | 0.55 | 1.06 | 1254161lrseBlperiplasmic negative regulator of sigmaEl- |
| STM2639_193_227 | 0.08 | 1.28 | 1254162lrseAlanti-RNA polymerase sigma factor SigEl- |
| STM2640_417_451 | 0.05 | 1.24 | 1254163lrpoElRNA polymerase sigma factor RpoEl- |
| STM2641_912_946 | 0.78 | -1.06 | 1254164lnadBlL-aspartate oxidasel+ |
| STM2642_449_483 | 0.07 | -1.37 | 1254165lyfiClputative transferasel- |
| STM2643_504_538 | 0.02 | -1.47 | 1254166lsrmBlATP-dependent RNA helicase SrmBl+ |
| STM2644_529_563 | 0.00 | 2.10 | 1254167lyfiElputative transcriptional regulatorl- |
| STM2645_97_131 | 0.17 | -1.58 | 1254168lyfiKlneutral amino-acid efflux proteinl+ |
| STM2647_353_387 | 0.45 | 1.09 | 1254170lunglu racil-DNA glycosylasel+ |
| STM2648_839_873 | 0.86 | -1.02 | 1254171lyfiFlpredicted methyltransferasel- |
| STM2649_129_163 | 0.03 | 1.53 | 1254172ltrxClthioredoxin 2l+ |
| STM2650_529_563 | 0.00 | -1.62 | 1254173lyfiPlputative cytoplasmic proteinl+ |
| STM2651_2494_2528 | 0.05 | -1.57 | 1254174lyfiQlputative acetyl-CoA synthetasel+ |
| STM2652_421_455 | 0.00 | -2.25 | 1254175lpssAlphosphatidylserine synthasel+ |
| STM2653_101_135 | 0.00 | -1.66 | 1254176lyfiMlhypothetical proteinl+ |
| STM2654_815_849 | 0.02 | -1.64 | 1254177lkgtPlalpha-ketoglutarate transporterl- |
| STM2655_401_435 | 0.16 | 1.58 | 1254178lSTM2655lputative cytoplasmic proteinl- |
| STM2660_1719_1753 | 0.05 | 1.65 | 1254183lclpBlprotein disaggregation chaperonel- |
| STM2661_251_285 | 0.02 | 1.66 | 1254184lyfiHlhypothetical proteinl- |
| STM2662_129_163 | 0.73 | 1.08 | 1254185lrluDl23S rRNA pseudouridine synthase Dl- |
| STM2663_465_499 | 0.90 | 1.01 | 1254186lyfiOlpredicted lipoproteinl+ |
| STM2664_45_79 | 0.04 | 3.08 | 1254187lSTM2664lpseudol+ |
| STM2665_33_67 | 0.01 | -1.78 | 1254188lyfiAltranslation inhibitor protein RaiAl+ |
| STM2667_482_516 | 0.01 | 1.14 | 1254190lpheAlbifunctional chorismate mutase/prephenate dehydratasel+ |
| STM2669_507_541 | 0.86 | -1.10 | 1254192ltyrAlbifunctional chorismate mutase/prephenate dehydrogenasel- |
| STM2670_968_1002 | 0.61 | -1.36 | 1254193laroFlphospho-2-dehydro-3-deoxyheptonate aldolasel- |
| STM2671_97_131 | 0.04 | -1.44 | 1254194lyfiRlputative periplasmic proteinl+ |
| STM2672_926_960 | 0.06 | 1.23 | 1254195lyfiNlputative diguanylate cyclase/phosphodiesterasel+ |
| STM2673_289_323 | 0.02 | -2.03 | 1254196lrplSl50S ribosomal protein L19l- |
| STM2674_385_419 | 0.02 | -2.95 | 1254197ltrmDltRNA (guanine-N(1)-)-methyltransferasel- |
| STM2675_353_387 | 0.02 | -2.62 | 1254198lrimMl16S rRNA-processing proteinl- |

| | | | |
|---|---|---|---|
| STM2676_49_83 | 0.02 | -1.42 | 1254199\|rpsP\|30S ribosomal protein S16\|- |
| STM2677_971_1005 | 0.09 | 1.32 | 1254200\|ffh\|signal recognition particle protein\|- |
| STM2678_561_595 | 0.34 | -1.27 | 1254201\|corE\|hypothetical protein\|+ |
| STM2679_454_488 | 0.31 | 1.40 | 1254202\|yfjD\|hypothetical protein\|+ |
| STM2680_177_211 | 0.01 | 1.33 | 1254203\|STM2680\|putative cytoplasmic protein\|+ |
| STM2681_257_291 | 0.49 | 1.26 | 1254204\|grpE\|heat shock protein GrpE\|- |
| STM2682_7_41 | 0.03 | 1.94 | 2673728\|STM2682\|-\|- |
| STM2683_641_675 | 0.02 | 1.91 | 1254206\|ppnK\|inorganic polyphosphate/ATP-NAD kinase\|+ |
| STM2684_1159_1193 | 0.03 | 2.10 | 1254207\|recN\|recombination and repair protein\|+ |
| STM2685_169_203 | 0.06 | 1.32 | 1254208\|smpA\|hypothetical protein\|+ |
| STM2686_216_251 | 0.02 | 1.62 | 1254209\|yfjF\|hypothetical protein\|- |
| STM2687_193_227 | 0.01 | 2.42 | 1254210\|yfjG\|hypothetical protein\|- |
| STM2688_305_339 | 0.01 | 1.68 | 1254211\|smpB\|SsrA-binding protein\|+ |
| STM2689_7512_7546 | 0.02 | -3.37 | 1254212\|STM2689\|pseudo\|+ |
| STM2690_1371_1405 | 0.04 | -2.76 | 1254213\|STM2690\|putative outer membrane efflux protein\|+ |
| STM2691_1346_1380 | 0.07 | -1.67 | 1254214\|STM2691\|putative ABC transporter transmembrane region\|+ |
| STM2694_181_215 | 0.01 | 2.11 | 1254217\|STM2694\|late control-like protein\|- |
| STM2695_934_968 | 0.36 | 1.19 | 1254218\|STM2695\|late control-like protein\|- |
| STM2696_25_59 | 0.46 | 1.05 | 1254219\|STM2696\|putative phage tail protein\|- |
| STM2697_2577_2611 | 0.68 | 1.08 | 1254220\|STM2697\|phage tail-like protein\|- |
| STM2698_33_67 | 0.67 | 1.22 | 1254221\|STM2698\|gpE-like protein\|- |
| STM2700_417_451 | 0.23 | 1.11 | 1254223\|STM2700\|phage tail fiber-like protein\|- |
| STM2702_205_239 | 0.24 | 1.19 | 1254225\|STM2702\|DNA invertase-like protein\|- |
| STM2705_369_403 | 0.80 | -1.03 | 1254228\|STM2705\|hypothetical protein\|- |
| STM2707_417_451 | 0.82 | -1.05 | 1254230\|STM2707\|phage tail-like protein\|- |
| STM2711_182_216 | 0.23 | -1.31 | 1254234\|STM2711\|phage tail-like protein\|- |
| STM2712_113_147 | 0.07 | -1.64 | 1254235\|STM2712\|phage tail-like protein\|- |
| STM2713_193_227 | 0.86 | 1.03 | 1254236\|STM2713\|hypothetical protein\|- |
| STM2714_153_187 | 0.20 | 1.44 | 1254237\|STM2714\|lysis-like protein\|- |
| STM2715.S_27_61 | 0.48 | 1.21 | 1254238\|STM2715.S\|probable prophage lysozyme\|- |
| STM2717_67_101 | 0.00 | -1.41 | 1254240\|STM2717\|phage tail-like protein\|- |
| STM2718_161_195 | 0.04 | -1.28 | 1254241\|STM2718\|head completion-like protein\|- |
| STM2719_457_491 | 0.07 | 1.91 | 1254242\|STM2719\|terminase-like protein\|- |
| STM2720_532_566 | 0.17 | 1.41 | 1254243\|STM2720\|major capsid-like protein\|- |
| STM2722_1472_1506 | 0.01 | 2.12 | 1254245\|STM2722\|terminase-like protein\|+ |
| STM2725_47_81 | 0.03 | -1.57 | 1254248\|STM2725\|pseudo\|- |

| | | | |
|---|---|---|---|
| STM2726_241_275 | 0.07 | -1.54 | 1254249lSTM2726lhypothetical proteinl- |
| STM2728_105_139 | 0.07 | -1.61 | 1254251lSTM2728lhypothetical proteinl- |
| STM2729_2367_2401 | 0.16 | -1.26 | 1254252lSTM2729lhypothetical proteinl- |
| STM2730_513_547 | 0.48 | -1.05 | 1254253lSTM2730lDNA adenine methylase-like proteinl- |
| STM2732_133_167 | 0.01 | -1.66 | 1254255lSTM2732lhypothetical proteinl- |
| STM2734_145_179 | 0.58 | -1.08 | 1254257lSTM2734lhypothetical proteinl+ |
| STM2737_153_187 | 0.25 | -1.27 | 1254260lSTM2737lhypothetical proteinl- |
| STM2738_33_67 | 0.24 | -1.37 | 1254261lSTM2738lhypothetical proteinl+ |
| STM2740_226_260 | 0.00 | -3.52 | 1254263lSTM2740lintegrase-like proteinl+ |
| STM2740.1N_37_71 | 0.00 | -18.32 | 2673740lSTM2740.1Nlhypothetical proteinl+ |
| STM2741_465_499 | 0.00 | -5.60 | 1254264lSTM2741lputative periplasmic proteinl+ |
| STM2742_675_709 | 0.03 | -1.30 | 1254265lSTM2742lputative cytoplasmic proteinl+ |
| STM2743_57_91 | 0.02 | 1.55 | 1254266lSTM2743lputative cytoplasmic proteinl- |
| STM2745_1515_1549 | 0.15 | -1.29 | 1254268lSTM2745lputative inner membrane proteinl+ |
| STM2746_794_830 | 0.34 | 1.18 | 1254269lSTM2746lputative ATPasel+ |
| STM2747_637_674 | 0.02 | 2.17 | 1254270lSTM2747lputative cytoplasmic proteinl+ |
| STM2748_193_227 | 0.51 | 1.16 | 1254271lSTM2748lputative transcriptional regulatorl- |
| STM2749_97_131 | 0.05 | 1.15 | 1254272lSTM2749lputative cytoplasmic proteinl+ |
| STM2750_443_477 | 0.50 | 1.11 | 1254273lSTM2750lputative PTS system glucitol/sorbitol-specific enzyme IIl+ |
| STM2751_97_131 | 0.84 | -1.03 | 1254274lSTM2751lputative glucitol-specific PTS enzyme IIIl+ |
| STM2752_97_131 | 0.27 | -1.18 | 1254275lSTM2752lputative glucitol-specific PTS enzyme IIIl+ |
| STM2753_751_785 | 0.94 | 1.01 | 1254276lSTM2753lputative dehydrogenasel+ |
| STM2754_686_720 | 0.02 | 2.41 | 1254277lSTM2754lputative hexulose 6 phosphate synthasel+ |
| STM2755_161_195 | 0.23 | -1.21 | 1254278lSTM2755lputative hexulose 6 phosphate synthasel- |
| STM2756_497_531 | 0.02 | 1.54 | 1254279lSTM2756lputative sugar phosphate aminotransferasel- |
| STM2759_1417_1451 | 0.03 | -1.91 | 1254282lSTM2759lputative dipeptide/oligopeptide/nickel ABC-type transport system periplasmic componentl+ |
| STM2760_353_387 | 0.02 | 1.29 | 1254283lSTM2760lputative integrasel+ |
| STM2761_1252_1286 | 0.05 | 1.30 | 1254284lSTM2761lputative inner membrane proteinl+ |
| STM2762_685_723 | 0.01 | 2.41 | 1254285lSTM2762lputative inner membrane proteinl- |
| STM2763_417_451 | 0.02 | 1.63 | 2673753lSTM2763l-l+ |
| STM2764_239_274 | 0.01 | 1.57 | 2673748lSTM2764l-l- |
| STM2766_89_127 | 0.05 | 1.58 | 1254289lSTM2766lputative cytoplasmic proteinl- |
| STM2767_1688_1722 | 0.12 | 1.48 | 1254290lSTM2767lputative DNA/RNA helicasel- |
| STM2768_195_229 | 0.02 | 1.59 | 1254291lSTM2768lputative transposasel+ |
| STM2769_435_469 | 0.75 | -1.08 | 1254292lSTM2769lputative transposasel+ |

| | | | |
|---|---|---|---|
| STM2770_49_83 | 0.03 | 1.55 | 1254293\|fljA\|phase-1 flagellin repressor\|- |
| STM2771_1354_1388 | 0.58 | -1.08 | 1254294\|fljB\|flagellin\|- |
| STM2772_221_257 | 0.28 | -1.16 | 1254295\|hin\|DNA-invertase Hin\|+ |
| STM2773_341_375 | 0.23 | -1.13 | 1254296\|iroB\|putative glycosyl transferase\|+ |
| STM2774_2943_2977 | 0.94 | 1.02 | 1254297\|iroC\|putative ABC transporter protein\|+ |
| STM2775_630_664 | 0.02 | -1.72 | 1254298\|iroD\|enterochelin esterase=-like protein\|+ |
| STM2776_321_355 | 0.25 | 1.21 | 1254299\|iroE\|putative hydrolase\|+ |
| STM2777_2014_2048 | 0.20 | 1.46 | 1254300\|iroN\|TonB-dependent siderophore receptor protein\|- |
| STM2778_39_73 | 0.35 | 1.17 | 1254301\|STM2778\|pseudo\|+ |
| STM2780_470_504 | 0.00 | 2.33 | 1254303\|pipB2\|secreted effector protein\|- |
| STM2781_561_595 | 0.01 | 4.13 | 1254304\|virK\|virulence protein\|+ |
| STM2782_729_763 | 0.03 | 2.23 | 1254305\|mig-14\|putative transcriptional activator\|+ |
| STM2783_175_209 | 0.04 | -1.59 | 1254306\|nixA\|putative nickel transporter\|- |
| STM2784_609_643 | 0.00 | -2.54 | 1254307\|tctE\|regulatory protein\|- |
| STM2785_465_499 | 0.01 | -2.66 | 1254308\|tctD\|regulatory protein\|- |
| STM2786_113_147 | 0.00 | -3.64 | 1254309\|STM2786\|tricarboxylic transport\|+ |
| STM2787_3_37 | 0.00 | -6.24 | 1254310\|STM2787\|tricarboxylic transport\|+ |
| STM2788_1124_1158 | 0.01 | -2.19 | 1254311\|STM2788\|tricarboxylic transport\|+ |
| STM2789_713_747 | 0.01 | -5.05 | 1254312\|STM2789\|hypothetical protein\|+ |
| STM2790_494_528 | 0.00 | -5.64 | 1254313\|ygaF\|hypothetical protein\|+ |
| STM2791_866_900 | 0.01 | -2.12 | 1254314\|gabD\|succinate-semialdehyde dehydrogenase I\|+ |
| STM2792_765_799 | 0.00 | -7.17 | 1254315\|gabT\|4-aminobutyrate aminotransferase\|+ |
| STM2793_786_820 | 0.04 | -1.52 | 1254316\|gabP\|gamma-aminobutyrate transporter\|+ |
| STM2794_505_539 | 0.00 | -2.81 | 1254317\|ygaE\|DNA-binding transcriptional regulator CsiR\|+ |
| STM2795_233_267 | 0.28 | 1.18 | 1254318\|ygaU\|hypothetical protein\|- |
| STM2796_63_97 | 0.17 | 1.53 | 1254319\|yqaE\|putative transport protein\|- |
| STM2797_191_225 | 0.50 | 1.05 | 1254320\|STM2797\|putative regulatory protein\|+ |
| STM2798_75_109 | 0.23 | -1.15 | 1254321\|ygaP\|putative rhodanese-like sulfurtransferase\|+ |
| STM2799_49_83 | 0.02 | 1.77 | 1254322\|stpA\|DNA binding protein  nucleoid-associated\|- |
| STM2801_241_275 | 0.02 | 2.03 | 1254324\|ygaC\|hypothetical protein\|- |
| STM2802_17_52 | 0.05 | 1.35 | 1254325\|ygaM\|hypothetical protein\|+ |
| STM2803_544_578 | 0.76 | -1.04 | 1254326\|STM2803\|putative regulatory protein\|- |
| STM2804.1n_27_61 | 0.02 | 1.40 | 2673735\|STM2804.1n\|hypothetical protein\|+ |
| STM2806_97_131 | 0.49 | -1.31 | 1254329\|nrdI\|ribonucleotide reductase stimulatory protein\|+ |
| STM2807_1338_1372 | 0.16 | -1.54 | 1254330\|nrdE\|ribonucleotide-diphosphate reductase subunit alpha\|+ |
| STM2808_721_755 | 0.35 | -1.18 | 1254331\|nrdF\|ribonucleotide-diphosphate reductase subunit beta\|+ |

| | | | |
|---|---|---|---|
| STM2809_940_974 | 0.62 | 1.07 | 1254332\|proV\|glycine betaine transporter ATP-binding subunit\|+ |
| STM2810_162_196 | 0.39 | 1.11 | 1254333\|proW\|glycine betaine transporter membrane protein\|+ |
| STM2811_641_675 | 0.17 | 1.36 | 1254334\|proX\|glycine betaine transporter periplasmic subunit\|+ |
| STM2812_890_924 | 0.01 | -2.41 | 1254335\|STM2812\|putative inner membrane protein\|+ |
| STM2813_81_115 | 0.04 | -1.38 | 1254336\|emrR\|transcriptional repressor MprA\|+ |
| STM2814_430_464 | 0.04 | 1.30 | 1254337\|emrA\|multidrug resistance secretion protein\|+ |
| STM2815_828_862 | 0.16 | -1.18 | 1254338\|emrB\|putative multidrug transport protein\|+ |
| STM2816_372_406 | 0.02 | 1.44 | 1254339\|STM2816\|putative glycoporin\|- |
| STM2817_9_43 | 0.15 | 1.15 | 1254340\|luxS\|S-ribosylhomocysteinase\|- |
| STM2818_846_880 | 0.09 | -1.30 | 1254341\|gshA\|glutamate--cysteine ligase\|- |
| STM2819_257_291 | 0.05 | -1.52 | 1254342\|yqaA\|putative inner membrane protein\|- |
| STM2820_161_195 | 0.00 | 1.96 | 1254343\|yqaB\|predicted hydrolase\|- |
| STM2821/STM2822/STM282 | 0.11 | -1.52 | 1254344/1254345/1254346/1254347\|STM2821/STM2822/STM2823/STM2824\|tRNA\|- |
| STM2826_9_43 | 0.47 | 1.06 | 1254349\|csrA\|carbon storage regulator\|- |
| STM2827_2032_2066 | 0.03 | -1.74 | 1254350\|alaS\|alanyl-tRNA synthetase\|- |
| STM2828_29_63 | 0.08 | 1.81 | 1254351\|recX\|recombination regulator RecX\|- |
| STM2829_799_833 | 0.12 | 1.56 | 1254352\|recA\|recombinase A\|- |
| STM2830_139_173 | 0.25 | -1.13 | 1254353\|ygaD\|competence damage-inducible protein A\|- |
| STM2831_225_259 | 0.09 | -1.36 | 1254354\|mltB\|membrane-bound lytic murein transglycosylase B; catalyzes the cleavage of the glycosidic bonds between N-acetylmuramic acid and N-acetylglucosamine in peptidoglycan\|- |
| STM2832_257_291 | 0.26 | -1.24 | 1254355\|srlA\|glucitol/sorbitol-specific enzyme IIC component\|+ |
| STM2833_263_297 | 0.06 | 2.19 | 1254356\|srlE\|glucitol/sorbitol-specific enzyme IIB component\|+ |
| STM2834_169_203 | 0.04 | 1.71 | 1254357\|slrB\|glucitol/sorbitol-specific PTS system component IIA\|+ |
| STM2835_705_739 | 0.31 | 1.11 | 1254358\|srlD\|sorbitol-6-phosphate dehydrogenase\|+ |
| STM2836_305_339 | 0.02 | 1.45 | 1254359\|gutM\|DNA-binding transcriptional activator of glucitol operon\|+ |
| STM2837_545_579 | 0.15 | -1.18 | 1254360\|srlR\|DNA-bindng transcriptional repressor\|+ |
| STM2838.S_401_435 | 0.04 | -1.48 | 1254361\|gutQ\|D-arabinose 5-phosphate isomerase\|+ |
| STM2839_810_844 | 0.44 | -1.10 | 1254362\|ygaA\|anaerobic nitric oxide reductase transcription regulator\|- |
| STM2840_1105_1139 | 0.37 | 1.07 | 1254363\|STM2840\|anaerobic nitric oxide reductase flavorubredoxin\|+ |
| STM2841_663_697 | 0.07 | -1.36 | 1254364\|ygbD\|nitric oxide reductase\|+ |
| STM2842_1250_1284 | 0.28 | 1.18 | 1254365\|hypF\|hydrogenase maturation protein\|- |
| STM2843_257_291 | 0.67 | 1.07 | 1254366\|hydN\|electron transport protein HydN\|- |
| STM2844_545_579 | 0.01 | 1.12 | 1254367\|STM2844\|hypothetical protein\|- |
| STM2845_265_299 | 0.06 | 1.15 | 1254368\|hycI\|hydrogenase 3 maturation protease\|- |

| | | | |
|---|---|---|---|
| STM2846_81_115 | 0.02 | 1.28 | 1254369\|hycH\|hydrogenase 3 large subunit processing protein\|- |
| STM2848_209_243 | 0.03 | 1.48 | 1254371\|hycF\|formate hydrogenlyase complex iron-sulfur subunit\|- |
| STM2849_967_1001 | 0.59 | -1.06 | 1254372\|hycE\|hydrogenase 3 large subunit\|- |
| STM2850_257_291 | 0.20 | 1.36 | 1254373\|hycD\|hydrogenase 3 membrane subunit\|- |
| STM2851_1532_1566 | 0.99 | 1.00 | 1254374\|hycC\|formate hydrogenlyase subunit 3\|- |
| STM2853_385_419 | 0.88 | 1.01 | 1254376\|hycA\|transcriptional repressor\|- |
| STM2854_145_179 | 0.04 | 1.39 | 1254377\|hypA\|hydrogenase nickel incorporation protein\|+ |
| STM2855_209_243 | 0.13 | 1.24 | 1254378\|hypB\|hydrogenase nickel incorporation protein HypB\|+ |
| STM2856_73_107 | 0.13 | -1.09 | 1254379\|hypC\|hydrogenase isoenzymes formation protein\|+ |
| STM2857_152_186 | 0.03 | 1.39 | 1254380\|hypD\|putative hydrogenase formation protein\|+ |
| STM2858_716_750 | 0.06 | -1.17 | 1254381\|hypE\|putative hydrogenase formation protein\|+ |
| STM2859_1848_1882 | 0.18 | -1.18 | 1254382\|fhlA\|formate hydrogen-lyase transcriptional activator\|+ |
| STM2860_187_221 | 0.01 | 1.62 | 1254383\|ygbA\|hypothetical protein\|- |
| STM2861_441_475 | 0.91 | 1.02 | 1254384\|sitA\|putative periplasmic binding protein\|+ |
| STM2862_257_291 | 0.02 | 1.58 | 1254385\|sitB\|putative ATP-binding protein\|+ |
| STM2863_129_163 | 0.67 | 1.09 | 1254386\|sitC\|putative permease\|+ |
| STM2864_513_547 | 0.99 | -1.00 | 1254387\|sitD\|putative permease\|+ |
| STM2865_113_147 | 0.02 | 2.16 | 1254388\|avrA\|secreted effector protein\|- |
| STM2866_705_739 | 0.03 | 1.44 | 1254389\|sprB\|transcriptional regulator\|- |
| STM2868_115_149 | 0.18 | -1.35 | 1254391\|orgC\|putative cytoplasmic protein\|- |
| STM2869_545_579 | 0.32 | -1.17 | 1254392\|orgB\|needle complex export protein\|- |
| STM2871_33_67 | 0.45 | 1.07 | 1254394\|prgK\|needle complex inner membrane lipoprotein\|- |
| STM2873_129_163 | 0.01 | -1.54 | 1254396\|prgI\|needle complex major subunit\|- |
| STM2874_308_342 | 0.03 | 2.67 | 1254397\|prgH\|needle complex inner membrane protein\|- |
| STM2875_577_611 | 0.02 | 1.60 | 1254398\|hilD\|invasion protein regulatory protein\|+ |
| STM2876_1455_1489 | 0.02 | 2.93 | 1254399\|hilA\|invasion protein regulator\|+ |
| STM2877_145_179 | 0.08 | 2.03 | 1254400\|iagB\|invasion protein precursor\|+ |
| STM2878_1337_1371 | 0.13 | 1.31 | 1254401\|sptP\|protein tyrosine phosphatase/GTPase activating protein\|- |
| STM2879_253_287 | 0.17 | 1.28 | 1254402\|sicP\|secretion chaparone\|- |
| STM2881_81_115 | 0.02 | 1.51 | 1254404\|iacP\|acyl carrier protein\|- |
| STM2882_1699_1733 | 0.31 | 1.15 | 1254405\|sipA\|secreted effector protein\|- |
| STM2883_425_459 | 0.01 | 2.20 | 1254406\|sipD\|translocation machinery component\|- |
| STM2884_423_457 | 0.12 | -1.18 | 1254407\|sipC\|translocation machinery component\|- |
| STM2885_927_961 | 0.01 | -1.42 | 1254408\|sipB\|translocation machinery component\|- |
| STM2886_409_443 | 0.07 | 1.82 | 1254409\|sicA\|secretion chaperone\|- |
| STM2887_936_970 | 0.14 | 1.13 | 1254410\|spaS\|surface presentation of antigens protein SpaS\|- |

| | | | |
|---|---|---|---|
| STM2888_481_515 | 0.41 | -1.11 | 1254411\|spaR\|needle complex export protein\|- |
| STM2889_193_227 | 0.90 | 1.02 | 1254412\|spaQ\|needle complex export protein\|- |
| STM2890_422_459 | 0.34 | 1.45 | 1254413\|spaP\|surface presentation of antigens protein SpaP\|- |
| STM2891_769_803 | 0.57 | -1.15 | 1254414\|spaO\|surface presentation of antigens protein SpaO\|- |
| STM2892_876_910 | 0.65 | -1.04 | 1254415\|invJ\|needle length control protein\|- |
| STM2895_9_43 | 0.98 | 1.00 | 1254418\|invB\|secretion chaperone\|- |
| STM2896_1795_1829 | 0.95 | 1.01 | 1254419\|invA\|needle complex export protein\|- |
| STM2897_376_410 | 0.19 | 1.13 | 1254420\|invE\|invasion protein\|- |
| STM2898_1346_1380 | 0.20 | -1.26 | 1254421\|invG\|outer membrane secretin precursor\|- |
| STM2899_513_547 | 0.36 | 1.22 | 1254422\|invF\|invasion regulatory protein\|- |
| STM2900_114_148 | 0.03 | 2.30 | 1254423\|invH\|needle complex outer membrane lipoprotein precursor\|+ |
| STM2901_321_355 | 0.63 | -1.05 | 1254424\|STM2901\|putative cytoplasmic protein\|+ |
| STM2902_261_297 | 0.33 | 1.14 | 1254425\|STM2902\|putative cytoplasmic protein\|+ |
| STM2903_281_315 | 0.76 | 1.08 | 1254426\|STM2903\|putative cytoplasmic protein\|- |
| STM2904_169_203 | 0.61 | -1.06 | 1254427\|STM2904\|putative ABC-type transporter\|+ |
| STM2905_145_179 | 0.34 | 1.17 | 1254428\|STM2905\|putative acetyltransferase\|+ |
| STM2906_129_163 | 0.02 | 1.42 | 1254429\|STM2906\|-\|- |
| STM2907_401_435 | 0.18 | 1.22 | 1254430\|pphB\|serine/threonine-specific protein phosphatase 2\|+ |
| STM2908_193_227 | 0.01 | 1.85 | 1254431\|STM2908\|putative cytoplasmic protein\|- |
| STM2909_1697_1731 | 0.07 | 1.29 | 1254432\|mutS\|DNA mismatch repair protein\|+ |
| STM2910_193_227 | 0.89 | 1.05 | 1254433\|STM2910\|putative cytoplasmic protein\|- |
| STM2911_514_548 | 0.59 | -1.27 | 1254434\|STM2911\|putative permease\|- |
| STM2912_321_355 | 0.78 | -1.03 | 1254435\|STM2912\|putative transcriptional regulator\|+ |
| STM2913_946_980 | 0.00 | 1.53 | 1254436\|STM2913\|putative permease\|- |
| STM2915_705_739 | 0.37 | -1.22 | 1254438\|ygbM\|hypothetical protein\|- |
| STM2916_161_195 | 0.03 | -1.65 | 1254439\|ygbL\|putative aldolase\|- |
| STM2917_360_394 | 0.03 | -1.48 | 1254440\|ygbK\|putative tRNA synthase\|- |
| STM2918_193_227 | 0.07 | -1.21 | 1254441\|ygbJ\|3-hydroxyisobutyrate dehydrogenase\|- |
| STM2919_193_227 | 0.03 | 1.19 | 1254442\|ygbI\|putative regulatory protein\|+ |
| STM2920_113_147 | 0.02 | 1.34 | 1254443\|STM2920\|putative transcriptional regulator\|- |
| STM2921_65_99 | 0.17 | -1.15 | 1254444\|STM2921\|putative flavoprotein\|+ |
| STM2922_1197_1231 | 0.39 | 1.06 | 1254445\|STM2922\|putative 3-polyprenyl-4-hydroxybenzoate decarboxylase\|+ |
| STM2923_81_115 | 0.03 | 2.38 | 1254446\|STM2923\|putative cytoplasmic protein\|+ |
| STM2924_257_291 | 0.00 | -2.52 | 1254447\|rpoS\|RNA polymerase sigma factor RpoS\|- |
| STM2925_327_361 | 0.36 | -1.16 | 1254448\|nlpD\|predicted outer membrane lipoprotein\|- |
| STM2926_273_307 | 0.44 | -1.10 | 1254449\|pcm\|protein-L-isoaspartate O-methyltransferase\|- |

| | | | |
|---|---|---|---|
| STM2927_481_515 | 0.03 | -1.47 | 1254450\|surE\|stationary phase survival protein SurE\|- |
| STM2928_643_677 | 0.00 | -1.35 | 1254451\|truD\|tRNA pseudouridine synthase D\|- |
| STM2929_353_387 | 0.40 | -1.19 | 1254452\|ispF\|2-C-methyl-D-erythritol 2 4-cyclodiphosphate synthase\|- |
| STM2930_513_547 | 0.93 | -1.02 | 1254453\|ispD\|2-C-methyl-D-erythritol 4-phosphate cytidylyltransferase\|- |
| STM2931_49_83 | 0.01 | -1.60 | 1254454\|ftsB\|cell divison protein FtsB\|- |
| STM2932_229_263 | 0.12 | 1.77 | 1254455\|ygbE\|hypothetical protein\|- |
| STM2933_161_195 | 0.43 | -1.45 | 1254456\|cysC\|adenylylsulfate kinase\|- |
| STM2934_697_731 | 0.48 | -1.39 | 1254457\|cysN\|sulfate adenylyltransferase subunit 1\|- |
| STM2935_161_195 | 0.99 | 1.01 | 1254458\|cysD\|sulfate adenylyltransferase subunit 2\|- |
| STM2936_336_370 | 0.51 | 1.13 | 1254459\|iap\|aminopeptidase in alkaline phosphatase isozyme conversion\|+ |
| STM2937_146_180 | 0.03 | -1.22 | 1254460\|ygbF\|hypothetical protein\|- |
| STM2938_769_803 | 0.05 | -1.21 | 1254461\|STM2938\|putative cytoplasmic protein\|- |
| STM2939_481_515 | 0.02 | -1.55 | 1254462\|ygcH\|putative cytoplasmic protein\|- |
| STM2940_513_547 | 0.08 | -1.30 | 1254463\|STM2940\|putative cytoplasmic protein\|- |
| STM2941_988_1022 | 0.01 | -2.11 | 1254464\|yghJ\|putative cytoplasmic protein\|- |
| STM2943_622_656 | 0.01 | -2.18 | 1254466\|STM2943\|putative cytoplasmic protein\|- |
| STM2944_1857_1891 | 0.00 | -1.68 | 1254467\|ygcB\|putative helicase\|- |
| STM2945_713_747 | 0.04 | 1.64 | 1254468\|sopD\|secreted effector protein\|+ |
| STM2946_553_587 | 0.44 | -1.22 | 1254469\|cysH\|phosphoadenosine phosphosulfate reductase\|- |
| STM2947_1146_1180 | 0.39 | -1.34 | 1254470\|cysI\|sulfite reductase alpha subunit\|- |
| STM2948_1201_1235 | 0.39 | -1.35 | 1254471\|cysJ\|sulfite reductase subunit alpha\|- |
| STM2949_129_163 | 0.86 | 1.03 | 1254472\|ptpS\|putative 6-pyruvoyl tetrahydrobiopterin synthase\|+ |
| STM2950_193_227 | 0.31 | 1.25 | 1254473\|STM2950\|putative metal-dependent hydrolase\|- |
| STM2951_129_163 | 0.01 | 2.11 | 1254474\|ygcF\|hypothetical protein\|- |
| STM2952_684_718 | 0.07 | 1.41 | 1254475\|eno\|phosphopyruvate hydratase\|- |
| STM2953_895_929 | 0.02 | 2.07 | 1254476\|pyrG\|CTP synthase\|- |
| STM2954_609_643 | 0.01 | 1.44 | 1254477\|mazG\|nucleoside triphosphate pyrophosphohydrolase\|- |
| STM2954.1n_217_254 | 0.01 | 2.45 | 2673739\|STM2954.1n\|hypothetical protein\|- |
| STM2955.S_77_111 | 0.01 | 1.44 | 1254478\|STM2955.S\|putative transcriptional regulator\|- |
| STM2956_2176_2210 | 0.09 | 1.44 | 1254479\|relA\|GDP/GTP pyrophosphokinase\|- |
| STM2957_1225_1259 | 0.03 | 1.29 | 1254480\|rumA\|23S rRNA 5-methyluridine methyltransferase\|- |
| STM2958_1790_1824 | 0.11 | -1.16 | 1254481\|barA\|hybrid sensory histidine kinase BarA\|+ |
| STM2959_800_834 | 0.04 | 1.22 | 1254482\|STM2959\|putative glycerate kinase 2\|- |
| STM2960_1190_1224 | 0.07 | -1.50 | 1254483\|gudD\|d-glucarate dehydratase\|- |
| STM2961_374_408 | 0.46 | 1.26 | 1254484\|ygcY\|putative D-glucarate dehydratase\|- |
| STM2962_748_782 | 0.64 | -1.10 | 1254485\|gudT\|putative D-glucarate permease\|- |

| | | | |
|---|---|---|---|
| STM2963_137_171 | 0.92 | 1.01 | 1254486\|STM2963\|flavodoxin\|- |
| STM2964_513_547 | 0.02 | -1.84 | 1254487\|yqcB\|tRNA pseudouridine synthase C\|- |
| STM2965_169_203 | 0.02 | -1.47 | 1254488\|yqcC\|putative cytoplasmic protein\|- |
| STM2966_210_244 | 0.04 | -2.45 | 1254489\|csrB\|misc_RNA\|- |
| STM2967_417_451 | 0.15 | -1.17 | 1254490\|syd\|SecY interacting protein Syd\|- |
| STM2968_785_819 | 0.59 | 1.07 | 1254491\|queF\|7-cyano-7-deazaguanine reductase\|+ |
| STM2969_638_672 | 0.05 | 1.17 | 1254492\|ygdH\|putative nucleotide binding\|+ |
| STM2970_1187_1221 | 0.03 | -2.09 | 1254493\|sdaC\|putative serine transport protein\|+ |
| STM2971_497_531 | 0.14 | -1.43 | 1254494\|sdaB\|L-serine dehydratase/L-threonine deaminase 2\|+ |
| STM2972_417_451 | 0.01 | 1.50 | 1254495\|xni\|exonuclease IX\|+ |
| STM2973_182_216 | 0.24 | -1.27 | 1254496\|fucO\|L-1 2-propanediol oxidoreductase\|- |
| STM2974_545_579 | 0.08 | -1.35 | 1254497\|fucA\|L-fuculose phosphate aldolase\|- |
| STM2975_831_865 | 0.00 | -2.39 | 1254498\|fucP\|pseudo\|+ |
| STM2977_708_742 | 0.09 | -1.49 | 1254500\|fucK\|L-fuculokinase\|+ |
| STM2978_137_171 | 0.00 | -1.45 | 1254501\|fucU\|putative L-fucose-binding protein\|+ |
| STM2979_225_259 | 0.00 | -7.83 | 1254502\|fucR\|DNA-binding transcriptional activator\|+ |
| STM2980_1014_1048 | 0.33 | 1.13 | 1254503\|ygdE\|putative SAM-dependent methyltransferase\|- |
| STM2981_345_379 | 0.37 | 1.07 | 1254504\|ygdD\|hypothetical protein\|- |
| STM2982_833_867 | 0.08 | 1.80 | 1254505\|gcvA\|DNA-binding transcriptional activator GcvA\|- |
| STM2983_49_83 | 0.92 | -1.01 | 1254506\|ygdI\|putative lipoprotein\|- |
| STM2984_741_775 | 0.32 | 1.07 | 1254507\|csdA\|cysteine sulfinate desulfinase\|+ |
| STM2985_199_233 | 0.05 | 1.78 | 1254508\|ygdK\|putative FeS center assembly protein\|+ |
| STM2986.Sc_705_739 | 0.05 | 1.23 | 1254509\|STM2986.Sc\|putative integral membrane protein\|+ |
| STM2987_193_227 | 0.94 | -1.01 | 1254510\|ygdL\|putative enzyme\|- |
| STM2988_867_901 | 0.11 | -1.37 | 1254511\|mltA\|murein transglycosylase A\|- |
| STM2991_1087_1121 | 0.11 | 1.57 | 1254514\|amiC\|N-acetylmuramoyl-L-alanine amidase\|- |
| STM2992_333_367 | 0.09 | 1.21 | 1254515\|argA\|N-acetylglutamate synthase\|+ |
| STM2993_1581_1615 | 0.06 | -1.66 | 1254516\|RecD\|exonuclease V subunit alpha\|- |
| STM2994_2739_2773 | 0.17 | -1.19 | 1254517\|RecB\|exonuclease V subunit beta\|- |
| STM2995_2370_2404 | 0.43 | 1.07 | 1254518\|ptr\|protease III\|- |
| STM2996_3301_3335 | 0.16 | 1.28 | 1254519\|recC\|exonuclease V subunit gamma\|- |
| STM2997_65_99 | 0.83 | -1.02 | 1254520\|ppdC\|hypothetical protein\|- |
| STM2998_286_320 | 0.41 | -1.06 | 1254521\|ygdB\|hypothetical protein\|- |
| STM2999_321_355 | 0.04 | 2.31 | 1254522\|ppdB\|hypothetical protein\|- |
| STM3000_17_51 | 0.07 | 1.37 | 1254523\|ppdA\|hypothetical protein\|- |
| STM3001_321_355 | 0.05 | -1.28 | 1254524\|thyA\|thymidylate synthase\|- |

| | | | |
|---|---|---|---|
| STM3002_593_627 | 0.21 | -1.16 | 1254525\|lgt\|prolipoprotein diacylglyceryl transferase\|- |
| STM3003_1344_1378 | 0.25 | -1.14 | 1254526\|ptsP\|fused phosphoenolpyruvate-protein phosphotransferase PtsP/GAF domain\|- |
| STM3004_449_483 | 0.06 | 1.18 | 1254527\|ygdP\|dinucleoside polyphosphate hydrolase\|- |
| STM3004.1n_21_55 | 0.07 | 1.66 | 2673726\|STM3004.1n\|hypothetical protein\|- |
| STM3005_233_267 | 0.12 | 1.44 | 1254528\|mutH\|DNA mismatch repair protein\|+ |
| STM3006_417_451 | 0.01 | -1.23 | 1254529\|ygdQ\|putative transport protein\|+ |
| STM3007_57_91 | 0.00 | -1.89 | 1254530\|ygdR\|putative peptide transport protein\|+ |
| STM3008_266_300 | 0.02 | -2.30 | 1254531\|tas\|putative aldo/keto reductase\|+ |
| STM3009_356_390 | 0.03 | -1.36 | 1254532\|ygeD\|hypothetical protein\|- |
| STM3010_2009_2043 | 0.03 | -1.33 | 1254533\|aas\|bifunctional acyl-[acyl carrier protein] synthetase/2-acylglycerophosphoethanolamine acyltransferase\|- |
| STM3011_318_352 | 0.37 | -1.08 | 1254534\|galR\|DNA-binding transcriptional repressor\|+ |
| STM3012_392_426 | 0.03 | 1.62 | 1254535\|STM3012\|putative transcriptional regulator\|+ |
| STM3013_776_810 | 0.76 | 1.04 | 1254536\|lysA\|diaminopimelate decarboxylase\|- |
| STM3014_193_227 | 0.02 | 1.43 | 1254537\|lysR\|DNA-binding transcriptional regulator LysR\|+ |
| STM3015_353_387 | 0.05 | -1.42 | 1254538\|ygeA\|putative racemase\|- |
| STM3016_1188_1222 | 0.80 | 1.04 | 1254539\|araE\|L-arabinose/proton symport protein\|- |
| STM3017_193_227 | 0.63 | 1.03 | 1254540\|kduD\|2-deoxy-D-gluconate 3-dehydrogenase\|- |
| STM3019_692_726 | 0.01 | -6.30 | 1254542\|yqeF\|acetyl-CoA acetyltransferase\|- |
| STM3020_337_371 | 0.29 | 1.13 | 1254543\|STM3020\|putative transcriptional regulator\|- |
| STM3021_321_355 | 0.17 | 1.09 | 1254544\|STM3021\|putative inner membrane protein\|+ |
| STM3022_615_649 | 0.03 | 1.76 | 1254545\|STM3022\|putative transport protein\|+ |
| STM3023_149_183 | 0.01 | 1.47 | 1254546\|yohL\|putative cytoplasmic protein\|- |
| STM3024_449_483 | 0.11 | -1.07 | 1254547\|yohM\|nickel/cobalt efflux protein RcnA\|+ |
| STM3025_145_179 | 0.01 | 1.31 | 1254548\|STM3025\|putative cytoplasmic protein\|- |
| STM3025.1N_181_215 | 0.14 | 1.45 | 2673750\|STM3025.1N\|hypothetical protein\|- |
| STM3026_174_209 | 0.05 | -1.33 | 1254549\|STM3026\|putative outer membrane protein\|- |
| STM3028_1779_1813 | 0.03 | -1.67 | 1254551\|stdB\|putative outer membrane usher protein\|- |
| STM3031_473_507 | 0.00 | 3.41 | 1254554\|STM3031\|Ail/OmpX-like protein\|- |
| STM3033_225_259 | 0.01 | 1.45 | 1254556\|STM3033\|putative nucleic acid-binding protein\|- |
| STM3034_37_72 | 0.01 | 1.58 | 1254557\|STM3034\|putative cytoplasmic protein\|- |
| STM3036_257_291 | 0.01 | -1.53 | 1254559\|STM3036\|putative inner membrane protein\|+ |
| STM3037_2_36 | 0.55 | -1.08 | 1254560\|glyU\|tRNA\|- |
| STM3038_101_135 | 0.12 | 1.66 | 1254561\|STM3038\|putative metalloendopeptidase\|- |
| STM3039_305_339 | 0.01 | 1.52 | 1254562\|idi\|isopentenyl-diphosphate delta-isomerase\|+ |

| | | | |
|---|---|---|---|
| STM3040_775_809 | 0.04 | -1.70 | 1254563llysSllysyl-tRNA synthetasel- |
| STM3041c_638_672 | 0.53 | -1.13 | 1254564lprfBlpeptide chain release factor 2l- |
| STM3042_1407_1441 | 0.92 | 1.01 | 1254565lrecJlssDNA exonuclease RecJl- |
| STM3043_553_587 | 0.04 | 1.37 | 1254566ldsbClthiol:disulfide interchange protein DsbCl- |
| STM3044_321_355 | 0.15 | -1.30 | 1254567lxerDlsite-specific tyrosine recombinase XerDl- |
| STM3045_361_395 | 0.92 | -1.03 | 1254568lfldBlflavodoxin FldBl+ |
| STM3046_193_227 | 0.02 | -1.40 | 1254569lygfXlputative inner membrane proteinl- |
| STM3047_169_203 | 0.95 | -1.01 | 1254570lygfYlhypothetical proteinl- |
| STM3048_513_547 | 0.05 | 1.57 | 1254571lygfZlputative global regulatorl+ |
| STM3049_257_291 | 0.06 | -1.28 | 1254572lyqfAlputative hemolysinl- |
| STM3050_68_102 | 0.07 | 1.16 | 1254573lyqfBlhypothetical proteinl- |
| STM3051_827_861 | 0.09 | 1.21 | 1254574lbglAl6-phospho-beta-glucosidase Al+ |
| STM3052_561_595 | 0.02 | 2.69 | 1254575lSTM3052lputative outer membrane proteinl- |
| STM3053_2483_2517 | 0.01 | -1.94 | 1254576lgcvPlglycine dehydrogenasel- |
| STM3054_49_83 | 0.01 | -3.26 | 1254577lgcvHlglycine cleavage system protein Hl- |
| STM3055_864_898 | 0.01 | -3.23 | 1254578lgcvTlglycine cleavage system aminomethyltransferase Tl- |
| STM3056_268_302 | 0.07 | 1.41 | 1254579lvisClhypothetical proteinl- |
| STM3057_884_918 | 0.34 | 1.18 | 1254580lubiHl2-octaprenyl-6-methoxyphenyl hydroxylasel- |
| STM3058_990_1024 | 0.71 | 1.05 | 1254581lpepPlproline aminopeptidase P IIl- |
| STM3059.S_385_419 | 0.02 | 1.38 | 1254582lygfBlhypothetical proteinl- |
| STM3060_265_299 | 0.02 | 1.55 | 1254583lygfElZ-ring-associated proteinl+ |
| STM3061_445_479 | 0.02 | 1.62 | 1254584lygfAlpredicted ligasel+ |
| STM3062_298_332 | 0.81 | -1.03 | 1254585lserAlD-3-phosphoglycerate dehydrogenasel- |
| STM3063_241_275 | 0.00 | -3.26 | 1254586lrpiAlribose-5-phosphate isomerase Al- |
| STM3064_641_675 | 0.03 | 1.23 | 1254587liciAlchromosome replication initiation inhibitor proteinl+ |
| STM3065_273_307 | 0.05 | -1.54 | 1254588lyggElhypothetical proteinl- |
| STM3066_377_411 | 1.00 | -1.00 | 1254589lyggAlarginine exporter proteinl- |
| STM3067_769_803 | 0.02 | -1.48 | 1254590lyggBlmechanosensitive channel MscSl- |
| STM3068_593_627 | 0.28 | -1.22 | 1254591lfbalfructose-bisphosphate aldolasel- |
| STM3069_677_711 | 0.62 | -1.05 | 1254592lpgklphosphoglycerate kinasel- |
| STM3070_432_466 | 0.01 | -1.77 | 1254593lepdlD-erythrose 4-phosphate dehydrogenasel- |
| STM3071_193_227 | 0.06 | -1.44 | 1254594lSTM3071lputative DNA-binding proteinl+ |
| STM3072_453_487 | 0.03 | -1.64 | 1254595lSTM3072lputative inner membrane proteinl+ |
| STM3073_289_323 | 0.01 | -1.97 | 1254596lSTM3073lputative ABC-type cobalt transport system permease componentl+ |

| | | | |
|---|---|---|---|
| STM3074_385_419 | 0.09 | 1.34 | 1254597\|STM3074\|putative ABC-type cobalt transport system ATP-binding component\|+ |
| STM3075_9_43 | 0.44 | -1.10 | 1254598\|STM3075\|putative ABC-type cobalt transport system ATP-binding component\|+ |
| STM3076_1201_1235 | 0.60 | -1.13 | 1254599\|tktA\|transketolase\|- |
| STM3077_257_291 | 0.01 | 1.38 | 1254600\|yggG\|putative Zn-dependent protease\|+ |
| STM3078_97_131 | 0.74 | -1.06 | 1254601\|speB\|agmatinase\|- |
| STM3079.S_129_163 | 0.54 | -1.11 | 1254602\|STM3079.S\|putative hydrolase/acyltransferase\|- |
| STM3081_809_843 | 0.05 | 2.09 | 1254604\|STM3081\|putative malate/L-lactate dehydrogenase\|- |
| STM3082_770_804 | 0.04 | 2.25 | 1254605\|STM3082\|putative zinc-binding dehydrogenase\|- |
| STM3083_922_956 | 0.37 | -1.13 | 1254606\|STM3083\|putative mannitol dehydrogenase\|- |
| STM3084.S_297_331 | 0.25 | 1.17 | 1254607\|STM3084.S\|putative regulatory protein\|- |
| STM3086_1106_1140 | 0.04 | 1.11 | 1254609\|speA\|arginine decarboxylase\|- |
| STM3087_49_83 | 0.96 | 1.01 | 1254610\|yqgB\|putative inner membrane protein\|- |
| STM3088_10_44 | 0.04 | 1.80 | 2673737\|yqgC\|-\|+ |
| STM3089_77_111 | 0.02 | 1.21 | 1254612\|yqgD\|putative inner membrane protein\|- |
| STM3090_284_318 | 0.01 | 9.64 | 1254613\|metK\|methionine adenosyltransferase\|+ |
| STM3091_460_494 | 0.77 | -1.08 | 1254614\|galP\|galactose/proton symporter\|+ |
| STM3093_177_211 | 0.03 | 2.10 | 1254616\|endA\|DNA-specific endonuclease I\|+ |
| STM3094_65_99 | 0.02 | -1.51 | 1254617\|yggJ\|hypothetical protein\|+ |
| STM3095_217_251 | 0.18 | -1.20 | 1254618\|gshB\|glutathione synthetase\|+ |
| STM3096_43_77 | 0.18 | 1.42 | 1254619\|yqgE\|hypothetical protein\|+ |
| STM3097_177_211 | 0.01 | -2.03 | 1254620\|yqgF\|Holliday junction resolvase-like protein\|+ |
| STM3100_9_43 | 0.06 | 1.45 | 1254623\|yggS\|hypothetical protein\|+ |
| STM3101_257_291 | 0.04 | -1.16 | 1254624\|yggT\|putative integral membrane protein\|+ |
| STM3102_65_99 | 0.03 | -1.54 | 1254625\|yggU\|hypothetical protein\|+ |
| STM3103_305_339 | 0.13 | 1.41 | 1254626\|yggV\|putative deoxyribonucleotide triphosphate pyrophosphatase\|+ |
| STM3104_490_524 | 0.03 | -1.68 | 1254627\|yggW\|coproporphyrinogen III oxidase\|+ |
| STM3105_299_333 | 0.00 | 1.78 | 1254628\|yggM\|putative periplasmic protein\|- |
| STM3106_592_626 | 0.02 | -1.82 | 1254629\|ansB\|L-asparaginase II\|- |
| STM3107_577_611 | 0.36 | 1.13 | 1254630\|yggN\|hypothetical protein\|- |
| STM3108_289_323 | 0.02 | 1.81 | 1254631\|yggL\|hypothetical protein\|- |
| STM3109_673_707 | 0.03 | 1.38 | 1254632\|trmB\|tRNA (guanine-N(7))-methyltransferase\|- |
| STM3110_118_152 | 0.15 | -1.36 | 1254633\|mutY\|adenine DNA glycosylase\|+ |
| STM3111_85_119 | 0.68 | -1.05 | 1254634\|yggX\|hypothetical protein\|+ |
| STM3112_407_441 | 0.02 | -1.50 | 1254635\|mltC\|murein transglycosylase C\|+ |

| | | | |
|---|---|---|---|
| STM3113_514_548 | 0.35 | 1.20 | 1254636lnupGlnucleoside transportl+ |
| STM3114_1457_1491 | 0.47 | -1.09 | 1254637lspeClornithine decarboxylase isozymel- |
| STM3115_481_515 | 0.14 | 1.24 | 1254638lyqgAlputative inner membrane proteinl+ |
| STM3117_361_395 | 0.04 | 1.37 | 1254640lSTM3117lputative lactoylglutathione lyasel- |
| STM3118_1063_1097 | 0.05 | 1.62 | 1254641lSTM3118lputative acetyl-CoA hydrolasel- |
| STM3119_449_483 | 0.08 | 1.20 | 1254642lSTM3119lputative monoamine oxidasel- |
| STM3120_753_787 | 0.39 | -1.07 | 1254643lSTM3120lputative transcriptional regulatorl- |
| STM3121_673_707 | 0.01 | 1.80 | 1254644lSTM3121lputative transcriptional regulatorl+ |
| STM3122_1637_1671 | 0.18 | 1.33 | 1254645lSTM3122lputative arylsulfatasel- |
| STM3123_1146_1180 | 0.03 | 2.04 | 1254646lSTM3123lputative arylsulfatase regulatorl- |
| STM3124_17_51 | 0.01 | -8.38 | 1254647lSTM3124lputative response regulatorl+ |
| STM3125_277_313 | 0.05 | 1.30 | 1254648lSTM3125lputative cytoplasmic proteinl- |
| STM3126_501_535 | 0.09 | 2.29 | 1254649lSTM3126lputative amino acid transporterl- |
| STM3127_173_207 | 0.31 | -1.19 | 1254650lSTM3127lputative cytoplasmic proteinl- |
| STM3128_495_529 | 0.13 | 1.06 | 1254651lSTM3128lputative oxidoreductasel- |
| STM3129_646_680 | 0.12 | 1.18 | 1254652lSTM3129lputative NAD-dependent aldehyde dehydrogenasel+ |
| STM3130_345_379 | 0.04 | 3.01 | 1254653lSTM3130lputative cytoplasmic proteinl- |
| STM3131_273_307 | 0.13 | -1.11 | 1254654lSTM3131lputative cytoplasmic proteinl- |
| STM3132_65_99 | 0.68 | 1.11 | 1254655lSTM3132lputative xylanase/chitin deacetylasel- |
| STM3133_354_388 | 0.02 | -1.45 | 1254656lSTM3133lputative amidohydrolasel- |
| STM3134_354_388 | 0.60 | 1.12 | 1254657lSTM3134lputative permeasel- |
| STM3135_954_988 | 0.01 | -3.58 | 1254658lSTM3135lmannonate dehydratasel+ |
| STM3137_1342_1376 | 0.00 | -4.09 | 1254660lSTM3137lglucuronate isomerasel+ |
| STM3138_636_670 | 0.04 | 1.67 | 1254661lSTM3138lputative methyl-accepting chemotaxis proteinl- |
| STM3139_1002_1036 | 0.01 | 1.60 | 1254662lgsplbifunctional glutathionylspermidine amidase/glutathionylspermidine synthetasel- |
| STM3140_769_803 | 0.21 | 1.10 | 1254663lyghUlputative glutathione S-transferase YghUl+ |
| STM3141_545_579 | 0.00 | 2.01 | 1254664lSTM3141lhypothetical proteinl- |
| STM3142_846_880 | 0.02 | 3.20 | 1254665lSTM3142lputative periplasmic ferrichrome-binding proteinl- |
| STM3143_113_147 | 0.01 | -1.50 | 1254666lhybGlhydrogenase 2 accessory protein HypGl- |
| STM3144_12_46 | 0.02 | -1.28 | 1254667lhypAlhydrogenase nickel incorporation protein HybFl- |
| STM3145_265_299 | 0.23 | -1.16 | 1254668lhybElhydrogenase 2-specific chaperonel- |
| STM3146_449_483 | 0.13 | -1.30 | 1254669lhybDlpredicted maturation element for hydrogenase 2l- |
| STM3147_1249_1283 | 0.73 | -1.03 | 1254670lhybClhydrogenase 2 large subunitl- |
| STM3148_228_262 | 0.16 | -1.28 | 1254671lhybBlpredicted hydrogenase 2 cytochrome b type componentl- |
| STM3149_673_707 | 0.04 | 1.19 | 1254672lhybAlhydrogenase 2 protein HybAl- |

| | | | |
|---|---|---|---|
| STM3150_120_154 | 0.42 | 1.18 | 1254673\|hypO\|hydrogenase 2 small subunit\|- |
| STM3151_113_147 | 0.20 | -1.10 | 1254674\|yghW\|putative cytoplasmic protein\|- |
| STM3152_1061_1095 | 0.05 | -1.34 | 1254675\|STM3152\|putative methyl-accepting chemotaxis protein\|- |
| STM3153_201_235 | 0.18 | 1.17 | 1254676\|yqhA\|hypothetical protein\|- |
| STM3154_41_75 | 0.07 | 1.82 | 1254677\|STM3154\|putative ATP-dependent RNA helicase-like protein\|- |
| STM3155_177_213 | 0.03 | 1.52 | 1254678\|STM3155\|putative cytoplasmic protein\|+ |
| STM3156_177_211 | 0.10 | 1.51 | 1254679\|STM3156\|putative cytoplasmic protein\|+ |
| STM3157_545_579 | 0.72 | 1.06 | 1254680\|yghA\|oxidoreductase\|+ |
| STM3158_129_163 | 0.34 | 1.08 | 1254681\|exbD\|biopolymer transport protein ExbD\|- |
| STM3159_81_115 | 0.06 | -1.20 | 1254682\|exbB\|biopolymer transport protein ExbB\|- |
| STM3160_41_80 | 0.01 | -2.20 | 1254683\|STM3160\|putative inner membrane protein\|- |
| STM3161_1101_1135 | 0.17 | -1.23 | 1254684\|metC\|cystathionine beta-lyase\|+ |
| STM3162_513_547 | 0.02 | 1.90 | 1254685\|yghB\|hypothetical protein\|+ |
| STM3163_577_611 | 0.73 | -1.02 | 1254686\|yqhC\|putative transcriptional regulator\|- |
| STM3164_229_263 | 0.04 | 1.99 | 1254687\|yqhD\|putative alcohol dehydrogenase\|+ |
| STM3165_117_151 | 0.43 | 1.10 | 1254688\|dkgA\|2 5-diketo-D-gluconate reductase A\|+ |
| STM3167_377_411 | 0.14 | -1.21 | 1254690\|STM3167\|putative diadenosine tetraphosphatehydrolase\|+ |
| STM3168_1301_1335 | 0.03 | -1.61 | 1254691\|ygiR\|hypothetical protein\|- |
| STM3170_53_87 | 0.03 | 1.46 | 1254693\|STM3170\|putative inner membrane protein\|+ |
| STM3171_1237_1271 | 0.50 | 1.11 | 1254694\|ygiK\|putative transporter\|+ |
| STM3172_494_528 | 0.04 | -1.38 | 1254695\|sufI\|repressor protein for FtsI\|- |
| STM3173_337_371 | 0.62 | -1.02 | 1254696\|plsC\|1-acyl-sn-glycerol-3-phosphate acyltransferase\|- |
| STM3174_1644_1678 | 0.02 | -1.74 | 1254697\|parC\|DNA topoisomerase IV subunit A\|- |
| STM3175_801_835 | 0.15 | -1.05 | 1254698\|STM3175\|putative regulatory protein\|- |
| STM3176_203_237 | 0.23 | -1.26 | 1254699\|ygiW\|putative outer membrane protein\|- |
| STM3177_25_59 | 0.15 | 1.18 | 1254700\|ygiX\|DNA-binding response regulator in two-component regulatory system with QseC\|+ |
| STM3179_49_83 | 0.03 | 1.36 | 1254702\|mdaB\|NADPH-specific quinone oxidoreductase\|+ |
| STM3180_161_195 | 0.72 | -1.08 | 1254703\|ygiN\|putative cytoplasmic protein\|+ |
| STM3181_1406_1440 | 0.02 | -1.43 | 1254704\|parE\|DNA topoisomerase IV subunit B\|- |
| STM3182_425_459 | 0.01 | -1.96 | 1254705\|yqiA\|esterase YqiA\|- |
| STM3183_135_169 | 0.00 | -3.11 | 1254706\|icc\|cyclic 3' 5'-adenosine monophosphate phosphodiesterase\|- |
| STM3184_385_419 | 0.02 | -1.55 | 1254707\|yqiB\|putative cytoplasmic protein\|- |
| STM3185_489_523 | 0.03 | 1.48 | 1254708\|nudF\|ADP-ribose pyrophosphatase NudF\|- |
| STM3186_535_569 | 0.14 | -1.13 | 1254709\|tolC\|outer membrane channel protein\|+ |
| STM3187_123_157 | 0.00 | 2.84 | 1254710\|ygiB\|hypothetical protein\|+ |

| | | | |
|---|---|---|---|
| STM3188_1029_1063 | 0.29 | 1.09 | 1254711lygiClputative glutathionylspermidine synthasel+ |
| STM3189_529_563 | 0.17 | -1.07 | 1254712lygiDlputative cytoplasmic proteinl- |
| STM3190_585_619 | 0.02 | -1.21 | 1254713lygiElzinc transporter ZupTl+ |
| STM3191_833_867 | 0.78 | 1.04 | 1254714lSTM3191lputative arylsulfate sulfotransferasel+ |
| STM3192_641_675 | 0.43 | 1.08 | 1254715lSTM3192lputative arylsulfate sulfotransferasel+ |
| STM3193_625_659 | 0.06 | 2.75 | 1254716lSTM3193lputative disulfide bond formation proteinl+ |
| STM3194_141_175 | 0.24 | 1.10 | 1254717lSTM3194lputative disulfide oxidoreductasel+ |
| STM3195_505_539 | 0.01 | -3.93 | 1254718lribBl3 4-dihydroxy-2-butanone 4-phosphate synthasel- |
| STM3196.S_33_67 | 0.01 | 1.78 | 1254719lyqiClputative cytoplasmic proteinl+ |
| STM3197_113_147 | 0.02 | -1.73 | 1254720lglgSlglycogen synthesis protein GlgSl- |
| STM3198_321_355 | 0.23 | 1.49 | 1254721lSTM3198lputative inner membrane proteinl+ |
| STM3199_1561_1595 | 0.94 | -1.00 | 1254722lyqiKlhypothetical proteinl+ |
| STM3200_659_693 | 0.02 | -1.84 | 1254723lrfaElbifunctional heptose 7-phosphate kinase/heptose 1-phosphate adenyltransferasel- |
| STM3201_2549_2583 | 0.00 | -2.82 | 1254724lglnElbifunctional glutamine-synthetase adenylyltransferase/deadenyltransferasel- |
| STM3202_1199_1233 | 0.02 | 1.26 | 1254725lygiFlputative cytoplasmic proteinl- |
| STM3203_113_147 | 0.02 | -1.35 | 1254726lygiMlputative signal transduction proteinl+ |
| STM3204_1139_1173 | 0.33 | 1.18 | 1254727lccalmultifunctional tRNA nucleotidyl transferase/2'3'-cyclic phosphodiesterase/2'nucleotidase/phosphatasel+ |
| STM3205_161_195 | 0.03 | -1.29 | 1254728luppPlundecaprenyl pyrophosphate phosphatasel- |
| STM3206_257_291 | 0.09 | 1.84 | 1254729lfolBlbifunctional dihydroneopterin aldolase/dihydroneopterin triphosphate 2'-epimerasel- |
| STM3207_389_423 | 0.67 | -1.07 | 1254730lygiHlhypothetical proteinl+ |
| STM3208_307_341 | 0.05 | 1.23 | 1254731lgcplO-sialoglycoprotein endopeptidasel- |
| STM3209_11_45 | 0.03 | -2.18 | 1254732lrpsUl30S ribosomal protein S21l+ |
| STM3210_875_909 | 0.00 | -1.58 | 1254733ldnaGlDNA primasel+ |
| STM3211.S_1185_1219 | 0.03 | -1.44 | 1254734lrpoDlRNA polymerase sigma factor RpoDl+ |
| STM3212_241_275 | 0.02 | 1.87 | 1254735lmuglG/U mismatch-specific DNA glycosylasel- |
| STM3213_29_63 | 0.45 | -1.18 | 1254736lileXltRNAl+ |
| STM3214_425_459 | 0.45 | 1.08 | 1254737lyqjHlputative transporterl- |
| STM3215_297_331 | 0.06 | 1.41 | 1254738lyqjIlputative transcriptional regulatorl+ |
| STM3216_567_601 | 0.05 | -1.24 | 1254739lSTM3216lputative methyl-accepting chemotaxis proteinl- |
| STM3217_1042_1076 | 0.01 | -1.45 | 1254740laerlaerotaxis sensor receptorl- |
| STM3218_1144_1178 | 0.01 | -3.65 | 1254741loatlputrescine--2-oxoglutarate aminotransferasel+ |
| STM3220_786_820 | 0.76 | -1.05 | 1254743lygjOlputative methyltransferasel- |

| | | | |
|---|---|---|---|
| STM3221_337_371 | 0.11 | 1.39 | 1254744lygjPlputative metal-dependent hydrolasel+ |
| STM3223_641_675 | 0.00 | -2.94 | 1254746lygjRlputative dehydrogenasel+ |
| STM3224_225_259 | 0.07 | 1.40 | 1254747lygjTlputative tellurite resistance proteinl+ |
| STM3225_1142_1176 | 0.00 | -4.52 | 1254748lygjUlputative dicarboxylate permeasel+ |
| STM3226_353_387 | 0.18 | -1.28 | 1254749lyqjAlhypothetical proteinl+ |
| STM3227_273_307 | 0.05 | 1.20 | 1254750lyqjBlhypothetical proteinl+ |
| STM3228_297_331 | 0.01 | 1.88 | 1254751lyqjClputative periplasmic proteinl+ |
| STM3229_9_43 | 0.49 | -1.09 | 1254752lyqjDlputative inner membrane proteinl+ |
| STM3230_257_291 | 0.02 | -1.42 | 1254753lyqjElputative inner membrane proteinl+ |
| STM3231_113_147 | 0.04 | -1.37 | 1254754lyqjKlputative inner membrane proteinl+ |
| STM3232_193_227 | 0.89 | 1.03 | 1254755lyqjFlhypothetical proteinl+ |
| STM3233_769_803 | 0.51 | 1.14 | 1254756lyqjGlputative glutathione S-transferasel+ |
| STM3234_277_311 | 0.13 | -1.15 | 1254757lyhaHlputative inner membrane proteinl+ |
| STM3235_137_171 | 0.04 | -1.16 | 1254758lyhaJlputative transcriptional regulatorl- |
| STM3236_449_483 | 0.02 | 1.60 | 1254759lyhaKlputative cytoplasmic proteinl+ |
| STM3237_20_54 | 0.00 | -3.02 | 1254760lyhaLlputative cytoplasmic proteinl+ |
| STM3239_397_431 | 0.07 | -1.11 | 1254762lyhaOlputative transport proteinl- |
| STM3240_918_952 | 0.78 | 1.13 | 1254763ltdcGlL-serine deaminasel- |
| STM3241_1664_1698 | 0.06 | 1.65 | 1254764ltdcElpyruvate formate-lyase 4/2-ketobutyrate formate-lyasel- |
| STM3242_258_292 | 0.01 | -1.45 | 1254765ltdcDlpropionate/acetate kinasel- |
| STM3243_333_367 | 0.02 | -1.61 | 1254766ltdcClL-threonine/L-serine permeasel- |
| STM3244_641_675 | 0.40 | -1.14 | 1254767ltdcBlthreonine dehydratasel- |
| STM3245_65_99 | 0.03 | -1.83 | 1254768ltdcAlDNA-binding transcriptional activatorl- |
| STM3246_9_43 | 0.99 | -1.00 | 1254769lrnpBlmisc_RNAl+ |
| STM3247_571_605 | 0.03 | 1.87 | 1254770lgarKlglycerate kinase II- |
| STM3248_185_219 | 0.00 | -1.66 | 1254771lgarRltartronate semialdehyde reductasel- |
| STM3249_385_419 | 0.05 | 1.53 | 1254772lgarLlalpha-dehydro-beta-deoxy-D-glucarate aldolasel- |
| STM3250_1277_1311 | 0.02 | -1.79 | 1254773lgarDlgalactarate dehydrogenasel+ |
| STM3251_649_683 | 0.80 | -1.03 | 1254774lSTM3251lputative sugar kinasel- |
| STM3252_401_435 | 0.03 | 1.43 | 1254775lagaRlAga operon transcriptional repressorl- |
| STM3253_289_323 | 0.14 | -1.26 | 1254776lgatYltagatose-bisphosphate aldolasel+ |
| STM3254_833_867 | 0.03 | 2.15 | 1254777lSTM3254lputative fructose-1-phosphate kinasel+ |
| STM3255_701_735 | 0.56 | -1.07 | 1254778lSTM3255lputative phosphotransferase system fructose-specific component IIBl+ |
| STM3256_97_131 | 0.03 | -1.37 | 1254779lSTM3256lputative phosphotransferase system mannitol/fructose-specific IIA domainl+ |

| | | | |
|---|---|---|---|
| STM3258_81_115 | 0.13 | -1.30 | 1254781lSTM3258lgalactitol-specific PTS system component IIAl+ |
| STM3260_599_633 | 0.15 | -1.15 | 1254783lSTM3260lPTS family galactitol-specific enzyme IICl+ |
| STM3262_561_595 | 0.12 | -1.32 | 1254785lSTM3262lgalactitol utilization operon transcriptional repressorl+ |
| STM3263_689_723 | 0.03 | 1.82 | 1254786lyraLlputative methyltransferasel- |
| STM3264_1236_1270 | 0.00 | 1.41 | 1254787lyraMlputative transglycosylasel+ |
| STM3265_321_355 | 0.13 | 1.26 | 1254788lyraNlhypothetical proteinl+ |
| STM3266_401_435 | 0.00 | 1.74 | 1254789lyraOlDnaA initiator-associating protein DiaAl+ |
| STM3267_321_355 | 0.09 | 1.44 | 1254790lyraPlhypothetical proteinl+ |
| STM3268_449_483 | 0.03 | 1.17 | 1254791lyraRlputative nucleoside-diphosphate-sugar epimerasel- |
| STM3269_193_227 | 0.02 | -1.78 | 1254792lyhbOlputative intracellular proteinasel+ |
| STM3271_47_81 | 0.88 | 1.02 | 1254794lyhbQlhypothetical proteinl+ |
| STM3272_49_83 | 0.13 | -1.28 | 1254795lyhbSlputative transport proteinl- |
| STM3273_45_79 | 0.49 | 1.08 | 1254796lyhbTlputative lipid carrier proteinl- |
| STM3274_337_371 | 0.08 | -1.29 | 1254797lyhbUlputative proteasel+ |
| STM3276_289_323 | 0.09 | -1.28 | 1254799lyhbWlhypothetical proteinl+ |
| STM3277_609_643 | 0.03 | 3.11 | 1254800lSTM3277lputative inner membrane proteinl- |
| STM3278_405_439 | 0.73 | 1.03 | 1254801lSTM3278lputative cytoplasmic proteinl+ |
| STM3279_694_728 | 0.10 | -1.34 | 1254802lmtrltryptophan permeasel- |
| STM3280.S_1563_1597 | 0.95 | -1.01 | 1254803ldeaDlATP-dependent RNA helicase DeaDl- |
| STM3281_449_483 | 0.01 | 1.59 | 1254804lnlpIllipoprotein NlpIl- |
| STM3282_1841_1875 | 0.36 | -1.16 | 1254805lpnplpolynucleotide phosphorylase/polyadenylasel- |
| STM3283_201_235 | 0.34 | -1.28 | 1254806lrpsOl30S ribosomal protein S15l- |
| STM3284_609_643 | 0.08 | -1.57 | 1254807ltruBltRNA pseudouridine synthase Bl- |
| STM3285_321_355 | 0.34 | 1.11 | 1254808lrbfAlribosome-binding factor Al- |
| STM3286_2256_2290 | 0.04 | -1.24 | 1254809linfBltranslation initiation factor IF-2l- |
| STM3287_1400_1434 | 0.26 | 1.08 | 1254810lnusAltranscription elongation factor NusAl- |
| STM3288_241_275 | 0.46 | 1.21 | 1254811lyhbClhypothetical proteinl- |
| STM3291_434_468 | 0.78 | 1.06 | 1254814lSTM3291lputative cytoplasmic proteinl- |
| STM3292_9_43 | 0.35 | -1.19 | 1254815lleuUltRNAl- |
| STM3293_137_171 | 0.03 | -1.26 | 1254816lsecGlpreprotein translocase subunit SecGl- |
| STM3294_339_373 | 0.60 | 1.18 | 1254817lglmMlphosphoglucosamine mutasel- |
| STM3295_673_707 | 0.15 | -1.29 | 1254818lfolPldihydropteroate synthasel- |
| STM3296_1416_1450 | 0.04 | -1.41 | 1254819lhflBlATP-dependent metalloproteasel- |
| STM3297_289_323 | 0.64 | 1.02 | 1254820lrrmJl23S rRNA methyltransferase Jl- |
| STM3298.S_33_67 | 0.06 | -1.20 | 1254821lyhbYlRNA-binding protein YhbYl+ |
| STM3299_203_237 | 0.11 | -1.31 | 1254822lgreAltranscription elongation factor GreAl- |

| | | | |
|---|---|---|---|
| STM3300_563_597 | 0.35 | 1.09 | 1254823\|dacB\|D-alanyl-D-alanine carboxypeptidase/endopeptidase\|+ |
| STM3301_510_544 | 0.01 | 1.60 | 1254824\|obgE\|GTPase ObgE\|- |
| STM3302_217_251 | 0.24 | -1.09 | 1254825\|yhbE\|pseudo\|- |
| STM3303_37_71 | 0.01 | -3.11 | 1254826\|rpmA\|50S ribosomal protein L27\|- |
| STM3304_225_259 | 0.02 | -2.43 | 1254827\|rplU\|50S ribosomal protein L21\|- |
| STM3305_497_531 | 0.13 | 1.09 | 1254828\|ispB\|octaprenyl diphosphate synthase\|+ |
| STM3306_65_99 | 0.01 | 2.07 | 1254829\|nlp\|DNA-binding transcriptional activator of maltose metabolism\|+ |
| STM3307_517_551 | 0.11 | -1.38 | 1254830\|murA\|UDP-N-acetylglucosamine 1-carboxyvinyltransferase\|- |
| STM3308_209_243 | 0.03 | -1.69 | 1254831\|yrbA\|putative transcriptional regulator\|- |
| STM3309_97_131 | 0.01 | 1.57 | 1254832\|yrbB\|hypothetical protein\|- |
| STM3310_145_179 | 0.03 | 1.28 | 1254833\|yrbC\|putative transport protein\|- |
| STM3311_97_131 | 0.13 | -1.50 | 1254834\|yrbD\|putative transport protein\|- |
| STM3312_229_263 | 0.03 | -1.24 | 1254835\|yrbE\|putative transport protein\|- |
| STM3313_617_651 | 0.08 | 2.04 | 1254836\|yrbF\|putative ABC transporter ATP-binding protein YrbF\|- |
| STM3314_273_307 | 0.10 | 1.12 | 1254837\|yrbG\|putative calcium/sodium:proton antiporter\|+ |
| STM3315_929_963 | 0.04 | -1.31 | 1254838\|yrbH\|D-arabinose 5-phosphate isomerase\|+ |
| STM3316_161_195 | 0.18 | -1.15 | 1254839\|yrbI\|3-deoxy-D-manno-octulosonate 8-phosphate phosphatase\|+ |
| STM3317_385_419 | 0.06 | 1.14 | 1254840\|yrbK\|hypothetical protein\|+ |
| STM3318_129_163 | 0.65 | -1.03 | 1254841\|yhbN\|hypothetical protein\|+ |
| STM3319_565_599 | 0.14 | -1.27 | 1254842\|yhbG\|putative ABC transporter ATP-binding protein YhbG\|+ |
| STM3320_643_677 | 0.00 | -1.52 | 1254843\|rpoN\|RNA polymerase factor sigma-54\|+ |
| STM3321_217_251 | 0.09 | -1.58 | 1254844\|yhbH\|putative sigma(54) modulation protein\|+ |
| STM3323_457_491 | 0.02 | -1.69 | 1254846\|yhbJ\|hypothetical protein\|+ |
| STM3324_233_267 | 0.01 | 2.12 | 1254847\|ptsO\|phosphohistidinoprotein-hexose phosphotransferase component of N-regulated PTS system (Npr)\|+ |
| STM3325_369_403 | 0.94 | 1.01 | 1254848\|yrbL\|hypothetical protein\|+ |
| STM3326_465_499 | 0.49 | -1.17 | 1254849\|mtgA\|monofunctional biosynthetic peptidoglycan transglycosylase\|- |
| STM3327_129_163 | 0.16 | 1.22 | 1254850\|yhbL\|isoprenoid biosynthesis protein with amidotransferase-like domain\|- |
| STM3328_2026_2060 | 0.07 | 1.68 | 1254851\|arcB\|aerobic respiration control sensor protein ArcB\|- |
| STM3329_283_317 | 0.17 | -1.30 | 1254852\|yhcC\|putative FeS oxidoreductase\|- |
| STM3330_4230_4264 | 0.39 | 1.14 | 1254853\|gltB\|glutamate synthase subunit alpha\|+ |
| STM3331_1380_1414 | 0.39 | 1.58 | 1254854\|gltD\|glutamate synthase subunit beta\|+ |
| STM3334_1114_1148 | 0.79 | 1.04 | 1254857\|STM3334\|cytosine deaminase\|+ |
| STM3335_209_243 | 0.01 | -2.05 | 1254858\|yhcH\|putative cytoplasmic protein\|- |
| STM3336_129_163 | 0.00 | -3.13 | 1254859\|nanK\|N-acetylmannosamine kinase\|- |

| | | | |
|---|---|---|---|
| STM3337_193_227 | 0.03 | -1.48 | 1254860lnanElN-acetylmannosamine-6-phosphate 2-epimerasel- |
| STM3340_545_579 | 0.09 | 1.69 | 1254863lyhcKltranscriptional regulator NanRl- |
| STM3341_409_443 | 0.86 | 1.03 | 1254864lsspBlClpXP protease specificity-enhancing factorl- |
| STM3342_145_179 | 0.08 | 1.25 | 1254865lsspAlstringent starvation protein Al- |
| STM3343_33_67 | 0.06 | -1.88 | 1254866lSTM3343lputative cytoplasmic proteinl- |
| STM3344_321_355 | 0.13 | 1.34 | 1254867lrpsIl30S ribosomal protein S9l- |
| STM3345_129_163 | 0.01 | -2.90 | 1254868lrplMl50S ribosomal protein L13l- |
| STM3346_1086_1120 | 0.02 | -1.91 | 1254869lyhcMlputative ATPasel- |
| STM3347_33_67 | 0.13 | 1.31 | 1254870lyhcBlcytochrome d ubiquinol oxidase subunit IIIl+ |
| STM3348_1153_1187 | 0.01 | 2.26 | 1254871ldegQlserine endoproteasel+ |
| STM3349_456_490 | 0.16 | -1.09 | 1254872ldegSlserine endoproteasel+ |
| STM3350_417_451 | 0.07 | 1.35 | 1254873lSTM3350lputative inner membrane proteinl- |
| STM3353_2_37 | 0.03 | 1.26 | 1254876loadGloxaloacetate decarboxylase subunit gammal- |
| STM3354_97_131 | 0.26 | 1.29 | 1254877lSTM3354lL(+)-tartrate dehydratase subunit betal- |
| STM3355_129_163 | 0.81 | -1.02 | 1254878lSTM3355ltartrate dehydratase subunit alphal- |
| STM3356_302_336 | 0.13 | -1.32 | 1254879lSTM3356lputative cation transporterl- |
| STM3357_65_99 | 0.22 | 1.22 | 1254880lSTM3357lputative regulatory proteinl- |
| STM3358_105_139 | 0.09 | -1.20 | 1254881lSTM3358lputative regulatory proteinl- |
| STM3359_833_867 | 0.00 | -5.18 | 1254882lmdhlmalate dehydrogenasel- |
| STM3360_129_163 | 0.02 | -1.51 | 1254883largRlarginine repressorl+ |
| STM3361_129_163 | 0.88 | -1.02 | 1254884lyhcNlputative outer membrane proteinl+ |
| STM3362_97_131 | 0.00 | 3.30 | 1254885lSTM3362lputative periplasmic proteinl+ |
| STM3363_95_133 | 0.03 | 1.77 | 1254886lyhcOlputative cytoplasmic proteinl- |
| STM3364_1673_1707 | 0.82 | 1.04 | 1254887lyhcPlp-hydroxybenzoic acid efflux subunit AaeBl- |
| STM3365_161_195 | 0.12 | 1.30 | 1254888lyhcQlp-hydroxybenzoic acid efflux subunit AaeAl- |
| STM3366_132_166 | 0.03 | 2.37 | 1254889lyhcRlhypothetical proteinl- |
| STM3367_193_227 | 0.65 | 1.07 | 1254890lyhcSlputative DNA-binding transcriptional regulatorl+ |
| STM3368_1215_1249 | 0.03 | -1.54 | 1254891ltldDlmicrocin B17-processing proteinl- |
| STM3369_3314_3348 | 0.01 | -1.59 | 1254892lyhdPlhypothetical proteinl- |
| STM3370_727_761 | 0.21 | 1.20 | 1254893lcafAlribonuclease Gl- |
| STM3371_513_547 | 0.00 | -1.80 | 1254894lmaflMaf-like proteinl- |
| STM3372_241_275 | 0.03 | -1.70 | 1254895lmreDlrod shape-determining protein MreDl- |
| STM3373_374_408 | 0.03 | -1.42 | 1254896lmreClcell wall structural complex MreBCD transmembrane component MreCl- |
| STM3374_637_671 | 0.08 | -1.58 | 1254897lmreBlrod shape-determining proteinl- |
| STM3374.1n_61_95 | 0.05 | 1.93 | 2673730lSTM3374.1nlhypothetical proteinl+ |

| | | | |
|---|---|---|---|
| STM3375_1438_1472 | 0.04 | -1.32 | 1254898lyhdAlregulatory protein CsrDl- |
| STM3376_929_963 | 0.01 | 2.06 | 1254899lyhdHlputative oxidoreductasel+ |
| STM3377_870_904 | 0.02 | -1.43 | 1254900lSTM3377lputative sulfite oxidase subunit YedYl+ |
| STM3378_449_483 | 0.04 | -1.36 | 1254901lSTM3378lputative sulfite oxidase subunit YedZl+ |
| STM3379_401_435 | 0.04 | -1.81 | 1254902laccBlacetyl-CoA carboxylase biotin carboxyl carrier protein subunitl+ |
| STM3380_863_897 | 0.83 | -1.06 | 1254903laccClacetyl-CoA carboxylase biotin carboxylase subunitl+ |
| STM3381_65_99 | 0.06 | -1.27 | 1254904lyhdTlhypothetical proteinl+ |
| STM3382_1093_1127 | 0.06 | -1.41 | 1254905lpanFlsodium/panthothenate symporterl+ |
| STM3383_237_271 | 0.83 | -1.05 | 1254906lprmAlribosomal protein L11 methyltransferasel+ |
| STM3385_25_59 | 0.06 | -1.30 | 1254908lfislDNA-binding protein Fisl+ |
| STM3386_697_731 | 0.11 | 1.85 | 1254909lyhdJlputative methyltransferasel+ |
| STM3387_81_115 | 0.28 | 1.13 | 1254910lyhdUlputative periplasmic proteinl+ |
| STM3388_1101_1135 | 0.05 | 2.51 | 1254911lSTM3388lputative signal transduction proteinl+ |
| STM3389_81_115 | 0.12 | 1.13 | 1254912lenvRlDNA-binding transcriptional regulatorl- |
| STM3390_255_289 | 0.36 | 1.09 | 1254913lacrElacriflavine resistance protein E precursorl+ |
| STM3391_2563_2597 | 0.30 | 1.11 | 1254914lacrFlmultidrug transport proteinl+ |
| STM3392_73_107 | 0.00 | 12.55 | 1254915lyhdVlputative outer membrane lipoproteinl+ |
| STM3399_225_259 | 0.01 | -2.00 | 1254922lyrdAlputative ferripyochelin-binding proteinl+ |
| STM3400_17_51 | 0.15 | 1.35 | 1254923lyrdBlputative periplasmic proteinl- |
| STM3401_433_467 | 0.03 | -1.44 | 1254924laroElshikimate 5-dehydrogenasel- |
| STM3402_446_480 | 0.09 | -1.47 | 1254925lyrdClputative ribosome maturation factorl- |
| STM3403_113_147 | 0.07 | -1.19 | 1254926lyrdDlputative DNA topoisomerasel- |
| STM3404_97_131 | 0.01 | -1.59 | 1254927lsmglhypothetical proteinl- |
| STM3405_846_880 | 0.03 | -1.47 | 1254928lsmflhypothetical proteinl- |
| STM3406_161_195 | 0.06 | 1.14 | 1254929ldeflpeptide deformylasel+ |
| STM3407_393_427 | 0.26 | 1.12 | 1254930lfmtlmethionyl-tRNA formyltransferasel+ |
| STM3409_1082_1116 | 0.07 | 1.25 | 1254932ltrkAlpotassium transporter peripheral membrane componentl+ |
| STM3410_193_227 | 0.00 | 1.30 | 1254933lmscLllarge-conductance mechanosensitive channell+ |
| STM3411_117_152 | 0.04 | 1.65 | 1254934lSTM3411lputative cytoplasmic proteinl- |
| STM3412_81_115 | 0.07 | 1.39 | 1254935lzntRlzinc-responsive transcriptional regulatorl- |
| STM3413_241_275 | 0.01 | 2.01 | 1254936lyhdNlhypothetical proteinl- |
| STM3414_9_43 | 0.02 | -1.87 | 1254937lrplQl50S ribosomal protein L17l- |
| STM3415_769_803 | 0.00 | -1.91 | 1254938lrpoAlDNA-directed RNA polymerase subunit alphal- |
| STM3416_545_579 | 0.02 | -2.46 | 1254939lrpsDl30S ribosomal protein S4l- |
| STM3417_329_363 | 0.10 | 1.84 | 1254940lrpsKl30S ribosomal protein S11l- |
| STM3418_233_267 | 0.01 | -1.81 | 1254941lrpsMl30S ribosomal protein S13l- |

| | | | |
|---|---|---|---|
| STM3419_33_67 | 0.80 | -1.05 | 1254942lrpmJl50S ribosomal protein L36l- |
| STM3420_333_367 | 0.01 | -2.48 | 1254943lsecYlpreprotein translocase subunit SecYl- |
| STM3422_17_51 | 0.01 | -3.25 | 1254945lrpmDl50S ribosomal protein L30l- |
| STM3423_209_243 | 0.01 | -2.99 | 1254946lrpsEl30S ribosomal protein S5l- |
| STM3424_97_131 | 0.04 | -2.39 | 1254947lrplRl50S ribosomal protein L18l- |
| STM3425_137_171 | 0.01 | -3.17 | 1254948lrplFl50S ribosomal protein L6l- |
| STM3426_357_391 | 0.02 | -1.88 | 1254949lrpsHl30S ribosomal protein S8l- |
| STM3427.S_97_131 | 0.02 | -3.29 | 1254950lrpsNl30S ribosomal subunit protein S14l- |
| STM3428_353_387 | 0.01 | -4.21 | 1254951lrplEl50S ribosomal protein L5l- |
| STM3429_233_267 | 0.01 | -1.63 | 1254952lrplXl50S ribosomal protein L24l- |
| STM3430_33_67 | 0.00 | -3.80 | 1254953lrplNl50S ribosomal protein L14l- |
| STM3431_129_163 | 0.03 | -2.74 | 1254954lrpsQl30S ribosomal protein S17l- |
| STM3432_143_177 | 0.02 | -3.00 | 1254955lrpmCl50S ribosomal protein L29l- |
| STM3433_257_291 | 0.01 | -2.95 | 1254956lrplPl50S ribosomal protein L16l- |
| STM3434_385_419 | 0.03 | -2.58 | 1254957lrpsCl30S ribosomal protein S3l- |
| STM3435_177_211 | 0.00 | -8.12 | 1254958lrplVl50S ribosomal protein L22l- |
| STM3436_121_155 | 0.01 | -2.85 | 1254959lrpsSl30S ribosomal protein S19l- |
| STM3437_225_259 | 0.01 | -5.23 | 1254960lrplBl50S ribosomal protein L2l- |
| STM3438_153_187 | 0.02 | -1.66 | 1254961lrplWl50S ribosomal protein L23l- |
| STM3439_529_563 | 0.01 | -2.44 | 1254962lrplDl50S ribosomal protein L4l- |
| STM3440_385_419 | 0.00 | -4.72 | 1254963lrplCl50S ribosomal protein L3l- |
| STM3441_225_259 | 0.01 | -2.58 | 1254964lrpsJl30S ribosomal protein S10l- |
| STM3443_315_349 | 0.16 | -1.29 | 1254966lbfrlbacterioferritin  iron storage and detoxification proteinl- |
| STM3444_105_139 | 0.36 | 1.17 | 1254967lbfdlbacterioferritin-associated ferredoxinl- |
| STM3445_972_1006 | 0.04 | -1.84 | 1254968ltuflelongation factor Tul- |
| STM3446_1628_1662 | 0.04 | -1.97 | 1254969lfusAlelongation factor Gl- |
| STM3447_193_227 | 0.03 | -1.89 | 1254970lrpsGl30S ribosomal protein S7l- |
| STM3448_321_355 | 0.05 | -1.81 | 1254971lrpsLl30S ribosomal protein S12l- |
| STM3449_217_252 | 0.66 | 1.05 | 1254972lyheLlhypothetical proteinl- |
| STM3450_165_199 | 0.04 | -1.56 | 1254973lyheMlhypothetical proteinl- |
| STM3451_117_151 | 0.09 | -1.59 | 1254974lyheNlhypothetical proteinl- |
| STM3452_105_139 | 0.09 | -1.51 | 1254975lyheOlputative regulatory proteinl- |
| STM3453_697_731 | 0.58 | 1.09 | 1254976lfkpAlFKBP-type peptidyl-prolyl cis-trans isomerase (rotamase)l- |
| STM3454_156_190 | 0.05 | 2.14 | 1254977lslyXlhypothetical proteinl+ |
| STM3455_257_291 | 0.23 | 1.32 | 1254978lslyDlFKBP-type peptidyl-prolyl cis-trans isomerase (rotamase)l- |
| STM3456_105_139 | 0.74 | -1.09 | 1254979lyheVlputative cytoplasmic proteinl- |

| | | | |
|---|---|---|---|
| STM3457_1639_1673 | 0.03 | -1.21 | 1254980lkefBlglutathione-regulated potassium-efflux system protein KefBl- |
| STM3458_97_131 | 0.08 | -1.27 | 1254981lyheRlglutathione-regulated potassium-efflux system ancillary protein KefGl- |
| STM3459_1261_1295 | 0.04 | -1.73 | 1254982lyheSlputative ABC transporter ATP-binding proteinl+ |
| STM3460_97_131 | 0.00 | -2.50 | 1254983lSTM3460lpseudol- |
| STM3461_161_195 | 0.06 | 1.45 | 1254984lSTM3461lhypothetical proteinl- |
| STM3462_389_423 | 0.01 | 2.03 | 1254985lyheTlpredicted hydrolasel+ |
| STM3463_137_171 | 0.07 | -1.21 | 1254986lyheUlhypothetical proteinl+ |
| STM3464_545_579 | 0.11 | 1.23 | 1254987lprkBlputative phosphoribulokinasel+ |
| STM3465_173_207 | 0.00 | -2.69 | 1254988lyhfAlhypothetical proteinl- |
| STM3466_25_59 | 0.36 | 1.08 | 1254989lcrplcAMP-regulatory proteinl+ |
| STM3468_515_549 | 0.05 | 1.61 | 1254991largDlbifunctional N-succinyldiaminopimelate-aminotransferase/acetylornithine transaminase proteinl- |
| STM3469_289_323 | 0.98 | -1.00 | 1254992lpabAlpara-aminobenzoate synthase component IIl- |
| STM3470_329_363 | 0.39 | 1.12 | 1254993lficlcell filamentation protein Ficl- |
| STM3471_129_163 | 0.54 | -1.11 | 1254994lyhfGlhypothetical proteinl- |
| STM3472_533_567 | 0.00 | 4.65 | 1254995lppiAlpeptidyl-prolyl cis-trans isomerase A (rotamase A)l- |
| STM3473_463_497 | 0.67 | 1.10 | 1254996lyhfClhypothetical proteinl+ |
| STM3474_1753_1787 | 0.04 | 1.39 | 1254997lnirBlnitrite reductase large subunitl+ |
| STM3475_81_115 | 0.02 | -1.28 | 1254998lnirDlnitrite reductase small subunitl+ |
| STM3476_197_231 | 0.40 | 1.06 | 1254999lnirClnitrite transporter NirCl+ |
| STM3477_1271_1305 | 0.01 | -1.90 | 1255000lcysGlsiroheme synthasel+ |
| STM3478_5535_5569 | 0.27 | 1.08 | 1255001lbigAlputative surface-exposed virulence proteinl+ |
| STM3480_38_72 | 0.17 | 1.90 | 1255003lyhfLlputative outer membrane lipoproteinl+ |
| STM3481_278_312 | 0.21 | -1.38 | 1255004ltrpSltryptophanyl-tRNA synthetasel- |
| STM3482_385_419 | 0.71 | 1.04 | 1255005lgphlphosphoglycolate phosphatasel- |
| STM3483_105_139 | 0.21 | -1.17 | 1255006lrpelribulose-phosphate 3-epimerasel- |
| STM3484_417_451 | 0.07 | 1.39 | 1255007ldamlDNA adenine methylasel- |
| STM3485_631_665 | 0.08 | -1.73 | 1255008ldamXlhypothetical proteinl- |
| STM3486_642_676 | 0.01 | -2.43 | 1255009laroBl3-dehydroquinate synthasel- |
| STM3487_449_483 | 0.02 | -2.20 | 1255010laroKlshikimate kinase Il- |
| STM3488_1000_1034 | 0.19 | 1.08 | 1255011lhofQlpredicted fimbrial transporterl- |
| STM3489_129_163 | 0.84 | -1.05 | 1255012lyrfAlputative inner membrane proteinl- |
| STM3490_363_397 | 0.80 | -1.03 | 1255013lyrfBlputative inner membrane proteinl- |
| STM3491_481_515 | 0.65 | -1.18 | 1255014lyrfClputative inner membrane proteinl- |
| STM3492_325_359 | 0.73 | 1.07 | 1255015lyrfDlputative periplasmic proteinl- |

| | | | |
|---|---|---|---|
| STM3493_2190_2224 | 0.15 | -1.34 | 1255016lmrcAlpeptidoglycan synthetasel+ |
| STM3494.S_145_179 | 0.86 | 1.02 | 1255017lnudElADP-ribose diphosphatase NudEl- |
| STM3495_1246_1280 | 0.87 | -1.03 | 1255018lyrfFlputative inner membrane proteinl+ |
| STM3496_177_211 | 0.76 | -1.02 | 1255019lyrfGlputative hydrolasel+ |
| STM3497_353_387 | 0.38 | -1.07 | 1255020lyrfHlribosome-associated heat shock protein Hsp15l+ |
| STM3498_65_99 | 0.04 | 1.40 | 1255021lhslOlHsp33-like chaperoninl+ |
| STM3499_1111_1145 | 0.03 | -1.60 | 1255022lyhgElputative inner membrane proteinl- |
| STM3500_954_988 | 0.00 | -3.82 | 1255023lpckAlphosphoenolpyruvate carboxykinasel+ |
| STM3501_930_964 | 0.02 | -1.21 | 1255024lenvZlosmolarity sensor proteinl- |
| STM3502_193_227 | 0.01 | 1.70 | 1255025lompRlosmolarity response regulatorl- |
| STM3503_429_463 | 0.01 | 1.55 | 1255026lgreBltranscription elongation factor GreBl+ |
| STM3504_1969_2003 | 0.16 | -1.22 | 1255027lyhgFlputative RNase Rl+ |
| STM3505_145_179 | 0.01 | 2.45 | 1255028lfeoAlferrous iron transport protein Al+ |
| STM3506_1896_1930 | 0.64 | 1.15 | 1255029lfeoBlferrous iron transport protein Bl+ |
| STM3507_89_123 | 0.33 | 1.13 | 1255030lyhgGlputative cytoplasmic proteinl+ |
| STM3508_841_875 | 0.02 | 1.67 | 1255031lSTM3508lputative cytoplasmic proteinl+ |
| STM3509_649_683 | 0.13 | 1.39 | 1255032lbioHlcarboxylesterase BioHl- |
| STM3510_17_51 | 0.12 | 1.17 | 1255033lyhgHlgluconate periplasmic binding proteinl+ |
| STM3511_449_483 | 0.02 | 5.17 | 1255034lyhgIlpredicted gluconate transport-associated proteinl+ |
| STM3512_846_880 | 0.04 | -1.58 | 1255035lgntTlhigh-affinity gluconate permeasel+ |
| STM3513_1856_1890 | 0.01 | 1.45 | 1255036lmalQl4-alpha-glucanotransferasel- |
| STM3514_1427_1461 | 0.06 | 1.54 | 1255037lmalPlmaltodextrin phosphorylasel- |
| STM3515_1707_1741 | 0.01 | -3.18 | 1255038lmalTltranscriptional regulator MalTl+ |
| STM3516_153_187 | 0.70 | 1.06 | 1255039lSTM3516lputative cytoplasmic proteinl- |
| STM3517_149_183 | 0.44 | -1.09 | 1255040lSTM3517lputative DNA-damage-inducibile proteinl- |
| STM3518_981_1015 | 0.06 | 1.42 | 1255041lrtcAlRNA 3'-terminal-phosphate cyclasel- |
| STM3519_1099_1133 | 0.64 | 1.08 | 1255042lrtcBlputative cytoplasmic proteinl- |
| STM3520_17_51 | 0.01 | 2.10 | 1255043lSTM3520lpseudol- |
| STM3519.1N_124_158 | 0.48 | 1.10 | 2673723lSTM3519.1Nl-l- |
| STM3521_1291_1325 | 0.02 | 1.59 | 1255044lSTM3521lputative ribonucleoprotein related-proteinl- |
| STM3523_513_547 | 0.02 | -1.84 | 1255046lglpRlDNA-binding transcriptional repressorl- |
| STM3524_305_339 | 0.21 | -1.12 | 1255047lglpGlpredicted intramembrane serine proteasel- |
| STM3525_65_99 | 0.03 | -1.41 | 1255048lglpElthiosulfate sulfurtransferasel- |
| STM3526_1318_1352 | 0.26 | 1.13 | 1255049lglpDlglycerol-3-phosphate dehydrogenasel+ |
| STM3527_65_99 | 0.10 | -1.11 | 1255050lSTM3527lhypothetical proteinl- |
| STM3528_1265_1299 | 0.03 | 1.40 | 1255051lSTM3528lputative periplasmic phosphate-binding proteinl- |

| | | | |
|---|---|---|---|
| STM3529_367_401 | 0.04 | -1.74 | 1255052\|gldA\|glycerol dehydrogenase\|- |
| STM3530_975_1009 | 0.33 | 1.26 | 1255053\|STM3530\|pseudo\|+ |
| STM3533_201_235 | 0.06 | 1.41 | 1255056\|STM3533\|putative transcriptional regulator\|- |
| STM3534_2345_2379 | 0.14 | -1.17 | 1255057\|glgP\|glycogen phosphorylase\|- |
| STM3535_1043_1077 | 0.02 | -1.62 | 1255058\|glgA\|glycogen synthase\|- |
| STM3536_905_939 | 0.17 | -1.12 | 1255059\|glgC\|glucose-1-phosphate adenylyltransferase\|- |
| STM3537_1074_1108 | 0.02 | 1.50 | 1255060\|glgX\|glycogen debranching enzyme\|- |
| STM3538_1484_1518 | 0.81 | -1.02 | 1255061\|glgB\|glycogen branching enzyme\|- |
| STM3539_1020_1054 | 0.97 | 1.01 | 1255062\|asd\|aspartate-semialdehyde dehydrogenase\|- |
| STM3540_73_107 | 0.04 | 2.37 | 2673721\|STM3540\|-\|+ |
| STM3541_598_632 | 0.43 | -1.05 | 1255064\|gntU\|gluconate transporter  low affinity GNT 1 system\|- |
| STM3542_175_209 | 0.03 | 2.35 | 1255065\|gntK\|gluconate kinase 1\|- |
| STM3543_209_243 | 0.01 | 1.94 | 1255066\|gntR\|gluconate operon transcriptional repressor\|- |
| STM3544_641_675 | 0.14 | 1.39 | 1255067\|yhhW\|putative cytoplasmic protein\|- |
| STM3545_871_905 | 0.05 | -1.43 | 1255068\|yhhX\|putative oxidoreductase\|- |
| STM3546_321_355 | 0.53 | 1.06 | 1255069\|yhhY\|putative acetyltransferase YhhY\|+ |
| STM3547.Sc_113_147 | 0.01 | 1.55 | 1255070\|STM3547.Sc\|putative transcriptional regulator\|+ |
| STM3548_609_643 | 0.06 | 2.06 | 1255071\|STM3548\|putative cytoplasmic protein\|+ |
| STM3549_97_131 | 0.08 | 2.04 | 1255072\|STM3549\|putative inner membrane protein\|+ |
| STM3550_836_870 | 0.09 | 1.16 | 1255073\|STM3550\|putative phosphotriesterase\|+ |
| STM3551_880_914 | 0.33 | -1.09 | 1255074\|ggt\|gamma-glutamyltranspeptidase periplasmic precursor\|- |
| STM3552_201_235 | 0.12 | 1.32 | 1255075\|yhhA\|hypothetical protein\|+ |
| STM3553_93_127 | 0.03 | -1.64 | 1255076\|ugpQ\|cytoplasmic glycerophosphodiester phosphodiesterase\|- |
| STM3554_552_586 | 0.18 | -1.13 | 1255077\|ugpC\|glycerol-3-phosphate transporter ATP-binding subunit\|- |
| STM3555_641_675 | 0.01 | -1.41 | 1255078\|ugpE\|glycerol-3-phosphate transporter membrane protein\|- |
| STM3557_1118_1152 | 0.01 | -2.39 | 1255080\|ugpB\|glycerol-3-phosphate transporter periplasmic binding protein\|- |
| STM3558_273_307 | 0.46 | 1.07 | 1255081\|STM3558\|death-on-curing protein\|- |
| STM3559_3_37 | 0.21 | -1.31 | 1255082\|yhhV\|putative cytoplasmic protein\|- |
| STM3560_521_555 | 0.12 | -1.23 | 1255083\|livF\|leucine/isoleucine/valine transporter ATP-binding subunit\|- |
| STM3561_49_83 | 0.41 | 1.07 | 1255084\|livG\|leucine/isoleucine/valine transporter ATP-binding subunit\|- |
| STM3562_663_697 | 0.85 | 1.02 | 1255085\|livM\|leucine/isoleucine/valine transporter permease subunit\|- |
| STM3563_657_691 | 0.06 | -1.41 | 1255086\|livH\|branched-chain amino acid transporter permease subunit LivH\|- |
| STM3564_191_225 | 0.72 | 1.03 | 1255087\|livK\|high-affinity branched-chain amino acid transporter\|- |
| STM3565_25_59 | 0.01 | 1.64 | 1255088\|yhhK\|putative acetyltransferase\|+ |
| STM3566_85_120 | 0.00 | 1.64 | 1255089\|STM3566\|putative cytoplasmic protein\|+ |
| STM3567_259_293 | 0.82 | 1.03 | 1255090\|livJ\|high-affinity branched-chain amino acid transporter\|- |

| | | | |
|---|---|---|---|
| STM3568_353_387 | 0.14 | 1.29 | 1255091\|rpoH\|RNA polymerase factor sigma-32\|- |
| STM3569_281_315 | 0.01 | 2.40 | 1255092\|ftsX\|cell division protein FtsX\|- |
| STM3570_577_611 | 0.04 | 1.84 | 1255093\|ftsE\|cell division protein FtsE\|- |
| STM3571_1357_1391 | 0.04 | -1.13 | 1255094\|ftsY\|cell division protein FtsY\|- |
| STM3572_529_563 | 0.96 | 1.01 | 1255095\|yhhF\|predicted methyltransferase\|+ |
| STM3573_13_47 | 0.03 | 1.27 | 1255096\|yhhL\|hypothetical protein\|+ |
| STM3574_129_163 | 0.21 | -1.18 | 1255097\|yhhM\|putative inner membrane protein\|- |
| STM3575_385_419 | 0.58 | 1.03 | 1255098\|yhhN\|putative inner membrane protein\|+ |
| STM3576_1712_1746 | 0.27 | -1.09 | 1255099\|zntA\|zinc/cadmium/mercury/lead-transporting ATPase\|+ |
| STM3577_813_847 | 0.25 | 1.14 | 1255100\|tcp\|methyl-accepting transmembrane citrate/phenol chemoreceptor\|+ |
| STM3578_89_123 | 0.00 | 1.86 | 1255101\|yhhP\|cell developmental protein SirA\|- |
| STM3579_249_283 | 0.35 | -1.14 | 1255102\|yhhQ\|hypothetical protein\|+ |
| STM3580_345_379 | 0.01 | 2.46 | 1255103\|STM3580\|hypothetical protein\|+ |
| STM3581.S_1021_1055 | 0.01 | 1.97 | 1255104\|yhhS\|hypothetical protein\|- |
| STM3582_83_117 | 0.90 | -1.03 | 1255105\|yhhT\|putative permease\|+ |
| STM3583_33_67 | 0.03 | 1.67 | 1255106\|acpT\|holo-(acyl carrier protein) synthase 2\|+ |
| STM3584_17_51 | 0.13 | 1.16 | 1255107\|nikR\|nickel responsive regulator\|+ |
| STM3586.S_1791_1825 | 0.18 | -1.11 | 1255109\|yhiH\|putative ABC-type multidrug transport system ATPase component\|- |
| STM3587_165_199 | 0.03 | 1.37 | 1255110\|yhiI\|hypothetical protein\|- |
| STM3588_774_808 | 0.04 | -1.44 | 1255111\|yhiN\|putative periplasmic protein\|- |
| STM3589_914_948 | 0.03 | -1.34 | 1255112\|pitA\|low-affinity phosphate transporter\|+ |
| STM3590_65_99 | 0.02 | 1.74 | 1255113\|uspB\|universal stress protein UspB\|- |
| STM3591_161_195 | 0.11 | 1.34 | 1255114\|uspA\|universal stress protein A\|+ |
| STM3592_1111_1145 | 0.04 | -1.14 | 1255115\|yhiP\|inner membrane transporter YhiP\|+ |
| STM3593_233_267 | 0.40 | -1.12 | 1255116\|yhiQ\|putative methyltransferase\|- |
| STM3594_1908_1942 | 0.12 | 1.34 | 1255117\|prlC\|oligopeptidase A\|- |
| STM3595_601_635 | 0.00 | 2.33 | 1255118\|STM3595\|putative phosphatase\|- |
| STM3596_33_67 | 0.11 | 2.14 | 1255119\|yhiR\|putative cytoplasmic protein\|+ |
| STM3597_386_420 | 0.83 | 1.05 | 1255120\|gor\|glutathione reductase\|+ |
| STM3598_573_607 | 0.05 | 2.14 | 1255121\|STM3598\|putative L-asparaginase\|- |
| STM3599_705_739 | 0.10 | -1.29 | 1255122\|STM3599\|anaerobic C4-dicarboxylate transporter\|- |
| STM3600_33_67 | 0.81 | 1.03 | 1255123\|STM3600\|putative sugar kinase\|- |
| STM3601_289_323 | 0.10 | -1.21 | 1255124\|STM3601\|putative phosphosugar isomerase\|- |
| STM3602_257_291 | 0.48 | -1.08 | 1255125\|STM3602\|putative regulatory protein\|- |

| | | | |
|---|---|---|---|
| STM3603_1195_1229 | 0.00 | 2.43 | 1255126\|treF\|trehalase\|+ |
| STM3604_1071_1105 | 0.09 | 1.22 | 1255127\|STM3604\|putative inner membrane protein\|- |
| STM3605_97_131 | 0.17 | 1.22 | 1255128\|STM3605\|putative phage endolysin\|+ |
| STM3606_273_307 | 0.01 | 2.48 | 1255129\|yhjB\|putative transcriptional regulator\|- |
| STM3607_705_739 | 0.07 | 1.43 | 1255130\|yhjC\|putative transcriptional regulator\|+ |
| STM3608_606_640 | 0.27 | 1.56 | 1255131\|yhjD\|putative tRNA-processing ribonuclease\|+ |
| STM3609_1140_1174 | 0.00 | -2.55 | 1255132\|yhjE\|putative transport protein\|+ |
| STM3610_1114_1148 | 0.22 | -1.12 | 1255133\|yhjG\|putative inner membrane protein\|- |
| STM3611_385_419 | 0.51 | -1.10 | 1255134\|yhjH\|EAL domain containing protein involved in flagellar function\|- |
| STM3612_289_323 | 0.01 | -3.55 | 1255135\|kdgK\|ketodeoxygluconokinase\|+ |
| STM3613_809_843 | 0.03 | -1.96 | 1255136\|yhjJ\|putative Zn-dependent peptidase\|- |
| STM3614_896_930 | 0.01 | -8.22 | 1255137\|dctA\|C4-dicarboxylate transporter DctA\|- |
| STM3615_1743_1777 | 0.97 | 1.01 | 1255138\|yhjK\|putative diguanylate cyclase/phosphodiesterase\|- |
| STM3616_2768_2802 | 0.18 | -1.21 | 1255139\|yhjL\|cellulose synthase subunit BcsC\|- |
| STM3617_511_545 | 0.07 | -1.34 | 1255140\|STM3617\|endo-1 4-D-glucanase\|- |
| STM3618_1750_1784 | 0.39 | -1.13 | 1255141\|yhjN\|cellulose synthase regulator protein\|- |
| STM3619_1882_1916 | 0.39 | -1.15 | 1255142\|bcsA\|cellulose synthase catalytic subunit\|- |
| STM3620_433_467 | 0.09 | 1.11 | 1255143\|yhjQ\|cell division protein\|- |
| STM3621_5_39 | 0.69 | 1.03 | 1255144\|yhjR\|putative cytoplasmic protein\|- |
| STM3622_717_751 | 0.03 | 1.59 | 1255145\|yhjS\|putative cytoplasmic protein\|+ |
| STM3623_125_159 | 0.01 | 2.62 | 1255146\|yhjT\|putative inner membrane protein\|+ |
| STM3624_1449_1483 | 0.30 | 1.11 | 1255147\|yhjU\|putative inner membrane protein\|+ |
| STM3624.1N_239_273 | 0.74 | 1.10 | 2673734\|STM3624.1N\|-\|- |
| STM3624A_97_131 | 0.05 | -1.66 | 1255148\|STM3624A\|cystathionine gamma-synthase\|+ |
| STM3625_1140_1174 | 0.06 | 1.42 | 1255149\|yhjV\|putative transport protein\|+ |
| STM3626_919_953 | 0.02 | -2.04 | 1255150\|dppF\|dipeptide transporter ATP-binding subunit\|- |
| STM3627_401_435 | 0.00 | -1.70 | 1255151\|dppD\|dipeptide transporter ATP-binding subunit\|- |
| STM3628_369_403 | 0.02 | -1.53 | 1255152\|dppC\|dipeptide transporter\|- |
| STM3629_533_567 | 0.00 | -3.54 | 1255153\|dppB\|dipeptide transporter permease DppB\|- |
| STM3630_777_811 | 0.88 | 1.03 | 1255154\|dppA\|dipeptide transport protein\|- |
| STM3631_727_761 | 0.05 | 1.23 | 1255155\|STM3631\|putative xanthine permease\|- |
| STM3632_926_960 | 0.17 | -1.35 | 1255156\|STM3632\|hypothetical protein\|- |
| STM3635_1149_1183 | 0.40 | 1.18 | 1255159\|yhjW\|phosphoethanolamine transferase\|- |
| STM3636_145_179 | 0.01 | 2.00 | 1255160\|lpfE\|long polar fimbrial minor protein\|- |
| STM3638_2106_2140 | 0.05 | -1.30 | 1255162\|lpfC\|long polar fimbrial outer membrane usher protein\|- |
| STM3640_353_387 | 0.04 | 1.23 | 1255164\|lpfA\|long polar fimbrial protein A precursor\|- |

| | | | |
|---|---|---|---|
| STM3641_385_419 | 0.79 | 1.04 | 1255165lyhjYlputative lipasel- |
| STM3642_97_131 | 0.96 | 1.00 | 1255166ltagl3-methyl-adenine DNA glycosylase Il+ |
| STM3643_81_115 | 0.01 | -1.90 | 1255167lyiaClhypothetical proteinl+ |
| STM3644_1399_1433 | 0.06 | -1.40 | 1255168lbisClbiotin sulfoxide reductasel- |
| STM3645_625_659 | 0.01 | 3.07 | 1255169lyiaDlpredicted outer membrane lipoproteinl+ |
| STM3646_209_243 | 0.00 | -1.60 | 1255170lyiaEl2-hydroxyacid dehydrogenasel+ |
| STM3647_513_547 | 0.37 | 1.09 | 1255171lyiaFlputative outer membrane lipoproteinl- |
| STM3648_221_255 | 0.57 | 1.14 | 1255172lyiaGlputative transcriptional regulatorl+ |
| STM3649_66_100 | 0.01 | 2.16 | 1255173lcspAlmajor cold shock proteinl+ |
| STM3650_161_195 | 0.06 | 1.99 | 1255174lSTM3650lhypothetical proteinl+ |
| STM3651_193_227 | 0.01 | 2.20 | 1255175lSTM3651lputative acetyltransferasel- |
| STM3652_215_249 | 0.01 | 1.94 | 1255176lSTM3652lputative cytoplasmic proteinl- |
| STM3653_129_163 | 0.07 | 1.22 | 1255177lSTM3653lputative acetyltransferasel- |
| STM3654_53_87 | 0.78 | -1.02 | 1255178lSTM3654lpseudol- |
| STM3655_1615_1649 | 0.03 | -1.95 | 1255179lglySlglycyl-tRNA synthetase subunit betal- |
| STM3656_785_819 | 0.01 | -1.49 | 1255180lglyQlglycyl-tRNA synthetase subunit alphal- |
| STM3657_25_59 | 0.04 | -1.47 | 1255181lSTM3657lputative outer membrane lipoproteinl- |
| STM3658_693_727 | 0.88 | -1.01 | 1255182lyiaHlputative inner membrane proteinl+ |
| STM3659_153_187 | 0.02 | 1.41 | 1255183lyiaBlputative inner membrane proteinl- |
| STM3660_1384_1418 | 0.04 | 1.28 | 1255184lxylBlxylulokinasel- |
| STM3662_1140_1174 | 0.01 | -1.66 | 1255186lxylRlxylose operon regulatory proteinl+ |
| STM3663_769_803 | 0.05 | 1.47 | 1255187lbaxlhypothetical proteinl- |
| STM3664_1157_1191 | 0.00 | 1.29 | 1255188lmalSlperiplasmic alpha-amylase precursorl+ |
| STM3666_241_275 | 0.02 | 1.95 | 1255190lysaAlputative oxidoreductasel- |
| STM3667_465_499 | 0.39 | -1.15 | 1255191lyiaJltranscriptional repressorl- |
| STM3668_489_523 | 0.88 | -1.03 | 1255192lyiaKl2 3-diketo-L-gulonate reductasel+ |
| STM3669_173_207 | 0.03 | 1.38 | 1255193lyiaLlputative cytoplasmic proteinl+ |
| STM3670_273_307 | 0.10 | 1.64 | 1255194lSTM3670lputative chemotaxis proteinl+ |
| STM3672_1175_1209 | 0.11 | 2.10 | 1255196lyiaNlhypothetical proteinl+ |
| STM3673_577_611 | 0.02 | 1.73 | 1255197lyiaOlputative periplasmic dicarboxylate-binding proteinl+ |
| STM3675_481_515 | 0.45 | -1.09 | 1255199lsgbHl3-keto-L-gulonate-6-phosphate decarboxylasel+ |
| STM3676_513_547 | 0.49 | 1.15 | 1255200lsgbUlputative L-xylulose 5-phosphate 3-epimerasel+ |
| STM3677_417_451 | 0.67 | -1.05 | 1255201lsgbElL-ribulose-5-phosphate 4-epimerasel+ |
| STM3680_732_766 | 0.00 | -15.10 | 1255204laldBlaldehyde dehydrogenase Bl- |
| STM3681_609_643 | 0.01 | 1.86 | 1255205lSTM3681lputative transcriptional regulatorl+ |
| STM3682_1428_1462 | 0.12 | -1.13 | 1255206lselBlselenocysteinyl-tRNA-specific translation factorl- |

| | | | |
|---|---|---|---|
| STM3683_1001_1035 | 0.13 | -1.11 | 1255207lselAlselenocysteine synthasel- |
| STM3684_87_121 | 0.13 | -1.15 | 1255208lyibFlpredicted glutathione S-transferasel- |
| STM3685_1574_1608 | 0.01 | -4.19 | 1255209lmtlAlmannitol-specific enzyme IIABC componentl+ |
| STM3686_694_728 | 0.00 | -4.60 | 1255210lmtlDlmannitol-1-phosphate 5-dehydrogenasel+ |
| STM3687_193_227 | 0.00 | -4.38 | 1255211lmtlRlmannitol repressor proteinl+ |
| STM3688_33_67 | 0.04 | 1.81 | 1255212lSTM3688lputative cytoplasmic proteinl- |
| STM3689_141_175 | 0.01 | 2.10 | 1255213lyibLlhypothetical proteinl+ |
| STM3690_513_547 | 0.04 | 2.62 | 1255214lSTM3690lputative inner membrane lipoproteinl+ |
| STM3692_1009_1043 | 0.02 | -2.02 | 1255216llldPlL-lactate permeasel+ |
| STM3693_545_579 | 0.75 | -1.16 | 1255217llldRlDNA-binding transcriptional repressorl+ |
| STM3694_592_626 | 0.01 | -2.65 | 1255218llldDlL-lactate dehydrogenasel+ |
| STM3695_417_451 | 0.01 | -1.44 | 1255219lyibKlputative tRNA/rRNA methyltransferase YibKl+ |
| STM3696_70_104 | 0.98 | -1.00 | 1255220lSTM3696lputative transcriptional regulatorl- |
| STM3697_838_872 | 0.18 | 1.18 | 1255221lSTM3697lputative mandelate racemasel+ |
| STM3698_888_922 | 0.27 | 1.33 | 1255222lSTM3698lputative permeasel+ |
| STM3699_513_547 | 0.03 | 1.51 | 1255223lcysElserine acetyltransferasel- |
| STM3700_533_567 | 0.00 | -2.76 | 1255224lgpsAlNAD(P)H-dependent glycerol-3-phosphate dehydrogenasel- |
| STM3701_169_203 | 0.00 | -2.30 | 1255225lsecBlpreprotein translocase subunit SecBl- |
| STM3702_129_163 | 0.02 | -2.36 | 1255226lgrxClglutaredoxin 3l- |
| STM3703_25_59 | 0.02 | -2.18 | 1255227lyibNlputative rhodanese-like sulfurtransferasel- |
| STM3704_826_860 | 0.00 | 1.41 | 1255228lpmgIlphosphoglyceromutasel+ |
| STM3705_1082_1116 | 0.01 | -1.65 | 1255229lyibPlhypothetical proteinl+ |
| STM3706_513_547 | 0.23 | 1.25 | 1255230lyigQlputative periplasmic proteinl+ |
| STM3707_740_774 | 0.01 | 1.27 | 1255231lyibDlpredicted glycosyl transferasel- |
| STM3708_59_93 | 0.02 | 1.50 | 1255232ltdhlL-threonine 3-dehydrogenasel- |
| STM3709_582_616 | 0.01 | -2.33 | 1255233lkbll2-amino-3-ketobutyrate coenzyme A ligasel- |
| STM3710_305_339 | 0.05 | 1.33 | 1255234lrfaDlADP-L-glycero-D-mannoheptose-6-epimerasel+ |
| STM3711_48_82 | 0.45 | 1.06 | 1255235lrfaFlADP-heptose:LPS heptosyltransferase IIl+ |
| STM3712_817_851 | 0.14 | 1.21 | 1255236lrfaClADP-heptose:LPS heptosyl transferase Il+ |
| STM3713_856_890 | 0.28 | 1.34 | 1255237lrfaLlO-antigen ligasel+ |
| STM3714_947_981 | 0.02 | 2.23 | 1255238lrfaKlputative hexose transferasel- |
| STM3715_593_627 | 0.07 | 1.36 | 1255239lrfaZllipopolysaccharide core biosynthesis proteinl- |
| STM3716_445_479 | 0.05 | 1.52 | 1255240lrfaYllipopolysaccharide core biosynthesis proteinl- |
| STM3717_784_818 | 0.06 | 1.64 | 1255241lrfaJllipopolysaccharide glucosyltransferasel- |
| STM3718_480_514 | 0.03 | -1.60 | 1255242lrfaIllipopolysaccharide-alpha-1 3-D-galactosyltransferasel- |

| | | | |
|---|---|---|---|
| STM3719_865_899 | 0.21 | 1.16 | 1255243\|rfaB\|UDP-D-galactose:(glucosyl)lipopolysaccharide-1 6-D-galactosyltransferase\|- |
| STM3720_111_147 | 0.02 | 1.63 | 1255244\|yibR\|putative inner membrane protein\|- |
| STM3721_649_683 | 0.05 | 1.60 | 1255245\|rfaP\|lipopolysaccharide core biosynthetic protein\|- |
| STM3722_254_288 | 0.11 | 1.17 | 1255246\|rfaG\|glucosyltransferase I\|- |
| STM3723_740_774 | 0.27 | 1.20 | 1255247\|rfaQ\|lipopolysaccharide core biosynthesis protein\|- |
| STM3724_1175_1209 | 0.00 | -1.32 | 1255248\|kdtA\|3-deoxy-D-manno-octulosonic-acid transferase\|+ |
| STM3725_433_467 | 0.02 | -1.80 | 1255249\|coaD\|phosphopantetheine adenylyltransferase\|+ |
| STM3726_129_163 | 0.06 | 1.30 | 1255250\|mutM\|formamidopyrimidine-DNA glycosylase\|- |
| STM3727_117_151 | 0.01 | -2.37 | 1255251\|rpmG\|50S ribosomal protein L33\|- |
| STM3728_113_147 | 0.01 | -2.23 | 1255252\|rpmB\|50S ribosomal protein L28\|- |
| STM3729_577_611 | 0.01 | -1.77 | 1255253\|radC\|DNA repair protein RadC\|- |
| STM3730_686_720 | 0.10 | 1.59 | 1255254\|dfp\|bifunctional phosphopantothenoylcysteine decarboxylase/phosphopantothenate synthase\|+ |
| STM3731_145_179 | 0.17 | -1.28 | 1255255\|dut\|deoxyuridine 5'-triphosphate nucleotidohydrolase\|+ |
| STM3732_161_195 | 0.05 | 2.36 | 1255256\|slmA\|nucleoid occlusion protein\|+ |
| STM3733_509_543 | 0.56 | -1.07 | 1255257\|pyrE\|orotate phosphoribosyltransferase\|- |
| STM3734_65_99 | 0.04 | 1.16 | 1255258\|rph\|ribonuclease PH\|- |
| STM3735_177_211 | 0.04 | 1.54 | 1255259\|yicC\|hypothetical protein\|+ |
| STM3736_105_139 | 0.05 | -1.31 | 1255260\|STM3736\|putative transcriptional regulator\|- |
| STM3737_577_611 | 0.01 | 2.07 | 1255261\|STM3737\|putative Zn-dependent hydrolase\|+ |
| STM3738_393_427 | 0.94 | -1.02 | 1255262\|yigC\|putative inner membrane protein\|+ |
| STM3739_1023_1057 | 0.00 | -2.17 | 1255263\|ligB\|NAD-dependent DNA ligase LigB\|- |
| STM3740_81_115 | 0.45 | 1.13 | 1255264\|gmk\|guanylate kinase\|+ |
| STM3741_225_259 | 0.21 | -1.25 | 1255265\|rpoZ\|DNA-directed RNA polymerase subunit omega\|+ |
| STM3742_2073_2107 | 0.60 | 1.05 | 1255266\|spoT\|bifunctional (p)ppGpp synthetase II/ guanosine-3' 5'-bis pyrophosphate 3'-pyrophosphohydrolase\|+ |
| STM3743_513_547 | 0.40 | -1.23 | 1255267\|spoU\|tRNA guanosine-2'-O-methyltransferase\|+ |
| STM3744_1979_2013 | 0.05 | 1.15 | 1255268\|recG\|ATP-dependent DNA helicase RecG\|+ |
| STM3745_273_307 | 0.96 | -1.01 | 1255269\|STM3745\|putative cytoplasmic protein\|- |
| STM3746_655_689 | 0.02 | 1.40 | 1255270\|gltS\|glutamate transport protein\|- |
| STM3747_993_1027 | 0.01 | -3.32 | 1255271\|yicE\|putative purine/xanthine transport protein\|+ |
| STM3748_919_953 | 0.01 | 1.48 | 1255272\|yicH\|putative inner membrane protein\|+ |
| STM3749_2104_2138 | 0.36 | 1.08 | 1255273\|yicI\|alpha-xylosidase YicI\|- |
| STM3750_992_1026 | 0.29 | 1.09 | 1255274\|yicJ\|putative transporter\|- |
| STM3752_305_340 | 0.01 | 1.45 | 1255276\|STM3752\|putative cytoplasmic protein\|- |

| | | | |
|---|---|---|---|
| STM3753_512_546 | 0.18 | 1.09 | 1255277\|sugR\|ATP binding protein\|+ |
| STM3754_257_291 | 0.03 | 1.32 | 1255278\|STM3754\|putative cytoplasmic protein\|+ |
| STM3755_871_905 | 0.02 | 1.72 | 1255279\|rhuM\|putative cytoplasmic protein\|+ |
| STM3755.1N_80_115 | 0.01 | 1.55 | 2673725\|STM3755.1N\|-\|- |
| STM3757_2349_2383 | 0.44 | -1.11 | 1255281\|misL\|putative autotransporter\|+ |
| STM3758_305_339 | 0.04 | 1.45 | 1255282\|fidL\|putative inner membrane protein\|- |
| STM3759.S_705_739 | 0.01 | 2.72 | 1255283\|marT\|putative transcriptional regulator\|- |
| STM3760_65_99 | 0.24 | 1.34 | 1255284\|STM3760\|-\|- |
| STM3761_417_451 | 0.01 | 3.86 | 1255285\|slsA\|putative inner membrane protein\|+ |
| STM3762_193_227 | 0.02 | 1.83 | 1255286\|cigR\|putative inner membrane protein\|- |
| STM3763_2000_2034 | 0.33 | 1.16 | 1255287\|mgtB\|Mg2+ transporter\|- |
| STM3764_577_611 | 0.31 | 1.31 | 1255288\|mgtC\|Mg2+ transport protein\|- |
| STM3765_337_371 | 0.01 | 2.28 | 1255289\|yicL\|putative permease\|+ |
| STM3766_561_595 | 0.20 | -1.20 | 1255290\|STM3766\|putative cytoplasmic protein\|- |
| STM3767_657_691 | 0.07 | -1.09 | 1255291\|STM3767\|putative cytoplasmic protein\|- |
| STM3768_655_689 | 0.35 | 1.18 | 1255292\|STM3768\|putative selenocysteine synthase\|- |
| STM3769.S_321_355 | 0.90 | -1.03 | 1255293\|STM3769.S\|putative phosphotransferase system enzyme II\|- |
| STM3770_641_675 | 0.05 | 1.24 | 1255294\|STM3770\|putative phosphotransferase system enzyme IIC\|- |
| STM3771_81_115 | 0.15 | 1.21 | 1255295\|STM3771\|putative phosphotransferase system enzyme IIB\|- |
| STM3772_385_419 | 0.12 | 1.19 | 1255296\|STM3772\|putative phosphotransferase system enzyme IIA\|- |
| STM3773_2376_2410 | 0.01 | -1.57 | 1255297\|STM3773\|putative transcriptional regulator\|- |
| STM3774_229_263 | 0.15 | 1.32 | 1255298\|STM3774\|putative inner membrane protein\|+ |
| STM3775_640_674 | 0.02 | -1.94 | 1255299\|STM3775\|putative glycosyl hydrolase\|+ |
| STM3776_723_757 | 0.10 | -1.29 | 1255300\|nepI\|ribonucleoside transporter\|- |
| STM3777_273_307 | 0.02 | 2.75 | 1255301\|STM3777\|putative cytoplasmic protein\|+ |
| STM3778_193_227 | 0.01 | 1.61 | 1255302\|STM3778\|putative DNA-binding protein\|+ |
| STM3779_16_50 | 0.28 | 1.36 | 1255303\|STM3779\|putative phosphotransferase system HPr protein\|- |
| STM3780_549_583 | 0.52 | 1.08 | 1255304\|gatY\|putative fructose-1 6-bisphosphate aldolase\|- |
| STM3781_694_728 | 0.11 | 1.36 | 1255305\|STM3781\|putative sugar kinase\|- |
| STM3782_1096_1130 | 0.03 | -1.49 | 1255306\|STM3782\|putative PTS system galactitol-specific enzyme IIC component\|- |
| STM3783_185_219 | 0.04 | 2.15 | 1255307\|STM3783\|putative periplasmic protein\|- |
| STM3784_209_243 | 0.01 | -2.25 | 1255308\|STM3784\|putative phosphotransferase system mannitol/fructose-specific IIA domain\|- |
| STM3785_17_52 | 0.01 | -1.38 | 1255309\|STM3785\|putative regulatory protein\|- |
| STM3786_175_209 | 0.00 | -2.32 | 1255310\|yicN\|putative inner membrane protein\|- |

| | | | |
|---|---|---|---|
| STM3787_593_627 | 0.09 | 1.34 | 1255311\|uhpT\|sugar phosphate antiporter\|- |
| STM3788_854_888 | 0.03 | 1.33 | 1255312\|uhpC\|regulatory protein UhpC\|- |
| STM3789_744_778 | 0.44 | 1.13 | 1255313\|uhpB\|sensory histidine kinase UhpB\|- |
| STM3790_137_171 | 0.78 | -1.05 | 1255314\|uhpA\|DNA-binding response regulator in two-component regulatory system wtih UhpB\|- |
| STM3791_175_209 | 0.04 | -1.93 | 1255315\|STM3791\|putative cytoplasmic protein\|- |
| STM3794_161_195 | 0.48 | 1.07 | 1255318\|STM3794\|putative regulatory protein\|+ |
| STM3795_89_123 | 0.07 | -1.72 | 1255319\|ilvN\|acetolactate synthase 1 regulatory subunit\|- |
| STM3796_962_996 | 0.02 | -1.91 | 1255320\|ilvB\|acetolactate synthase catalytic subunit\|- |
| STM3797_9_43 | 0.52 | -1.42 | 1255321\|ivbL\|ilvB operon leader peptide\|- |
| STM3797A_3_37 | 0.49 | 1.22 | 1255322\|ysdA\|-\|+ |
| STM3796B_20_55 | 0.12 | 1.67 | 1255323\|ysdB\|-\|+ |
| STM3798_378_412 | 0.02 | 1.61 | 1255325\|emrD\|multidrug resistance protein D\|+ |
| STM3799_279_313 | 0.01 | 1.70 | 1255326\|STM3799\|putative periplasmic protein\|- |
| STM3800_17_51 | 0.67 | 1.06 | 1255327\|dsdC\|DNA-binding transcriptional dual regulator\|- |
| STM3801_851_885 | 0.37 | 1.09 | 1255328\|dsdX\|DsdX permease\|+ |
| STM3802_516_550 | 0.04 | 2.22 | 1255329\|dsdA\|D-serine dehydratase\|+ |
| STM3803_97_131 | 0.00 | -4.73 | 1255330\|yidF\|putative cytoplasmic protein\|- |
| STM3804_148_182 | 0.27 | -1.12 | 1255331\|yidG\|putative inner membrane protein\|- |
| STM3805_257_291 | 0.25 | 1.13 | 1255332\|yidH\|putative inner membrane protein\|- |
| STM3806_16_50 | 0.03 | 1.93 | 1255333\|STM3806\|pseudo\|+ |
| STM3807_1575_1609 | 0.18 | 1.16 | 1255334\|yidE\|hypothetical protein\|- |
| STM3808.S_137_171 | 0.18 | 1.46 | 1255335\|ibpB\|heat shock chaperone IbpB\|- |
| STM3809.S_257_291 | 0.04 | 1.90 | 1255336\|ibpA\|heat shock protein IbpA\|- |
| STM3810_121_155 | 0.00 | -2.53 | 1255337\|yidQ\|hypothetical protein\|+ |
| STM3811_804_838 | 0.05 | 1.31 | 1255338\|yidR\|putative cytoplasmic protein\|- |
| STM3812_882_916 | 0.31 | 1.10 | 1255339\|ccmH\|putative heme lyase subunit\|- |
| STM3820_690_724 | 0.05 | 2.68 | 1255347\|STM3820\|putative cytochrome c peroxidase\|- |
| STM3821_383_417 | 0.10 | 1.55 | 1255348\|torD\|chaperone protein TorD\|- |
| STM3822_2066_2100 | 0.33 | -1.20 | 1255349\|torA\|trimethylamine N-oxide reductase subunit\|- |
| STM3823_970_1004 | 0.90 | 1.08 | 1255350\|torC\|trimethylamine N-oxide reductase cytochrome c-like subunit\|- |
| STM3824_569_603 | 0.13 | -1.09 | 1255351\|torR\|DNA-binding response regulator in two-component regulatory system with TorS\|+ |
| STM3825_570_604 | 0.20 | -1.12 | 1255352\|torT\|periplasmic sensory protein associated with the TorRS two-component regulatory system\|- |
| STM3827_1043_1077 | 0.17 | -1.30 | 1255354\|dgoT\|D-galactonate transport protein\|- |

| | | | |
|---|---|---|---|
| STM3828_446_480 | 0.02 | -1.72 | 1255355\|dgoA\|2-oxo-3-deoxygalactonate 6-phosphate aldolase/galactonate dehydratase\|- |
| STM3828.1N_734_768 | 0.04 | -1.33 | 2673742\|STM3828.1N\|-\|- |
| STM3829_113_147 | 0.80 | 1.02 | 1255356\|dgoK\|2-oxo-3-deoxygalactonate kinase\|- |
| STM3830_385_419 | 0.03 | -1.59 | 1255357\|dgoR\|galactonate operon transcriptional repressor\|- |
| STM3831_353_387 | 0.09 | 1.24 | 1255358\|yidA\|predicted hydrolase\|- |
| STM3832_680_714 | 0.01 | 1.38 | 1255359\|STM3832\|putative permease\|+ |
| STM3833_931_965 | 0.07 | 1.36 | 1255360\|STM3833\|putative mandelate racemase\|+ |
| STM3834_485_519 | 0.04 | 2.70 | 1255361\|STM3834\|putative transcriptional regulator\|+ |
| STM3835_2056_2090 | 0.39 | -1.14 | 1255362\|gyrB\|DNA gyrase subunit B\|- |
| STM3837_1062_1096 | 0.01 | -2.23 | 1255364\|dnaN\|DNA polymerase III subunit beta\|- |
| STM3838_658_692 | 0.27 | 1.21 | 1255365\|dnaA\|chromosomal replication initiation protein\|- |
| STM3839_13_47 | 0.01 | -3.04 | 1255366\|rpmH\|50S ribosomal protein L34\|+ |
| STM3840_177_211 | 0.05 | -1.58 | 1255367\|rnpA\|ribonuclease P\|+ |
| STM3842_1128_1162 | 0.00 | -2.21 | 1255369\|yidC\|putative inner membrane protein translocase component YidC\|+ |
| STM3843_1006_1040 | 0.04 | 1.12 | 1255370\|trmE\|tRNA modification GTPase TrmE\|+ |
| STM3844_478_512 | 0.31 | 1.14 | 1255371\|STM3844\|pseudo\|+ |
| STM3845_369_403 | 0.01 | 2.33 | 1255372\|STM3845\|putative inner membrane protein\|+ |
| STM3847_477_511 | 0.00 | 1.38 | 1255374\|yidY\|multidrug efflux system protein MdtL\|+ |
| STM3848_521_555 | 0.09 | 1.23 | 1255375\|yidZ\|DNA-binding transcriptional regulator YidZ\|+ |
| STM3849_561_595 | 0.43 | 1.23 | 1255376\|yieE\|putative cytoplasmic protein\|+ |
| STM3850_489_523 | 0.13 | -1.23 | 1255377\|yieF\|putative oxidoreductase\|+ |
| STM3851.S_1147_1181 | 0.05 | -1.27 | 1255378\|yieG\|hypothetical protein\|- |
| STM3852_97_131 | 0.00 | -2.16 | 1255379\|yieH\|predicted hydrolase\|+ |
| STM3853_53_87 | 0.05 | -1.48 | 1255380\|phoU\|transcriptional regulator PhoU\|- |
| STM3854_673_707 | 0.02 | -1.51 | 1255381\|pstB\|phosphate transporter subunit\|- |
| STM3855_73_107 | 0.11 | -1.27 | 1255382\|pstA\|phosphate transporter permease subunit\|- |
| STM3856_897_931 | 0.03 | -1.39 | 1255383\|pstC\|phosphate transporter permease subunit\|- |
| STM3857_634_668 | 0.11 | -1.34 | 1255384\|pstS\|phosphate transporter subunit\|- |
| STM3858_923_957 | 0.63 | 1.02 | 1255385\|STM3858\|putative phosphotransferase system fructose-specific component IIB\|- |
| STM3859_481_515 | 0.48 | 1.12 | 1255386\|aroE\|shikimate 5-dehydrogenase\|- |
| STM3860_999_1033 | 0.11 | 1.25 | 1255387\|STM3860\|putative dipeptide/oligopeptide/nickel ABC-type transport system periplasmic component\|+ |
| STM3861_895_929 | 0.14 | -1.11 | 1255388\|glmS\|D-fructose-6-phosphate amidotransferase\|- |

| | | | |
|---|---|---|---|
| STM3862_1268_1302 | 0.02 | 1.69 | 1255389\|glmU\|bifunctional N-acetylglucosamine-1-phosphate uridyltransferase/glucosamine-1-phosphate acetyltransferase\|- |
| STM3863_177_211 | 0.11 | 1.29 | 1255390\|STM3863\|putative permease\|- |
| STM3864_129_163 | 0.11 | -1.31 | 1255391\|atpC\|F0F1 ATP synthase subunit epsilon\|- |
| STM3865_480_514 | 0.22 | -1.33 | 1255392\|atpD\|F0F1 ATP synthase subunit beta\|- |
| STM3866_641_675 | 0.01 | -1.83 | 1255393\|atpG\|F0F1 ATP synthase subunit gamma\|- |
| STM3867_1343_1377 | 0.20 | -1.34 | 1255394\|atpA\|F0F1 ATP synthase subunit alpha\|- |
| STM3868_321_355 | 0.07 | -1.27 | 1255395\|atpH\|F0F1 ATP synthase subunit delta\|- |
| STM3869_57_91 | 0.51 | 1.07 | 1255396\|atpF\|F0F1 ATP synthase subunit B\|- |
| STM3870_177_211 | 0.07 | -1.38 | 1255397\|atpE\|F0F1 ATP synthase subunit C\|- |
| STM3871_769_803 | 0.01 | -1.68 | 1255398\|atpB\|F0F1 ATP synthase subunit A\|- |
| STM3872_9_43 | 0.04 | -1.43 | 1255399\|atpI\|F0F1 ATP synthase subunit I\|- |
| STM3873.S_65_99 | 0.01 | -1.68 | 1255400\|gidB\|glucose-inhibited division protein B\|- |
| STM3874_1275_1309 | 0.02 | -1.73 | 1255401\|gidA\|tRNA uridine 5-carboxymethylaminomethyl modification enzyme GidA\|- |
| STM3875_105_139 | 0.01 | -2.21 | 1255402\|mioC\|flavodoxin\|- |
| STM3876_337_371 | 0.04 | 1.41 | 1255403\|asnC\|DNA-binding transcriptional regulator AsnC\|- |
| STM3877_745_779 | 0.02 | 1.73 | 1255404\|asnA\|asparagine synthetase AsnA\|+ |
| STM3878.S_1381_1415 | 0.13 | -1.22 | 1255405\|yieM\|hypothetical protein\|- |
| STM3879_538_572 | 0.01 | 1.98 | 1255406\|yieN\|putative regulatory protein\|- |
| STM3880_1510_1544 | 0.01 | -1.51 | 1255407\|trkD\|potassium transport protein Kup\|+ |
| STM3881_225_259 | 0.98 | 1.01 | 1255408\|rbsD\|high-affinity D-ribose transport protein\|+ |
| STM3882_763_797 | 0.00 | -2.10 | 1255409\|rbsA\|D-ribose transporter ATP binding protein\|+ |
| STM3883_561_595 | 0.02 | -2.11 | 1255410\|rbsC\|ribose ABC transporter permease protein\|+ |
| STM3884_361_395 | 0.04 | -1.47 | 1255411\|rbsB\|D-ribose transporter subunit RbsB\|+ |
| STM3885_193_227 | 0.01 | -3.96 | 1255412\|rbsK\|ribokinase\|+ |
| STM3886_161_195 | 0.01 | -1.62 | 1255413\|rbsR\|transcriptional repressor RbsR\|+ |
| STM3887_1197_1231 | 0.41 | -1.09 | 1255414\|yieO\|putative tranport protein\|- |
| STM3888_481_515 | 0.20 | 1.13 | 1255415\|yieP\|putative regulatory protein\|- |
| STM3893_11_48 | 0.02 | -2.09 | 1255419\|9S\|misc_RNA\|+ |
| STM3896_6_40 | 0.02 | -2.23 | 1255422\|trpT\|tRNA\|+ |
| STM3897_97_131 | 0.01 | 1.18 | 1255423\|yifA\|transcriptional regulator HdfR\|- |
| STM3898_97_131 | 0.01 | -2.29 | 1255424\|yifE\|hypothetical protein\|+ |
| STM3899_522_556 | 0.03 | 1.36 | 1255425\|yifB\|putative ATP-dependent protease\|- |
| STM3900_9_43 | 0.88 | -1.10 | 1255426\|ilvL\|ilvG operon leader peptide\|+ |
| STM3901_776_810 | 0.00 | -13.21 | 1255427\|ilvG\|acetolactate synthase 2 catalytic subunit\|+ |

| | | | |
|---|---|---|---|
| STM3902_9_43 | 0.00 | -11.02 | 1255428\|ilvM\|acetolactate synthase 2 regulatory subunit\|+ |
| STM3903_833_867 | 0.00 | -9.87 | 1255429\|ilvE\|branched-chain amino acid aminotransferase\|+ |
| STM3904_1236_1270 | 0.00 | -13.51 | 1255430\|ilvD\|dihydroxy-acid dehydratase\|+ |
| STM3905_1202_1236 | 0.00 | -13.42 | 1255431\|ilvA\|threonine dehydratase\|+ |
| STM3906_33_67 | 0.31 | -1.18 | 1255432\|STM3906\|putative cytoplasmic protein\|+ |
| STM3907_13_47 | 0.03 | -1.33 | 1255433\|STM3907\|putative cytoplasmic protein\|+ |
| STM3908_609_643 | 0.03 | 2.52 | 1255434\|ilvY\|DNA-binding transcriptional regulator IlvY\|- |
| STM3910_165_199 | 0.13 | 1.22 | 1255436\|ppiC\|peptidyl-prolyl cis-trans isomerase C\|- |
| STM3911_89_123 | 0.01 | 1.51 | 1255437\|STM3911\|putative inner membrane protein\|+ |
| STM3912_1330_1364 | 0.03 | -1.55 | 1255438\|rep\|ATP-dependent DNA helicase Rep\|+ |
| STM3913_1059_1093 | 0.26 | 1.21 | 1255439\|gppA\|guanosine pentaphosphate phosphohydrolase\|- |
| STM3914_1099_1133 | 0.03 | -1.22 | 1255440\|rhlB\|ATP-dependent RNA helicase RhlB\|- |
| STM3915_205_239 | 0.63 | 1.09 | 1255441\|trxA\|thioredoxin\|+ |
| STM3916_3_37 | 0.12 | 1.61 | 1255442\|rhoL\|pseudo\|+ |
| STM3917_421_455 | 0.92 | 1.02 | 1255443\|rho\|transcription termination factor Rho\|+ |
| STM3918_649_683 | 0.96 | 1.00 | 1255444\|rfe\|undecaprenyl-phosphate N-acetylglucosaminyltransferase\|+ |
| STM3919_368_402 | 0.05 | -1.26 | 1255445\|wzzE\|lipopolysaccharide biosynthesis protein WzzE\|+ |
| STM3920_708_742 | 0.10 | 1.20 | 1255446\|wecB\|UDP-N-acetyl glucosamine-2-epimerase\|+ |
| STM3921_984_1018 | 0.09 | 1.23 | 1255447\|wecC\|UDP-N-acetyl-D-mannosamine dehydrogenase\|+ |
| STM3922_389_423 | 0.23 | 1.09 | 1255448\|rffG\|dTDP-glucose 4 6-dehydratase\|+ |
| STM3923_177_211 | 0.01 | 1.61 | 1255449\|rffH\|glucose-1-phosphate thymidyl transferase\|+ |
| STM3924_473_507 | 0.34 | 1.42 | 1255450\|wecD\|TDP-fucosamine acetyltransferase\|+ |
| STM3925_420_454 | 0.09 | 1.18 | 1255451\|wecE\|TDP-4-oxo-6-deoxy-D-glucose transaminase\|+ |
| STM3926_556_590 | 0.60 | 1.10 | 1255452\|wzxE\|O-antigen translocase\|+ |
| STM3927_929_963 | 0.28 | 1.08 | 1255453\|STM3927\|4-alpha-L-fucosyltransferase\|+ |
| STM3928_1288_1322 | 0.23 | 1.35 | 1255454\|wecF\|putative enterobacterial common antigen polymerase\|+ |
| STM3929_65_99 | 0.02 | 1.41 | 1255455\|wecG\|putative UDP-N-acetyl-D-mannosaminuronic acid transferase\|+ |
| STM3930_1203_1237 | 0.27 | -1.28 | 1255456\|yifK\|putative transport protein YifK\|+ |
| STM3931_33_67 | 0.85 | 1.16 | 1255457\|argX\|tRNA\|+ |
| STM3935_841_875 | 0.19 | -1.21 | 1255461\|hemY\|putative protoheme IX biogenesis protein\|- |
| STM3936_659_693 | 0.68 | -1.05 | 1255462\|hemX\|putative uroporphyrinogen III C-methyltransferase\|- |
| STM3937_321_355 | 0.01 | -1.44 | 1255463\|hemD\|uroporphyrinogen-III synthase\|- |
| STM3938_241_275 | 0.03 | -1.58 | 1255464\|hemC\|porphobilinogen deaminase\|- |
| STM3939_1964_1998 | 0.00 | 3.19 | 1255465\|cyaA\|adenylate cyclase\|+ |
| STM3940_313_347 | 0.60 | 1.05 | 1255466\|STM3940\|putative inner membrane protein\|+ |
| STM3941_297_331 | 0.96 | -1.00 | 1255467\|STM3941\|putative inner membrane protein\|+ |

| | | | |
|---|---|---|---|
| STM3942_315_349 | 0.76 | 1.01 | 1255468lSTM3942lputative cytoplasmic proteinl+ |
| STM3943_141_175 | 0.07 | 1.53 | 1255469lcyaYlfrataxin-like proteinl- |
| STM3944_305_339 | 0.29 | 1.39 | 1255470lSTM3944lputative inner membrane proteinl- |
| STM3945_145_179 | 0.04 | 1.70 | 1255471lSTM3945lpseudol- |
| STM3946_9_45 | 0.02 | 1.69 | 1255472lyifLlputative outer membrane lipoproteinl+ |
| STM3947_769_803 | 0.48 | -1.08 | 1255473ldapFldiaminopimelate epimerasel+ |
| STM3948_409_443 | 0.86 | -1.02 | 1255474lyigAlhypothetical proteinl+ |
| STM3949_97_131 | 0.02 | 1.26 | 1255475lxerClsite-specific tyrosine recombinase XerCl+ |
| STM3950_65_99 | 0.02 | -1.28 | 1255476lyigBlpredicted hydrolasel+ |
| STM3951_1836_1870 | 0.06 | 1.43 | 1255477luvrDlDNA-dependent helicase IIl+ |
| STM3952_273_307 | 0.51 | 1.09 | 1255478lcorAlmagnesium/nickel/cobalt transporter CorAl+ |
| STM3953_273_307 | 0.01 | 1.79 | 1255479lyigFlputative inner membrane proteinl- |
| STM3955_577_611 | 0.14 | 1.14 | 1255481lrarDlchloramphenicol resistancel- |
| STM3956_49_83 | 0.00 | -3.44 | 1255482lyigIlhypothetical proteinl- |
| STM3957_769_803 | 0.00 | -1.68 | 1255483lpldAlphospholipase Al+ |
| STM3958_1361_1395 | 0.08 | -1.29 | 1255484lrecQlATP-dependent DNA helicase RecQl+ |
| STM3959_533_567 | 0.03 | -1.41 | 1255485lrhtClthreonine efflux systeml+ |
| STM3960_65_99 | 0.08 | -1.27 | 1255486lrhtBlhomoserine/homoserine lactone efflux proteinl- |
| STM3961_482_516 | 0.13 | 1.16 | 1255487lpldBllysophospholipase L2l+ |
| STM3962_129_163 | 0.07 | 1.08 | 1255488lyigLlpredicted hydrolasel+ |
| STM3963_273_307 | 0.22 | 1.21 | 1255489lyigMlputative transport proteinl+ |
| STM3964_529_563 | 0.01 | 11.23 | 1255490lmetRlmetE/metH regulatorl- |
| STM3965_1930_1964 | 0.26 | 1.81 | 1255491lmetEl5-methyltetrahydropteroyltriglutamate--homocysteine methyltransferasel+ |
| STM3966_521_555 | 0.40 | -1.11 | 1255492lSTM3966lputative arylsulfatase regulatorl+ |
| STM3967_705_739 | 0.03 | -1.33 | 1255493ldlhHlputative dienelactone hydrolasel- |
| STM3968_481_515 | 1.00 | 1.00 | 1255494ludpluridine phosphorylasel+ |
| STM3969_1152_1186 | 0.04 | 1.86 | 1255495lyigNlDNA recombination protein RmuCl+ |
| STM3970_321_355 | 0.30 | 1.17 | 1255496lubiElubiquinone/menaquinone biosynthesis methyltransferasel+ |
| STM3971_193_227 | 0.10 | -1.23 | 1255497lyigPlputative inner membrane proteinl+ |
| STM3972_1122_1156 | 0.10 | 1.33 | 1255498lubiBlputative ubiquinone biosynthesis protein UbiBl+ |
| STM3973_29_63 | 0.09 | 1.19 | 1255499ltatAltwin argininte translocase protein Al+ |
| STM3974_433_467 | 0.03 | 1.49 | 1255500ltatBlsec-independent translocasel+ |
| STM3975_297_331 | 0.22 | 1.20 | 1255501ltatClTatABCE protein translocation system subunitl+ |
| STM3977_289_323 | 0.28 | 1.18 | 1255503lrfaHltranscriptional activator RfaHl- |
| STM3978_1232_1266 | 0.00 | -1.44 | 1255504lyigCl3-octaprenyl-4-hydroxybenzoate decarboxylasel+ |

| | | | |
|---|---|---|---|
| STM3979_529_563 | 0.04 | -1.38 | 1255505\|fre\|FMN reductase\|+ |
| STM3980_193_227 | 0.01 | 2.73 | 1255506\|STM3980\|putative outer membrane protein\|+ |
| STM3981_60_94 | 0.03 | 2.02 | 1255507\|STM3981\|putative cytoplasmic protein\|+ |
| STM3982_287_321 | 0.00 | -12.42 | 1255508\|fadA\|3-ketoacyl-CoA thiolase\|- |
| STM3983_1447_1481 | 0.03 | -2.05 | 1255509\|fadB\|multifunctional fatty acid oxidation complex subunit alpha\|- |
| STM3984_1005_1039 | 0.03 | -1.48 | 1255510\|pepQ\|proline dipeptidase\|+ |
| STM3985_289_323 | 0.02 | -1.77 | 1255511\|yigZ\|hypothetical protein\|+ |
| STM3986_869_903 | 0.19 | -1.40 | 1255512\|trkH\|potassium transporter\|+ |
| STM3987_153_187 | 0.06 | -1.21 | 1255513\|hemG\|protoporphyrinogen oxidase\|+ |
| STM3989/STM4133_4_38 | 0.03 | -1.58 | 1255515/1255659\|ileT/ileU\|tRNA\|+ |
| STM3993_417_451 | 0.04 | 2.14 | 1255519\|mobB\|molybdopterin-guanine dinucleotide biosynthesis protein B\|- |
| STM3994_129_163 | 0.04 | 1.33 | 1255520\|mobA\|molybdopterin-guanine dinucleotide biosynthesis protein A\|- |
| STM3995_97_131 | 0.94 | 1.02 | 1255521\|yihD\|putative cytoplasmic protein\|+ |
| STM3996_641_675 | 0.04 | 1.59 | 1255522\|yihE\|predicted kinase\|+ |
| STM3997_353_387 | 0.01 | 2.42 | 1255523\|dsbA\|periplasmic protein disulfide isomerase I\|+ |
| STM3998_193_227 | 0.08 | 2.88 | 1255524\|yihG\|putative endonuclease\|- |
| STM3999_2300_2334 | 0.29 | -1.06 | 1255525\|polA\|DNA polymerase I\|+ |
| STM4000_27_65 | 0.05 | 4.19 | 2673732\|spf\|misc_RNA\|+ |
| STM4001_465_499 | 0.12 | 1.19 | 1255527\|yihA\|GTPase EngB\|- |
| STM4002_48_82 | 0.80 | -1.09 | 1255528\|STM4002\|putative cytoplasmic protein\|+ |
| STM4003_289_323 | 0.06 | -1.63 | 1255529\|yihI\|hypothetical protein\|+ |
| STM4004_1079_1113 | 0.19 | 1.19 | 1255530\|hemN\|coproporphyrinogen III oxidase\|+ |
| STM4005_475_509 | 0.29 | -1.10 | 1255531\|glnG\|nitrogen regulation protein NR(I)\|- |
| STM4006_307_341 | 0.02 | 2.68 | 1255532\|glnL\|nitrogen regulation protein NR(II)\|- |
| STM4007_475_509 | 0.04 | 1.85 | 1255533\|glnA\|glutamine synthetase\|- |
| STM4009_1145_1179 | 0.94 | 1.04 | 1255535\|typA\|GTP-binding protein\|+ |
| STM4010_129_163 | 0.17 | 1.42 | 1255536\|STM4010\|putative hydrolase\|- |
| STM4011_833_867 | 0.08 | 1.66 | 1255537\|STM4011\|putative inner membrane protein\|- |
| STM4013.S_737_771 | 0.24 | 1.37 | 1255539\|STM4013.S\|putative membrane-associated metal-dependent hydrolase\|- |
| STM4014_289_323 | 0.63 | -1.06 | 1255540\|STM4014\|putative periplasmic protein\|- |
| STM4015_625_659 | 0.01 | 1.52 | 1255541\|STM4015\|putative cytoplasmic protein\|- |
| STM4016_307_341 | 1.00 | -1.00 | 1255542\|yshA\|predicted outer membrane porin L\|- |
| STM4017_1095_1129 | 0.10 | 1.59 | 1255543\|yihO\|putative GPH family transport protein\|- |
| STM4018_1120_1154 | 0.01 | 1.63 | 1255544\|yihP\|putative GPH family transport protein\|- |
| STM4020.S_353_387 | 0.11 | 1.19 | 1255546\|yihR\|putative aldose-1-epimerase\|- |

| | | | |
|---|---|---|---|
| STM4021_979_1013 | 0.91 | -1.09 | 1255547\|yihS\|putative isomerase\|- |
| STM4022_801_835 | 0.71 | -1.03 | 1255548\|yihT\|putative aldolase\|- |
| STM4023_257_291 | 0.16 | -1.29 | 1255549\|yihU\|putative oxidoreductase\|- |
| STM4024.S_33_67 | 0.37 | -1.16 | 1255550\|yihV\|putative sugar kinase\|+ |
| STM4025_33_67 | 0.02 | -1.57 | 1255551\|yihW\|putative glycerol-3-phosphate regulon repressor\|+ |
| STM4026_257_291 | 0.01 | 1.72 | 1255552\|yihX\|phosphatase\|+ |
| STM4027_593_627 | 0.18 | 1.14 | 1255553\|rbn\|ribonuclease BN\|+ |
| STM4028_2_36 | 0.10 | 1.72 | 1255554\|yihZ\|D-tyrosyl-tRNA deacylase\|+ |
| STM4029_929_963 | 0.41 | -1.10 | 1255555\|yiiD\|putative acetyltransferase\|+ |
| STM4030.S_253_287 | 0.03 | 2.25 | 1255556\|STM4030.S\|putative cytoplasmic protein\|- |
| STM4031_257_291 | 0.00 | 2.32 | 1255557\|STM4031\|putative cytoplasmic protein\|- |
| STM4032_257_291 | 0.07 | 1.14 | 1255558\|STM4032\|putative acetyl esterase\|- |
| STM4032.2N_97_131 | 0.48 | 1.21 | 2673731\|STM4032.2N\|hypothetical protein\|+ |
| STM4033_161_195 | 0.23 | -1.21 | 1255559\|STM4033\|putative regulatory protein\|+ |
| STM4034_321_355 | 0.35 | 1.11 | 1255560\|fdhE\|formate dehydrogenase accessory protein FdhE\|- |
| STM4035_421_455 | 0.87 | -1.03 | 1255561\|fdoI\|formate dehydrogenase-O subunit gamma\|- |
| STM4036_681_715 | 0.04 | -1.25 | 1255562\|fdoH\|formate dehydrogenase-O beta subunit\|- |
| STM4037_2884_2918 | 0.07 | 1.86 | 1255563\|fdoG\|formate dehydrogenase alpha subunit\|- |
| STM4038_425_459 | 0.01 | 1.92 | 1255564\|fdhD\|formate dehydrogenase accessory protein\|+ |
| STM4039_929_963 | 0.33 | 1.26 | 1255565\|STM4039\|putative inner membrane lipoprotein\|- |
| STM4040_548_582 | 0.45 | 1.06 | 1255566\|yiiG\|putative cytoplasmic protein\|+ |
| STM4041_249_283 | 0.05 | 1.54 | 1255567\|STM4041\|putative inner membrane protein\|- |
| STM4042_493_527 | 0.02 | 1.95 | 1255568\|STM4042\|putative branched-chain amino acid permease\|- |
| STM4042A_105_139 | 0.00 | 2.93 | 1255569\|STM4042A\|hypothetical protein\|+ |
| STM4043_49_83 | 0.01 | -2.60 | 1255570\|yiiL\|putative cytoplasmic protein\|- |
| STM4044_438_472 | 0.00 | -5.55 | 1255571\|STM4044\|putative alcohol dehydrogenase\|- |
| STM4046_709_743 | 0.53 | 1.19 | 1255573\|rhaA\|L-rhamnose isomerase\|- |
| STM4047_983_1017 | 0.70 | 1.08 | 1255574\|rhaB\|rhamnulokinase\|- |
| STM4048_449_483 | 0.24 | -1.06 | 1255575\|rhaS\|L-rhamnose operon regulatory protein\|+ |
| STM4049_289_323 | 0.01 | -2.58 | 1255576\|rhaR\|rhaRS operon regulatory protein\|+ |
| STM4050_740_774 | 0.80 | -1.12 | 1255577\|rhaT\|L-rhamnose/H+ symporter protein\|- |
| STM4052_741_775 | 0.02 | -1.17 | 1255579\|STM4052\|putative C4-dicarboxylate transport system\|- |
| STM4053_209_243 | 0.01 | 1.49 | 1255580\|STM4053\|putative C4-dicarboxylate transport system\|- |
| STM4054_321_355 | 0.51 | 1.25 | 1255581\|STM4054\|putative periplasmic dicarboxylate-binding protein\|- |
| STM4055_49_83 | 0.11 | -1.13 | 1255582\|sodA\|superoxide dismutase\|+ |
| STM4056.S_545_579 | 0.03 | -1.83 | 1255583\|yiiM\|hypothetical protein\|+ |

| | | | |
|---|---|---|---|
| STM4057_327_361 | 0.02 | -1.06 | 1255584\|STM4057\|putative inner membrane protein\|+ |
| STM4058_1079_1113 | 0.25 | 1.15 | 1255585\|cpxA\|two-component sensor protein\|- |
| STM4059_399_433 | 0.12 | 1.55 | 1255586\|cpxR\|DNA-binding response regulator in two-component regulatory system with CpxA\|- |
| STM4060_137_171 | 0.00 | 13.88 | 1255587\|cpxP\|periplasmic repressor CpxP\|+ |
| STM4061_385_419 | 0.04 | 1.28 | 1255588\|fieF\|ferrous iron efflux protein F\|+ |
| STM4062_129_163 | 0.06 | 1.37 | 1255589\|pfkA\|6-phosphofructokinase\|+ |
| STM4063_769_803 | 0.76 | 1.29 | 1255590\|sbp\|sulfate transporter subunit\|+ |
| STM4064_161_195 | 0.13 | 1.60 | 1255591\|ushB\|CDP-diacylglycerol pyrophosphatase\|+ |
| STM4066_625_659 | 0.14 | -1.62 | 1255593\|STM4066\|aminoimidazole riboside kinase\|+ |
| STM4067_778_812 | 0.14 | -1.12 | 1255594\|STM4067\|putative ADP-ribosylglycohydrolase\|+ |
| STM4068_161_195 | 0.14 | 1.30 | 1255595\|STM4068\|putative regulatory protein\|+ |
| STM4069_33_67 | 0.13 | 1.42 | 1255596\|STM4069\|putative periplasmic protein\|+ |
| STM4070_53_87 | 0.75 | -1.05 | 1255597\|STM4070\|putative cytoplasmic protein\|+ |
| STM4071_25_59 | 0.05 | -12.37 | 1255598\|STM4071\|putative mannose-6-phosphate isomerase\|- |
| STM4072_1330_1364 | 0.15 | -4.36 | 1255599\|ydeV\|autoinducer-2 (AI-2) kinase\|- |
| STM4074_1257_1291 | 0.06 | -8.53 | 1255601\|ego\|putative ABC-type aldose transport system ATPase component\|+ |
| STM4075_333_367 | 0.06 | -9.36 | 1255602\|ydeY\|putative sugar transport protein\|+ |
| STM4076_139_173 | 0.15 | -4.14 | 1255603\|ydeZ\|putative sugar transport protein\|+ |
| STM4078_289_323 | 0.02 | -22.30 | 1255605\|yneB\|aldolase\|+ |
| STM4079.S_97_131 | 0.19 | -3.71 | 1255606\|yneC\|autoinducer-2 (AI-2) modifying protein LsrG\|+ |
| STM4080_673_707 | 0.03 | -11.48 | 1255607\|STM4080\|ribulose-phosphate 3-epimerase\|+ |
| STM4081_193_227 | 0.10 | 1.28 | 1255608\|tpiA\|triosephosphate isomerase\|- |
| STM4082_457_491 | 0.40 | -1.15 | 1255609\|yiiQ\|putative periplasmic protein\|- |
| STM4083_333_367 | 0.36 | 1.12 | 1255610\|yiiR\|putative inner membrane protein\|+ |
| STM4084_37_71 | 0.01 | 1.73 | 1255611\|fpr\|ferredoxin-NADP reductase\|- |
| STM4085_940_974 | 0.04 | -2.05 | 1255612\|glpX\|fructose 1 6-bisphosphatase II\|- |
| STM4086_678_712 | 0.00 | -51.35 | 1255613\|glpK\|glycerol kinase\|- |
| STM4087_545_579 | 0.00 | -32.58 | 1255614\|glpF\|glycerol diffusion\|- |
| STM4088_199_233 | 0.07 | -1.33 | 1255615\|yiiU\|putative cytoplasmic protein\|+ |
| STM4089_441_475 | 0.00 | 3.21 | 1255616\|menG\|ribonuclease activity regulator protein RraA\|- |
| STM4090_289_323 | 0.26 | 1.15 | 1255617\|menA\|1 4-dihydroxy-2-naphthoate octaprenyltransferase\|- |
| STM4091_365_399 | 0.08 | 1.38 | 1255618\|hslU\|ATP-dependent protease ATP-binding subunit\|- |
| STM4092_233_267 | 0.05 | 1.77 | 1255619\|hslV\|ATP-dependent protease peptidase subunit\|- |
| STM4093_897_931 | 0.02 | 1.37 | 1255620\|ftsN\|essential cell division protein\|- |
| STM4094_603_637 | 0.92 | 1.02 | 1255621\|cytR\|DNA-binding transcriptional regulator CytR\|- |

| | | | |
|---|---|---|---|
| STM4095_1912_1946 | 0.70 | -1.06 | 1255622lpriAlprimosome assembly protein PriAl- |
| STM4096_127_161 | 0.17 | 1.33 | 1255623lrpmEl50S ribosomal protein L31l+ |
| STM4097_321_355 | 0.08 | 1.79 | 1255624lSTM4097lputative outer membrane lipoproteinl- |
| STM4098_1144_1178 | 0.07 | 1.92 | 1255625lSTM4098lputative arylsulfate sulfotransferasel- |
| STM4099_205_239 | 0.01 | 3.02 | 1255626lmetJltranscriptional repressor protein MetJl- |
| STM4100_290_324 | 0.02 | 10.64 | 1255627lmetBlcystathionine gamma-synthasel+ |
| STM4101_1658_1692 | 0.02 | 4.25 | 1255628lmetLlbifunctional aspartate kinase II/homoserine dehydrogenase IIl+ |
| STM4102_245_279 | 0.01 | 2.09 | 1255629lSTM4102lputative inner membrane proteinl- |
| STM4103_926_960 | 0.01 | -2.88 | 1255630lSTM4103lputative cytoplasmic proteinl- |
| STM4104_1198_1232 | 0.18 | 1.28 | 1255631lSTM4104lputative 5'-nucleotidase/2' 3'-cyclic phosphodiesterasel+ |
| STM4105_385_419 | 0.01 | 6.11 | 1255632lmetFl5 10-methylenetetrahydrofolate reductasel+ |
| STM4106_1726_1760 | 0.01 | -3.63 | 1255633lkatGlhydroperoxidasel+ |
| STM4107_65_99 | 0.16 | 1.65 | 1255634lyijFlputative periplasmic proteinl- |
| STM4108_605_639 | 0.98 | -1.01 | 1255635lgldAlglycerol dehydrogenasel- |
| STM4109_577_611 | 0.01 | 1.69 | 1255636ltalClfructose-6-phosphate aldolasel- |
| STM4110_1631_1665 | 0.01 | -2.13 | 1255637lptsAlPEP-protein phosphotransferasel- |
| STM4112_363_397 | 0.03 | 1.24 | 1255639lfrwClPTS system fructose-like IIC componentl+ |
| STM4113_193_227 | 0.12 | -1.32 | 1255640lfrwBlPTS system fructose-like IIB component 1l+ |
| STM4114_2003_2037 | 0.07 | 1.27 | 1255641lpflDlpredicted formate acetyltransferase 2 (pyruvate formate lyase II)l+ |
| STM4115_161_195 | 0.12 | 1.44 | 1255642lpflClpyruvate formate lyase II activasel+ |
| STM4116_177_211 | 0.09 | 1.20 | 1255643lfrwDlPTS system fructose-like IIB component 2l+ |
| STM4117_417_451 | 0.40 | -1.10 | 1255644lyijOlputative regulatory proteinl- |
| STM4119_2517_2551 | 0.42 | 1.23 | 1255646lppclphosphoenolpyruvate carboxylasel- |
| STM4120_793_827 | 0.01 | 2.49 | 1255647largElacetylornithine deacetylasel- |
| STM4122_321_355 | 0.46 | 1.14 | 1255649largBlacetylglutamate kinasel+ |
| STM4123_1154_1188 | 0.04 | 1.54 | 1255650largHlargininosuccinate lyasel+ |
| STM4125_321_355 | 0.11 | 1.35 | 1255651loxyRlDNA-binding transcriptional regulator OxyRl+ |
| STM4126_1330_1364 | 0.00 | -2.04 | 1255652ludhAlsoluble pyridine nucleotide transhydrogenasel- |
| STM4127_401_435 | 0.02 | 1.42 | 1255653lyijClDNA-binding transcriptional repressor FabRl+ |
| STM4128_97_131 | 0.20 | 1.29 | 1255654lyijDlhypothetical proteinl+ |
| STM4129_102_136 | 0.04 | -1.26 | 1255655ltrmAltRNA (uracil-5-)-methyltransferasel- |
| STM4130_1486_1520 | 0.02 | -1.76 | 1255656lbtuBlvitamin B12/cobalamin outer membrane transporterl+ |
| STM4131_449_483 | 0.01 | 1.58 | 1255657lmurIlglutamate racemasel+ |
| STM4137_862_896 | 0.02 | 1.70 | 1255663lmurBlUDP-N-acetylenolpyruvoylglucosamine reductasel+ |
| STM4138_513_547 | 0.05 | 1.14 | 1255664lbirAlbiotin--protein ligasel+ |

| | | | |
|---|---|---|---|
| STM4139_609_643 | 0.83 | -1.05 | 1255665\|coaA\|pantothenate kinase\|- |
| STM4140_65_100 | 0.65 | 1.20 | 1255666\|STM4140\|pseudo\|- |
| STM4141_117_151 | 0.59 | -1.04 | 1255667\|STM4141\|putative cytoplasmic protein\|- |
| STM4146_980_1014 | 0.01 | -2.78 | 1255672\|tufB\|elongation factor Tu\|+ |
| STM4147_225_259 | 0.06 | -1.25 | 1255673\|secE\|preprotein translocase subunit SecE\|+ |
| STM4148_193_227 | 0.01 | -1.76 | 1255674\|nusG\|transcription antitermination protein NusG\|+ |
| STM4149_385_419 | 0.01 | -3.39 | 1255675\|rplK\|50S ribosomal protein L11\|+ |
| STM4150_137_171 | 0.01 | -4.70 | 1255676\|rplA\|50S ribosomal protein L1\|+ |
| STM4151_225_259 | 0.01 | -2.25 | 1255677\|rplJ\|50S ribosomal protein L10\|+ |
| STM4153_3350_3384 | 0.05 | -2.26 | 1255679\|rpoB\|DNA-directed RNA polymerase subunit beta\|+ |
| STM4154_3225_3259 | 0.01 | -3.86 | 1255680\|rpoC\|DNA-directed RNA polymerase subunit beta'\|+ |
| STM4155_3_37 | 0.01 | -1.89 | 1255681\|STM4155\|putative inner membrane protein\|+ |
| STM4156_127_164 | 0.01 | 2.38 | 1255682\|STM4156\|putative cytoplasmic protein\|- |
| STM4157_780_814 | 0.89 | -1.02 | 1255683\|STM4157\|putative cytoplasmic protein\|+ |
| STM4157.1N_55_89 | 0.28 | 1.96 | 2673736\|STM4157.1N\|-\|- |
| STM4158_93_127 | 0.01 | 3.28 | 1255684\|STM4158\|putative cytoplasmic protein\|+ |
| STM4159_647_681 | 0.15 | -1.64 | 1255685\|thiH\|thiamine biosynthesis protein ThiH\|- |
| STM4160_122_156 | 0.05 | -2.12 | 1255686\|thiG\|thiazole synthase\|- |
| STM4162_353_387 | 0.06 | -1.80 | 1255688\|thiF\|thiamine biosynthesis protein ThiF\|- |
| STM4163_193_227 | 0.04 | -2.35 | 1255689\|thiE\|thiamine-phosphate pyrophosphorylase\|- |
| STM4164_1313_1347 | 0.98 | 1.01 | 1255690\|thiC\|thiamine biosynthesis protein ThiC\|- |
| STM4165_81_115 | 0.06 | -1.48 | 1255691\|rsd\|anti-RNA polymerase sigma 70 factor\|- |
| STM4166_737_771 | 0.02 | 1.69 | 1255692\|nudC\|NADH pyrophosphatase\|+ |
| STM4167_402_436 | 0.03 | 1.32 | 1255693\|hemE\|uroporphyrinogen decarboxylase\|+ |
| STM4168_161_195 | 0.02 | 1.23 | 1255694\|nfi\|endonuclease V\|+ |
| STM4169_481_515 | 0.10 | 1.52 | 1255695\|yjaG\|putative cytoplasmic protein\|+ |
| STM4170_113_147 | 0.01 | -1.80 | 1255696\|hupA\|transcriptional regulator HU subunit alpha\|+ |
| STM4171_577_611 | 0.13 | -1.32 | 1255697\|yjaH\|putative inner membrane protein\|+ |
| STM4172_305_339 | 0.09 | 1.45 | 1255698\|zraP\|zinc resistance protein\|- |
| STM4173_895_929 | 0.45 | -1.20 | 1255699\|hydH\|sensor protein ZraS\|+ |
| STM4174_471_505 | 0.15 | -1.28 | 1255700\|hydG\|transcriptional regulatory protein ZraR\|+ |
| STM4175_803_837 | 0.00 | -3.78 | 1255701\|purD\|phosphoribosylamine--glycine ligase\|- |
| STM4176_1503_1537 | 0.00 | -4.97 | 1255702\|purH\|bifunctional phosphoribosylaminoimidazolecarboxamide formyltransferase/IMP cyclohydrolase\|- |
| STM4181_289_323 | 0.94 | -1.01 | 1255707\|yjaB\|hypothetical protein\|- |
| STM4182_385_419 | 0.01 | 13.69 | 1255708\|metA\|homoserine O-succinyltransferase\|+ |

| | | | |
|---|---|---|---|
| STM4183_1115_1149 | 0.91 | 1.02 | 1255709\|aceB\|malate synthase\|+ |
| STM4184_434_468 | 0.07 | -1.62 | 1255710\|aceA\|isocitrate lyase\|+ |
| STM4185_1137_1171 | 0.05 | -1.39 | 1255711\|aceK\|bifunctional isocitrate dehydrogenase kinase/phosphatase protein\|+ |
| STM4186_345_379 | 0.19 | 1.29 | 1255712\|STM4186\|putative cytoplasmic protein\|- |
| STM4187_65_99 | 0.08 | -1.31 | 1255713\|iclR\|acetate operon transcriptional repressor IclR\|- |
| STM4188.S_2813_2847 | 0.10 | -1.44 | 1255714\|metH\|B12-dependent methionine synthase\|+ |
| STM4189_1113_1147 | 0.19 | 1.28 | 1255715\|yjbB\|putative transport protein\|+ |
| STM4190_133_167 | 0.02 | -2.20 | 1255716\|pepE\|peptidase E\|- |
| STM4191_41_75 | 0.05 | 1.73 | 1255717\|STM4191\|putative cytoplasmic protein\|+ |
| STM4192_353_387 | 0.01 | -2.39 | 1255718\|STM4192\|putative cytoplasmic protein\|+ |
| STM4193_545_579 | 0.21 | -1.21 | 1255719\|yjbC\|23S rRNA pseudouridine synthase F\|+ |
| STM4194_132_166 | 0.02 | 1.48 | 1255720\|yjbD\|hypothetical protein\|- |
| STM4195_417_451 | 0.01 | -1.34 | 1255721\|STM4195\|putative Na+-dependent transporter\|- |
| STM4196_549_587 | 0.02 | 2.83 | 1255722\|STM4196\|putative cytoplasmic protein\|- |
| STM4197_109_144 | 0.07 | 2.18 | 1255723\|STM4197\|putative inner membrane protein\|- |
| STM4198_321_355 | 0.50 | 1.10 | 1255724\|STM4198\|putative cytoplasmic protein\|- |
| STM4199_481_515 | 0.19 | -1.13 | 1255725\|STM4199\|putative cytoplasmic protein\|- |
| STM4202_1077_1111 | 0.23 | -1.23 | 1255728\|STM4202\|putative phage baseplate protein\|- |
| STM4204_869_903 | 0.12 | 1.97 | 1255730\|STM4204\|putative inner membrane protein\|- |
| STM4205_841_875 | 0.09 | 2.10 | 1255731\|STM4205\|putative phage glycosyltransferase\|- |
| STM4206_200_234 | 0.02 | 1.26 | 1255732\|STM4206\|putative phage glucose translocase\|- |
| STM4207_369_403 | 0.04 | 2.00 | 1255733\|STM4207\|putative phage baseplate component\|- |
| STM4208_237_271 | 0.01 | 1.70 | 1255734\|STM4208\|putative cytoplasmic protein\|- |
| STM4209_33_67 | 0.11 | -1.18 | 1255735\|STM4209\|putative inner membrane protein\|- |
| STM4210_481_515 | 0.02 | 2.59 | 1255736\|STM4210\|putative methyl-accepting chemotaxis protein\|- |
| STM4211.1N_193_227 | 0.20 | -1.13 | 2673741\|STM4211.1N\|-\|- |
| STM4213_957_991 | 0.36 | 1.08 | 1255739\|STM4213\|putative phage tail sheath protein\|- |
| STM4216_205_239 | 0.10 | 1.55 | 1255742\|STM4216\|putative inner membrane protein\|- |
| STM4217_312_346 | 0.08 | 2.18 | 1255743\|STM4217\|putative soluble lytic murein transglycosylase\|- |
| STM4218_45_79 | 0.03 | 1.48 | 1255744\|STM4218\|putative inner membrane protein\|- |
| STM4219.S_209_243 | 0.10 | 1.38 | 1255745\|STM4219.S\|putative cytoplasmic protein\|+ |
| STM4220_1231_1265 | 0.44 | -1.13 | 1255746\|lysC\|aspartate kinase III\|- |
| STM4221_1323_1357 | 0.25 | 1.23 | 1255747\|pgi\|glucose-6-phosphate isomerase\|+ |
| STM4222.S_120_154 | 0.13 | 1.75 | 1255748\|yjbE\|putative outer membrane protein\|+ |
| STM4223_321_355 | 0.64 | -1.05 | 1255749\|yjbF\|putative outer membrane lipoprotein\|+ |

| | | | |
|---|---|---|---|
| STM4224_321_355 | 0.37 | -1.11 | 1255750lyjbGlputative periplasmic proteinl+ |
| STM4225_1274_1308 | 0.49 | 1.14 | 1255751lyjbHlputative outer membrane lipoproteinl+ |
| STM4226_321_355 | 0.04 | 2.36 | 1255752lyjbAlphosphate-starvation-inducible protein PsiEl+ |
| STM4227_737_771 | 0.39 | 1.16 | 1255753lmalGlmaltose transporter permeasel- |
| STM4229_1064_1098 | 0.09 | -1.34 | 1255755lmalElmaltose ABC transporter periplasmic proteinl- |
| STM4230_975_1009 | 0.05 | 2.48 | 1255756lmalKlmaltose/maltodextrin transporter ATP-binding proteinl+ |
| STM4231_760_794 | 0.08 | -1.24 | 1255757llamBlmaltoporinl+ |
| STM4232_545_579 | 0.05 | 1.10 | 1255758lmalMlmaltose regulon periplasmic proteinl+ |
| STM4233_193_227 | 0.01 | 1.91 | 1255759lubiClchorismate pyruvate lyasel+ |
| STM4234_33_67 | 0.02 | 1.25 | 1255760lubiAl4-hydroxybenzoate octaprenyltransferasel+ |
| STM4235_2254_2288 | 0.00 | -1.98 | 1255761lplsBlglycerol-3-phosphate acyltransferasel- |
| STM4236_278_312 | 0.05 | -1.51 | 1255762ldgkAldiacylglycerol kinasel+ |
| STM4237_283_317 | 0.10 | 1.26 | 1255763llexAlLexA repressorl+ |
| STM4238_1031_1065 | 0.95 | 1.01 | 1255764ldinFlDNA-damage-inducible SOS response proteinl+ |
| STM4239_73_107 | 0.03 | 1.43 | 1255765lSTM4239lputative cytoplasmic proteinl+ |
| STM4240_129_163 | 0.10 | 1.49 | 1255766lyjbJlputative stress-response proteinl+ |
| STM4241_113_147 | 0.03 | 1.43 | 1255767lzurlzinc uptake transcriptional repressorl- |
| STM4243_705_739 | 0.24 | -1.22 | 1255769lyjbNltRNA-dihydrouridine synthase Al+ |
| STM4244_121_155 | 0.01 | 1.76 | 1255770lpspGlphage shock protein Gl+ |
| STM4245_265_299 | 0.12 | -1.07 | 1255771lqorlquinone oxidoreductase  NADPH-dependentl- |
| STM4246_1313_1347 | 0.03 | -1.81 | 1255772ldnaBlreplicative DNA helicasel+ |
| STM4247_849_883 | 0.02 | -2.07 | 1255773lalrlalanine racemasel+ |
| STM4248_611_645 | 0.05 | -2.22 | 1255774ltyrBlaromatic amino acid aminotransferasel+ |
| STM4249_577_611 | 0.01 | -1.72 | 1255775laphAlacid phosphatase/phosphotransferasel+ |
| STM4250_369_403 | 0.03 | 1.45 | 1255776lyjbQlputative cytoplasmic proteinl+ |
| STM4251_257_291 | 0.30 | 1.19 | 1255777lyjbRlputative cytoplasmic proteinl+ |
| STM4252_281_315 | 0.15 | 1.26 | 1255778lSTM4252lputative inner membrane proteinl- |
| STM4254_2659_2693 | 0.98 | 1.01 | 1255780luvrAlexcinuclease ABC subunit Al- |
| STM4255_33_67 | 0.78 | -1.08 | 1255781lSTM4255lputative cytoplasmic proteinl+ |
| STM4256_226_260 | 0.23 | 1.24 | 1255782lssblsingle-strand DNA-binding proteinl+ |
| STM4257_273_307 | 0.06 | 3.22 | 1255783lSTM4257lhypothetical proteinl+ |
| STM4258_506_540 | 0.06 | 2.50 | 1255784lSTM4258lputative methyl-accepting chemotaxis proteinl+ |
| STM4259_753_787 | 0.02 | 3.05 | 1255785lSTM4259lputative ABC exporter outer membrane componentl+ |
| STM4260_1199_1233 | 0.82 | 1.15 | 1255786lSTM4260lpredicted cation efflux pumpl+ |
| STM4261_7001_7035 | 0.16 | -1.29 | 1255787lSTM4261lputative inner membrane proteinl+ |
| STM4262_1948_1982 | 0.01 | 2.20 | 1255788lSTM4262lputative ABC-type bacteriocin/lantibiotic exporterl+ |

| | | | |
|---|---|---|---|
| STM4263_177_211 | 0.00 | 12.42 | 1255789lyjcBlputative inner membrane proteinl- |
| STM4264_1467_1501 | 0.30 | -1.09 | 1255790lyjcClputative diguanylate cyclase/phosphodiesterasel+ |
| STM4265_113_147 | 0.16 | 1.47 | 1255791lsoxSlDNA-binding transcriptional dual regulatorl- |
| STM4266_289_323 | 0.02 | 3.22 | 1255792lsoxRlredox-sensing transcriptional activatorl+ |
| STM4267_145_179 | 0.50 | -1.11 | 1255793lSTM4267lputative glutathione S-transferasel+ |
| STM4268_735_769 | 0.01 | -3.26 | 1255794lyjcDlhypothetical proteinl+ |
| STM4269_1416_1450 | 0.02 | 1.34 | 1255795lyjcElNa/H transport proteinl+ |
| STM4270_161_195 | 0.20 | 1.16 | 1255796lSTM4270lputative transcriptional regulatorl- |
| STM4271_297_331 | 0.05 | -1.37 | 1255797lSTM4271lputative inner membrane proteinl+ |
| STM4273_763_797 | 0.01 | -7.54 | 1255799lactPlacetate permeasel- |
| STM4274_73_107 | 0.00 | -8.67 | 1255800lyjcHlputative inner membrane proteinl- |
| STM4275_1376_1410 | 0.00 | -46.59 | 1255801lacslacetyl-CoA synthetasel- |
| STM4276_45_83 | 0.17 | 1.27 | 1255802lSTM4276lputative cytoplasmic proteinl+ |
| STM4277_1142_1176 | 0.23 | 1.10 | 1255803lnrfAlcytochrome c nitrite reductasel+ |
| STM4279_545_579 | 0.33 | 1.15 | 1255805lnrfClputative formate-dependent nitrite reductasel+ |
| STM4280_441_475 | 0.29 | -1.14 | 1255806lnrfDlputative formate-dependent nitrate reductasel+ |
| STM4281_2008_2042 | 0.10 | 1.17 | 1255807lnrfElformate-dependent nitrite reductasel+ |
| STM4282_369_403 | 0.47 | 1.14 | 1255808lnrfGlformate-dependent nitrite reductase complex subunit NrfGl+ |
| STM4284_513_547 | 0.33 | 1.53 | 1255810lyjcOltetratricopeptide repeat proteinl- |
| STM4285_1597_1631 | 0.05 | 1.28 | 1255811lfdhFlformate dehydrogenasel- |
| STM4286_129_163 | 0.05 | 1.20 | 1255812llpxOlputative dioxygenasel- |
| STM4287.S_9_43 | 0.01 | 2.65 | 1255813lphnOlaminoalkylphosphonic acid N-acetyltransferasel- |
| STM4288_129_163 | 0.94 | -1.01 | 1255814lphnBlhypothetical proteinl- |
| STM4289_119_153 | 0.42 | 1.10 | 1255815lphnAlhypothetical proteinl- |
| STM4290_840_874 | 0.06 | 1.69 | 1255816lproPlproline/glycine betaine transporterl+ |
| STM4291_376_410 | 0.00 | 1.59 | 1255817lbasSlsensor protein BasS/PmrBl- |
| STM4292_385_419 | 0.01 | 1.96 | 1255818lbasRlDNA-binding response regulator in two-component regulatory system with BasSl- |
| STM4293_1349_1383 | 0.04 | 1.93 | 1255819lyjdBlpredicted metal dependent hydrolasel- |
| STM4294_531_565 | 0.09 | 1.14 | 1255820lyjdElarginine:agmatin antiporterl- |
| STM4295_257_291 | 0.01 | 3.10 | 1255821ladiYltranscriptional activatorl- |
| STM4297_577_611 | 0.91 | 1.04 | 1255823lmelRlDNA-binding transcriptional dual regulatorl- |
| STM4299_568_602 | 0.03 | 1.35 | 1255825lmelBlmelibiose:sodium symporterl+ |
| STM4300_1072_1106 | 0.02 | -1.85 | 1255826lfumBlfumarase Bl- |
| STM4301_1142_1176 | 0.01 | -1.54 | 1255827ldcuBlanaerobic C4-dicarboxylate transporterl- |
| STM4302_9_43 | 0.27 | -1.30 | 1255828lSTM4302lputative cytoplasmic proteinl- |

| | | | |
|---|---|---|---|
| STM4304_1369_1403 | 0.00 | -3.70 | 1255830\|dcuS\|sensory histidine kinase DcuS\|- |
| STM4305.S_2055_2089 | 0.01 | -3.00 | 1255831\|STM4305.S\|putative anaerobic dimethylsulfoxide reductase subunit A\|+ |
| STM4306_513_547 | 0.25 | -1.36 | 1255832\|STM4306\|putative anaerobic dimethylsulfoxide reductase subunit B\|+ |
| STM4307_105_139 | 0.06 | -1.24 | 1255833\|STM4307\|putative anaerobic dimethylsulfoxide reductase subunit C\|+ |
| STM4308_390_424 | 0.03 | -1.61 | 1255834\|STM4308\|putative anaerobic dehydrogenase component\|+ |
| STM4309_850_884 | 0.02 | 1.29 | 1255835\|STM4309\|hypothetical protein\|- |
| STM4310_641_675 | 0.06 | -2.09 | 1255836\|STM4310\|putative inner membrane protein\|+ |
| STM4312_49_83 | 0.02 | 2.12 | 1255838\|STM4312\|hypothetical protein\|- |
| STM4313_81_115 | 0.03 | 1.41 | 1255839\|STM4313\|putative cytoplasmic protein\|- |
| STM4314_119_153 | 0.08 | 1.90 | 1255840\|STM4314\|putative regulatory protein\|- |
| STM4316_51_85 | 0.16 | 1.24 | 1255842\|STM4316\|putative cytoplasmic protein\|- |
| STM4317_153_187 | 0.07 | 1.25 | 1255843\|STM4317\|hypothetical protein\|+ |
| STM4318_145_179 | 0.01 | 1.67 | 1255844\|STM4318\|putative acetyltransferase\|+ |
| STM4319_457_491 | 0.00 | 4.54 | 1255845\|phoN\|non-specific acid phosphatase\|- |
| STM4320_41_75 | 0.73 | 1.03 | 1255846\|STM4320\|putative regulatory protein\|+ |
| STM4322_513_547 | 0.48 | 1.09 | 1255848\|yjdC\|putative transcriptional regulator\|- |
| STM4324_247_281 | 0.03 | 1.27 | 1255850\|cutA\|divalent-cation tolerance protein CutA\|- |
| STM4325_911_945 | 0.13 | -1.71 | 1255851\|dcuA\|anaerobic C4-dicarboxylate transporter\|- |
| STM4326_1078_1112 | 0.00 | -4.14 | 1255852\|aspA\|aspartate ammonia-lyase\|- |
| STM4327_49_83 | 0.26 | 1.24 | 1255853\|fxsA\|FxsA\|+ |
| STM4328_883_917 | 0.03 | 3.34 | 1255854\|yjeH\|inner membrane protein YjeH\|- |
| STM4329_49_83 | 0.17 | 1.24 | 1255855\|groES\|co-chaperonin GroES\|+ |
| STM4330_760_794 | 0.29 | 1.42 | 1255856\|groEL\|chaperonin GroEL\|+ |
| STM4331_146_180 | 0.22 | -1.11 | 1255857\|yjeI\|putative outer membrane lipoprotein\|+ |
| STM4332_353_387 | 0.78 | -1.02 | 1255858\|yjeJ\|putative inner membrane protein\|- |
| STM4333_366_400 | 0.01 | 1.24 | 1255859\|yjeK\|putative aminomutase\|- |
| STM4334_513_547 | 0.01 | -2.19 | 1255860\|efp\|elongation factor P\|+ |
| STM4335_41_75 | 0.02 | -1.63 | 1255861\|ecnA\|putative entericidin A precursor\|+ |
| STM4337_257_291 | 0.02 | 1.41 | 1255863\|ecnR\|putative regulatory protein\|- |
| STM4338_257_291 | 0.07 | 1.28 | 1255864\|sugE\|quaternary ammonium compound-resistance protein SugE\|+ |
| STM4339_401_435 | 0.08 | 1.49 | 1255865\|blc\|outer membrane lipoprotein Blc\|- |
| STM4340_291_325 | 0.00 | -3.79 | 1255866\|frdD\|fumarate reductase subunit D\|- |
| STM4341_359_393 | 0.00 | -11.56 | 1255867\|frdC\|fumarate reductase subunit C\|- |
| STM4342_193_227 | 0.00 | -6.59 | 1255868\|frdB\|fumarate reductase iron-sulfur subunit\|- |
| STM4343_984_1018 | 0.00 | -3.99 | 1255869\|frdA\|fumarate reductase flavoprotein subunit\|- |

| | | | |
|---|---|---|---|
| STM4344_417_451 | 0.01 | -2.37 | 1255870lyjeAllysyl-tRNA synthetasel+ |
| STM4345_672_706 | 0.07 | 1.31 | 1255871lyjeMlputative amino-acid transport proteinl+ |
| STM4346_87_121 | 0.07 | -1.27 | 1255872lyjeOlputative inner membrane proteinl+ |
| STM4347_2584_2618 | 0.45 | -1.07 | 1255873lyjePlpredicted mechanosensitive channell- |
| STM4348_449_483 | 0.01 | -1.28 | 1255874lpsdlphosphatidylserine decarboxylasel- |
| STM4349_182_216 | 0.05 | -1.35 | 1255875lyjeQlribosome-associated GTPasel- |
| STM4350_209_243 | 0.10 | -1.59 | 1255876lornloligoribonucleasel+ |
| STM4351_257_291 | 0.02 | 1.49 | 1255877lSTM4351lputative arginine-binding periplasmic proteinl- |
| STM4355.S_461_495 | 0.17 | 1.18 | 1255881lyjeSlputative FeS proteinl- |
| STM4356_1061_1095 | 0.01 | -1.20 | 1255882lyjeFlhypothetical proteinl+ |
| STM4357_417_451 | 0.00 | -1.35 | 1255883lyjeElputative ATPasel+ |
| STM4358_1121_1155 | 0.05 | -1.64 | 1255884lamiBlN-acetylmuramoyl-l-alanine amidase IIl+ |
| STM4359_1306_1340 | 0.43 | 1.05 | 1255885lmutLlDNA mismatch repair proteinl+ |
| STM4360_801_835 | 0.27 | -1.12 | 1255886lmiaAltRNA delta(2)-isopentenylpyrophosphate transferasel+ |
| STM4361_165_199 | 0.18 | 1.10 | 1255887lhfqlRNA-binding protein Hfql+ |
| STM4362_474_508 | 0.13 | -1.09 | 1255888lhflXlputative GTPase HflXl+ |
| STM4363_581_615 | 0.04 | -1.44 | 1255889lhflKlFtsH protease regulator HflKl+ |
| STM4364_934_968 | 0.18 | 1.26 | 1255890lhflClFtsH protease regulator HflCl+ |
| STM4365_153_187 | 0.08 | 1.87 | 1255891lyjeTlputative inner membrane proteinl+ |
| STM4366_876_910 | 0.40 | -1.15 | 1255892lpurAladenylosuccinate synthetasel+ |
| STM4367_353_387 | 0.02 | 1.33 | 1255893lyjeBltranscriptional repressor NsrRl+ |
| STM4368_2144_2178 | 0.01 | 1.83 | 1255894lvacBlexoribonuclease Rl+ |
| STM4369_193_227 | 0.02 | 1.53 | 1255895lyjfHl23S rRNA (guanosine-2'-O-)-methyltransferasel+ |
| STM4370_241_275 | 0.17 | 1.25 | 1255896lyjfIlputative cytoplasmic proteinl+ |
| STM4372_936_970 | 0.20 | 1.11 | 1255898lSTM4372lputative potassium channelsl+ |
| STM4374_184_218 | 0.04 | 2.22 | 1255900lyjfLlputative inner membrane proteinl+ |
| STM4375_209_243 | 0.37 | 1.34 | 1255901lyjfMlputative inner membrane proteinl+ |
| STM4376_357_391 | 0.08 | -1.26 | 1255902lyjfClputative glutathionylspermidine synthasel+ |
| STM4377_1426_1460 | 0.00 | -4.74 | 1255903laidBlisovaleryl CoA dehydrogenasel+ |
| STM4378_153_187 | 0.00 | -3.80 | 1255904lyjfNlputative inner membrane proteinl- |
| STM4379_9_43 | 0.05 | -1.77 | 1255905lyjfOlputative lipoproteinl- |
| STM4380_641_675 | 0.04 | 1.74 | 1255906lyjfPlpredicted hydrolasel+ |
| STM4381_273_307 | 0.09 | 1.24 | 1255907lyjfQlputative transcriptional repressorl- |
| STM4382_930_964 | 0.37 | -1.10 | 1255908lyjfRlputative L-ascorbate 6-phosphate lactonasel- |
| STM4384_161_195 | 0.98 | 1.01 | 1255910lsgaBlL-ascorbate-specific enzyme IIB component of PTSl+ |
| STM4386_321_355 | 0.44 | 1.08 | 1255912lulaDl3-keto-L-gulonate-6-phosphate decarboxylasel+ |

| | | | |
|---|---|---|---|
| STM4387_609_643 | 0.30 | 1.13 | 1255913\|sgaU\|L-xylulose 5-phosphate 3-epimerase\|+ |
| STM4388_593_627 | 0.02 | 1.90 | 1255914\|sgaE\|L-ribulose-5-phosphate 4-epimerase\|+ |
| STM4389_115_149 | 0.01 | 1.87 | 1255915\|yjfY\|putative outer membrane protein\|- |
| STM4391_41_75 | 0.01 | -2.89 | 1255917\|rpsF\|30S ribosomal protein S6\|+ |
| STM4392_65_99 | 0.01 | -3.55 | 1255918\|priB\|primosomal replication protein N\|+ |
| STM4393_105_139 | 0.01 | -3.43 | 1255919\|rpsR\|30S ribosomal protein S18\|+ |
| STM4394_129_163 | 0.02 | -2.89 | 1255920\|rplI\|50S ribosomal protein L9\|+ |
| STM4395_337_371 | 0.22 | -1.38 | 1255921\|yifZ\|putative permease\|+ |
| STM4396_41_75 | 0.77 | 1.02 | 1255922\|ytfB\|putative cell envelope opacity-associated protein A\|- |
| STM4397_17_51 | 0.05 | -1.65 | 1255923\|fklB\|peptidyl-prolyl cis-trans isomerase\|+ |
| STM4398_667_701 | 0.06 | -1.57 | 1255924\|cycA\|D-alanine/D-serine/glycine permease\|+ |
| STM4401_389_423 | 0.12 | 1.91 | 1255927\|ytfG\|putative reductase\|- |
| STM4402_225_259 | 0.44 | -1.26 | 1255928\|ytfH\|putative transcriptional regulator\|+ |
| STM4403_1297_1331 | 0.07 | -1.17 | 1255929\|cpdB\|bifunctional 2' 3'-cyclic nucleotide 2'-phosphodiesterase/3'-nucleotidase periplasmic precursor protein\|- |
| STM4404_513_547 | 0.01 | 1.87 | 1255930\|cysQ\|PAPS (adenosine 3'-phosphate 5'-phosphosulfate) 3'(2') 5'-bisphosphate nucleotidase\|+ |
| STM4405_321_355 | 0.45 | -1.20 | 1255931\|ytfJ\|putative transcriptional regulator\|- |
| STM4406.S_57_91 | 0.00 | -1.63 | 1255932\|ytfK\|putative cytoplasmic protein\|+ |
| STM4407_665_699 | 0.04 | 1.83 | 1255933\|ytfL\|putative hemolysin-like protein\|- |
| STM4408_225_259 | 0.01 | 2.72 | 1255934\|msrA\|methionine sulfoxide reductase A\|- |
| STM4409_1295_1329 | 0.36 | 1.07 | 1255935\|ytfM\|putative outer membrane protein\|+ |
| STM4410_3549_3583 | 0.16 | -1.13 | 1255936\|ytfN\|putative periplasmic protein\|+ |
| STM4411_257_291 | 0.41 | -1.09 | 1255937\|ytfP\|putative cytoplasmic protein\|+ |
| STM4412_770_804 | 0.02 | 1.49 | 1255938\|STM4412\|putative permease\|- |
| STM4413_421_455 | 0.00 | 3.10 | 1255939\|STM4413\|putative metallo-dependent hydrolase\|- |
| STM4414_65_99 | 0.19 | -1.33 | 1255940\|ppa\|inorganic pyrophosphatase\|- |
| STM4415_689_723 | 0.41 | -1.13 | 1255941\|fbp\|fructose-1 6-bisphosphatase\|- |
| STM4416_829_863 | 0.64 | 1.08 | 1255942\|mpl\|UDP-N-acetylmuramate/L-alanyl-gamma-D-glutamyl-meso-diaminopimelate ligase\|+ |
| STM4417_417_451 | 0.30 | 1.08 | 1255943\|STM4417\|putative transcriptional regulator\|+ |
| STM4418_835_869 | 0.19 | -1.19 | 1255944\|STM4418\|sugar transporter\|- |
| STM4419_1014_1048 | 0.62 | -1.10 | 1255945\|STM4419\|sugar transporter\|+ |
| STM4421_1405_1439 | 0.01 | -2.12 | 1255947\|STM4421\|putative NAD-dependent aldehyde dehydrogenase\|- |
| STM4425_12_46 | 0.05 | 1.24 | 1255951\|STM4425\|putative dehydrogenase\|+ |
| STM4426_505_539 | 0.01 | 2.72 | 1255952\|srfJ\|lysosomal glucosyl ceramidase-like protein\|+ |

| | | | |
|---|---|---|---|
| STM4427_513_547 | 0.02 | 1.41 | 1255953lSTM4427lputative endonucleasel+ |
| STM4429_754_788 | 0.29 | 1.30 | 1255955lSTM4429l-l- |
| STM4431_1396_1430 | 0.15 | 1.09 | 2673766lSTM4431l-l+ |
| STM4433_760_794 | 0.00 | 1.98 | 1255959lSTM4433lmyo-inositol 2-dehydrogenasel+ |
| STM4434_900_934 | 0.63 | -1.07 | 1255960lSTM4434lputative permeasel+ |
| STM4435_317_351 | 0.28 | 1.18 | 1255961lSTM4435lputative cytoplasmic proteinl+ |
| STM4436_289_323 | 0.01 | -1.38 | 1255962lSTM4436lputative endonucleasel+ |
| STM4437_425_459 | 0.02 | 1.73 | 1255963lyjgAlhypothetical proteinl- |
| STM4438_386_420 | 0.02 | 1.67 | 1255964lpmbAlpeptidase PmbAl+ |
| STM4439_249_283 | 0.04 | -1.34 | 1255965lcybClcytochrome b562l+ |
| STM4440_33_67 | 0.02 | -1.41 | 1255966lSTM4440lputative cytoplasmic proteinl+ |
| STM4441_225_259 | 0.69 | -1.04 | 1255967lSTM4441lputative cytoplasmic proteinl+ |
| STM4442_241_275 | 0.24 | 1.24 | 1255968lSTM4442lputative cytoplasmic proteinl+ |
| STM4444_97_131 | 0.10 | -1.25 | 1255970lSTM4444lputative inner membrane proteinl+ |
| STM4445_839_873 | 0.14 | -1.16 | 1255971lSTM4445ldihydroorotasel+ |
| STM4446_684_718 | 0.13 | 1.30 | 1255972lSTM4446lputative selenocysteine synthasel+ |
| STM4447_497_531 | 0.02 | -1.30 | 1255973lSTM4447lputative periplasmic proteinl+ |
| STM4448_1619_1653 | 0.30 | 1.11 | 1255974lSTM4448lputative phosphotransferase system mannitol/fructose-specific IIA domainl+ |
| STM4449_88_122 | 0.00 | -1.44 | 1255975lSTM4449lbifunctional antitoxin/transcriptional repressor RelBl+ |
| STM4450_160_194 | 0.00 | -1.31 | 1255976lSTM4450lputative inner membrane proteinl+ |
| STM4451_429_463 | 0.41 | 1.15 | 1255977lnrdGlanaerobic ribonucleotide reductase-activating proteinl- |
| STM4452_1908_1942 | 0.06 | -1.58 | 1255978lnrdDlanaerobic ribonucleoside triphosphate reductasel- |
| STM4452.1N_60_94 | 0.08 | 2.45 | 2673768lSTM4452.1Nlhypothetical proteinl+ |
| STM4454_739_773 | 0.02 | -2.64 | 1255980ltreBlpseudol- |
| STM4455_481_515 | 0.12 | 1.28 | 1255981ltreRltrehalose repressorl- |
| STM4457_281_315 | 0.03 | 1.79 | 1255983lSTM4457lputative transposasel- |
| STM4458_65_99 | 0.09 | -1.75 | 1255984lyjgFlputative translation initiation inhibitorl- |
| STM4459_33_67 | 0.01 | 2.00 | 1255985lpyrIlaspartate carbamoyltransferase regulatory subunitl- |
| STM4460_586_620 | 0.48 | -1.20 | 1255986lpyrBlaspartate carbamoyltransferase catalytic subunitl- |
| STM4461_50_84 | 0.01 | 2.14 | 1255987lpyrLlpyrBI operon leader peptidel- |
| STM4464_533_567 | 0.78 | -1.07 | 1255990lSTM4464lputative arginine repressorl- |
| STM4465_54_88 | 0.04 | 2.69 | 1255991lSTM4465lornithine carbamoyltransferasel- |
| STM4468_89_123 | 0.01 | -1.64 | 1255994lyjgKlputative cytoplasmic proteinl+ |
| STM4469_438_472 | 0.34 | -1.07 | 1255995larglIornithine carbamoyltransferase subunit Il- |
| STM4470_81_115 | 0.02 | 1.35 | 1255996lyjgDlhypothetical proteinl+ |

| | | | |
|---|---|---|---|
| STM4471_513_547 | 0.44 | -1.10 | 1255997lmiaElhydroxylasel+ |
| STM4472_267_306 | 0.00 | 2.18 | 1255998lytgAlputative inner membrane proteinl- |
| STM4473_55_89 | 0.00 | 2.15 | 1255999lyjgMlputative acetyltransferasel- |
| STM4474_717_751 | 0.04 | 1.39 | 1256000lyjgNlputative inner membrane proteinl+ |
| STM4475_2273_2307 | 0.35 | -1.14 | 1256001lvalSlvalyl-tRNA synthetasel- |
| STM4476.S_9_43 | 0.32 | 1.09 | 1256002lholClDNA polymerase III subunit chil- |
| STM4477_545_579 | 0.02 | -1.26 | 1256003lpepAlleucyl aminopeptidasel- |
| STM4478_85_119 | 0.34 | -1.15 | 1256004lSTM4478lputative cytoplasmic proteinl- |
| STM4479_998_1032 | 0.29 | 1.20 | 1256005lyjgPlputative permeasel+ |
| STM4480_516_550 | 0.04 | -1.32 | 1256006lyjgQlputative permeasel+ |
| STM4482_977_1011 | 0.01 | -2.95 | 1256008lidnTlL-idonate transport proteinl- |
| STM4483_289_323 | 0.45 | -1.08 | 1256009lidnOlgluconate 5-dehydrogenasel- |
| STM4484_241_275 | 0.01 | -1.61 | 1256010lidnDlL-idonate 5-dehydrogenase NAD-bindingl- |
| STM4486_853_887 | 0.01 | -2.68 | 1256012lyjgBlputative alcohol dehydrogenasel- |
| STM4487_21_55 | 0.46 | -1.41 | 1256013lleuXltRNAl+ |
| STM4488_97_131 | 0.01 | 2.43 | 1256014lSTM4488lputative integrasel+ |
| STM4489_3381_3415 | 0.07 | -1.51 | 1256015lSTM4489lputative DNA helicasesl+ |
| STM4490_321_355 | 0.08 | -1.36 | 1256016lSTM4490lputative restriction endonucleasel+ |
| STM4491_2046_2080 | 0.65 | -1.05 | 1256017lSTM4491lputative ATP-dependent Lon proteasel- |
| STM4492_2319_2353 | 0.63 | 1.04 | 1256018lSTM4492lputative cytoplasmic proteinl- |
| STM4493_273_307 | 0.09 | -1.18 | 1256019lSTM4493lputative cytoplasmic proteinl- |
| STM4494_797_831 | 0.04 | -1.54 | 1256020lSTM4494lputative ABC-type sugar/spermidine/putrescine transport system ATPase componentl- |
| STM4495_2951_2985 | 0.52 | -1.28 | 1256021lSTM4495lputative type II restriction enzyme methylase subunitl- |
| STM4496_2675_2709 | 0.03 | -1.66 | 1256022lSTM4496lputative DNA repair ATPasel- |
| STM4497_228_262 | 0.05 | 2.01 | 1256023lSTM4497lputative cytoplasmic proteinl- |
| STM4498_161_195 | 0.48 | -1.11 | 1256024lSTM4498lputative inner membrane proteinl- |
| STM4498.1N_2_36 | 0.83 | -1.01 | 2673776lSTM4498.1Nl-l+ |
| STM4499_257_291 | 0.05 | 1.15 | 1256025lyeeNlhypothetical proteinl- |
| STM4500_1049_1083 | 0.87 | -1.02 | 1256026lyjhPlputative SAM-dependent methyltransferasel- |
| STM4502_569_603 | 0.02 | 2.55 | 1256028lSTM4502lputative cytoplasmic proteinl+ |
| STM4503_249_283 | 0.02 | 1.34 | 1256029lSTM4503lputative inner membrane proteinl+ |
| STM4504_353_387 | 0.00 | 3.26 | 1256030lSTM4504lputative cytoplasmic proteinl+ |
| STM4505_385_419 | 0.03 | 2.09 | 1256031lSTM4505lhypothetical proteinl+ |
| STM4506_257_291 | 0.27 | 1.30 | 1256032lSTM4506lputative dienelactone hydrolasel- |
| STM4507_209_243 | 0.00 | -1.73 | 1256033luxuRlDNA-binding transcriptional repressorl+ |

| | | | |
|---|---|---|---|
| STM4509.S_229_263 | 0.36 | -1.10 | 1256035lSTM4509.Slputative cytoplasmic proteinl+ |
| STM4510_177_211 | 0.00 | -1.74 | 1256036lSTM4510lputative aspartate racemasel+ |
| STM4511_833_867 | 0.09 | -1.42 | 1256037lyjiElputative DNA-binding transcriptional regulatorl- |
| STM4512_750_784 | 0.01 | -1.62 | 1256038liadAlisoaspartyl dipeptidasel- |
| STM4513_129_163 | 0.09 | 1.35 | 1256039lyjiGlhypothetical proteinl- |
| STM4514.S_547_581 | 0.13 | -1.25 | 1256040lyjiHlputative inner membrane proteinl- |
| STM4517_1171_1205 | 0.45 | 1.23 | 1256043lyjiOlputative transport proteinl- |
| STM4518_257_291 | 0.17 | 1.18 | 1256044lSTM4518lputative inner membrane proteinl+ |
| STM4519_1028_1062 | 0.01 | -2.25 | 1256045lSTM4519lputative NAD-dependent aldehyde dehydrogenasel+ |
| STM4520_15_49 | 0.29 | 1.31 | 1256046lSTM4520lputative cytoplasmic proteinl- |
| STM4521_97_131 | 0.12 | 1.73 | 1256047lyjiSlputative cytoplasmic proteinl+ |
| STM4522_91_128 | 0.01 | 2.86 | 1256048lSTM4522lputative inner membrane proteinl- |
| STM4523_65_99 | 0.00 | 1.94 | 1256049lyjiWlputative SOS response proteinl- |
| STM4524_563_597 | 0.05 | 1.99 | 1256050lhsdSltype I restriction enzyme specificity proteinl- |
| STM4525_1207_1241 | 0.01 | 1.61 | 1256051lhsdMlDNA methylase Ml- |
| STM4527_593_627 | 0.03 | -1.26 | 1256053lmrrlrestriction endonucleasel+ |
| STM4528_217_251 | 0.02 | 2.13 | 1256054lSTM4528lputative inner membrane proteinl+ |
| STM4529_233_267 | 0.58 | 1.08 | 1256055lSTM4529lputative cytoplasmic proteinl+ |
| STM4530_337_371 | 0.17 | 1.20 | 1256056lyjiAlputative GTP-binding protein YjiAl- |
| STM4531_103_137 | 0.79 | 1.11 | 1256057lyjiXlputative cytoplasmic proteinl- |
| STM4532_1152_1186 | 1.00 | -1.00 | 1256058lyjiYlputative carbon starvation proteinl- |
| STM4533_1263_1297 | 0.01 | -2.31 | 1256059ltsrlmethyl-accepting chemotaxis protein Il+ |
| STM4534_2223_2257 | 0.00 | -3.84 | 1256060lSTM4534lputative transcriptional regulatorl+ |
| STM4535_309_343 | 0.16 | 1.87 | 1256061lSTM4535lputative PTS permeasel+ |
| STM4538_705_739 | 0.05 | 2.01 | 1256064lSTM4538lputative PTS permeasel+ |
| STM4539_575_609 | 0.01 | -3.09 | 1256065lSTM4539lputative glucosamine-fructose-6-phosphate aminotransferasel+ |
| STM4540.S_460_494 | 0.79 | 1.04 | 1256066lSTM4540.Slputative glucosamine-fructose-6-phosphate aminotransferasel+ |
| STM4541_1741_1775 | 0.75 | -1.04 | 1256067lmdoBlphosphoglycerol transferase Il- |
| STM4542_357_391 | 0.01 | 2.07 | 1256068lyjjAlhypothetical proteinl- |
| STM4543_257_291 | 0.54 | 1.09 | 1256069ldnaClDNA replication protein DnaCl- |
| STM4544_177_211 | 0.02 | 1.54 | 1256070ldnaTlprimosomal protein Il- |
| STM4545_225_259 | 0.19 | -1.16 | 1256071lSTM4545lhypothetical proteinl- |
| STM4546_385_419 | 0.01 | 1.48 | 1256072lyjjPlhypothetical proteinl- |
| STM4547_577_611 | 0.31 | 1.18 | 1256073lyjjQlputative transcriptional regulatorl+ |

| | | | |
|---|---|---|---|
| STM4549_409_443 | 0.52 | 1.11 | 1256075lSTM4549lputative cytoplasmic proteinl- |
| STM4550_17_51 | 0.48 | -1.18 | 1256076lfhuFlferric iron reductase involved in ferric hydroximate transportl- |
| STM4551_898_932 | 0.27 | 1.06 | 1256077lSTM4551lhypothetical proteinl- |
| STM4552_43_77 | 0.33 | 1.06 | 1256078lSTM4552lputative inner membrane proteinl+ |
| STM4556_742_776 | 0.26 | -1.15 | 1256082lrsmCl16S ribosomal RNA m2G1207 methyltransferasel- |
| STM4557_249_283 | 0.05 | 1.44 | 1256083lholDlDNA polymerase III subunit psil+ |
| STM4558_193_227 | 0.00 | -1.46 | 1256084lrimIlribosomal-protein-alanine N-acetyltransferasel+ |
| STM4559.S_129_163 | 0.01 | -1.74 | 1256085lyjjGlnucleotidasel+ |
| STM4560_1103_1137 | 0.13 | -1.29 | 1256086lprfClpeptide chain release factor 3l+ |
| STM4561_385_419 | 0.05 | 1.71 | 1256087losmYlperiplasmic proteinl+ |
| STM4562_43_77 | 0.04 | 1.59 | 1256088lSTM4562lputative inner membrane proteinl+ |
| STM4563_523_557 | 0.12 | 1.26 | 1256089lyjjUlputative phosphoesterasel+ |
| STM4564_737_771 | 0.57 | 1.07 | 1256090lyjjVlputative deoxyribonuclease YjjVl+ |
| STM4565_649_683 | 0.02 | -1.54 | 1256091lyjjWlpyruvate formate lyase-activating enzymel- |
| STM4566_1020_1054 | 0.02 | -1.56 | 1256092lyjjIlhypothetical proteinl- |
| STM4567_225_259 | 0.01 | 1.31 | 1256093ldeoClddeoxyribose-phosphate aldolasel+ |
| STM4568_580_614 | 0.08 | -1.23 | 1256094ldeoAlthymidine phosphorylasel+ |
| STM4569_1089_1123 | 0.51 | -1.13 | 1256095ldeoBlphosphopentomutasel+ |
| STM4570_129_163 | 0.70 | 1.08 | 1256096ldeoDlpurine nucleoside phosphorylasel+ |
| STM4572_2170_2204 | 0.07 | 3.04 | 1256098lstjBlputative fimbrial usher proteinl- |
| STM4573_321_355 | 0.02 | 3.01 | 1256099lstjClputative periplasmic chaperone proteinl- |
| STM4575_173_207 | 1.00 | -1.00 | 1256101lSTM4575lputative outer membrane proteinl- |
| STM4576_562_596 | 0.01 | 1.88 | 1256102llplAllipoate-protein ligase Al- |
| STM4577_265_299 | 0.03 | 2.26 | 1256103lsmplhypothetical proteinl- |
| STM4579_704_738 | 0.48 | 1.08 | 1256105lradAlDNA repair protein RadAl+ |
| STM4580.S_538_572 | 0.01 | -1.46 | 1256106lnadRlnicotinamide-nucleotide adenylyltransferasel+ |
| STM4581_1629_1663 | 0.33 | -1.30 | 1256107lyjjKlputative ABC transporter ATP-binding proteinl- |
| STM4582_1851_1885 | 0.06 | -1.35 | 1256108lsltllytic murein transglycosylasel+ |
| STM4583_277_311 | 0.02 | -1.90 | 1256109ltrpRlTrp operon repressorl+ |
| STM4584_209_243 | 0.01 | -1.93 | 1256110lyjjXlNTPasel- |
| STM4585_513_547 | 0.01 | -1.69 | 1256111lgpmBlphosphoglycerate mutasel+ |
| STM4586_801_835 | 0.00 | 2.07 | 1256112lrobltranscriptional regulatorl- |
| STM4587_215_249 | 0.18 | 1.10 | 1256113lcreAlhypothetical proteinl+ |
| STM4588_145_179 | 0.61 | -1.06 | 1256114lcreBlDNA-binding response regulator CreBl+ |
| STM4589_1210_1244 | 0.63 | 1.11 | 1256115lcreClsensory histidine kinase CreCl+ |
| STM4590_863_897 | 0.13 | -1.19 | 1256116lcreDlhypothetical proteinl+ |

| | | | |
|---|---|---|---|
| STM4591_631_665 | 0.51 | 1.11 | 1256117lsthElputative major fimbrial subunitl- |
| STM4592_33_67 | 0.19 | 1.16 | 1256118lsthDlputative fimbrial subunitl- |
| STM4593_1731_1765 | 0.08 | 1.60 | 1256119lsthBlputative fimbrial usher proteinl- |
| STM4594_641_675 | 0.09 | 1.71 | 1256120lsthAlputative fimbrial chaparonel- |
| STM4595_97_131 | 0.03 | 1.43 | 1256121lSTM4595lputative fimbrial chaparonel- |
| STM4596_209_243 | 0.03 | -1.25 | 1256122lSTM4596lputative inner membrane proteinl- |
| STM4597_197_231 | 0.01 | 3.09 | 1256123lSTM4597lputative periplasmic proteinl- |
| STM4598_65_99 | 0.02 | -2.17 | 1256124larcAlDNA-binding response regulator in two-component regulatory system with ArcB or CpxAl- |
| STM4599_49_83 | 0.47 | 1.16 | 1256125lyjjYlputative inner membrane proteinl+ |
| STM4600_257_291 | 0.09 | 1.33 | 1256126llasTlputative tRNA/tRNA methyltransferasel+ |
| PSLT001_2_36 | 0.25 | 1.14 | 1256238lPSLT001lputative cytoplasmic proteinl+ |
| PSLT002_100_134 | 0.69 | 1.06 | 1256237lPSLT002lputative phospholipase Dl+ |
| PSLT003_22_59 | 0.48 | 1.07 | 1256235lrepClDNA replication proteinl+ |
| PSLT004_56_90 | 0.24 | 1.21 | 1256236lrepA3lDNA replication proteinl+ |
| PSLT005_20_55 | 0.24 | -1.11 | 1256234ltaplplasmid replication control proteinl+ |
| PSLT006_627_661 | 0.01 | -1.47 | 1256233lrepAlDNA replication proteinl+ |
| PSLT007_72_106 | 0.09 | 1.45 | 1256232lPSLT007lputative outer membrane proteinl+ |
| PSLT008_338_372 | 0.11 | 1.45 | 1256231lsrgClputative regulatory proteinl- |
| PSLT009_477_511 | 0.24 | 1.41 | 1256230lrcKlresistance to complement killingl- |
| PSLT011_527_561 | 0.10 | 1.20 | 1256229lsrgAlputative thiol-disulfide isomerase or thioredoxinl- |
| PSLT012_54_88 | 0.80 | 1.04 | 1256227lorf7lputative bacterial regulatory proteinl- |
| PSLT013_178_212 | 0.35 | -1.18 | 1256226lpefIlregulatorl- |
| PSLT014_525_559 | 0.14 | 1.16 | 1256224lorf6lputative outer membrane proteinl- |
| PSLT017_1655_1689 | 0.18 | -1.23 | 1256222lpefClusher proteinl- |
| PSLT018_51_85 | 0.11 | -1.41 | 1256221lpefAlmajor fimbrial subunitl- |
| PSLT020_73_107 | 0.02 | 3.44 | 1256220lPSLT020l-l+ |
| PSLT020.1N_129_163 | 0.71 | -1.12 | 1256218lPSLT020.1Nl-l+ |
| PSLT023_813_847 | 0.03 | 1.28 | 1256216lrepA2lDNA replication proteinl- |
| PSLT024_208_246 | 0.51 | 1.26 | 1256217lPSLT024lhypothetical proteinl+ |
| PSLT025_152_189 | 0.06 | 1.27 | 1256215lPSLT025lputative cytoplasmic proteinl- |
| PSLT026_408_443 | 0.02 | 2.35 | 1256213lPSLT026lputative periplasmic proteinl- |
| PSLT027_56_90 | 0.20 | -1.22 | 1256214lccdAlantidotel+ |
| PSLT028_14_48 | 0.05 | 1.55 | 1256209lccdBltoxinl+ |
| PSLT029_154_188 | 0.04 | -1.36 | 1256212lPSLT029lputative cytoplasmic proteinl+ |
| PSLT030_451_485 | 0.16 | 1.18 | 1256211lPSLT030lputative cytoplasmic proteinl+ |

| | | | |
|---|---|---|---|
| PSLT031_371_405 | 0.05 | -1.30 | 1256210\|rsdB\|resolvase\|+ |
| PSLT032_38_73 | 0.17 | 1.25 | 1256207\|PSLT032\|putative diguanylate cyclase/phosphodiesterase\|+ |
| PSLT032.1N_209_243 | 0.02 | 1.41 | 1256208\|PSLT032.1N\|hypothetical protein\|+ |
| PSLT033_4_38 | 0.07 | 1.51 | 1256205\|PSLT033\|putative inner membrane protein\|+ |
| PSLT034_956_991 | 0.42 | 1.14 | 1256206\|PSLT034\|-\|+ |
| PSLT035_239_273 | 0.14 | 1.32 | 1256204\|PSLT035\|-\|- |
| PSLT036_202_236 | 0.01 | 1.92 | 1256203\|PSLT036\|putative transposase\|+ |
| PSLT037_292_327 | 0.10 | 1.50 | 1256202\|spvD\|hydrophilic protein\|- |
| PSLT039_1860_1894 | 0.02 | 1.57 | 1256199\|spvB\|hydrophilic protein\|- |
| PSLT040_3_37 | 0.02 | 3.02 | 1256200\|spvA\|outer membrane protein\|- |
| PSLT040.1N_117_151 | 0.02 | 1.72 | 1256198\|PSLT040.1N\|-\|- |
| PSLT041_831_865 | 0.01 | 1.95 | 1256197\|spvR\|regulator of spv operon\|- |
| PSLT042_857_891 | 0.57 | 1.12 | 1256195\|PSLT042\|putative integrase protein\|+ |
| PSLT043_161_195 | 0.02 | 1.36 | 1256196\|PSLT043\|type II secretion system protein\|- |
| PSLT044_848_882 | 0.01 | 1.53 | 1256194\|rlgA\|putative integrase protein\|- |
| PSLT045_312_346 | 0.10 | 1.33 | 1256192\|PSLT045\|putative resolvase\|- |
| PSLT046_413_447 | 0.01 | 2.36 | 1256193\|PSLT046\|putative carbonic anhydrase\|- |
| PSLT047_335_369 | 0.07 | -1.25 | 1256191\|PSLT047\|putative cytoplasmic protein\|- |
| PSLT048_687_721 | 0.73 | -1.05 | 1256190\|tlpA\|alpha-helical coiled-coil protein\|+ |
| PSLT049_53_87 | 0.29 | 1.32 | 1256189\|PSLT049\|-\|+ |
| PSLT050.1N_39_73 | 0.05 | -1.66 | 1256187\|PSLT050.1N\|hypothetical protein\|+ |
| PSLT050.2N_361_395 | 0.48 | -1.17 | 1256188\|PSLT050.2N\|hypothetical protein\|+ |
| PSLT051_107_141 | 0.71 | 1.03 | 1256185\|PSLT051\|putative cytoplasmic protein\|- |
| PSLT052_577_611 | 0.20 | -1.12 | 1256186\|parA\|plasmid partition protein A\|+ |
| PSLT053_310_344 | 0.08 | -1.72 | 1256184\|parB\|plasmid partition protein B\|+ |
| PSLT054_456_490 | 0.04 | 2.05 | 1256183\|umuC\|DNA polymerase V subunit UmuC\|- |
| PSLT055_259_293 | 0.01 | 1.72 | 1256182\|samA\|SamA\|- |
| PSLT056_403_437 | 0.18 | -1.32 | 1256180\|PSLT056\|putative cytoplasmic protein\|+ |
| PSLT057_247_281 | 0.81 | -1.06 | 1256181\|PSLT057\|putative cytoplasmic protein\|+ |
| PSLT058_145_179 | 0.58 | 1.09 | 1256179\|PSLT058\|-\|+ |
| PSLT059_470_504 | 0.67 | -1.06 | 1256178\|PSLT059\|putative adenine-specific DNA methylase\|+ |
| PSLT060_98_132 | 0.14 | -1.33 | 1256177\|PSLT060\|putative cytoplasmic protein\|+ |
| PSLT060.1N_93_127 | 0.01 | -1.60 | 1256175\|PSLT060.1N\|hypothetical protein\|+ |
| PSLT062_207_241 | 0.02 | 1.32 | 1256173\|PSLT062\|putative cytoplasmic protein\|+ |
| PSLT063_304_338 | 0.13 | 1.41 | 1256174\|PSLT063\|putative cytoplasmic protein\|+ |
| PSLT064_42_76 | 0.20 | -1.08 | 1256172\|PSLT064\|putative inner membrane protein\|+ |

| | | | |
|---|---|---|---|
| PSLT065_493_527 | 0.09 | 1.63 | 1256171lPSLT065l-l- |
| PSLT066_151_185 | 0.31 | 1.12 | 1256170lssbBlsingle-strand DNA binding proteinl+ |
| PSLT067_7_41 | 0.57 | -1.06 | 1256168lPSLT067lputative cytoplasmic proteinl+ |
| PSLT069_372_407 | 0.10 | 1.15 | 1256167lpsiBlPsiBl+ |
| PSLT070_608_643 | 0.04 | 1.44 | 1256166lpsiAlPsiAl+ |
| PSLT071_321_355 | 0.01 | 1.54 | 1256165lPSLT071l-l+ |
| PSLT072_23_57 | 0.28 | -1.20 | 1256164lPSLT072lputative transglycosylasel- |
| PSLT073_111_145 | 0.92 | -1.01 | 1256163ltraMlmating signal proteinl+ |
| PSLT075_180_214 | 0.64 | -1.32 | 1256127ltraJlregulatory proteinl+ |
| PSLT074_58_92 | 0.14 | -1.18 | 1256162lfinPlmisc_RNAl- |
| PSLT077_208_242 | 0.06 | 1.28 | 1256159ltraAlpilus subunitl+ |
| PSLT078_218_252 | 0.19 | -1.26 | 1256160ltraLlpilus assembly proteinl+ |
| PSLT079_447_481 | 0.01 | 2.57 | 1256158ltraElpilus assembly proteinl+ |
| PSLT080_550_584 | 0.61 | 1.13 | 1256157ltraKlpilus assembly proteinl+ |
| PSLT082_388_422 | 0.05 | 1.56 | 1256156ltraPlconjugative transfer proteinl+ |
| PSLT083_104_139 | 0.52 | -1.05 | 1256154ltrbDlconjugative transfer proteinl+ |
| PSLT084_226_260 | 0.03 | 1.40 | 1256153ltraVlpilus assembly proteinl+ |
| PSLT085_34_68 | 0.27 | 1.20 | 1256151ltraRlconjugative transfer proteinl+ |
| PSLT087_96_134 | 0.16 | -1.31 | 1256152lPSLT087lOrfG2l+ |
| PSLT088_2183_2217 | 0.11 | 1.48 | 1256150ltraClATP-binding proteinl+ |
| PSLT089_84_118 | 0.01 | 1.46 | 1256149ltrbIlpilus assembly proteinl+ |
| PSLT091_285_319 | 0.02 | -1.25 | 1256147ltraWlpilus assembly proteinl+ |
| PSLT092_415_449 | 0.97 | 1.01 | 1256148ltraUlpilus assembly proteinl+ |
| PSLT093_64_99 | 0.61 | -1.10 | 1256146lPSLT093lOrfFl+ |
| PSLT094_451_485 | 0.02 | -1.86 | 1256145ltrbClpilus assembly proteinl+ |
| PSLT094.1N_317_351 | 0.31 | 1.09 | 1256143lPSLT094.1Nlhypothetical proteinl+ |
| PSLT095_1553_1587 | 0.40 | 1.10 | 1256144ltraNlmating pair stabilization proteinl+ |
| PSLT096_63_97 | 0.02 | -1.46 | 1256142ltrbElconjugative transfer proteinl+ |
| PSLT097_322_356 | 0.02 | -1.25 | 1256140ltraFlpilus assembly proteinl+ |
| PSLT098_128_162 | 0.20 | -1.21 | 1256141ltraQlpilin chaperonel+ |
| PSLT099_59_93 | 0.06 | -1.24 | 1256139ltrbBlconjugative transfer proteinl+ |
| PSLT100_422_456 | 0.58 | -1.07 | 1256138ltraHlpilus assembly proteinl+ |
| PSLT101_2108_2142 | 0.11 | -1.11 | 1256136ltraGlmating pair stabilization and pilus assembly proteinl+ |
| PSLT102_436_470 | 0.05 | -1.44 | 1256137ltraSlentry exclusion proteinl+ |
| PSLT103_496_530 | 0.22 | -1.21 | 1256135ltraTlsurface exclusion proteinl+ |
| PSLT104_1579_1613 | 0.05 | -1.10 | 1256134ltraDlATP-binding protein/DNA transporterl+ |

| | | | |
|---|---|---|---|
| PSLT105_444_478 | 0.22 | 1.18 | 1256132\|trbH\|conjugative transfer protein\|+ |
| PSLT106_88_122 | 0.03 | 1.74 | 1256133\|PSLT106\|MvpA-like protein\|- |
| PSLT107_168_202 | 0.01 | 1.99 | 1256131\|PSLT107\|putative cytoplasmic protein\|- |
| PSLT108_5003_5038 | 0.56 | 1.06 | 1256130\|tralloriT nickase/helicase\|+ |
| PSLT110_6_40 | 0.23 | 1.14 | 1256128\|traX\|pilin subunit acetylation\|+ |
| PSLT111_431_465 | 0.04 | 1.36 | 1256129\|finO\|finP binding-protein\|+ |
| SLP1_0001_191_225 | 0.01 | 1.58 | \|\|\|- |
| SLP1_0002_179_213 | 0.45 | 1.07 | \|\|\|- |
| SLP1_0003_4387_4422 | 0.13 | -1.18 | \|\|\|- |
| SLP1_0004_428_462 | 0.05 | -1.37 | \|\|\|- |
| SLP1_0005_168_202 | 0.06 | 1.36 | \|\|\|+ |
| SLP1_0006_188_222 | 0.03 | 1.33 | \|\|\|+ |
| SLP1_0007_1378_1412 | 0.98 | -1.00 | \|\|\|- |
| SLP1_0008_496_530 | 0.06 | -1.33 | \|\|\|- |
| SLP1_0009_438_472 | 0.13 | -1.20 | \|\|\|- |
| SLP1_0010_2530_2564 | 0.66 | 1.16 | \|\|\|- |
| SLP1_0011_1326_1360 | 0.33 | -1.18 | \|\|\|- |
| SLP1_0012_175_209 | 0.04 | 1.21 | \|\|\|- |
| SLP1_0013_128_162 | 0.24 | -1.25 | \|\|\|- |
| SLP1_0014_615_649 | 0.96 | -1.00 | \|\|\|- |
| SLP1_0015_63_97 | 0.01 | -1.53 | \|\|\|- |
| SLP1_0016_1553_1587 | 0.37 | 1.15 | \|\|\|- |
| SLP1_0017_451_485 | 0.01 | -1.97 | \|\|\|- |
| SLP1_0018_72_107 | 0.30 | 1.17 | \|\|\|- |
| SLP1_0020_331_365 | 0.05 | -1.18 | \|\|\|- |
| SLP1_0022_1765_1799 | 0.05 | 1.25 | \|\|\|- |
| SLP1_0023_96_134 | 0.62 | 1.12 | \|\|\|- |
| SLP1_0024_34_68 | 0.06 | 1.71 | \|\|\|- |
| SLP1_0025_226_260 | 0.05 | 1.31 | \|\|\|- |
| SLP1_0026_104_139 | 0.05 | 1.25 | \|\|\|- |
| SLP1_0029_558_592 | 0.15 | 2.02 | \|\|\|- |
| SLP1_0030_143_177 | 0.03 | -1.59 | \|\|\|- |
| SLP1_0031_276_310 | 0.93 | 1.05 | \|\|\|- |
| SLP1_0032_208_242 | 0.07 | 1.25 | \|\|\|- |
| SLP1_0033_156_195 | 0.00 | 2.81 | \|\|\|- |
| SLP1_0034_176_215 | 0.09 | 1.26 | \|\|\|- |

| | | | |
|---|---|---|---|
| SLP1_0035_111_145 | 0.92 | 1.02 | III- |
| SLP1_0036_391_425 | 0.42 | 1.27 | III+ |
| SLP1_0037_612_646 | 0.07 | 1.22 | III- |
| SLP1_0038_372_407 | 0.41 | -1.04 | III- |
| SLP1_0040_71_105 | 0.16 | 1.13 | III- |
| SLP1_0041_475_509 | 0.27 | -1.15 | III- |
| SLP1_0042_34_68 | 0.04 | 1.16 | III- |
| SLP1_0043_306_340 | 0.07 | 1.56 | III- |
| SLP1_0050_71_105 | 0.15 | -1.12 | III- |
| SLP1_0051_403_437 | 0.18 | -1.30 | III- |
| SLP1_0052_119_153 | 0.02 | 1.52 | III+ |
| SLP1_0053_776_810 | 0.02 | 1.79 | III+ |
| SLP1_0054_434_468 | 0.23 | -1.43 | III- |
| SLP1_0055_579_613 | 0.31 | -1.14 | III- |
| SLP1_0056_107_141 | 0.67 | -1.03 | III+ |
| SLP1_0057_52_86 | 0.07 | -1.20 | III+ |
| SLP1_0058_40_74 | 0.38 | 1.18 | III- |
| SLP1_0059_119_153 | 0.49 | -1.18 | III- |
| SLP1_0060_335_369 | 0.35 | 1.08 | III+ |
| SLP1_0061_85_119 | 0.00 | 3.45 | III+ |
| SLP1_0062_312_346 | 0.09 | 1.24 | III+ |
| SLP1_0063_848_882 | 0.02 | 1.64 | III+ |
| SLP1_0064_161_195 | 0.04 | 1.56 | III+ |
| SLP1_0065_79_113 | 0.01 | 1.54 | III- |
| SLP1_0066_447_481 | 0.04 | 2.17 | III+ |
| SLP1_0067_413_447 | 0.00 | 5.45 | III+ |
| SLP1_0069_295_329 | 0.04 | 2.42 | III+ |
| SLP1_0070_292_327 | 0.06 | 1.68 | III+ |
| SLP1_0071_206_240 | 0.01 | 1.85 | III- |
| SLP1_0072_231_265 | 0.70 | 1.05 | III+ |
| SLP1_0073_496_531 | 0.94 | -1.02 | III- |
| SLP1_0074_72_106 | 0.32 | 1.13 | III- |
| SLP1_0075_38_73 | 0.88 | 1.03 | III- |
| SLP1_0076_371_405 | 0.01 | -1.37 | III- |
| SLP1_0077_455_489 | 0.06 | 1.32 | III- |
| SLP1_0078_166_200 | 0.02 | -1.47 | III- |

| | | | |
|---|---|---|---|
| SLP1_0079_14_48 | 0.07 | 1.52 | III- |
| SLP1_0080_56_90 | 0.03 | -1.58 | III- |
| SLP1_0081_408_443 | 0.01 | 2.79 | III+ |
| SLP1_0082_240_277 | 0.04 | 2.22 | III+ |
| SLP1_0083_208_246 | 0.50 | 1.20 | III- |
| SLP1_0084_813_847 | 0.04 | 1.30 | III+ |
| SLP1_0085_8_42 | 0.12 | 1.30 | III- |
| SLP1_0086_192_227 | 0.50 | -1.07 | III+ |
| SLP1_0087_455_489 | 0.03 | -1.65 | III+ |
| SLP1_0088_1908_1942 | 0.62 | 1.08 | III+ |
| SLP1_0090_75_109 | 0.25 | -1.13 | III+ |
| SLP1_0091_693_727 | 0.70 | 1.05 | III+ |
| SLP1_0092_102_136 | 0.36 | -1.10 | III+ |
| SLP1_0093_50_84 | 0.22 | -1.22 | III+ |
| SLP1_0094_527_561 | 0.04 | 1.29 | III+ |
| SLP1_0096_295_329 | 0.45 | 1.27 | III+ |
| SLP1_0097_418_452 | 0.16 | 1.62 | III+ |
| SLP1_0098_72_106 | 0.05 | 1.75 | III- |
| SLP1_0099_627_661 | 0.00 | -1.40 | III- |
| SLP1_0100_24_59 | 0.91 | -1.01 | III- |
| SLP1_0101_56_90 | 0.95 | -1.01 | III- |
| SLP1_0102_8_47 | 0.01 | 1.74 | III- |
| SLP1_0103_100_134 | 0.92 | -1.01 | III- |
| SLP1_0104_239_273 | 0.34 | 1.10 | III- |
| SLP2_0001_33_67 | 0.45 | 1.10 | III+ |
| SLP2_0002_177_211 | 0.22 | 1.15 | III+ |
| SLP2_0003_2_40 | 0.26 | -1.18 | III+ |
| SLP2_0004_81_115 | 0.72 | -1.03 | III+ |
| SLP2_0005_53_87 | 0.28 | -1.19 | III+ |
| SLP2_0006_225_259 | 0.02 | 1.79 | III+ |
| SLP2_0007_105_139 | 0.00 | 2.79 | III+ |
| SLP2_0008_225_259 | 0.01 | 2.06 | III- |
| SLP2_0009_243_279 | 0.01 | 1.97 | III- |
| SLP2_0010_375_409 | 0.07 | 1.30 | III- |
| SLP2_0011_1248_1282 | 0.79 | 1.02 | III+ |
| SLP2_0012_1106_1140 | 0.07 | 1.47 | III+ |

| | | | |
|---|---|---|---|
| SLP2_0014_101_135 | 0.79 | 1.07 | III+ |
| SLP2_0016_53_87 | 0.91 | 1.02 | III+ |
| SLP2_0017_81_115 | 0.15 | 1.19 | III+ |
| SLP2_0019_213_247 | 0.19 | -1.22 | III+ |
| SLP2_0020_69_103 | 0.15 | -1.18 | III+ |
| SLP2_0021_161_195 | 0.06 | -1.34 | III+ |
| SLP2_0022_205_239 | 0.03 | 1.53 | III+ |
| SLP2_0023_85_120 | 0.09 | 1.43 | III- |
| SLP2_0024_9_43 | 0.10 | 1.21 | III+ |
| SLP2_0025_577_611 | 0.60 | -1.05 | III+ |
| SLP2_0026_180_214 | 0.09 | -1.32 | III+ |
| SLP2_0027_17_51 | 0.06 | 1.94 | III+ |
| SLP2_0028_49_83 | 0.45 | -1.10 | III+ |
| SLP2_0029_209_243 | 0.52 | -1.13 | III+ |
| SLP2_0031_403_437 | 0.07 | -1.62 | III+ |
| SLP2_0032_261_295 | 0.67 | 1.14 | III+ |
| SLP2_0033_173_207 | 0.99 | 1.00 | III+ |
| SLP2_0034_14_48 | 0.03 | -1.28 | III+ |
| SLP2_0035_3_37 | 0.26 | -1.25 | III+ |
| SLP2_0036_1274_1308 | 0.07 | 1.21 | III+ |
| SLP2_0038_453_488 | 0.57 | -1.07 | III+ |
| SLP2_0039_9_43 | 0.88 | 1.02 | III+ |
| SLP2_0040_372_406 | 0.73 | 1.03 | III+ |
| SLP2_0041_282_317 | 0.62 | -1.07 | III+ |
| SLP2_0042_213_247 | 0.03 | -2.45 | III+ |
| SLP2_0043_17_51 | 0.68 | 1.03 | III+ |
| SLP2_0044_587_621 | 0.09 | -1.29 | III+ |
| SLP2_0045_193_227 | 0.33 | -1.11 | III+ |
| SLP2_0047_193_227 | 0.02 | 1.62 | III+ |
| SLP2_oriT_41_75 | 0.25 | -1.15 | III+ |
| SLP2_0048_257_291 | 0.02 | 2.13 | III+ |
| SLP2_0049_1861_1895 | 0.02 | -1.62 | III+ |
| SLP2_0050_2061_2095 | 0.00 | 3.22 | III- |
| SLP2_0053_89_123 | 0.37 | -1.16 | III+ |
| SLP2_0054_92_126 | 0.08 | 1.51 | III+ |
| SLP2_0055_553_587 | 0.02 | 1.84 | III- |

| | | | |
|---|---|---|---|
| SLP2_0056_1313_1347 | 0.02 | 1.54 | III- |
| SLP2_0058_1020_1054 | 0.04 | 2.41 | III- |
| SLP2_0059_321_355 | 0.06 | 1.51 | III- |
| SLP2_0060_2558_2592 | 0.03 | 1.28 | III- |
| SLP2_0061_337_371 | 0.47 | -1.26 | III- |
| SLP2_0062_137_171 | 0.10 | 1.89 | III- |
| SLP2_0063_129_163 | 0.02 | 2.28 | III- |
| SLP2_0065_113_147 | 0.09 | 2.11 | III- |
| SLP2_0066_451_485 | 0.01 | 1.85 | III- |
| SLP2_0067_545_579 | 0.55 | 1.17 | III- |
| SLP2_0068_533_567 | 0.07 | 1.51 | III- |
| SLP2_0069_97_131 | 0.05 | 2.31 | III- |
| SLP2_0072_481_515 | 0.09 | 1.70 | III- |
| SLP2_0073_243_277 | 0.07 | 2.38 | III- |
| SLP2_0074_246_280 | 0.73 | 1.05 | III- |
| SLP2_0076_401_435 | 0.02 | 1.91 | III- |
| SLP2_0077_529_563 | 0.88 | -1.02 | III- |
| SLP2_0079_449_483 | 0.03 | 3.02 | III- |
| SLP2_0081_113_147 | 0.14 | -1.16 | III+ |
| SLP2_0082_145_179 | 0.17 | -1.29 | III- |
| SLP2_0083_129_163 | 0.93 | -1.01 | III+ |
| SLP2_0085_1219_1253 | 0.05 | -1.28 | III- |
| SLP2_0086_457_491 | 0.02 | 1.99 | III- |
| SLP2_0088_541_575 | 0.26 | -1.20 | III- |
| SLP2_0089_323_357 | 0.04 | 3.45 | III- |
| SLP2_0090_555_589 | 0.00 | 2.04 | III- |
| SLP2_0091_321_355 | 0.28 | -1.19 | III- |
| SLP2_0094_97_131 | 0.04 | 1.49 | III- |
| SLP2_0096_273_307 | 0.14 | 1.31 | III- |
| SLP2_0097_331_370 | 0.03 | 3.22 | III- |
| SLP2_0098_129_163 | 0.07 | 2.31 | III- |
| SLP2_0100_217_252 | 0.54 | 1.13 | III- |
| SLP2_0101_385_419 | 0.04 | 1.30 | III- |
| SLP2_0102_225_259 | 0.04 | 1.43 | III- |
| SLP2_0103_145_179 | 0.07 | 2.09 | III- |
| SLP3_0001_257_291 | 0.33 | -1.23 | III- |

| | | | |
|---|---|---|---|
| SLP3_0002_129_163 | 0.06 | 1.43 | lll- |
| SLP3_0003_673_707 | 0.51 | 1.08 | lll- |
| SLP3_0004_609_643 | 0.21 | -1.18 | lll- |
| SLP3_0005_3_37 | 0.01 | 2.97 | lll- |
| SLP3_0006_137_171 | 0.02 | 1.80 | lll- |
| SLP3_0008_1459_1493 | 0.33 | 1.14 | lll- |
| SLP3_0007_840_874 | 0.05 | 1.81 | lll- |
| SLP3_0009_282_317 | 0.01 | 1.34 | lll- |
| SLP3_0010_355_389 | 0.01 | 1.92 | lll- |
| SLP3_0011_48_82 | 0.30 | 1.44 | lll+ |
| SLP3_0012_65_99 | 0.70 | -1.06 | lll+ |
| SLP3_0013_209_243 | 0.62 | -1.05 | lll- |
| SLP3_0014_417_451 | 0.09 | 1.14 | lll- |
| lytB STM0050_41_75 | 0.29 | 1.16 | lSTnc30ll+ |
| yabN/leuD_161_196 | 0.09 | 1.83 | lsgrSll+ |
| secA mutT_5_39 | 0.02 | 1.95 | lSTnc40ll+ |
| lpdA STM0155_11_45 | 0.10 | 2.12 | lSTnc50ll- |
| STM0294.1n / STM0295_204 | 0.16 | -1.34 | lisrAll+ |
| ybaK/ybaP_29_63 | 0.12 | 1.35 | lsroBll+ |
| dsbG ahpC_13_47 | 0.11 | -1.19 | lSTnc70ll+ |
| gltJ/gltI_33_67 | 0.07 | -1.43 | lsroCll- |
| STM0869/STM0870_5_39 | 0.95 | 1.06 | lrybBll- |
| STM0904 STM0905_88_122 | 0.38 | -1.21 | lSTnc100ll+ |
| serS dmsA_63_101 | 0.02 | 2.93 | lSTnc130ll- |
| icdA STM1239_158_193 | 0.24 | 1.59 | lSTnc150ll- |
| envF / msgA_233_267 | 0.86 | 1.03 | lisrCll+ |
| STM1273 / STM1274_22_56 | 0.41 | -1.75 | lisrEll- |
| STM1273/yeaQ_36_70 | 0.57 | -1.46 | lryhB-2ll- |
| ydiL/ydiK_60_94 | 0.18 | 1.63 | lrprAll- |
| ydiH/STM1368_10_44 | 0.01 | 1.78 | lrydBll+ |
| STM1528 STM1530_138_17 | 0.03 | 1.82 | lSTnc170ll- |
| acnA cysB_145_179 | 0.04 | -1.77 | lSTnc180ll- |
| STM1841 kdgR_45_84 | 0.02 | 2.40 | lSTnc190ll+ |
| STM1871/STM1872_21_55 | 0.62 | 1.47 | lryeBll- |
| edd zwf_2_36 | 0.28 | -1.12 | lSTnc200ll- |
| yecA STM1939_63_97 | 0.00 | 2.09 | lSTnc210ll+ |

| | | | |
|---|---|---|---|
| STM1994/ompS_17_54 | 0.01 | 3.22 | lrseX ll+ |
| ompS cspB_5_39 | 0.05 | 2.20 | lSTnc220ll- |
| yegD/STM2126_77_116 | 0.60 | 1.26 | lryeCll+ |
| yegQ/STM2137_17_51 | 0.02 | -13.14 | lcyaRll+ |
| STM2243 / STM2244_213_2 | 0.02 | 1.90 | lisrGll+ |
| ompC/yojN_40_76 | 0.29 | 1.97 | lmicF ll+ |
| glpC / STM2287_72_106 | 0.13 | -1.17 | lisrH-2 ll- |
| STM2287 /glpC_200_234 | 0.33 | 1.10 | lisrH-1ll- |
| acrD yffB_29_63 | 0.01 | -9.05 | lSTnc250ll- |
| STM2534/sseB_209_243 | 0.21 | 1.34 | lryfAll+ |
| yfhK/purG_33_67 | 0.36 | 1.38 | lglmY ll- |
| STM2614 / STM2616_33_67 | 0.04 | 1.89 | lisrIll- |
| STM2616 / STM2614_2_39 | 0.15 | 1.57 | lisrJll- |
| smpB / STM2690_178_212 | 0.07 | -1.33 | lisrLll- |
| STM2816 luxS_68_105 | 0.46 | 1.22 | lSTnc260ll+ |
| invH STM2901_16_54 | 0.95 | 1.01 | lSTnc270ll+ |
| invH/STM2901_8_46 | 0.59 | 1.06 | linvRll+ |
| yqcC/syd_311_345 | 0.03 | -4.07 | lcsrBll- |
| gcvA/ygdI_155_189 | 0.05 | -1.50 | lgcvBll+ |
| kduI yqeF_9_43 | 0.94 | -1.04 | lSTnc280ll+ |
| STM3038 / STM3039_65_99 | 0.58 | -1.09 | lisrOll+ |
| ygfE/ygfA_93_127 | 0.43 | 1.30 | lssrSll+ |
| ygfA/serA_57_91 | 0.60 | 1.31 | lrygC ll+ |
| STM3123 STM3124_109_14 | 0.66 | 1.32 | lSTnc300ll- |
| yqiK/rfaE_98_136 | 0.73 | 1.23 | lrygD ll- |
| greA dacB_3_37 | 0.04 | 1.84 | lSTnc330ll- |
| yhbL/arcB_17_51 | 0.06 | -1.88 | lsraH ll+ |
| tnpA_5 yhfL_64_103 | 0.02 | 1.43 | lSTnc340ll- |
| yhhX/yhhY_25_59 | 0.83 | -1.07 | lryhB-1ll- |
| yhjB yhjC_73_107 | 0.06 | 2.75 | lSTnc360ll+ |
| STM3691 lldP_3_38 | 0.17 | -1.31 | lSTnc380ll- |
| yibD tdh_12_46 | 0.04 | -1.66 | lSTnc390ll- |
| ilvB/emrD_49_83 | 0.14 | 1.22 | listR ll- |
| STM3844 STM3845_25_59 | 0.12 | 1.59 | lSTnc400ll+ |
| glmU STM3863_89_123 | 0.05 | 3.24 | lSTnc410ll+ |
| yihA/yihI_158_192 | 1.00 | -1.00 | lcsrCll+ |

| | | | |
|---|---|---|---|
| STM4097 / STM4098_65_99 | 0.35 | 1.11 | lisrPll+ |
| argH/oxyR_73_107 | 0.50 | 1.18 | loxySll- |
| pgi yjbE_87_121 | 0.03 | 1.98 | lSTnc430ll- |
| soxR/STM4267_65_99 | 0.37 | 2.98 | lsraLll- |
| STM4310 tnpA_6_11_46 | 0.14 | 5.19 | lSTnc440ll+ |
| STM4503 STM4504_85_119 | 0.00 | 1.88 | lSTnc460ll- |
