# Supplementary material for: Programmed Delay of a Virulence Circuit Promotes Salmonella Pathogenicity
Source: mBio. 2019 Apr 9;10(2):e00291-19. doi: 10.1128/mBio.00291-19 (PMC6456747; doi:10.1128/mBio.00291-19)
Supplement: FIG S5 [file mBio.00291-19-sf005.pdf]

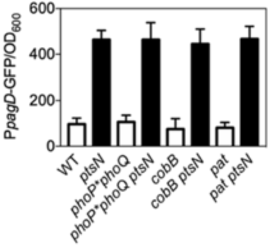

Fig. S5. EIIA^{Ntr} controls the PhoP-activated *pagD* gene expression independent of PhoQ and acetylation of PhoP. Fluorescence were determined from wild-type, *ptsN*, *phoP*phoQ*, *phoP*phoQ ptsN*, *cobB*, *cobB ptsN* , *pat*, and *pat ptsN Salmonella* harboring a plasmid with p_{pagD}-*gfp* fusion. Bacteria were grown M9 medium at acidic. The mean and SD from three independent experiments are shown.
