# Supplementary material for: Programmed Delay of a Virulence Circuit Promotes Salmonella Pathogenicity
Source: mBio. 2019 Apr 9;10(2):e00291-19. doi: 10.1128/mBio.00291-19 (PMC6456747; doi:10.1128/mBio.00291-19)
Supplement: FIG S6 [file mBio.00291-19-sf006.pdf]

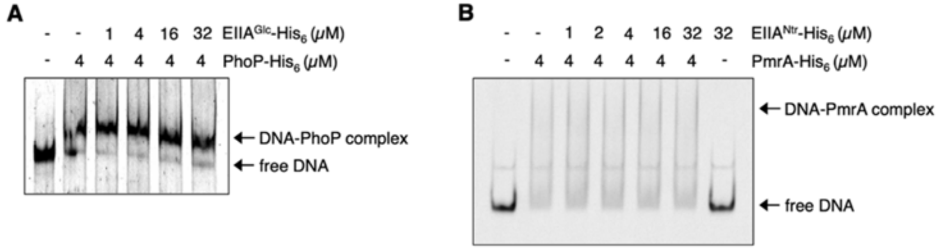

Fig. S6. Regulatory effects of EIIA^{Ntr} on PhoP is specific. (**A**) *In vitro* binding of PhoP to the *pagD* promoter with or without EIIA^{Glc}. The *pagD* promoter DNA (80 fmol) was incubated with PhoP (4 μM) and EIIA^{Glc} (1, 4, 16 and 32 μM) proteins. (**B**) *In vitro* binding of PmrA to the *pbgP* promoter with or without EIIA^{Ntr}. The *pbgP* promoter DNA (80 fmol) was incubated with PmrA (4 μM) and EIIA^{Ntr} (1, ,2 4, 16 and 32 μM) proteins. Representatives of at least three independent experiments are shown.
