# Supplementary material for: Programmed Delay of a Virulence Circuit Promotes Salmonella Pathogenicity
Source: mBio. 2019 Apr 9;10(2):e00291-19. doi: 10.1128/mBio.00291-19 (PMC6456747; doi:10.1128/mBio.00291-19)
Supplement: FIG S7 [file mBio.00291-19-sf007.pdf]

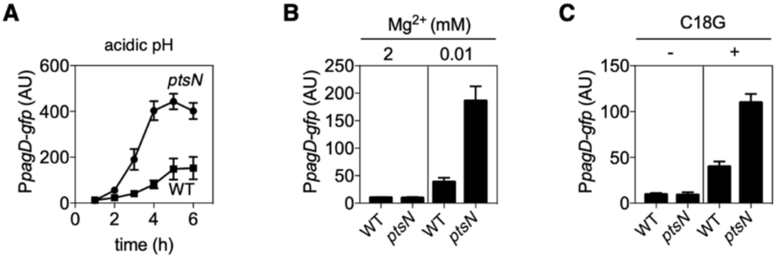

Fig. S7. EIIA^{Ntr} inhibits expression of the *pagD* gene in PhoQ inducing conditions. (**A**) *Salmonella* strains (wild-type and the *ptsN* mutant) harboring a plasmid containing a p_{pagD}-*gfp* fusion were grown in acidified M9 medium. Fluorescence was measured at the indicated time points after inoculation. (**B-C**) Fluorescence was measured from *Salmonella* strains (wild-type and the *ptsN* mutant) harboring a plasmid containing a p_{pagD}-*gfp* fusion were grown in (A) M9 medium with high or low Mg^{2+} (2 mM or 10 μM), or (B) M9 medium with or without of 5 μg/ml of an antimicrobial peptide C18G. The mean and SD from three independent experiments are shown.
