# Supplementary material for: Programmed Delay of a Virulence Circuit Promotes Salmonella Pathogenicity
Source: mBio. 2019 Apr 9;10(2):e00291-19. doi: 10.1128/mBio.00291-19 (PMC6456747; doi:10.1128/mBio.00291-19)
Supplement: TABLE S2 [file mBio.00291-19-st002.pdf]

Table S2. Bacterial strains, plasmids, and primers used in this study.

(A) Bacterial strains and plasmids used in this study.

Strain or plasmid	Description	Reference
Salmonella enterica		
SL1344	wild-type, Str^R	(1)
SR3203	*ΔptsN*	(2)
SR3202	*ΔptsP*::Cm^R	This work
SR3278	p_{pagD}::*lacZ* (Km^R)	This work
SR3280	p_{pagD}::*lacZ* (Km^R) *ΔptsN*	This work
SR4014	*ptsN-FLAG*::Km^R	This work
SR4045	*ptsN-FLAG*	This work
SR4145	P*ptsN*::*lacZ* (Km^R)	This work
SR4401	*ΔphoP*::Km^R	This work
SR4402	*ΔphoQ*::Km^R	This work
SR4405	*ΔphoP*	This work
SR4412	*ΔphoP ΔptsN*::Km^R	This work
SR4413	*ΔphoP ΔptsN*	This work
SR4417	*ptsN-FLAG ΔphoP*	This work
SR4420	*ptsN-FLAG ΔphoP*::Km^R	This work

SR4421	*ptsN-FLAG ΔphoQ*::Km^R	This work
SR4428	p_{ptsN}::*lacZ* (Km^R) *phoP*::Tn*10*	This work
SR4430	p_{pagD}::*lacZ* (Km^R) *phoP*::Tn*10*	This work
SR4445	*ptsN-FLAG Δlon*::Cm^R	This work
SR4449	*ptsN-FLAG Δlon*	This work
SR4450	*ptsN-FLAG Δlon ΔphoP*	This work
SR4451	*ptsN-FLAG Δlon ΔphoP*::Kan	This work
SR4452	*ptsN-FLAG ΔclpXP*::Cm^R	This work
SR4458	*ptsN-FLAG ΔptsP*::Cm^R	This work
SR4459	*ptsN-FLAG ΔphoP ΔptsP*::Cm^R	This work
SR4939	p_{pagD}::*lacZ* (Km^R) *ΔptsN phoP*::Tn*10*	This work
SR7081	*phoP**phoQ*::Tn*10*	This work
SR7082	*phoP**phoQ*::Tn*10 ΔptsN*	This work
SR7083	*ΔcobB*::Km^R	This work
SR7084	*ΔcobB*::Km^R *ΔptsN*	This work
SR7085	*Δpat*::Km^R	This work
SR7086	*Δpat*::Km^R *ΔptsN*	This work
Escherichia coli		
DH5α	F–*sup*E44 Δ*lac*U169 (*f* 80 *lac*ZΔM15) *hsd*R17	(3)

	*rec*A1 *end*A1 *gyr*A96 *thi*-1 *rel*A1	
BTH101	F^{-} *cya*-99 *araD*139 *galE*15 *galK*16 *rpsL*1 (Str^{R})	(4)
	hsdR2 mcrA1 mcrB1	
**Plasmids**		
pCP20	rep$_{pSC101}$ts Ap^{R} Cm^{R} *FLP*$^{+}$ *c*I857^{+}	(5)
pCE70	rep$_{R6K}$ Km^{R} Cm^{R} FRT *tnpR lacZY*	(6)
pKD3	rep$_{R6K}$ Ap^{R} FRT Cm^{R} FRT	(5)
pKD13	rep$_{R6K}$ Ap^{R} FRT Km^{R} FRT	(5)
pKD46	rep$_{pSC101}$ts Ap^{R} p$_{araBAD}$ γ β exo	(5)
pRS415	rep$_{pMB1}$ Ap^{R} *lacZ*	(7)
pJJ18	rep$_{pMB1}$ Ap^{R} P*ptsN*::*lacZ*	This work
pJJ19	rep$_{pMB1}$ Ap^{R} P*rpoN*::*lacZ*	This work
pACYC184	rep$_{p15A}$ Cm^{R} Tet^{R}	(8)
pJJ11	rep$_{p15A}$ Cm^{R} *ptsN*	(2)
pJJ12	rep$_{p15A}$ Cm^{R} *ptsN*(H73A)	(2)
pUHE21-2*lacI*^{q}	rep$_{pMB1}$ Ap^{R} *lacI*^{q}	(8)
pJJ14	rep$_{pMB1}$ Ap^{R} *lacI*^{q}*ptsN*	(2)
pJJ15	rep$_{pMB1}$ Ap^{R} *lacI*^{q}*ptsN* (H73A)	This work
pPtsN(H73E)	rep$_{pMB1}$ Ap^{R} *lacI*^{q}*ptsN* (H73E)	This work

pJJ37	rep_{pMB1} Ap^R *lacI*^{q} *ptsN-His6*	(2)
pPhoP-H6	rep_{pMB1} Ap^R *lacI*^{q} *phoP-His6*	This work
pHJ1	rep_{pMB1} Ap^R *lacI*^{q} *phoP*	This work
pHJ2	rep_{pMB1} Ap^R *lacI*^{q} *phoQ*	This work
pHJ3	rep_{pMB1} Ap^R *lacI*^{q} *ptsN-FLAG*	This work
pPmrA-H6	rep_{pMB1} Ap^R *lacI*^{q} *pmrA-His6*	This work
pFPV25	rep_{pColE1} Ap^R promoterless *gfp*	(9)
pWJ18	rep_{pColE1} Ap^R P*pagD-gfp*	This work
pKT25	Km^R rep_{p15A}	(10)
pUT18	Ap^R rep_{pMB1}	(10)
pT25-ptsN	Km^R rep_{p15A} *ptsN*	(2)
pT25-ptsN(H73A)	Km^R rep_{p15A} *ptsN*(H73A)	(2)
pT25-zip	Km^R rep_{p15A} *zip*	(10)
pT18-phoP	Ap^R rep_{pMB1} *phoP*	This work
pT18-zip	Ap^R rep_{pMB1} *zip*	(10)

(B) Primers used in this study.

**Primers**	**Sequence (from 5' to 3')**
**for qRT-PCR**	

RT-pmrD-F	GGT TAA GAA ATC GCA TTA TGT CAA AA
RT-pmrD-R	CGA ACC GCC GCT ATC G
RT-pagD-F	ACA TCA TGC TTT TAT GCT TTG GTC
RT-pagD-R	AAA CCA GAA CAA TGG CCT GAA
RT-mig-14-F	CGC AAT ACG GCG GTA GTA TCA
RT-mig14-R	ATG CCA GTT ATA GCG CTT CAT G
RT-mgtA-F	TTC AGG GTC CAT GTC GCC
RT-mgtA-R	CCA CAA AAC TTA TGG ATT TAT GCG T
RT-rrs-F	CCA CAA AAC TTA TGG ATT TAT GCG T
RT-rrs-R	TTT ACG CCC AGT AAT TCC GAT T

for chromosomal mutations

ptsN-FLAG-F	TCA AAT CAT TAC TGA CAC CGA AGG TGA GCA GAA TGA GGC AGA CTA CAA GGA CGA CGA TGA CAA GTG ATG TAG GCT GGA GCT GCT TCG
ptsN-FLAG-R	TCT CCT CAC AAC GAC AGA AAT AAA TGC CAT TGA GTT GTT AAT TCC GGG GAT CCG TCG ACC
ptsP-Red-F	ATG CTC ACT CGC CTG CGC GAA ATA GTC GAA AAA GTG GCC ATG TAG GCT GGA GCT GCT TCG
ptsP-Red-R	TTA CAA CCC TCC TCG AAT CAA TCC CCC CAT CCC

	GCG GCG CAT ATG AAT ATC CTC CTT AGT TC
phoP-Red-F	AAC GCT AGA CTG TTC TTA TTG TTA ACA CAA GGG
	AGA AGA GTG TAG GCT GGA GCT GCT TCG
phoP-Red-R	GCT ATT ACG GCG CAT TAA CGC CTG CAT ACG CGC
	CAT TAC CAT TCC GGG GAT CCG TCG ACC
clpX-Red-F	AGT ACA GCA GAT TTT TTC AAT TTT TAT CCA GGA
	GAC GGA ATG TAG GCT GGA GCT GCT TCG
clpP-Red-R2	GCA AAC GCG CCG CCG CAG ATA AAC AGA ATC TTA
	GAG GTA TAT ATG AAT ATC CTC CTT AGT TC
lon-Red-F	ATC TGA TTA CCT GGC GGA CAC TAA ACT AAG AGA
	GAG CTC TTG TAG GCT GGA GCT GCT TCG
lon-Red-R	CAA GCG TCA GAA CTT CCT CAA TGC GCT TCA CAG
	GAT GAA TAT ATG AAT ATC CTC CTT AGT TC
pagD-1	TCT ACG ATT TTG GTA GTA AAA CCC CGC AAC CAC
	CTA CAA ATG TAG GCT GGA GCT GCT TCG
pagD-2	ATG GGT TTT GTC GTC GGG CAG GAC GGT GAA CTA
	ATC TGC CAT TCC GGG GAT CCG TCG ACC
cobB-P1-F-kan	TTT TTT ACA TCT TAC CGA CTA ATC AAA AAA AGA
	GGT TGT TTG TAG GCT GGA GCT GCT TC
cobB-P4-R-kan	TGA AAT GTA GGC CGG ATA AGG CGT TAC CGG GCA

	AAC AGC ACT GTC AAA CAT GAG AAT TAA
pat-P1-F-kan	TTT AAA ATT ATC CGG TCA CTT CTG TGT AAG GGA AAC CGG TTG TAG GCT GGA GCT GCT TC
pat-P4-R-kan	TCA GTA CCC GTT AAA GTG GTC AAC ATT TCC AGT ACA TTA CCT GTC AAA CAT GAG AAT TAA
phoQ-Red-F	CCA CCG TAC GCG GAC AAG GAT ATC TTT TTG AAT TGC GCT ATG TAG GCT GGA GCT GCT TCG
phoQ-Red-R	GCT CGC CGA CAA AAC TGA TTT CTG GTG AAA TAT CCA TAC TAT TCC GGG GAT CCG TCG ACC

for plasmid construction

ptsN-pF2	CAG GTT CTG AAT TCA AAT TAT GA
ptsN-pR2	GTA CCA GGA TCC GTT TCT C
ptsN-pF4	CCT GCG TGA ATT CGT GAC G
ptsN-pR4	TGG TTA AGG ATC CTG CTC AG
ptsN_H73A-F	AGT ACC GGT ATT GGT AAG CGT ATC GCC ATC CCG CAC
ptsN_H73A-R	GTG CGG GAT GGC GAT ACG CTT ACC AAT ACC GGT ACT
ptsN_H73E-F	GGT ATC GCC ATC CCG GAA GGG AAA CTG GAA GAA

ptsN_H73E-R	TTC TTC CAG TTT CCC TTC CGG GAT GGC GAT ACC
rpoN-pF1	CTG ATG GAA GAA TTC CAT ATC GA
rpoN-pR1	CTG CTG AGG ATC CAG CGT A
phoP-com-F2	GTT AAC AGA ATT CAG AAG AGA TGA TGC GC
phoP-com-R2	AAA ATG GCG AGC GGA TCC ATT CAT TAG C
phoP-F2	TTA ACA CAA GGA TCC AGA GAT GAT GC
phoP-R1	CAG CGG CAG AAT TCG GCG AG
pPhoP-F	GCG AGG ATC CAT GAT GCG CGT ACT GGT TGT AGA GGA TAA TGC ATT AT
pPhoP-His-R	CGA TAA GCT TCA TTA GTG GTG GTG GTG GTG GTG GCG CAA TTC AAA AAG ATA TCC TTG TCC GCG
phoQ-com-F	ATA TCT TTT TGA ATT CCG CTA ATG A
phoQ-com-R	ATT ATA ACG GAT CCT TAA CGA GA
PpagD-gfp-F	AAA GAA TTC AGC ACG CTT TAT TCC CGC TC
PpagD-gfp-R	AAA GGA TCC AGC ACC TCC TGT TAT ATA TA
pPmrA-F	GCG AGG ATC CAT GAA GAT ACT GAT TGT TGA AGA CGA CAC GCT ATT
pPmrA-His-R	CGA TAA GCT TAC TTA GTG GTG GTG GTG GTG GTG GCT TTC CTC AGT GGC AAC CAG CAT GTA

**for primer extension and EMSA**	
ptsN-P1	TCC TGG TTA AGT ACA CTG CTC AGT TGT AGA
ptsN-PE-F	GTT GCG AAG TAC CGA GAG TCT TTA TC
ptsN-PE-R	CGT GTC AGA ATG GCT TCA AAC ACG A
EMSA-pagD-F	AGC ACG CTT TAT TCC CGC T
EMSA-pagD-R2	TTT CAT AGC ACC TCC TGT TAT AT
EMSA-pbgP-F	TCG CCG GAC GGG AGA AAG GC
EMSA-pbgP-R	CAT TAA CCT CTC AGG CAG AC

References

1. Lucas RL, Lee CA. 2000. Unravelling the mysteries of virulence gene regulation in *Salmonella* Typhimurium. Mol Microbiol 36:1024-33.
2. Choi J, Shin D, Yoon H, Kim J, Lee CR, Kim M, Seok YJ, Ryu S. 2010. *Salmonella* pathogenicity island 2 expression negatively controlled by EIIA^{Ntr}-SsrB interaction is required for *Salmonella* virulence. Proc Natl Acad Sci U S A 107:20506-11.
3. Hanahan D. 1983. Studies on transformation of *Escherichia coli* with plasmids. J Mol Biol 166:557-80.

4. Karimova G, Pidoux J, Ullmann A, Ladant D. 1998. A bacterial two-hybrid system based on a reconstituted signal transduction pathway. Proc Natl Acad Sci U S A 95:5752-6.

5. Datsenko KA, Wanner BL. 2000. One-step inactivation of chromosomal genes in *Escherichia coli* K-12 using PCR products. Proc Natl Acad Sci U S A 97:6640-5.

6. Merighi M, Ellermeier CD, Slauch JM, Gunn JS. 2005. Resolvase-*in vivo* expression technology analysis of the *Salmonella enterica* serovar Typhimurium PhoP and PmrA regulons in BALB/c mice. J Bacteriol 187:7407-16.

7. Simons RW, Houman F, Kleckner N. 1987. Improved single and multicopy *lac*-based cloning vectors for protein and operon fusions. Gene 53:85-96.

8. Soncini FC, Vescovi EG, Groisman EA. 1995. Transcriptional autoregulation of the *Salmonella* Typhimurium *phoPQ* operon. J Bacteriol 177:4364-71.

9. Valdivia RH, Falkow S. 1996. Bacterial genetics by flow cytometry: rapid isolation of *Salmonella* Typhimurium acid-inducible promoters by differential fluorescence induction. Mol Microbiol 22:367-78.

10. Karimova G, Ullmann A, Ladant D. 2001. Protein-protein interaction between *Bacillus stearothermophilus* tyrosyl-tRNA synthetase subdomains revealed by a bacterial two-hybrid system. J Mol Microbiol Biotechnol 3:73-82.
